# Palladium-catalyzed difluoromethylation of heteroaryl chlorides, bromides and iodides†
†Electronic supplementary information (ESI) available. See DOI: 10.1039/c7sc00691h



**DOI:** 10.1039/c7sc00691h

**Published:** 2017-04-24

**Authors:** Changhui Lu, Yang Gu, Jiang Wu, Yucheng Gu, Qilong Shen

**Affiliations:** a Key Laboratory of Organofluorine Chemistry , Shanghai Institute of Organic Chemistry , University of Chinese Academy of Sciences , Chinese Academy of Sciences , 345 Lingling Road , Shanghai 200032 , China . Email: shenql@sioc.ac.cn; b Syngenta , Jealott's Hill International Research Centre Bracknell , Berkshire RG42 6EY , UK

## Abstract

A palladium-catalyzed difluoromethylation of a series of heteroaryl chlorides, bromides and iodides under mild conditions is described. A wide range of heteroaryl halides such as pyridyl, pyrimidyl, pyrazyl, funanyl, thienyl, pyazolyl, imidazolyl, thiazolyl, and oxazolyl halides were efficiently difluoromethylated, thus providing medicinal chemists an alternative choice for the preparation of drug candidates with the difluoromethylated heteroarene unit.

## Abstract 2

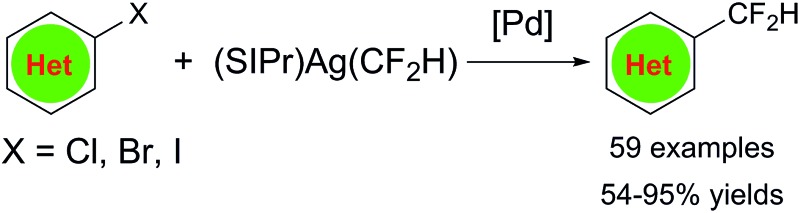
A palladium-catalyzed difluoromethylation of a series of heteroaryl chlorides, bromides and iodides under mild conditions is described.

## Introduction

**Fig. 1:**
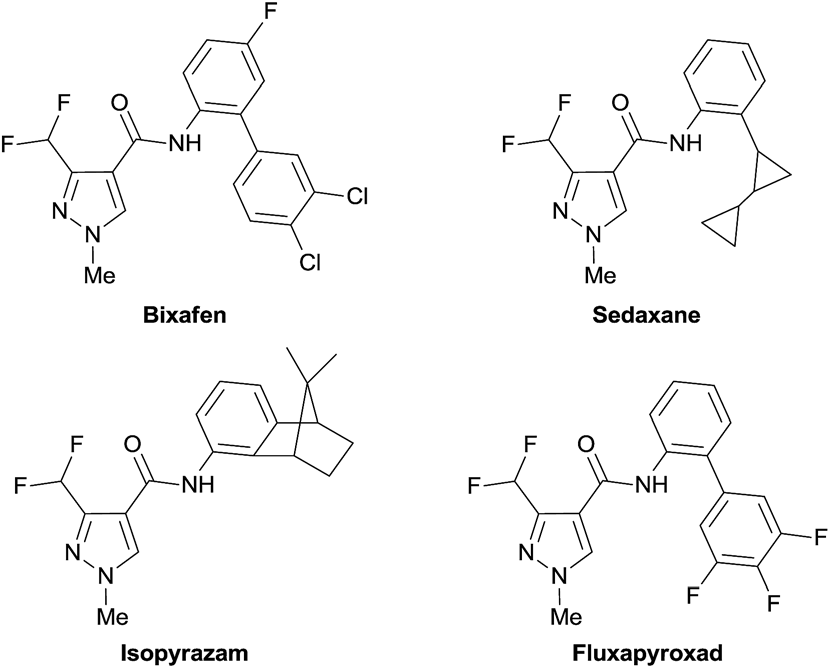
Fungicides with 3-difluoromethylpyrazole core structural unit.

Owing to the increased acidity of the proton in the difluoromethyl group that may interact with the targeting enzyme through hydrogen bonding, the difluoromethyl group (–CF_2_H), an analog of the well-recognized trifluoromethyl group (–CF_3_) in drug design,^1^ is generally considered by medicinal chemists as a bioisostere of a hydroxyl or a thiol group that will enhance the molecule's binding selectivity.^2^ On the other hand, in general, the heteroaryl moiety is regarded as one of the most common fragments in the majority of marketed drugs.^3^ As a logical consequence, difluoromethylated heteroarenes that combine beneficial properties from both units could be conceived as a promising family of pharmacores that are able to modulate the lipophilicity, polarity, and hydrogen bonding capacity of target molecules, and consequently the physiochemical and pharmacokinetics of drugs. One of several examples that support this point of view is the fact that 3-difluoromethylpyrazole^4^ is found to be the common core structural unit in four recently marketed fungicides including bixafen, sedaxane, isopyrazam and fluxapyroxad (Fig. 1). Thus, methods that can provide easy access to various difluoromethylated heteroarenes under mild conditions may aid medicinal chemists in their endeavor to hunt for novel lead compounds for new drug discoveries.^5^

In the past several decades, three different strategies have been reported for the preparation of difluoromethylated heteroarenes. The first general method to obtain difluoromethylated heteroarenes involved the use of fluorinated precursors and subsequent cyclocondensation of these building blocks with other coupling partners to give a specific difluoromethylated heteroarene and its derivatives. For example, Leroux successfully synthesized a series of difluoromethylated pyrazoles in excellent yields by 1,3-dipolar cyclo-addition of the easily available 1,1,2,2-tetrafluoro-*N*,*N*-dimethylethan-1-amine (TFEDMA) with hydrazine.^6^ This strategy is highly efficient for the preparation of one type of difluormethylated heteroarenes. Yet, the availability of the versatile difluoromethylated heteroarenes is limited. Alternatively, difluoromethylated heteroarenes may be accessed by direct radical difluoromethylation of heteroarenes with a radical difluoromethylating reagent. For example, in 2012, Baran^5^ reported that Zn(SO_2_CF_2_H)_2_ (DFMS) can difluoromethylate a variety of nitrogen-containing heteroarenes such as pyridines, pyrroles, pyrimidines, quinoxalines, pyrazines, xanthines, purines, quinoline, thiadiazoles, and pyridinones.^7^ These reactions were proposed to proceed *via* a radical pathway. Yet, regioselectivity in this method is problematic, which may cause difficulties in separation of the resulting isomers. A third strategy for the preparation of difluoromethylated heteroarenes relies on the transition metal-mediated or -catalyzed difluoromethylation of heteroaryl electrophiles, such as heteroaryl diazonium salts or halides, with an appropriate nucleophilic difluoromethylating reagent.^8^ While several transition metal-mediated/-catalyzed difluoromethylation of aryl halides have been reported to occur with broad scope, efforts for a similar transformation with heteroaryl electrophiles achieved only limited success. For example, Prakash and coworkers reported that copper-mediated difluoromethylation of 2-iodo-pyridine or 2-iodo-quinoline with Bu_3_SnCF_2_H was obtained in moderate to good yields, while similar transformation of more challenging heteroaryl halides such as 3-iodopyridine or halothiophenes were not described.^8*c*^ More recently, Vicic and coworkers reported the preparation of a new difluoromethylating reagent (DMPU)Zn(CF_2_H)_2_ that was allowed to couple with iodo- or bromo-substituted pyridine, quinoline, furan or thiophene in good yields in the presence of a nickel catalyst.^8*h*^ However, a high catalyst loading of nickel complex (15 mol%) was required and the turnover number of the catalytic reaction was not high enough. Shortly after, Mikami reported that (DMPU)Zn(CF_2_H)_2_ was able to couple with several activated heteroaryl iodides in the presence of 10 mol% of CuI, while reactions of non-activated heteroaryl iodides or heteroaryl bromides occurred with much less efficiency.^8*f*^ Later on, Mikami and coworkers developed (TMEDA)Zn(CF_2_H)_2_ that was allowed to couple with two activated heteroaryl iodides in high yields.^8*g*^ Clearly, new methods that allow the generation of a variety of difluoromethylated heteroarenes under mild conditions are still urgently needed.

Very recently, we developed a cooperative bimetallic Pd/Ag catalyst system that was quite efficient for the catalytic difluoromethylation of various aryl bromides and iodides with TMSCF_2_H.^9^ Nevertheless, our efforts to extend this catalyst system for the difluoromethylation of heteroaryl halides only resulted in moderate yields. We realized that to successfully develop efficient methods for the difluoromethylation of heteroaryl halides, we need to overcome two challenges: (1) reductive-elimination from a key product-forming intermediate [L_2_Pd(heteroaryl)(CF_2_H)] is much slower than that from [L_2_Pd(aryl)(CF_2_H)]; (2) the heteroatom may competingly coordinate to the palladium catalyst and consequently, the phosphino ligand in the catalyst may be replaced by the heteroarene substrates, which may lead to the deactivation of the catalyst.

In this report, we detailed the development of an effecient palladium-catalyst that overcomes these challenges and is capable of direct difluoromethylation of a vast range of different bromo- or iodo-substituted heteroarenes such as pyridine, pyrimidine, pyrole, funan, thiophene, quinoline, carbazole, dibenzo[*b*,*d*]thiophene, pyrazine, thiazole, oxazole, pyrazole and activated heteroaryl chlorides. Furthermore, the current method was successfully applied to the synthesis of three examples of difluoromethylated natural product derivatives.

## Results and discussion

**Fig. 2:**
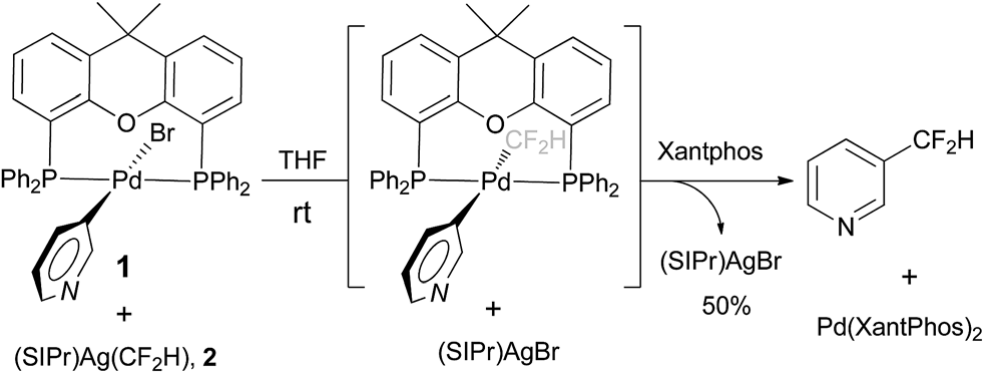
Stoichiometric reaction of complex [(xantphos)Pd(3-Py)(Br)] **1** with [(SIPr)Ag(CF_2_H)] **2** at room temperature.

In order to overcome the challenges associated with the palladium-catalyzed difluoromethylation of the heteroarene halides, we began our investigation by preparing the key intermediates and studying their elementary steps in the catalytic cycle. Our choice of xantphos as the ligand for the putative palladium intermediates was guided by previous observations in stoichiometric or catalytic organometallic chemistry: (1) reductive elimination is accelerated by wide bite angle bisphosphine ligands in various transition-metal-catalyzed C–C bond formation reactions.^10^ For example, Grushin reported that reductive-elimination from xantphos-ligated trifluoromethylated palladium complex [(xantphos)Pd(Ph)(CF_3_)] was much faster than those ligated with other bidendate ligands;^11^ (2) xantphos-ligated palladium complex [(xantphos)Pd(heteroaryl)(X)] (X = halide or amine) is able to resist the ligand replacement by the heteroaryl substrates. For example, Yin and coworkers have reported that palladium complex ligated with xantphos was able to efficiently catalyze the amination reactions of a broad range of heteroaryl halides even at high temperatures.^12^ Accordingly, [(xantphos)Pd(3-Py)(Br)] was prepared by following a known procedure.^11^ Interestingly, mixing a 1/1 ratio of [(xantphos)Pd(3-Py)(Br)] and [(SIPr)Ag(CF_2_H)] (SIPr = 1,3-bis(2,6-diisopropyl phenyl)imidazolin-2-ylidene), a nucleophilic difluoromethylating reagent which was isolated in our laboratory previously, generated the reductive-elimination product 3-difluoromethylpyridine in 50% yield after 1 h at room temperature, as determined by ^19^F NMR spectroscopy Fig. 2. These results clearly suggest that C–CF_2_H bond-forming reductive elimination from [(xantphos)Pd(3-Py)(CF_2_H)] is a facile process even at room temperature and likely not the rate-limiting step of the catalytic reaction.

**Scheme 1:**
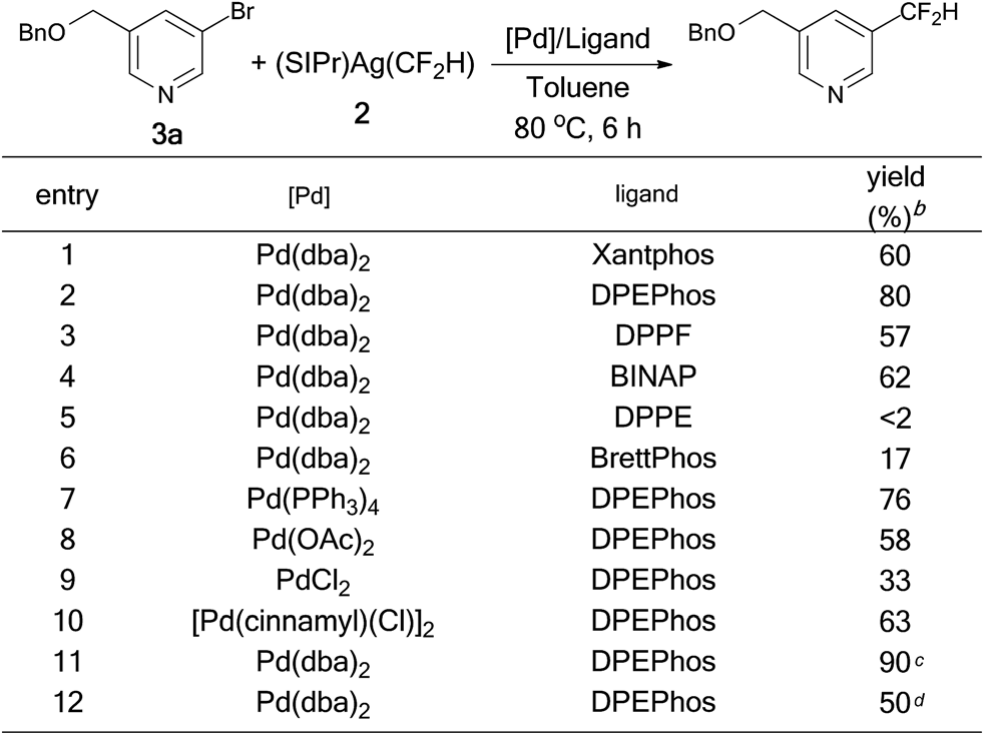
Optimization conditions for palladium-catalyzed difluoromethylation of 3-((benzyloxy)methyl)-5-bromopyridine. ^a^Reaction condition: **3a** (27.7 mg, 0.1 mmol), (SIPr)Ag(CF_2_H) **2** (55 mg, 0.1 mmol), [Pd] (5 mol%) and ligand (10 mol%) in 1.0 mL toluene for 6 h under Ar atmosphere; ^b^yields were determined by ^19^F NMR analysis of the crude reaction mixture with trifluorotoluene as an internal standard; ^c^1.3 equiv. of (SIPr)Ag(CF_2_H) was used; ^d^[Pd] (2.0 mol%) and DPEPhos (4.0 mol%).

Having established that reductive elimination from xantphos-ligated complex [(Xantphos)Pd(3-Py)(CF_2_H)] proceeds smoothly even at room temperature, we explored the cooperative bimetallic Pd/Ag catalyst system using xantphos as the ligand for the reaction of 3-((benzyloxy)methyl)-5-bromopyridine with TMSCF_2_H with NaO^*t*^Bu as the base. However, low yield was observed, likely due to the incompatibility of the elementary reactions in two catalytic cycles. Instead, when [(SIPr)Ag(CF_2_H)] was used as the nucleophilic difluoromethylation reagent, the formation of the desired product was observed in 60% yield after 6 h at 80 °C when a combination of 5.0 mol% Pd(dba)_2_ and 10.0 mol% xantphos was used as the catalyst (Scheme 1, entry 1). The yield was improved to 80% when another bidentate bisphosphino ligand with a wide bite angle DPEPhos was used as the ligand (Scheme 1, entry 2). In contrast, catalysts generated from ligands with smaller bite angle ligand such as DPPF, BINAP, DPPE were less effective, forming the desired product in 65%, 17% and less than 2% yields, respectively (Scheme 1, entries 3–5). Likewise, catalyst derived from sterically hindered, electron-rich, mono-dentate phosphine brettphos was also less effective (Scheme 1, entry 6). Next, we studied the effect of the different palladium precursors on the palladium-catalyzed difluoromethylation of the heteroaryl bromide. It turned out that reactions occurred in lower yields when catalysts derived from Pd(PPh_3_)_4_, Pd(OAc)_2_, PdCl_2_ or [Pd(cinnamyl)(Cl)]_2_ were used (Scheme 1, entries 7–10). Finally, the yield of the desired product was further improved to 90% when 1.3 equivalents of [(SIPr)Ag(CF_2_H)] was used (Scheme 1, entry 11), while efforts to decrease the catalyst loading were ineffective. Lower yield (50%) was observed when 2.0 mol% Pd(dba)_2_/4.0 mol% DPEPhos was used as the catalyst (Scheme 1, entry 12).

**Scheme 2:**
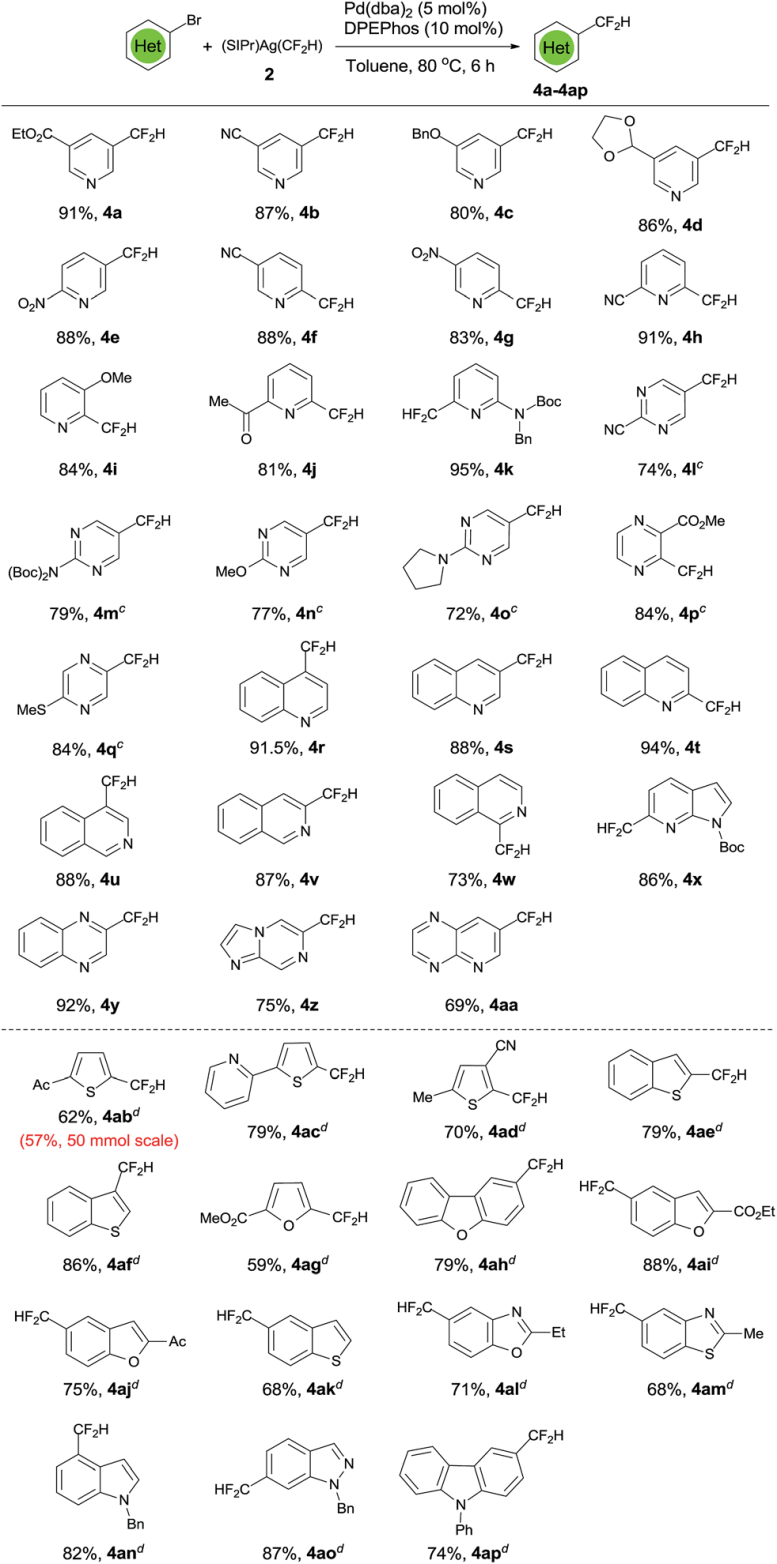
Scope of the palladium-catalyzed difluoromethylation of heteroaryl bromides. ^a^Reaction conditions: heteroaryl bromide (0.5 mmol), (SIPr)Ag(CF_2_H) **2** (0.65 mmol), Pd(dba)_2_ (5.0 mol%), DPEPhos (10 mol%) in 2.0 mL toluene at 80 °C for 6–12 h under an argon atmosphere; ^b^isolated yields; ^c^Pd(dba)_2_ (10 mol%) and DPEPhos (20 mol%) are used; ^d^reactions conducted with Pd(dba)_2_ (10 mol%) and DPEPhos (20 mol%) for 24 h.

With the above optimized reaction conditions, we first studied the difluoromethylation of an array of heteroaryl bromides to investigate the generality and scope of the reaction. As summarized in Scheme 2, a wide range of electron-deficient bromo-substituted nitrogen containing heteroarenes such as pyridine (**3a–k**), pyrimidine (**3l–o**), pyrazine (**3p–q**), quinoline (**3r–t**), isoquinoline (**3u–w**), 1*H*-pyrrolo[2,3-*b*]pyridine (**3x**), quinoxaline (**3y**), imidazo[1,2-*a*]pyrazine (**3z**), pyrido[2,3-*b*]pyrazine (**3aa**) underwent difluoromethylation smoothly under mild conditions to produce the corresponding difluoromethylated heteroarenes **4a–4aa** in high yields. Remarkably, these reaction conditions can also be applied to electron-rich bromo-substituted heteroarenes that are typically more challenging substrates due to slow oxidative addition/reductive-elimination, although reactions of these bromo-heteroarenes are slower (24 h) and require higher catalyst loading (10 mol% Pd(dba)_2_ and 20 mol% DPEPhos) to proceed to full conversion. Nevertheless, good to excellent yields for the formation of a variety of difluoromethylated electron-rich heteroarenes including thiophene (**4ab–ad**), benzothiophene (**4ae–4af**, **4ak**), furan (**4ag**), dibenzo[*b*,*d*]furan (**4ah**), benzofuran (**4ai–aj**), indole (**4an**), benzo[*d*]oxazole (**4al**), benzo[*d*]thiazole (**4am**), indazole (**4ao**) and carbazole (**4ap**) were achieved. Notably, bromo-substituted heteroarenes with functional groups such esters, cyano, protected aldehyde, enolizable ketone, thioether, or Boc-protected amino group, all underwent smooth difluoromethylation, illustrating the good functional compatibility of the current method. Furthermore, the reaction is scalable. The reaction of 1.0 g of 1-(5-bromothiophen-2-yl)ethanone with [(SIPr)Ag(CF_2_H)] **2** formed 0.50 g of the corresponding product **4ab** in 57% yield under the standard conditions (Scheme 2, **4ab**).

**Scheme 3:**
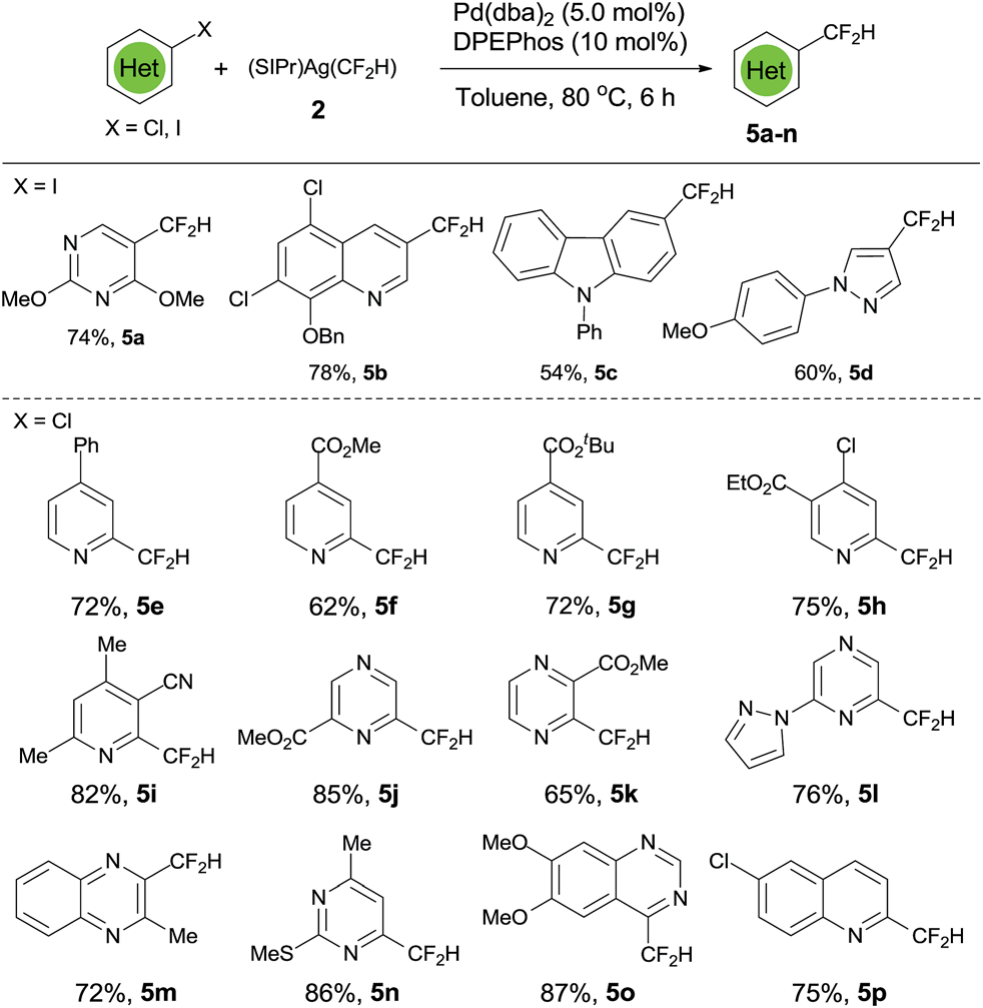
Scope of the palladium-catalyzed difluoromethylation of heteroaryl chlorides. ^a^Reaction conditions: heteroaryl chlorides (0.5 mmol), (SIPr)Ag(CF_2_H) **2** (0.65 mmol), Pd(dba)_2_ (5 mol%), DPEPhos (10 mol%) in toluene (2.5 mL) at 80 °C for 18 h; ^b^isolated yields.

In general, carbon–iodine bonds in heteroarenes are weaker than carbon–bromide bonds and the Pd-catalyzed cross-coupling reactions are easier. In fact, reactions of a few heteroaryl iodides with [(SIPr)Ag(CF_2_H)] **2** in the presence of 5.0 mol% or Pd(dba)_2_/10.0 mol% of DPEPhos occurred smoothly after 18 h at 80 °C to give the corresponding difluoromethylated heteroarenes in good to excellent yields (Scheme 3, **5a–d**). For example, reaction of 4-iodo-1-(4-methoxyphenyl)-1*H*-pyrazole with [(SIPr)Ag(CF_2_H)] **2** generated the corresponding 4-difluoromethyl-1-(4-methoxyphenyl)-1*H*-pyrazole **5d** in 60% yield, while efforts to convert its analog 3-iodo-1-(4-methoxyphenyl)-1*H*-pyrazole resulted in low yield (Scheme 3, **5d**). On the other hand, carbon–chloride bonds in heteroarenes are stronger than carbon–bromide bonds and the Pd-catalyzed cross-coupling reactions are much more difficult. Nevertheless, it was found that activated heteroaryl chlorides, in which the chlorine atom is at the *ortho*-position of the heteroatom in the heteroarene, coupled with [(SIPr)Ag(CF_2_H)] **2** under optimized conditions to give the corresponding difluoromethylated heteroarenes in high yields (Scheme 3, **5e–p**). When the carbon–chloride bond was at the *meta*- or *para*-position of the heteroatom in the heteroarene, the formation of the difluoromethyled heteroarene was not observed. For example, reaction of ethyl 4,6-dichloronicotinate with [(SIPr)Ag(CF_2_H)] **2** generated exclusively ethyl 4-chloro-6-difluoromethylnicotinate in 75% yield (Scheme 3, **5h**). The 4-difluoromethylated isomer was not observed. Control experiments in the absence of the palladium catalyst did not form the corresponding difluoromethylated product. To the best of our knowledge, this represents the first difluoromethylation reaction of heteroaryl chloride by any transition metal catalyst.

**Fig. 3:**
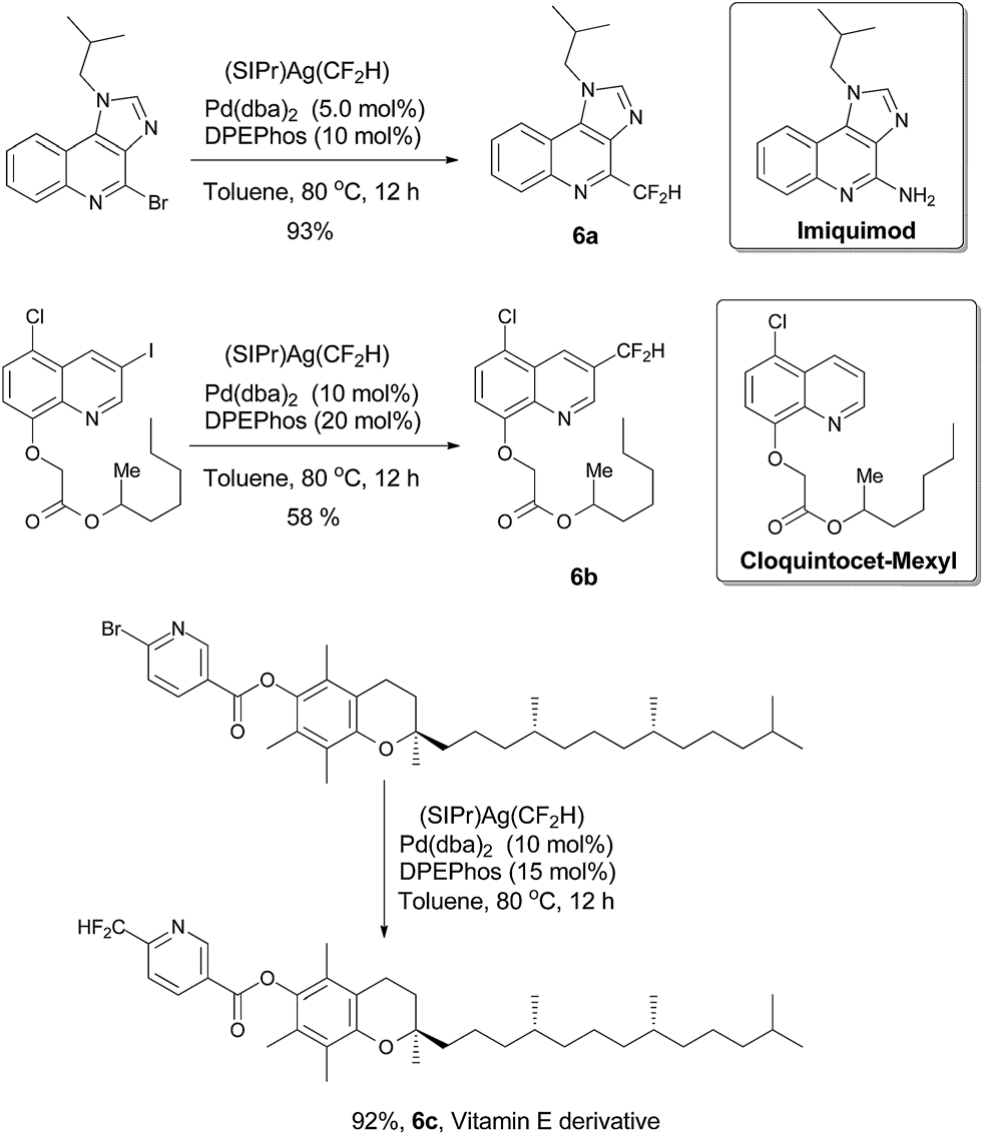
Preparation of difluoromethylated derivatives of drug compounds.

To demonstrate the applicability of this difuoromethylating protocol, we applied this method to the difuoromethylation of three medicinally important compounds. Compound **6a**, a difuoromethylthiolated analog of imiquimod,^13^ a medication that acts as an immune response modifier to treat genital warts, was generated in a 93% yield. Likewise, compound **6b**, a difluoromethylated derivative of herbicide safener cloquintocet-mexyl,^14^ was formed in 58% yield under standard reaction conditions. Furthermore, a difuoromethylated derivative of vitamin E was prepared from its bromo-substituted precursor in a 92% yield under standard reaction conditions (Fig. 3).

## Conclusions

In summary, we have developed a palladium-catalyzed direct difluoromethylation of heteroaryl chlorides, bromides, and iodides. The reaction was conducted under mild reaction conditions and several common functional groups were tolerated. Thus, the current method represents the first general method for the site-specific incorporation of difluoromethyl into heteroarenes. Currently, expansion of the reaction scope to aryl chlorides and unactivated heteroaryl chlorides are undergoing in our laboratory.
