# Supplementary material for: Palladium-catalyzed difluoromethylation of heteroaryl chlorides, bromides and iodides
Source: Chem Sci. 2017 Apr 24;8(7):4848–52. doi: 10.1039/c7sc00691h (PMC5603793; doi:10.1039/c7sc00691h)

Electronic Supplementary Material (ESI) for Chemical Science.
This journal is © The Royal Society of Chemistry 2017

Supporting Information

Table of Contents

General information 2
General procedure for preparation of [(SIPr)Ag(CF_2H)][1] 3
Synthesis of Starting Materials 4
General Procedure for Difluoromethylation of (Hetero-)Aryl Halides 10
Method A 10
Method B 10
Method C 10
Reference 42
^{1}H, ^{13}C and ^{19}F NMR Spectra of compound **4a-aq**, **5a-r**.................................43

General information

All solvents were purified by standard method. ^{1}H NMR spectra were recorded on a 500 MHz, 400 MHz or 300 MHz. ^{19}F NMR were recorded on a 376 MHz or 282 MHz spectrometer. ^{13}C NMR spectra were recorded on a Bruker AM400 spectrometer and Agilent 400 or 500 MHz spectrometer. ^{1}H NMR and ^{13}C NMR chemical shifts were determined relative to internal standard TMS at δ 0.0 and ^{19}F NMR chemical shifts were determined relative to $CFCl_3$ as inter standard. Chemical shifts (δ) are reported in ppm, and coupling constants (*J*) are in Hertz (Hz). The following abbreviations were used to explain the multiplicities: s = singlet, d = doublet, t = triplet, q = quartet, m = multiplet, br = broad. Flash column chromatograph was carried out using 300-400 mesh silica gel at medium pressure.

All reagents were received from commercial sources. Solvents were freshly dried and degassed according to the purification handbook *Purification of Laboratory Chemicals* before using.

General procedure for preparation of [(SIPr)Ag(CF_2H)][1]

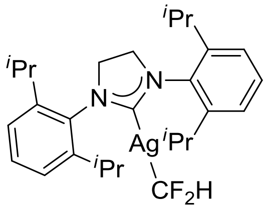

To a solution of [(SIPr)AgCl] (831 mg, 1.50 mmol) and NaO^{t}Bu (285 mg, 3.00 mmol) in THF (30 mL) was added TMSCF_2H (375 uL, 3.00 mmol). The resulting mixture was stirred for 1.5 h at ambient temperature. The mixture was filtered through a short plug with Celite and the solvent was evaporated under vacuum to give an off-white solid. The solid was recrystallized from CH_2Cl_2/pentane to give (1,3-bis(2,6-diisopropylphenyl)imidazolidin-2-yl)(difluoromethyl)silver [(SIPr)Ag(CF_2H)] as a white solid (698 mg, 82%). ^{1}H NMR (400 MHz, THF-d_8) δ 7.36 (t, J = 8.0 Hz, 2 H), 7.26 (d, J = 8.0 Hz, 4 H), 5.90 (td, J = 43.6, 14.0 Hz, 1 H), 4.04 (s, 4 H), 3.15 (hept, J = 6.8 Hz, 4 H), 1.34 (d, J = 7.2 Hz, 12 H), 1.32 (d, J = 7.2 Hz, 12 H); ^{19}F NMR (376 MHz,THF-d_8) δ -113.66 (dd, $J^{109}{}_{Ag\text{-}F}$ = 62.4 Hz, $J^{107}{}_{Ag\text{-}F}$ = 54.5 Hz, $J_{H\text{-}F}$ = 43.6 Hz); ^{13}C NMR (101 MHz, $CDCl_3$) δ 24.11, 25.76, 28.96, 124.55, 129.78, 134.80, 146.76, 153.67 (dt, $J^{109}{}_{Ag\text{-}C}$ = 260.6Hz, $J^{107}{}_{Ag\text{-}C}$ = 225.2 Hz, $J_{C\text{-}F}$ = 280.8 Hz), 211.41 (d, $J^{109}{}_{Ag\text{-}C}$ = 151.5 Hz, $J^{109}{}_{Ag\text{-}C}$ = 131.3 Hz) ppm. Anal. Calcd for $C_{28}H_{39}AgF_2N_2$: C, 61.20; H, 7.15; N, 5.10; Found: C, 60.91; H, 6.87; N, 4.78.

Synthesis of Starting Materials

8-(Benzyloxy)-5,7-dichloroquinoline[2,3]

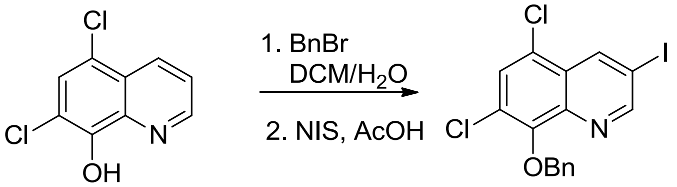

In a 500 mL round-bottom flask, the 5,7-dichloroquinolin-8-ol (8.5 g, 40 mmol), sodium hydroxide (2.4 g, 60 mmol) and *tetra*-butylammonium bromide (332 mg, 1.00 mmol) were dissolved in a mixture of dichloromethane (100.0 mL) and water (100.0 mL). Benzyl bromide (13.6 g, 80.0 mmol) was added dropwise to the solution. The mixture was stirred at room temperature for 4 h. The organic layer was separated and the aqueous layer extracted with dichloromethane (2 × 100 mL). The organic phase was combined and dried over anhydrous Na_2SO_4, and then concentrated in vacuo. The resulting residue was purified by flash chromatography (eluent: hexane / EtOAc = 20:1) to give the 8-(benzyloxy)-5,7- dichloroquinoline as a white solid (9.5 g, 78 %).

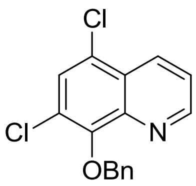

8-(Benzyloxy)-5,7-dichloroquinoline. ^{1}H NMR (400 MHz, $CDCl_3$) δ 9.02 (d, *J* = 4.1 Hz, 1 H), 8.52 (d, *J* = 8.6 Hz, 1 H), 7.64 (s, 1 H), 7.60 (d, *J* = 7.3 Hz, 2 H), 7.54 (dd, *J* = 8.5, 4.1 Hz, 1 H), 7.39 – 7.31 (m, 3 H), 5.47 (s, 2 H); ^{13}C NMR (101 MHz, $CDCl_3$) δ 150.77, 150.34, 143.90, 137.03, 133.33, 128.65, 128.35, 128.21, 127.88, 127.02, 126.28, 126.21, 122.02, 76.67 ppm. MS (EI): 91.1 (100), 303. HRMS (EI) for $C_{16}H_{11}Cl_2NO$ Calcd: 303.0218; Found: 303.0223. IR (KBr): ν = 3085, 3064, 3030, 2956, 1600, 1580, 1496, 1483, 1456, 1446, 1369, 1349, 1284, 1238, 1215, 1138, 1095, 1041, 990, 945, 883, 877, 807 cm^{-1}. Mp: 87.9 – 89.2 ^{o}C.

N-Iodo-succinimide (2.25 g, 10.0 mmol) was added in portions to a stirred solution of 8-(benzyloxy)-5,7-dichloroquinoline (3.00 g, 10.0 mmol) in acetic acid (10.0 mL) at 70 ℃ under argon. The mixture was heated to 70 ℃ for 18 h. After cooling to room temperature, the mixture was concentrated in vacuo. The residue was redissolved in

dichloromethane (50.0 mL) and the solution was washed successively with 10 % aqueous sodium thiosulfate solution (2 × 30 mL) and 10 % aqueous sodium hydrogen carbonate solution (2 × 30 mL), dried ($NaSO_4$) and then concentrated in vacuo. The resulting residue was purified by flash chromatography (eluent: hexane / EtOAc = 20:1) to give the 8-(benzyloxy)-5,7-dichloro-3- iodoquinoline as a white solid (1.4 g, 33 %).

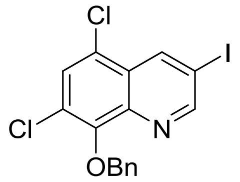

8-(Benzyloxy)-5,7-dichloro-3-iodoquinoline. ^{1}H NMR (400 MHz, $CDCl_3$) δ 9.13 (d, *J* = 1.9 Hz, 1 H), 8.88 (d, *J* = 2.0 Hz, 1 H), 7.63 (s, 1 H), 7.57 (d, *J* = 6.6 Hz, 2 H), 7.39 – 7.33 (m, 3 H), 5.44 (s, 2 H); ^{13}C NMR (101 MHz, $CDCl_3$) δ 156.06, 150.53, 142.00, 141.08, 136.75, 128.84, 128.62, 128.39, 128.32, 127.68, 127.55, 124.95, 91.59, 77.82 ppm. MS (EI): 91.1 (100), 429. HRMS (EI) for $C_{16}H_{10}Cl_2INO$ Calcd: 428.9184, Found: 428.9181. IR (KBr): ν = 3072, 3028, 2949, 1569, 1455, 1438, 1384, 1350, 1280, 1242, 1206, 1101, 1072, 940, 890, 762, 729, 693, 623 cm^{-1}. Mp: 130.8 – 132.4 °C.

4-Bromo-1-*iso*butyl-1H-imidazo[4,5-c]quinolone[4]

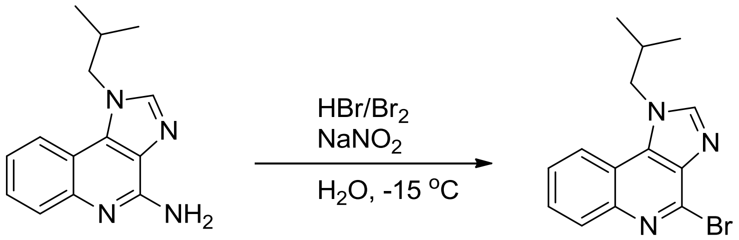

1-*iso*Butyl-1*H*-imidazo[4,5-c]quinolin-4-amine (932 mg, 3.88 mmol) was suspended in hydrobromic acid (5.0 mL, 48% in water), and the mixture was cooled to −15 ℃. Bromine (1.20 mL, 23.5 mmol) was added dropwise to the mixture followed by addition of sodium nitrite (1.50 g, 21.7 mmol) in water (2.0 mL). The reaction mixture was warmed to room temperature and was further stirred for 3 h. The reaction mixture was cooled to −15 ℃ and quenched with aqueous solution of potassium hydroxiode. The mixture was extracted with ethyl acetate (3 × 30 mL). The combined extracts were washed with water (2 × 20 mL), saturated aqueous sodium bicarbonate

(20 mL), dried with sodium sulfate, and concentrated in vacuo. The resulting residue was purified by flash chromatography (eluent: hexane / EtOAc = 10:1) to give the 4-bromo-1-*iso*butyl-1*H*-imidazo[4,5-c]quinoline as a white solid (732 mg, 66 %).

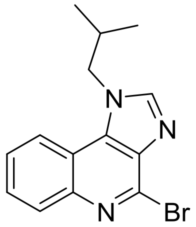

4-Bromo-1-*iso*butyl-1*H*-imidazo[4,5-c]quinolone. ^{1}H NMR (400 MHz, $CDCl_3$) δ 8.22 (d, *J* = 7.8 Hz, 1 H), 8.08 (d, *J* = 7.7 Hz, 1 H), 7.94 (s, 1 H), 7.71 – 7.64 (m, 2 H), 4.35 (d, *J* = 7.4 Hz, 2 H), 2.40 – 2.29 (m, 1 H), 1.04 (d, *J* = 6.6 Hz, 6 H). ^{13}C NMR (126 MHz, $CDCl_3$) δ 144.84, 143.94, 136.48, 132.93, 130.32, 127.97, 127.00, 120.16, 117.81, 109.99, 55.41, 28.83, 19.78 ppm. MS (EI): 303 (100), 305 (100). HRMS (EI) for $C_{14}H_{14}BrN_3$ Calcd: 303.0371; Found: 303.0377. IR (KBr): ν = 3092, 2962, 1740, 1561, 1390, 1354, 1218, 1137, 1077, 1014, 915, 773 cm^{-1}. Mp: 176.2 – 177.8 ^{o}C.

2,5,7,8-Tetramethyl-2-(4,8,12-trimethyltridecyl)chroman-6-yl 6-bromonicotinate

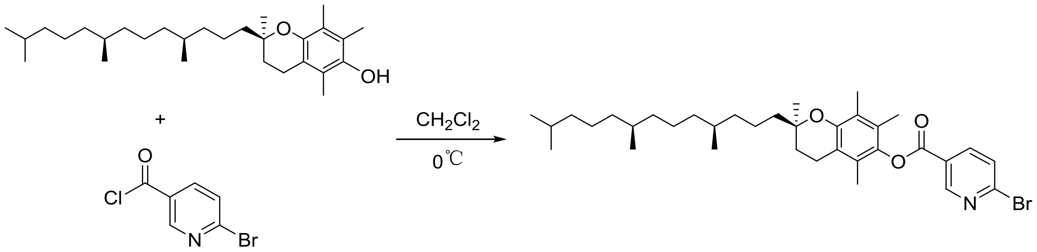

2,5,7,8-Tetramethyl-2-(4,8,12-trimethyltridecyl)chroman-6-ol (4.30 g, 10.0 mmol) was dissolved in anhydrous dicloromethane (20.0 mL), cooled to 0 ^{o}C, then 6-bromo nicotinoyl chloride was added dropwise to the mixture via syringe. The mixture was stirred for 1 h at 0 ^{o}C and for 1 h at room temperature. When the reaction was completed as monitored by TLC, the reaction was quenched. The mixture was exacted by dicloromethane for two or three times, dried with sodium sulfate, and concentrated in vacuo. The resulting residue was purified by flash chromatography (eluent: hexane / EtOAc = 5:1) to give the product as a yellow oil (6.14 g, 77 %).

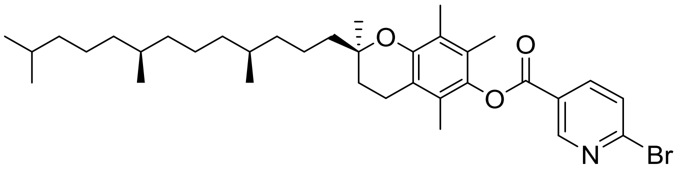

2,5,7,8-Tetramethyl-2-(4,8,12-trimethyltridecyl)chroman-6-yl 6-bromonicotinate

^{1}H NMR (400 MHz, $CDCl_3$) δ 9.23 (d, *J* = 4.0 Hz, 1 H), 8.44 (dd, *J* = 8.0 Hz, 1 H), 7.51 (d, *J* = 12.0 Hz, 1 H), 2.63 (t, *J* = 8.0 Hz, 2 H), 2.13 (s, 3 H), 2.05 (s, 3 H), 2.01 (s, 3 H), 1.1 - 1.9 (m, 27 H), 0.85 - 0.95 (m, 11 H); ^{13}C NMR (125 MHz, $CDCl_3$) δ 163.05, 156.20, 151.59, 149.76, 140.19, 140.05, 126.60, 124.89, 124.56, 124.43, 123.36, 117.64, 75.21, 39.39, 37.57, 37.54, 37.48, 37.43, 37.30, 32.80, 32.79, 27.99, 24.83, 24.46, 22.73, 22.64, 20.64, 21.05, 20.64, 19.77, 19.71, 13.07, 12.23, 11.88 ppm. MS (EI): 569 (100). IR (KBr): ν = 2926, 2867, 2250, 1739, 1587, 1563, 1458, 1415, 1377, 1240, 1105, 1017, 734 cm^{-1}.

Preparation of 2-((5-chloro-3-iodoquinolin-8-yl)oxy)acetate.

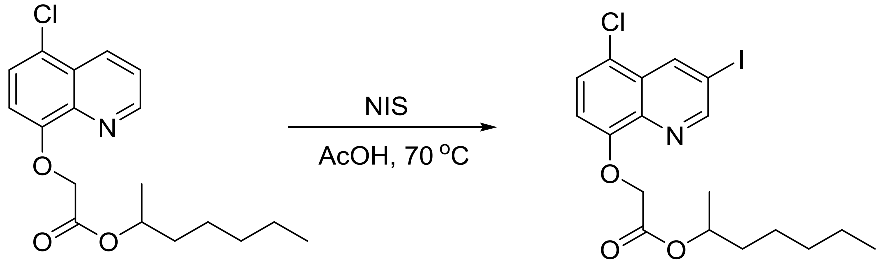

N-Iodo-succinimide (4.5 g, 20 mmol) was added in portions to a stirred solution of heptan-2-yl 2-((5-chloroquinolin-8-yl)oxy)acetate (6.7 g, 20 mmol) in acetic acid (20 mL) at 70 ℃ under argon. The mixture was heated to 70 ℃ for 18 h. After cooling to room temperature, the mixture was concentrated in vacuo. The residue was redissolved in dichloromethane (100 mL) and the solution was washed successively with 10 % aqueous sodium thiosulfate solution (2 × 60 mL) and 10 % aqueous sodium hydrogen carbonate solution (2 × 60 mL), dried (Na_2SO_4) and then concentrated in vacuo. The resulting residue was purified by flash chromatography (eluent: hexane / EtOAc = 20:1) to give the heptan-2-yl 2-((5-chloro-3-iodoquinolin-8-yl)oxy)acetate as a white solid (1.02 g, 11 %).

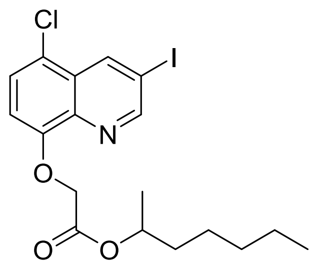

2-((5-Chloro-3-iodoquinolin-8-yl)oxy)acetate. ^{1}H NMR (400 MHz, $CDCl_3$) δ 9.09 (s, 1 H), 8.89 (s, 1 H), 7.49 (d, *J* = 8.4 Hz, 1 H), 6.89 (d, *J* = 8.4 Hz, 1 H), 5.07 – 4.96 (m, 1 H), 4.92 (s, 2 H), 1.56 – 1.42 (m, 2 H), 1.28 – 1.18 (m, 9 H), 0.87 – 0.82 (m, 3 H); ^{13}C NMR (101 MHz, $CDCl_3$) δ 167.91, 155.19, 153.11, 140.85, 138.76, 128.60, 127.06, 122.17, 109.95, 92.24, 72.83, 66.39, 35.65, 31.46, 24.88, 22.47, 19.86, 13.93 ppm. MS (EI): 318 (100), 461. HRMS (EI) for $C_{18}H_{21}ClINO_3$ Calcd: 461.0255; Found: 461.0253. IR (KBr): ν 2930, 1743, 1605, 1557, 1446, 1364, 1338, 1314, 1217, 1158, 1119, 947, 892, 834, 720 cm^{-1}. Mp: 66.8 – 68.2 °C.

Preparation of 1-(4-methoxyphenyl)-4-iodo-pyrazole.

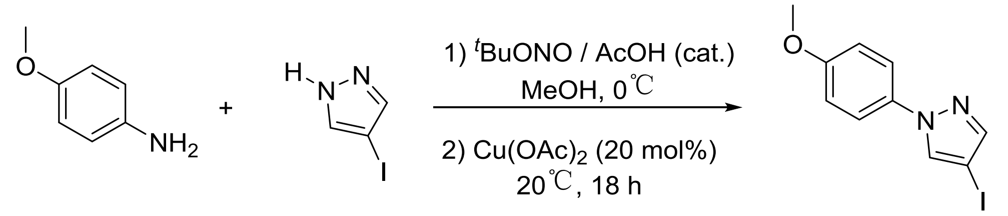

After standard cycles of evacuation and back-filling with dry and pure nitrogen, a Schlenk tube equipped with a magnetic stirring bar was charged with the 4-methoxyaniline (1.5 mmol) under an atmosphere of dry nitrogen. Methanol (3 mL) was added by syringe. The mixture was stirred, cooled, and ^{t}BuONO (1.1 equiv) and AcOH (20 mol%) were added dropwise by syringe. The resulting mixture was stirred for 30 min and added to a mixture of copper acetate (0.2 equiv, 36.4 mg) and the 4-iodo-1*H*-pyrazole (1 mmol) in methanol (1.5 mL). The reaction mixture was stirred at ambient temperature for up to 18 h. The resulting mixture was filtered through a pad of celite (5 g) and rinsed with ethyl acetate (20 mL). The resulting organic solution was washed with brine (3*10 mL). The organic layer was dried over $MgSO_4$, filtered, and concentrated. The residue was further purified by flash chromatography

to give the product as a white solid (150 mg, 50 %).

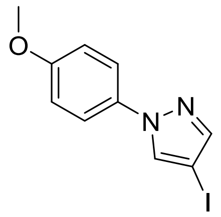

1-(4-methoxyphenyl)-4-indo-pyrazole. ^{1}H NMR (400 MHz, $CDCl_3$) δ 7.84 (s, 1 H), 7.67 (s, 1 H), 7.52 (d, *J* = 8.7 Hz, 2 H), 6.95 (d, *J* = 8.7 Hz, 2 H), 3.82 (s, 3 H) ; ^{13}C NMR (101 MHz, $CDCl_3$) δ 158.52, 145.46, 133.27, 131.24, 120.83, 114.55, 104.23, 55.56 ppm; MS (EI): 300 (100). HRMS (EI) for $C_{10}H_9N_2IO$ Calcd: 299.9763; Found: 299.9760. IR (KBr): ν = 3118, 2960, 1525, 1368, 1245, 1036, 1022, 952, 819, 651, 540 cm^{-1}.

General Procedure for Difluoromethylation of heteroaryl halides

Method A

In a Argon-filled glove box, heteroaryl halide (0.50 mmol, 1.0 equiv), Pd(dba)$_2$ (14 mg, 0.050 equiv), DPEPhos (28 mg, 0.10 equiv), and [(SIPr)Ag(CF$_2$H)] (357 mg, 1.30 equiv) were combined in a 20.0 mL vial. To this vial was added 2.0 mL of anhydrous toluene and the mixture was stirred at 80 ^{o}C for 6~12 h. The brown solution was diluted with Et$_2$O (10.0 mL), and filtered through a short plug of silica gel, washed with Et$_2$O (20.0 mL). The organic layer was combined and concentrated under vacuum. The crude product was purified by column chromatography on silica gel with pentane/ethyl acetate as the eluent to give the product.

Method B

In a Argon-filled glove box, heteroaryl halide (0.50 mmol, 1.0 equiv), Pd(dba)$_2$ (28 mg, 0.10 equiv), DPEPhos (56 mg, 0.20 equiv), and [(SIPr)Ag(CF$_2$H)] (357 mg, 1.30 equiv) were combined in a 20.0 mL vial. To this vial was added 2.0 mL of anhydrous toluene and the mixture was stired at 80 ^{o}C for 6~12 h. The brown solution was diluted with Et$_2$O (10.0 mL), and filtered through a short plug of silica gel, washed with Et$_2$O (20.0 mL). The organic layer was combined and concentrated under vacuum. The crude product was purified by column chromatography on silica gel with pentane/ethyl acetate as the eluent to give the product.

Method C

In a Argon-filled glove box, heteroaryl halide (0.50 mmol, 1.0 equiv), Pd(dba)$_2$ (28 mg, 0.10 equiv), DPEPhos (56 mg, 0.20 equiv), and [(SIPr)Ag(CF$_2$H)] (550 mg, 2.00 equiv) were combined in a 20.0 mL vial. To this vial was added 2.0 mL of anhydrous toluene and the mixture was stired at 80 ^{o}C for 24 h. The brown solution was diluted with Et$_2$O (10.0 mL), and filtered through a short plug of silica gel, washed with Et$_2$O (20.0 mL). The organic layer was combined and concentrated under vacuum. The crude product was purified by column chromatography on silica gel with pentane/ethyl acetate as the eluent to give the product.

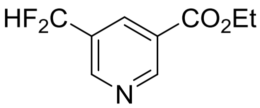

Ethyl 5-(difluoromethyl)-pyridine-3-carboxylate 4a. The general procedure **A** conducted with ethyl 5-bromo-pyridine-3-carboxylate (115 mg, 0.500 mmol), [(SIPr)Ag(CF_2H)] (357 mg, 0.650 mmol), Pd(dba)$_2$ (14 mg, 5.0 mol%), DPEPhos (27.0 mg, 10.0 mol%) in dry toluene (2.0 mL) gave 91 mg (90%) of ethyl 5-(difluoromethyl)-pyridine-3-carboxylate as a yellow oil. ^{1}H NMR (400 MHz, $CDCl_3$) δ 9.25 (s, 1 H), 8.85 (s, 1 H), 8.37 (s, 1 H), 6.72 (t, J = 55.5 Hz, 1 H), 4.37 (q, J = 7.1 Hz, 2 H), 1.36 (t, J = 7.1 Hz, 3 H); ^{19}F NMR (376 MHz, $CDCl_3$) δ -112.94 (d, J = 55.5 Hz, 2 F); ^{13}C NMR (126 MHz, $CDCl_3$) δ 164.31, 152.78, 150.55 (t, J = 6.4 Hz), 134.40 (t, J = 5.7 Hz), 130.01 (t, J = 23.5 Hz), 126.35, 112.85 (t, J = 240.3 Hz), 61.85, 14.16 ppm. MS (EI): 156 (100), 201 (47.23). HRMS (EI) for $C_9H_9N_2O_2F_2$ Calcd: 201.0601; Found: 201.0599. IR (KBr): ν = 2985, 1727, 1609, 1369, 1317, 1164, 1074, 913, 733 cm^{-1}.

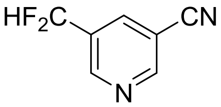

5-(Difluoromethyl)pyridine-3-carbonitrile 4b. The general procedure **A** conducted with 5-bromo-pyridine-3-carbonitrile (91 mg, 0.50 mmol), [(SIPr)Ag(CF_2H)] (357 mg, 0.650 mmol), Pd(dba)$_2$ (14 mg, 5.0 mol%), DPEPhos (27.0 mg, 10.0 mol%) in dry toluene (2.0 mL) gave 67 mg (87%) of 5-(difluoromethyl)pyridine-3-carbonitrile as a yellow solid. ^{1}H NMR (400 MHz, $CDCl_3$) δ 8.98 (s, 1 H), 8.93 (s, 1 H), 8.11 (s, 1 H), 6.75 (t, J = 55.3 Hz, 1 H); ^{19}F NMR (376 MHz, $CDCl_3$) δ -113.91 (d, J = 55.3 Hz, 2 F); ^{13}C NMR (126 MHz, $CDCl_3$) δ 154.26 (t, J = 1.7 Hz), 150.40 (t, J = 6.4 Hz), 136.71 (t, J = 5.8 Hz), 130.51 (t, J = 23.9 Hz), 115.60, 111.96 (t, J = 241.8 Hz), 110.35 ppm. MS (EI): 154 (100). HRMS (EI) for $C_7H_4N_2F_2$ Calcd: 154.0338; Found: 154.0340. IR (KBr): ν = 3039, 2919, 2241, 1603, 1572, 1434, 1222, 1041, 959, 746 cm^{-1}. Mp：58.1 – 59.4 ^{o}C.

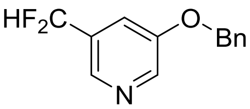

3-Benzyloxy-5-(difluoromethyl)pyridine 4c. The general procedure **A** conducted with 3-benzyloxy-5-bromo-pyridine (132 mg, 0.500 mmol), [(SIPr)Ag(CF_2H)] (357 mg, 0.650 mmol), Pd(dba)$_2$ (14 mg, 5.0 mol%), DPEPhos (27.0 mg, 10.0 mol%) in dry toluene (2.0 mL) gave 94 mg (80%) of 3-benzyloxy-5-(difluoromethyl)pyridine as a yellow oil. ^{1}H NMR (400 MHz, $CDCl_3$) δ 8.49 (d, J = 2.7 Hz, 1 H), 8.35 (d, J = 1.2 Hz, 1 H), 7.63 – 7.32 (m, 6 H), 6.68 (t, J = 55.8 Hz, 1 H), 5.13 (s, 2 H); ^{19}F NMR (376 MHz, $CDCl_3$) δ -112.09 (d, J = 55.8 Hz, 2 F); ^{13}C NMR (126 MHz, $CDCl_3$) δ 154.79, 141.07 (t, J = 2.0 Hz), 139.49 (t, J = 7.0 Hz), 135.53, 130.56 (t, J = 22.8 Hz), 128.78, 128.50, 127.58, 117.96 (t, J = 5.5 Hz), 113.12 (t, J = 239.8 Hz), 70.52 ppm. MS (EI): 91 (100), 235 (28.14). HRMS (EI) for $C_{13}H_{11}NOF_2$ Calcd: 235.0809; Found: 235.0813. IR (KBr): ν = 3066, 2935, 2877, 1597, 1497, 1375, 1288, 1028, 737 cm^{-1}.

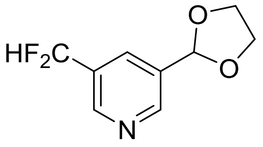

3-(Difluoromethyl)-5-(1,3-dioxolan-2-yl)pyridine 4d. The general procedure **A** conducted with 3-bromo-5-(1,3-dioxolan-2-yl)pyridine (115 mg, 0.500 mmol), [(SIPr)Ag(CF_2H)] (357 mg, 0.650 mmol), Pd(dba)$_2$ (14 mg, 5.0 mol%), DPEPhos (27.0 mg, 10.0 mol%) in dry toluene (2.0 mL) gave 86 mg (86%) of 3-(difluoromethyl)-5-(1,3-dioxolan-2-yl)pyridine as a yellow oil. ^{1}H NMR (400 MHz, $CDCl_3$) δ 8.79 (s, 1 H), 8.72 (s, 1 H), 7.92 (s, 1 H), 6.70 (t, J = 55.7 Hz, 1 H), 5.87 (s, 1 H), 4.69 – 3.78 (m, 4 H); ^{19}F NMR (376 MHz, $CDCl_3$) δ -112.59 (d, J = 55.8 Hz, 2 F); ^{13}C NMR (126 MHz, $CDCl_3$) δ 150.49 (t, J = 1.9 Hz), 147.72 (t, J = 6.6 Hz), 133.98, 131.52 (t, J = 5.6 Hz), 129.82 (t, J = 23.1 Hz), 113.21 (t, J = 239.7 Hz), 101.27, 65.46 ppm. MS (EI): 200 (100), 201 (42.95). HRMS (EI) for $C_9H_9NO_2F_2$ Calcd: 201.0601; Found: 201.0598. IR (KBr): ν = 3408, 2965, 2895, 2363, 2253, 1662, 1374, 1311, 1177, 1097, 903, 733 cm^{-1}.

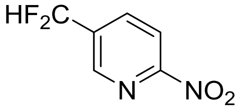

5-(Difluoromethyl)-2-nitro-pyridine 4e. The general procedure **A** conducted with 5-bromo-2-nitro-pyridine (101 mg, 0.500 mmol), [(SIPr)Ag(CF_2H)] (357 mg, 0.650

mmol), $Pd(dba)_2$ (14 mg, 5.0 mol%), DPEPhos (27.0 mg, 10.0 mol%) in dry toluene (2.0 mL) gave 76 mg (88%) of 5-(difluoromethyl)-2-nitro-pyridine as a yellow oil. ^{1}H NMR (400 MHz, $CDCl_3$) δ 8.93 (s, 1 H), 8.15 (d, *J* = 8.3 Hz, 1 H), 7.79 (d, *J* = 8.2 Hz, 1 H), 6.67 (t, *J* = 55.1 Hz, 1 H); ^{19}F NMR (376 MHz, $CDCl_3$) δ -114.02 (d, *J* = 55.1 Hz, 2 F); ^{13}C NMR (126 MHz, $CDCl_3$) δ 157.65, 146.75 (t, *J* = 6.6 Hz), 137.77 (t, *J* = 5.5 Hz), 135.45 (t, *J* = 23.6 Hz), 118.11, 111.90 (t, *J* = 241.6 Hz) ppm. MS (EI): 128 (100), 174 (1.77). HRMS (EI) for $C_6H_4N_2O_2F_2$ Calcd: 174.0241; Found: 174.0242. IR (KBr): ν = 3093, 2920, 2002, 1541, 1466, 1365, 1081, 1023, 953, 831, 678 cm^{-1}.

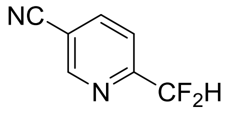

6-(Difluoromethyl)pyridine-3-carbonitrile 4f. The general procedure **A** conducted with 6-bromo-pyridine-3-carbonitrile (91.5 mg, 0.500 mmol), [(SIPr)Ag(CF_2H)] (357 mg, 0.650 mmol), $Pd(dba)_2$ (14 mg, 5.0 mol%), DPEPhos (27.0 mg, 10.0 mol%) in dry toluene (2.0 mL) gave 67 mg (88%) of 6-(difluoromethyl)pyridine-3-carbonitrile as a yellow solid. ^{1}H NMR (400 MHz, $CDCl_3$) δ 8.93 (s, 1 H), 8.16 (d, *J* = 8 Hz, 1 H), 7.79 (d, *J* = 8 Hz, 1 H), 6.67 (t, *J* = 56 Hz, 1 H); ^{19}F NMR (376 MHz, $CDCl_3$) δ -117.12 (d, *J* = 54.9 Hz, 2 F); ^{13}C NMR (126 MHz, $CDCl_3$) δ 155.92 (t, *J* = 26.5 Hz), 152.10, 140.77, 120.25 (t, *J* = 3.1 Hz), 115.69, 112.80 (t, *J* = 241.9 Hz), 111.82 (t, *J* = 1.5 Hz) ppm. MS (EI): 154 (100). HRMS (EI) for $C_7H_4N_2F_2$ Calcd: 154.0343; Found: 154.0344. IR (KBr): ν = 3081, 2237, 1993, 1652, 1594, 1486, 1388, 1223, 1091, 889, 846 cm^{-1}. Mp：70.1 – 71.1 °C.

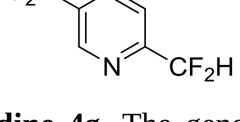

2-(Difluoromethyl)-5-nitro-pyridine 4g. The general procedure **A** conducted with 2-bromo-5-nitro-pyridine (101 mg, 0.500 mmol), [(SIPr)Ag(CF_2H)] (357 mg, 0.650 mmol), $Pd(dba)_2$ (14 mg, 5.0 mol%), DPEPhos (27.0 mg, 10.0 mol%) in dry toluene (2.0 mL) gave 72 mg (83%) of 2-(difluoromethyl)-5-nitro-pyridine as a yellow oil. ^{1}H NMR (400 MHz, $CDCl_3$) δ 9.47 (d, *J* = 1.8 Hz, 1 H), 8.65 (dd, *J* = 8.6, 2.4 Hz, 1 H), 7.88 (d, *J* = 8.6 Hz, 1 H), 6.72 (t, *J* = 54.8 Hz, 1 H); ^{19}F NMR (376 MHz, $CDCl_3$) δ -116.75 (d, *J* = 54.8 Hz, 2 F); ^{13}C NMR (126 MHz, $CDCl_3$) δ 157.61 (t, *J* = 26.5 Hz),

144.98, 144.91, 132.63, 120.70 (t, *J* = 3.0 Hz), 112.64 (t, *J* = 242.0 Hz) ppm. MS (EI): 174 (100). HRMS (EI) for $C_6H_4N_2O_2F_2$ Calcd: 174.0241; Found: 174.0237. IR (KBr): ν = 3105, 2363, 1608, 1588, 1537, 1355, 1223, 1094, 1053, 831, 724 cm^{-1}.

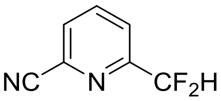

6-(Difluoromethyl)pyridine-2-carbonitrile 4h. The general procedure **A** conducted with 6-bromo-pyridine-2-carbonitrile (92 mg, 0.50 mmol), [(SIPr)Ag(CF_2H)] (357 mg, 0.650 mmol), Pd(dba)$_2$ (14 mg, 5.0 mol%), DPEPhos (27.0 mg, 10.0 mol%) in dry toluene (2.0 mL) gave 70 mg (91%) of 6-(difluoromethyl)pyridine-2-carbonitrile as a yellow solid. ^{1}H NMR (400 MHz, $CDCl_3$) δ 8.05 (t, *J* = 7.9 Hz, 1 H), 7.88 (d, *J* = 8.0 Hz, 1 H), 7.83 (d, *J* = 7.7 Hz, 1 H), 6.64 (t, *J* = 54.9 Hz, 1 H); ^{19}F NMR (376 MHz, $CDCl_3$) δ -116.21 (d, *J* = 54.8 Hz, 2 F); ^{13}C NMR (126 MHz, $CDCl_3$) δ 154.51 (t, *J* = 27.2 Hz), 138.84, 133.49, 129.90, 123.48, 116.28, 112.83 (t, *J* = 241.5 Hz) ppm. MS (EI): 154 (100). HRMS (EI) for $C_7H_4N_2F_2$ Calcd: 154.0343; Found: 154.0341. IR (KBr): ν = 3087, 2242, 1590, 1457, 1358, 1276, 1110, 1082, 1050, 826 cm^{-1}. Mp: 47.6 – 47.9 ^oC.

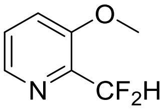

2-(Difluoromethyl)-3-methoxy-pyridine 4i. The general procedure **A** conducted with 2-bromo-3-methoxy-pyridine (94 mg, 0.50 mmol), [(SIPr)Ag(CF_2H)] (357 mg, 0.650 mmol), Pd(dba)$_2$ (14 mg, 5.0 mol%), DPEPhos (27.0 mg, 10.0 mol%) in dry toluene (2.0 mL) gave 66 mg (84%) of 2-(difluoromethyl)-3-methoxy-pyridine as a yellow oil. ^{1}H NMR (500 MHz, $CDCl_3$) δ 8.24 (dd, *J* = 4.6, 1.1 Hz, 1 H), 7.36 (dd, *J* = 8.5, 4.6 Hz, 1 H), 7.29 (d, *J* = 8.3 Hz, 1 H), 6.88 (t, *J* = 54.4 Hz, 1 H), 3.88 (s, 3 H); ^{19}F NMR (376 MHz, $CDCl_3$) δ -119.55 (d, *J* = 54.3 Hz, 2 F); ^{13}C NMR (126 MHz, $CDCl_3$) δ 154.18, 141.07, 140.84 (t, *J* = 22.6 Hz), 126.42 (t, *J* = 1.6 Hz), 119.02, 111.41 (t, *J* = 238.6 Hz), 55.53 ppm. MS (EI): 159 (100). HRMS (EI) for $C_7H_7NOF_2$ Calcd: 159.0496; Found: 159.0502. IR (KBr): ν = 3079, 2948, 1584, 1467, 1389, 1240, 1135, 1037, 871, 661 cm^{-1}.

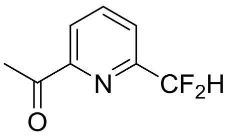

1-[6-(Difluoromethyl)-2-pyridyl]ethanone 4j. The general procedure **A** conducted with 1-[6-bromo-2-pyridyl]ethanone (100 mg, 0.500 mmol), [(SIPr)Ag(CF_2H)] (357 mg, 0.650 mmol), Pd(dba)$_2$ (14 mg, 5.0 mol%), DPEPhos (27.0 mg, 10.0 mol%) in dry toluene (2.0 mL) gave 69 mg (81%) of 1-[6-(difluoromethyl)-2-pyridyl]ethanone as a yellow oil. ^{1}H NMR (400 MHz, $CDCl_3$) δ 8.12 (d, *J* = 7.8 Hz, 1 H), 7.98 (t, *J* = 7.8 Hz, 1 H), 7.81 (d, *J* = 7.7 Hz, 1 H), 6.67 (t, *J* = 55.3 Hz, 1 H), 2.71 (s, 3 H); ^{19}F NMR (376 MHz, $CDCl_3$) δ -115.35 (d, *J* = 55.3 Hz, 2 F); ^{13}C NMR (126 MHz, $CDCl_3$) δ 199.18, 153.03, 152.30 (t, *J* = 26.5 Hz), 138.31, 123.36 (t, *J* = 2.6 Hz), 123.12 (t, *J* = 1.4 Hz), 113.76 (t, *J* = 240.6 Hz), 25.56 ppm. MS (EI): 171 (100). HRMS (EI) for $C_8H_7NOF_2$ Calcd: 171.0496; Found: 171.0499. IR (KBr): ν = 1702, 1589, 1437, 1364, 1295, 1227, 1118, 1097, 996, 822 cm^{-1}.

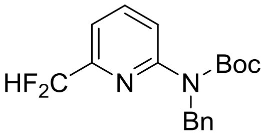

***tert*-Butyl *N*-benzyl-*N*-[6-(difluoromethyl)-2-pyridyl]carbamate 4k.** The general procedure **A** conducted with *tert*-butyl *N*-benzyl-*N*-[6-bromo-2-pyridyl]carbamate (182 mg, 0.500 mmol), [(SIPr)Ag(CF_2H)] (357 mg, 0.650 mmol), Pd(dba)$_2$ (14 mg, 5.0 mol%), DPEPhos (27.0 mg, 10.0 mol%) in dry toluene (2.0 mL) gave 158 mg (95%) of *tert*-butyl *N*-benzyl-*N*-[6-(difluoromethyl)-2-pyridyl]carbamate as a yellow solid. ^{1}H NMR (400 MHz, $CDCl_3$) δ 7.88 (d, *J* = 8.3 Hz, 1 H), 7.73 (t, *J* = 7.9 Hz, 1 H), 7.34 – 7.18 (m, 6 H), 6.49 (t, *J* = 55.6 Hz, 1 H), 5.22 (s, 2 H), 1.42 (s, 9 H); ^{19}F NMR (376 MHz, $CDCl_3$) δ -115.98 (d, *J* = 55.6 Hz, 2 F); ^{13}C NMR (126 MHz, $CDCl_3$) δ 154.14, 154.07, 150.58 (t, *J* = 26.2 Hz), 139.21, 138.16, 128.17, 127.46, 126.84, 120.67, 115.19 (t, *J* = 3.2 Hz), 113.69 (t, *J* = 240.4 Hz), 81.59, 49.71, 28.12 ppm. MS (EI): 145 (100), 334 (0.12). HRMS (EI) for $C_{18}H_{20}N_2O_2F_2$ Calcd: 334.1493; Found: 334.1487. IR (KBr): ν = 3011, 1716, 1595, 1581, 1497, 1430, 1285, 1110, 1034, 818, 705 cm^{-1}. Mp：42.1 – 44.2 ^{o}C.

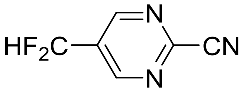

5-(Difluoromethyl)pyrimidine-2-carbonitrile 4l. The general procedure **B** conducted with 5-bromo-pyrimidine-2-carbonitrile (92 mg, 0.50 mmol), [(SIPr)Ag(CF_2H)] (357 mg, 0.650 mmol), Pd(dba)$_2$ (28.0 mg, 10.0 mol%), DPEPhos (56.0 mg, 20.0 mol%) in dry toluene (2.0 mL) gave 57 mg (74%) of 5-(difluoromethyl)pyrimidine-2-carbonitrile as a yellow oil. ^{1}H NMR (400 MHz, $CDCl_3$) δ 9.01 (s, 2 H), 6.85 (t, *J* = 52.0 Hz, 1 H); ^{19}F NMR (376 MHz, $CDCl_3$) δ -115.91 (d, *J* = 56.4 Hz, 2 F); ^{13}C NMR (125 MHz, $CDCl_3$) δ 150.61(t, *J* = 7.5 Hz), 141.29(t, *J* = 2.5 Hz), 124.85 (t, *J* = 30.0 Hz), 109.84, 105.81 (t, *J* = 243.2 Hz) ppm; MS (EI): 155 (100). HRMS (EI) for $C_6H_3N_3F_2$ Calcd: 155.0295; Found: 155.0299. IR (KBr): ν = 1589, 1562, 1424, 1365, 1226, 1093, 1037, 956, 860, 762, 658 cm^{-1}.

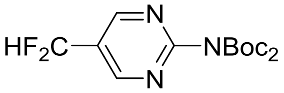

***tert*-Butyl-*N*-*tert*-butoxycarbonyl-*N*-[5-(difluoromethyl)pyrimidin-2-yl]carbamate 4m.** The general procedure **B** conducted with *tert*-butyl-*N*-*tert*-butoxycarbonyl -*N*-[5-bromo-pyrimidin-2-yl]carbamate (187 mg, 0.500 mmol), [(SIPr)Ag(CF_2H)] (357 mg, 0.650 mmol), Pd(dba)$_2$ (28.0 mg, 10.0 mol%), DPEPhos (56.0 mg, 20.0 mol%) in dry toluene (2.0 mL) gave 136 mg (79%) of *tert*-Butyl-*N*-*tert*-butoxycarbonyl-*N*-[5-(difluoromethyl)pyrimidin-2-yl]carbamate as a yellow solid. ^{1}H NMR (400 MHz, $CDCl_3$) δ 8.86 (s, 2 H), 6.77 (t, *J* = 56.0 Hz, 1 H), 1.48 (s, 18 H); ^{19}F NMR (376 MHz, $CDCl_3$) δ -113.82 (d, *J* = 56.4 Hz, 2 F); ^{13}C NMR (125 MHz, $CDCl_3$) δ 160.16, 156.36 (t, *J* = 6.3 Hz), 150.36, 125.36, 111.90 (t, *J* = 239.2 Hz), 84.03, 27.79 ppm; MS (EI): 57 (100); 345 (0.2). HRMS (EI) for $C_{15}H_{21}N_3O_4F_2$ Calcd: 345.1500; Found: 345.1505. IR (KBr): ν = 3052, 2982, 2936, 1762, 1738, 1600, 1478, 1268, 1125, 1033, 875, 778 cm^{-1}. Mp：67 – 68.1 ^{o}C.

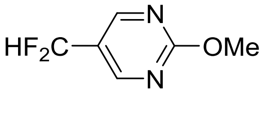

5-(Difluoromethyl)-2-methoxy-pyrimidine 4n. The general procedure **B** conducted with 5-bromo-2-methoxy-pyrimidine (94 mg, 0.50 mmol), [(SIPr)Ag(CF_2H)] (357 mg,

0.650 mmol), $Pd(dba)_2$ (28.0 mg, 10.0 mol%), DPEPhos (56.0 mg, 20.0 mol%) in dry toluene (2.0 mL) gave 61 mg (77%) of 5-(difluoromethyl)-2-methoxy-pyrimidine as a yellow solid. ^{1}H NMR (400 MHz, $CDCl_3$) δ 8.66 (s, 2 H), 6.70 (t, J = 56.0 Hz, 1 H), 4.06 (s, 3 H); ^{19}F NMR (376 MHz, $CDCl_3$) δ -111.88 (d, J = 52.6 Hz, 2 F); ^{13}C NMR (125 MHz, $CDCl_3$) δ 166.80, 157.39 (t, J = 5.1 Hz), 122.00 (t, J = 25.0 Hz), 112.32 (t, J = 248.0 Hz), 55.38 ppm; MS (EI): 130 (100); 160 (88.12). HRMS (EI) for $C_6H_6N_2OF_2$ Calcd: 160.0448; Found: 160.0449. IR (KBr): ν = 3051, 3009, 1700, 1485, 1447, 1411, 1371, 1197, 1080, 954, 864 cm^{-1}. Mp：55.1 – 56.6 ^{o}C.

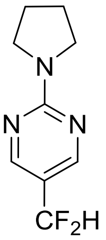

5-(Difluoromethyl)-2-pyrrolidin-1-yl-pyrimidine 4o. The general procedure **B** conducted with 5-bromo-2-pyrrolidin-1-yl-pyrimidine (114 mg, 0.500 mmol), [(SIPr)Ag(CF_2H)] (357 mg, 0.650 mmol), $Pd(dba)_2$ (28.0 mg, 10.0 mol%), DPEPhos (56.0 mg, 20.0 mol%) in dry toluene (2.0 mL) gave 70 mg (71%) of 5-(difluoromethyl) -2-pyrrolidin-1-yl-pyrimidine as a yellow solid. ^{1}H NMR (400 MHz, $CDCl_3$) δ 8.41 (s, 2 H), 6.56 (t, J = 56.0 Hz, 1 H), 3.60 (t, J = 6.0 Hz, 4 H), 2.01 (m, 4 H); ^{19}F NMR (376 MHz, $CDCl_3$) δ -109.45 (d, J = 56.4 Hz, 2 F); ^{13}C NMR (125 MHz, $CDCl_3$) δ 155.81 (t, J = 5.0 Hz), 115.47 (t, J = 23.8 Hz), 113.82 (t, J = 235. 5 Hz), 46.82, 25.40 ppm. MS (EI): 170 (100), 199 (48.38). HRMS (EI) for $C_9H_{11}N_3F_2$ Calcd: 199.0921; Found: 199.0913. IR (KBr): ν= 2976, 2874, 1608, 1545, 1477, 1291, 1241, 1066, 1006, 952, 825, 683 cm^{-1}. Mp：71.2 – 72 ^{o}C.

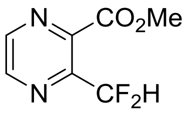

Methyl-3-(difluoromethyl)pyrazine-2-carboxylate 4p. The general procedure **B** conducted with methyl-3-bromo-pyrazine-2-carboxylate (108 mg, 0.500 mmol), [(SIPr)Ag(CF_2H)] (357 mg, 0.650 mmol), $Pd(dba)_2$ (28.0 mg, 10.0 mol%), DPEPhos (56.0 mg, 20.0 mol%) in dry toluene (2.0 mL) gave 79 mg (84%) of

methyl-3-(difluoromethyl)pyrazine -2-carboxylate as a yellow oil. ^{1}H NMR (400 MHz, $CDCl_3$) δ 9.38 (s, 1 H), 9.08 (s, 1 H), 6.78 (t, *J* = 52.0 Hz, 1 H), 4.02 (s, 3 H); ^{19}F NMR (376 MHz, $CDCl_3$) δ 8.95 (d, *J* = 52.6 Hz, 2 F); ^{13}C NMR (125 MHz, $CDCl_3$) δ 163.56, 147.67, 147.66 (t, *J* = 27 Hz), 144.98 (t, *J* = 3.0 Hz), 142.20，112.90 (t, *J* = 242.4 Hz), 53.39 ppm. MS (EI): 130 (100), 188 (33.56). HRMS (EI) for $C_7H_6N_2O_2F_2$ Calcd: 188.0397; Found: 188.0398. IR (KBr): ν = 3058, 2958, 2853, 2250, 1734, 1444, 1425, 1367, 1300, 1206, 1179, 912, 734 cm^{-1}.

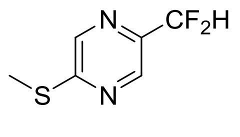

2-(difluoromethyl)-5-methylsulfanyl-pyrazine 4q. The general procedure **B** conducted with 2-bromo-5-methylsulfanyl-pyrazine (102 mg, 0.500 mmol), [(SIPr)Ag(CF_2H)] (357 mg, 0.650 mmol), Pd(dba)$_2$ (28.0 mg, 10.0 mol%), DPEPhos (56.0 mg, 20.0 mol%) in dry toluene (2.0 mL) gave 74 mg (84%) of 2-(difluoromethyl)-5- methylsulfanyl-pyrazine as a yellow oil. ^{1}H NMR (400 MHz, $CDCl_3$) δ 8.65 (s, 1 H), 8.45 (s, 1 H), 6.66 (t, *J* = 56.0 Hz, 1 H), 2.59 (s, 3 H); ^{19}F NMR (376 MHz, $CDCl_3$) δ -116.55 (d, *J* = 52.6 Hz, 2 F); ^{13}C NMR (125 MHz, $CDCl_3$) δ 155.22, 137.21, 137.11 (t, *J* = 26.3 Hz), 135.79 (t, *J* = 3.0 Hz), 108.15 (t, *J* = 241.4 Hz), 7.38 ppm. MS (EI): 176 (100). HRMS (EI) for $C_6H_6N_2F_2S$ Calcd: 176.0220; Found: 176.0225. IR (KBr): ν = 2960, 2868, 1491, 1458, 1362, 1210, 1157, 1082, 848, 772 cm^{-1}.

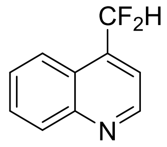

4-(Difluoromethyl)quinoline 4r. The general procedure **A** conducted with 4-bromo-quinoline (104 mg, 0.500 mmol), [(SIPr)Ag(CF_2H)] (357 mg, 0.650 mmol), Pd(dba)$_2$ (14 mg, 5.0 mol%), DPEPhos (28.0 mg, 10.0 mol%) in dry toluene (2.0 mL) gave 81 mg (91%) of 4-(difluoromethyl)quinoline as a yellow oil. ^{1}H NMR (400 MHz, $CDCl_3$) δ 9.02 (d, *J* = 4.3 Hz, 1 H), 8.21 (d, *J* = 8.5 Hz, 1 H), 8.09 (d, *J* = 8.4 Hz, 1 H), 7.80 (t, *J* = 7.7 Hz, 1 H), 7.66 (t, *J* = 7.7 Hz, 1 H), 7.59 (d, *J* = 4.2 Hz, 1 H), 7.16 (t, *J* = 54.5 Hz, 1 H); ^{19}F NMR (376 MHz, $CDCl_3$) δ -115.14 (d, *J* = 54.5 Hz, 2 F); ^{13}C

NMR (126 MHz, $CDCl_3$) δ 149.99, 148.59, 137.77 (t, *J* = 21.9 Hz), 130.41, 129.92, 127.81, 124.11 (t, *J* = 3.2 Hz), 123.28, 117.93 (t, *J* = 7.7 Hz), 113.27 (t, *J* = 240.4 Hz) ppm. MS (EI): 179 (100). HRMS (EI) for $C_{10}H_7F_2N$ Calcd: 179.0547; Found: 179.0543. IR (KBr): ν = 3853, 3079, 2966, 2040, 1741, 1375, 1041, 962, 750 cm^{-1}.

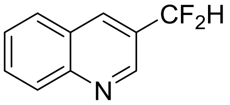

3-(Difluoromethyl)quinoline 4s. The general procedure **A** conducted with 3-bromo-quinoline (104 mg, 0.500 mmol), [(SIPr)Ag(CF_2H)] (357 mg, 0.650 mmol), $Pd(dba)_2$ (14 mg, 5.0 mol%), DPEPhos (28.0 mg, 10.0 mol%) in dry toluene (2.0 mL) gave 78 mg (88%) of 3-(difluoromethyl)quinoline as a yellow oil. ^1H NMR (400 MHz, $CDCl_3$) δ 9.01 (d, *J* = 4 Hz, 1 H), 8.26 (s, 1 H), 8.14 (d, *J* = 8.0 Hz, 1 H), 7.79 (d, *J* = 8.2 Hz, 1 H), 7.78 (t, *J* = 7.7 Hz, 1 H), 7.59 (t, *J* = 7.5 Hz, 1 H), 6.86 (t, *J* = 55.8 Hz, 1 H); ^{19}F NMR (376 MHz, $CDCl_3$) δ -111.56 (d, *J* = 55.8 Hz, 2 F); ^{13}C NMR (126 MHz, $CDCl_3$) δ 149.06, 147.12 (t, *J* = 5.3 Hz), 133.87 (t, *J* = 6.7 Hz), 131.03, 129.53, 128.37, 127.58, 127.10 (t, *J* = 22.7 Hz), 126.80, 113.72 (t, *J* = 239.4 Hz) ppm. MS (EI): 179 (100). HRMS (EI) for $C_{10}H_7F_2N$ Calcd: 179.0547; Found: 179.0550. IR (KBr): ν = 1625, 1610, 1499, 1395, 1180, 1140, 1089, 1034, 916, 791 cm^{-1}.

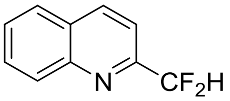

2-(Difluoromethyl)quinoline 4t. The general procedure **A** conducted with 2-bromo-quinoline (104 mg, 0.500 mmol), [(SIPr)Ag(CF_2H)] (357 mg, 0.650 mmol), $Pd(dba)_2$ (14 mg, 5.0 mol%), DPEPhos (28.0 mg, 10.0 mol%) in dry toluene (2.0 mL) gave 84 mg (94%) of 2-(difluoromethyl)quinoline as a yellow oil. ^1H NMR (400 MHz, $CDCl_3$) δ 8.33 (d, *J* = 8.5 Hz, 1 H), 8.15 (d, *J* = 8.5 Hz, 1 H), 7.87 (d, *J* = 8.3 Hz, 1 H), 7.77 (ddd, *J* = 8.4, 6.9, 1.4 Hz, 1 H), 7.73 (d, *J* = 8.5 Hz, 1 H), 7.63 (t, *J* = 7.5 Hz, 1 H), 6.79 (t, *J* = 55.3 Hz, 1 H); ^{19}F NMR (376 MHz, $CDCl_3$) δ -114.23 (d, *J* = 55.3 Hz, 2 F); ^{13}C NMR (126 MHz, $CDCl_3$) δ 152.71(t, *J* = 27.3 Hz), 147.12, 137.80, 130.30, 129.64, 128.69, 127.89, 127.72, 116.75, 114.63 (t, *J* = 240.6 Hz) ppm. MS (EI): 179 (100). HRMS (EI) for $C_{10}H_7F_2N$ Calcd: 179.0547; Found: 179.0548. IR (KBr): ν = 3066, 2962, 1717, 1647, 1540, 1372, 1260, 1122, 1039, 882, 767 cm^{-1}.

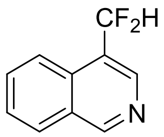

4-(Difluoromethyl)isoquinoline 4u. The general procedure **A** conducted with 4-bromoisoquinoline (104 mg, 0.500 mmol), [(SIPr)Ag(CF_2H)] (357 mg, 0.650 mmol), Pd(dba)$_2$ (14 mg, 5.0 mol%), DPEPhos (28.0 mg, 10.0 mol%) in dry toluene (2.0 mL) gave 78 mg (88%) of 4-(difluoromethyl)isoquinoline as a yellow oil. ^{1}H NMR (400 MHz, $CDCl_3$) δ 9.31 (s, 1 H), 8.63 (s, 1 H), 8.15 (d, J = 8.5 Hz, 1 H), 8.00 (d, J = 8.2 Hz, 1 H), 7.77 (t, J = 8.2 Hz, 1 H), 7.65 (t, J = 7.5 Hz, 1 H), 7.04 (t, J = 54.4 Hz, 1 H); ^{19}F NMR (376 MHz, $CDCl_3$) δ -110.99 (d, J = 54.4 Hz, 2 F); ^{13}C NMR (126 MHz, $CDCl_3$) δ 156.02, 141.66 (t, J = 9.4 Hz), 132.09, 131.62, 128.38, 128.36, 127.88, 123.20 (t, J = 21.6 Hz), 123.09 (t, J = 1.5 Hz), 115.06 (t, J = 238.4 Hz) ppm. MS (EI): 179 (100). HRMS (EI) for $C_{10}H_7F_2N$ Calcd: 179.0547; Found: 179.0543. IR (KBr): ν = 3060, 2967, 1624, 1593, 1458, 1258, 1174, 1079, 905, 883 cm^{-1}.

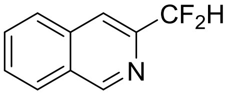

3-(Difluoromethyl)isoquinoline 4v. The general procedure **A** conducted with 3-bromo-isoquinoline (104 mg, 0.500 mmol), [(SIPr)Ag(CF_2H)] (357 mg, 0.650 mmol), Pd(dba)$_2$ (14 mg, 5.0 mol%), DPEPhos (28.0 mg, 10.0 mol%) in dry toluene (2.0 mL) gave 77 mg (87%) of 3-(difluoromethyl)isoquinoline as a yellow oil. ^{1}H NMR (400 MHz, $CDCl_3$) δ 9.22 (s, 1 H), 7.96 (d, J = 8.1 Hz, 1 H), 7.94 (s, 1 H), 7.85 (d, J = 8.2 Hz, 1 H), 7.72 (t, J = 7.5 Hz, 1 H), 7.65 (t, J = 7.5 Hz, 1 H), 6.80 (t, J = 55.7 Hz, 1 H); ^{19}F NMR (376 MHz, $CDCl_3$) δ -115.78 (d, J = 55.7 Hz, 2 F); ^{13}C NMR (126 MHz, $CDCl_3$) δ 152.71, 146.13 (t, J = 26.6 Hz), 135.59, 131.17, 129.16, 128.69, 127.65, 127.32, 117.66 (t, J = 4.3 Hz), 114.03 (t, J = 239.7 Hz) ppm. MS (EI): 179 (100). HRMS (EI) for $C_{10}H_7F_2N$ Calcd: 179.0547; Found: 179.0546. IR (KBr): ν = 3063, 2981, 1630, 1596, 1462, 1442, 1340, 1275, 1178, 1041, 950, 858, 687 cm^{-1}.

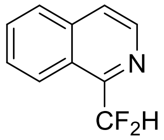

1-(Difluoromethyl)isoquinoline 4w. The general procedure **A** conducted with 1-bromoisoquinoline (104 mg, 0.500 mmol), [(SIPr)Ag(CF_2H)] (357 mg, 0.650 mmol), Pd(dba)$_2$ (14 mg, 5.0 mol%), DPEPhos (28.0 mg, 10.0 mol%) in dry toluene (2.0 mL) gave 65 mg (73%) of 1-(difluoromethyl)isoquinoline as a yellow oil. ^{1}H NMR (400 MHz,CDCl$_3$) δ 8.50 (t, *J* = 9.0 Hz, 2 H), 7.91 (d, *J* = 8.2 Hz, 1 H), 7.83 – 7.64 (m, 3 H), 6.98 (t, *J* = 54.4 Hz, 1 H); ^{19}F NMR (376 MHz, CDCl$_3$) δ -110.07 (d, *J* = 52.7 Hz, 2 F); ^{13}C NMR (126 MHz, CDCl$_3$) δ 151.30 (t, *J* = 26.5 Hz), 151.18, 142.51, 139.98, 127.28, 117.34 (t, *J* = 3.3 Hz), 114.15 (t, *J* = 1.6 Hz), 113.41 (t, *J* = 240.6 Hz), 108.27, 108.16 ppm. MS (EI): 179 (100). HRMS (EI) for $C_{10}H_7NF_2$ Calcd: 179.0547; Found: 179.0548. IR (KBr): ν = 2959, 2924, 1457, 1260, 1118, 1037, 895, 797, 662 cm^{-1}.

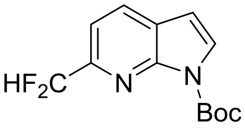

***tert*-Butyl 6-(difluoromethyl)pyrrolo[2,3-b]pyridine-1-carboxylate 4x.** The general procedure **A** conducted with tert-butyl 6-bromo-pyrrolo[2,3-b]pyridine-1-carboxylate (148 mg, 0.500 mmol), [(SIPr)Ag(CF_2H)] (357 mg, 0.650 mmol), Pd(dba)$_2$ (14 mg, 5.0 mol%), DPEPhos (28.0 mg, 10.0 mol%) in dry toluene (2.0 mL) gave 115 mg (86%) of *tert*-butyl 6-(difluoromethyl)pyrrolo[2,3-b]pyridine-1-carboxylate as a yellow oil. ^{1}H NMR (500 MHz, CDCl$_3$) δ 7.96 (d, *J* = 5 Hz, 1 H), 7.75 (d, *J* = 5 Hz, 1 H), 7.53 (d, *J* = 10 Hz, 1 H), 6.76 (t, *J* = 55 Hz, 1 H), 6.53 (d, *J* = 5 Hz, 1 H), 1.66 (s, 9 H); ^{19}F NMR (376 MHz, CDCl$_3$) δ -111.92 (d, *J* = 52.6 Hz, 2 F); ^{13}C NMR (125 MHz, CDCl$_3$) δ 147.89 (t, *J* = 6.3 Hz), 146.97, 130.06, 128.41, 124.74, 114.88 (t, *J* = 238.8 Hz), 114.75 (t, *J* = 2.5 Hz), 104.42, 84.56, 28.04 ppm. MS (EI): 168 (100), 268 (2.33). HRMS (EI) for $C_{13}H_{14}N_2O_2F_2$ Calcd: 268.1023; Found: 268.1014. IR (KBr): ν = 2982, 2934, 2247, 1738, 1525, 1458, 1340, 1260, 1035, 934, 846, 733 cm^{-1}.

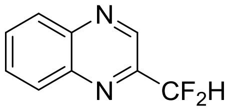

2-(Difluoromethyl)quinoxaline 4y. The general procedure **A** conducted with

2-bromoquinoxaline (104 mg, 0.500 mmol), [(SIPr)Ag(CF_2H)] (357 mg, 0.650 mmol), Pd(dba)$_2$ (14 mg, 5.0 mol%), DPEPhos (28.0 mg, 10.0 mol%) in dry toluene (2.0 mL) gave 96 mg (92%) of 2-(difluoromethyl)quinoxaline as a yellow solid. ^{1}H NMR (400 MHz, $CDCl_3$) δ 9.18 (s, 1 H), 8.40 – 8.04 (m, 2 H), 7.99 – 7.60 (m, 2 H), 6.84 (t, *J* = 54.6 Hz, 1 H); ^{19}F NMR (376 MHz, $CDCl_3$) δ -115.26 (d, *J* = 54.6 Hz, 2 F); ^{13}C NMR (126 MHz, $CDCl_3$) δ 147.11, 143.44, 141.71 (t, *J* = 2.4 Hz), 141.07, 131.49, 131.01, 129.52, 114.00 (t, *J* = 241.0 Hz), 109.97 ppm. MS (EI): 180 (100). HRMS (EI) for $C_9H_6N_2F_2$ Calcd: 180.0495; Found: 180.0494. IR (KBr): ν = 3058, 3001, 1972, 1640, 1496, 1386, 1260, 1138, 973, 885 cm^{-1}. Mp：44.6– 46.2 ^{o}C.

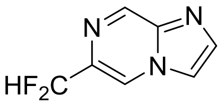

6-(Difluoromethyl)imidazo[1,2-a]pyridine 4z. The general procedure **A** conducted with 6-bromo-imidazo[1,2-a]pyridine (99 mg, 0.5 mmol), [(SIPr)Ag(CF_2H)] (357 mg, 0.650 mmol), Pd(dba)$_2$ (14 mg, 5.0 mol%), DPEPhos (28.0 mg, 10.0 mol%) in dry toluene (2.0 mL) gave 63 mg (75%) of 6-(difluoromethyl)imidazo[1,2-a]pyridine as a yellow solid. ^{1}H NMR (400 MHz, $CDCl_3$) δ 9.10 (d, *J* = 8.0 Hz, 1 H), 8.46 (d, *J* = 6.8 Hz, 1 H), 7.89 (d, *J* = 3.6 Hz, 1 H), 7.83 (d, *J* = 5.2 Hz, 1 H), 6.77 (t, *J* = 52.0 Hz, 1 H); ^{19}F NMR (376 MHz, $CDCl_3$) δ -117.43 (d, *J* = 56.4 Hz, 2 F); ^{13}C NMR (125 MHz, $CDCl_3$) δ 141.12, 140.31, 134.36, 132.16 (t, *J* = 25.0 Hz), 120.53, 116.75, 110.29 (t, *J* = 250.0 Hz) ppm; MS (EI): 169 (100). HRMS (EI) for $C_7H_5N_3F_2$ Calcd: 169.0452; Found: 169.0450. IR (KBr): ν = 3134, 1523, 1470, 1394, 1334, 1165, 1077, 1030, 914, 752 cm^{-1}. Mp：83.3 – 84.2 ^{o}C.

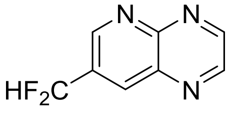

7-(Difluoromethyl)pyrido[2,3-b]pyrazine 4za. The general procedure **A** conducted with 7-bromopyrido[2,3-b]pyrazine (105 mg, 0.500 mmol), [(SIPr)Ag(CF_2H)] (357 mg, 0.650 mmol), Pd(dba)$_2$ (14 mg, 5.0 mol%), DPEPhos (28.0 mg, 10.0 mol%) in dry toluene (2.0 mL) gave 62 mg (69%) of 7-(difluoromethyl)pyrido[2,3-b]pyrazine as a yellow solid. ^{1}H NMR (400 MHz, $CDCl_3$) δ 9.33 (s, 1 H), 9.17 (s, 1 H), 9.04 (s, 1 H), 8.64 (s, 1 H), 6.99 (t, *J* = 52.0 Hz, 1 H); ^{19}F NMR (376 MHz, $CDCl_3$) δ -112.663

(d, J = 56.3 Hz, 2 F); ^{13}C NMR (125 MHz, $CDCl_3$) δ 152.20, 151.06 (t, J = 5.0 Hz), 149.02, 147.10, 137.29, 136.61 (t, J = 6.3 Hz), 120.11, 112.54 (t, J = 237.5 Hz) ppm. MS (EI): 181 (100). HRMS (EI) for $C_8H_5N_3F_2$ Calcd: 181.0452; Found: 181.0450. IR (KBr): ν = 2996, 1627, 1568, 1484, 1336, 1210, 1177, 1030, 985, 976 cm^{-1}. Mp：128 – 129 ^{o}C.

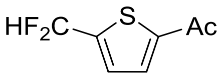

1-[5-(Difluoromethyl)-2-thienyl]ethanone 4ab. The general procedure **C** conducted with 1-[5-bromo-2-thienyl]ethanone (102 mg, 0.500 mmol), [(SIPr)Ag(CF_2H)] (550 mg, 1.00 mmol), Pd(dba)$_2$ (28.0 mg, 10.0 mol%), DPEPhos (56.0 mg, 20.0 mol%) in dry toluene (2.0 mL) gave 62 mg (70%) of 1-[5-(difluoromethyl)-2-thienyl]ethanone as a yellow solid. ^{1}H NMR (500 MHz, $CDCl_3$) δ 7.62 (s, 1 H), 7.30 (s, 1 H), 6.82 (t, J = 55.8 Hz, 1 H), 2.58 (s, 3 H); ^{19}F NMR (376 MHz, $CDCl_3$) δ -104.84 (d, J = 55.7 Hz, 2 F); ^{13}C NMR (126 MHz, $cdcl_3$) δ 190.44, 147.72, 143.39 (t, J = 26.2 Hz), 131.58, 127.80 (t, J = 6.3 Hz), 110.88 (t, J = 239.2 Hz), 26.81 ppm. MS (EI): 161 (100), 176 (42.71). HRMS (EI) for $C_7H_6OF_2S$ Calcd: 176.0107; Found: 176.0112. IR (KBr): ν = 3064, 2991, 1911, 1601, 1451, 1376, 1207, 1106, 1012, 809, 750 cm^{-1}. Mp： 58.5 – 59.2 ^{o}C.

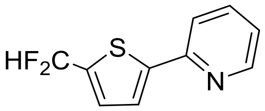

2-[5-(Difluoromethyl)-2-thienyl]pyridine 4ac. The general procedure **C** conducted with 2-[5-bromo-2-thienyl]pyridine (120 mg, 0.500 mmol), [(SIPr)Ag(CF_2H)] (550 mg, 1.00 mmol), Pd(dba)$_2$ (28.0 mg, 10.0 mol%), DPEPhos (56.0 mg, 20.0 mol%) in dry toluene (2.0 mL) gave 83 mg (79%) of 2-[5-(difluoromethyl)-2-thienyl]pyridine as a yellow solid. ^{1}H NMR (400 MHz, $CDCl_3$) δ 8.95 (d, J = 4.0 Hz, 1 H), 7.6-7.8 (m, 2 H), 7.5 (m, 1 H), 7.28 (m, 1 H), 7.21 (t, J = 4.0 Hz, 1 H), 6.84 (t, J = 56.0 Hz, 1 H); ^{19}F NMR (376 MHz, $CDCl_3$) δ -103.11 (d, J = 56.4 Hz, 2 F); ^{13}C NMR (125 MHz, $CDCl_3$) δ 151.63, 149.62, 147.69, 137.55, 136.73, 128.17 (t, J = 6.3 Hz), 123.66, 122.61, 118.87, 111.51 (t, J = 228.8 Hz) ppm; MS (EI): 211 (100). HRMS (EI) for

$C_{10}H_7NF_2S$ Calcd: 211.0269; Found: 211.0268. IR (KBr): ν = 3077, 1651, 1585, 1564, 1455, 1434, 1383, 1222, 1057, 971, 774, 764 cm^{-1}. Mp：65.1 – 66.7 °C.

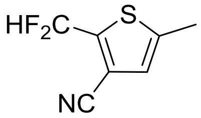

2-(Difluoromethyl)-5-methyl-thiophene-3-carbonitrile 4ad. The general procedure **C** conducted with 2-bromo-5-methyl-thiophene-3-carbonitrile (101 mg, 0.500 mmol), [(SIPr)Ag(CF_2H)] (550 mg, 1.00 mmol), $Pd(dba)_2$ (28.0 mg, 10.0 mol%), DPEPhos (56.0 mg, 20.0 mol%) in dry toluene (2.0 mL) gave 53 mg (62%) of 2-(difluoromethyl)-5- methyl-thiophene-3-carbonitrile as a yellow oil. ^1H NMR (400 MHz, $CDCl_3$) δ 6.94 (t, *J* = 56.0 Hz, 1 H), 6.94 (s, 1 H), 2.54 (s, 3 H); ^{19}F NMR (376 MHz, $CDCl_3$) δ -103.57 (d, *J* = 56.4 Hz, 2 F); ^{13}C NMR (125 MHz, $CDCl_3$) δ 144.41, 142.46, 126.48, 112.87, 110.48 (t, *J* = 6.3 Hz), 109.44 (t, *J* = 238.8 Hz), 15.01 ppm; MS (EI): 173 (100). HRMS (EI) for $C_7H_5NF_2S$ Calcd: 173.0111; Found: 173.0109. IR (KBr): ν = 3002, 2233, 1667, 1487, 1439, 1391, 1230, 1198, 1135, 877, 666 cm^{-1}.

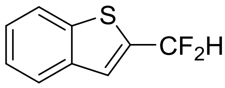

2-(Difluoromethyl)benzothiophene 4ae. The general procedure **C** conducted with 2-bromobenzothiophene (106 mg, 0.500 mmol), [(SIPr)Ag(CF_2H)] (550 mg, 1.00 mmol), $Pd(dba)_2$ (28.0 mg, 10.0 mol%), DPEPhos (56.0 mg, 20.0 mol%) in dry toluene (2.0 mL) gave 72.6 mg (79%) of 2-(difluoromethyl)benzothiophene as a yellow solid. ^1H NMR (400 MHz, $CDCl_3$) δ 8.04 – 7.86 (m, 1 H), 7.86 – 7.73 (m, 1 H), 7.53 (s, 1 H), 7.46 – 7.37 (m, 2 H), 6.94 (t, *J* = 55.8 Hz, 1 H); ^{19}F NMR (376 MHz, $CDCl_3$) δ -103.99 (d, *J* = 55.8 Hz, 2 F); ^{13}C NMR (126 MHz, $CDCl_3$) δ 140.12, 138.27, 136.37 (t, *J* = 25.7 Hz), 125.89, 124.87, 124.66, 124.52 (t, *J* = 7.4 Hz), 122.71, 111.86 (t, *J* = 237.7 Hz) ppm. MS (EI): 184 (100). HRMS (EI) for $C_9H_6F_2S$ Calcd: 184.0154; Found: 184.0155. IR (KBr): ν = 3057, 1570, 1539, 1372, 1223, 1057, 1021, 944, 815, 727 cm^{-1}. Mp：46.4 – 47.2 °C.

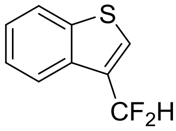

3-(Difluoromethyl)benzothiophene 4af. The general procedure **C** conducted with 3-bromobenzothiophene (106 mg, 0.500 mmol), [(SIPr)Ag(CF_2H)] (550 mg, 1.00 mmol), Pd(dba)$_2$ (28.0 mg, 10.0 mol%), DPEPhos (56.0 mg, 20.0 mol%) in dry toluene (2.0 mL) gave 79 mg (86%) of 3-(difluoromethyl)benzothiophene as a yellow oil. ^{1}H NMR (400 MHz, $CDCl_3$) δ 7.95 (d, J = 7.6 Hz, 1 H), 7.88 (d, J = 8.5 Hz, 1 H), 7.71 (s, 1 H), 7.57 – 7.31 (m, 2 H), 6.91 (t, J = 55.5 Hz, 1 H); ^{19}F NMR (376 MHz, $CDCl_3$) δ -111.47 (d, J = 55.4 Hz, 2 F); ^{13}C NMR (126 MHz, $CDCl_3$) δ 140.61, 135.47 (t, J = 2.0 Hz), 130.00 (t, J = 24.2 Hz), 127.57 (t, J = 8.8 Hz), 125.22, 124.90, 122.85, 122.50, 112.26 (t, J = 236.8 Hz) ppm. MS (EI): 184 (100). HRMS (EI) for $C_9H_6F_2S$ Calcd: 184.0158; Found: 184.0163. IR (KBr): ν = 3111, 2966, 2852, 1567, 1430, 1363, 1161, 1116, 1019, 771, 701 cm^{-1}.

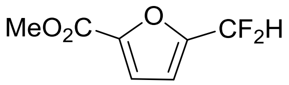

Methyl 5-(difluoromethyl)furan-2-carboxylate 4ag. The general procedure **C** conducted with methyl 5-bromo-furan-2-carboxylate (102 mg, 0.500 mmol), [(SIPr)Ag(CF_2H)] (550 mg, 1.00 mmol), Pd(dba)$_2$ (28.0 mg, 10.0 mol%), DPEPhos (56.0 mg, 20.0 mol%) in dry toluene (2.0 mL) gave 52 mg (59%) of methyl 5-(difluoromethyl)furan-2-carboxylate as a yellow oil. ^{1}H NMR (400 MHz, $CDCl_3$) δ 7.16 (d, J = 4 Hz, 1 H), 6.73 (m, 1 H), 6.63 (t, J = 52 Hz, 1 H), 3.15 (s, 3 H); ^{19}F NMR (376 MHz, $CDCl_3$) δ -116.34 (d, J = 52.6 Hz, 2 F); ^{13}C NMR (125 MHz, $CDCl_3$) δ 158.49, 149.81 (t, J = 31.3 Hz), 145.65, 117.97 (t, J = 3.76 Hz), 111.39 (t, J = 7.52 Hz), 107.91 (t, J = 233.8 Hz), 52.14 ppm. MS (EI): 57 (100), 176 (18.96). HRMS (EI) for $C_7H_6O_3F_2$ Calcd: 176.0285; Found: 176.0283. IR (KBr): ν = 3138, 2957, 2871, 1735, 1601, 1399, 1255, 1211, 1083, 1018, 764, 647 cm^{-1}.

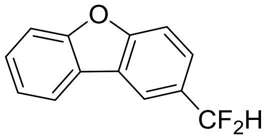

2-(Difluoromethyl)dibenzofuran 4ah. The general procedure **C** conducted with 2-bromo-dibenzofuran (124 mg, 0.500 mmol), [(SIPr)Ag(CF_2H)] (550 mg, 1.00 mmol), Pd(dba)$_2$ (28.0 mg, 10.0 mol%), DPEPhos (56.0 mg, 20.0 mol%) in dry

toluene (2.0 mL) gave 86 mg (79%) of 2-(difluoromethyl)dibenzofuran as a yellow solid. ^{1}H NMR (500 MHz, $CDCl_3$) δ 8.13 (s, 1 H), 7.98 (d, *J* = 7.6 Hz, 1 H), 7.64 (d, *J* = 8.0 Hz, 1 H), 7.61 (d, *J* = 4.0 Hz, 2 H), 7.51 (t, *J* = 7.7 Hz, 1 H), 7.39 (t, *J* = 7.5 Hz, 1 H), 6.82 (t, *J* = 56.7 Hz, 1 H); ^{19}F NMR (376 MHz, $CDCl_3$) δ -107.84 (d, *J* = 56.6 Hz, 2 F); ^{13}C NMR (126 MHz, $CDCl_3$) δ 156.71, 127.88, 124.63 (t, *J* = 5.9 Hz), 124.56, 123.57, 123.19, 123.16, 121.08, 120.85, 118.36 (t, *J* = 6.3 Hz), 114.99 (t, *J* = 238.6 Hz), 111.98, 111.90 ppm. MS (EI): 218 (100). HRMS (EI) for $C_{13}H_8OF_2$ Calcd: 218.0543; Found: 218.0545. IR (KBr): ν = 3310, 3111, 1884, 1818, 1664, 1544, 1346, 1271, 1062, 1026, 821 cm^{-1}. Mp：86.1 – 87.3 ^{o}C.

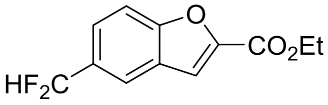

Ethyl 5-(difluoromethyl)benzofuran-2-carboxylate 4ai. The general procedure **C** conducted with ethyl 5-bromo-benzofuran-2-carboxylate (135 mg, 0.500 mmol), [(SIPr)Ag(CF_2H)] (550 mg, 1.00 mmol), Pd(dba)$_2$ (28.0 mg, 10.0 mol%), DPEPhos (56.0 mg, 20.0 mol%) in dry toluene (2.0 mL) gave 105 mg (88%) of ethyl 5-(difluoromethyl)benzofuran-2-carboxylate as a yellow solid. ^{1}H NMR (400 MHz, $CDCl_3$) δ 7.83 (s, 1 H), 7.64 (d, *J* = 8.7 Hz, 1 H), 7.57 (d, *J* = 9.0 Hz, 1 H), 7.54 (s, 1 H), 6.74 (t, *J* = 56.5 Hz, 1 H), 4.44 (q, *J* = 7.1 Hz, 2 H), 1.42 (t, *J* = 7.1 Hz, 3 H); ^{19}F NMR (376 MHz, $cdcl_3$) δ -108.55 (d, *J* = 56.5 Hz, 2 F); ^{13}C NMR (126 MHz, $CDCl_3$) δ 159.17, 156.43, 146.94, 130.36 (t, *J* = 22.5 Hz), 126.98, 124.84 (t, *J* = 5.5 Hz), 120.64 (t, *J* = 6.7 Hz), 114.64 (t, *J* = 238.9 Hz), 113.57, 112.85, 61.71, 14.20 ppm. MS (EI): 195 (100), 240 (78.47). HRMS (EI) for $C_{12}H_{10}F_2O_3$ Calcd: 240.0598; Found: 240.0600. IR (KBr): ν = 3076, 1890, 1828, 1725, 1621, 1374, 1342, 1321, 1150, 1120, 1014, 874 cm^{-1}. Mp：57.3 – 58.2 ^{o}C.

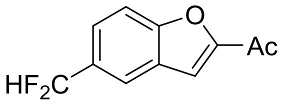

1-[5-(Difluoromethyl)benzofuran-2-yl]ethanone 4aj. The general procedure **C** conducted with 1-[5-bromo-benzofuran-2-yl]ethanone (120 mg, 0.5 mmol), [(SIPr)Ag(CF_2H)] (550 mg, 1.00 mmol), Pd(dba)$_2$ (28.0 mg, 10.0 mol%), DPEPhos (56.0 mg, 20.0 mol%) in dry toluene (2.0 mL) gave 78 mg (75%) of

1-[5-(difluoromethyl)benzofuran-2-yl]ethanone as a yellow solid. ^{1}H NMR (400 MHz, $CDCl_3$) δ 7.78 (s, 1 H), 7.60-7.66 (m, 2 H), 7.52 (s, 1 H), 6.75 (t, *J* = 56.0 Hz, 1 H), 2.62 (s, 3 H); ^{19}F NMR (376 MHz, $CDCl_3$) δ -108.56 (d, *J* = 56.4 Hz, 2 F); ^{13}C NMR (125 MHz, $CDCl_3$) δ 188.45, 156.40, 153.74, 130.52 (t, *J* = 23.8 Hz), 127.08, 125.48 (t, *J* = 5.0 Hz), 121.15 (t, *J* = 6.3 Hz), 114.57 (t, *J* = 237.5 Hz), 113.01, 112.68, 26.54 ppm. MS (EI): 210 (100). HRMS (EI) for $C_{11}H_8O_2F_2$ Calcd: 210.0492; Found: 210.0490. IR (KBr): ν = 3342, 3106, 2974, 1682, 1619, 1443, 1341, 1373, 1151, 1020, 900, 825 cm^{-1}. Mp：70.4 – 71.2 ^{o}C.

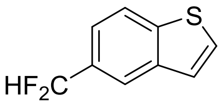

5-(Difluoromethyl)benzothiophene 4ak. The general procedure **C** conducted with 5-bromobenzothiophene (106 mg, 0.500 mmol), [(SIPr)Ag(CF_2H)] (550 mg, 1.00 mmol), Pd(dba)$_2$ (28.0 mg, 10.0 mol%), DPEPhos (56.0 mg, 20.0 mol%) in dry toluene (2.0 mL) gave 62 mg (68%) of 5-(difluoromethyl)benzothiophene as a yellow solid. ^{1}H NMR (400 MHz, $CDCl_3$) δ 7.97 (s, 1 H), 7.95 (s, 1 H), 7.54 (d, *J* = 5.5 Hz, 1 H), 7.49 (d, *J* = 8.6 Hz, 1 H), 7.40 (d, *J* = 5.5 Hz, 1 H), 6.79 (t, *J* = 56.6 Hz, 1 H); ^{19}F NMR (376 MHz, $CDCl_3$) δ -108.71 (d, *J* = 56.6 Hz, 2 F); ^{13}C NMR (126 MHz, $CDCl_3$) δ 141.88, 139.32, 130.69 (t, *J* = 22.3 Hz), 127.91, 123.99, 122.98, 121.08 (t, *J* = 6.9 Hz), 121.06 (t, *J* = 5.4 Hz), 115.13 (t, *J* = 239.5 Hz) ppm; MS (EI): 184 (100). HRMS (EI) for $C_9H_6F_2S$ Calcd: 184.0518; Found: 184.0519. IR (KBr): ν = 3853, 3649, 1230, 1116, 895, 777, 700, 612 cm^{-1}. Mp：32.1 – 33.6 ^{o}C.

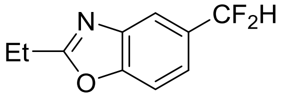

5-(Difluoromethyl)-2-ethyl-1,3-benzoxazole 4al. The general procedure **C** conducted with 5-bromo-2-ethyl-1,3-benzoxazole (113 mg, 0.500 mmol), [(SIPr)Ag(CF_2H)] (550 mg, 1.00 mmol), Pd(dba)$_2$ (28.0 mg, 10.0 mol%), DPEPhos (56.0 mg, 20.0 mol%) in dry toluene (2.0 mL) gave 70 mg (71%) of 5-(difluoromethyl)-2-ethyl-1,3-benzoxazole as a yellow oil. ^{1}H NMR (400 MHz, $CDCl_3$) δ 7.80 (s, 1 H), 7.54 (d, *J* = 8.0 Hz, 1 H), 7.46 (d, *J* = 8.0 Hz, 1 H), 6.73 (t, *J* =

56.0 Hz, 1 H), 3.00 (q, J = 8.0 Hz, 2 H), 1.45 (t, J = 8.0 Hz, 3 H); ^{19}F NMR (376 MHz, $CDCl_3$) δ -107.98 (d, J = 56.4 Hz, 2 F); ^{13}C NMR (125 MHz, $CDCl_3$) δ 169.48, 152.05(t, J = 2.53 Hz), 141.55, 130.70 (t, J = 29.0 Hz), 122.00 (t, J = 7.56 Hz), 117.29 (t, J = 8.8 Hz), 114.68 (t, J = 240.4 Hz), 110.60, 22.15, 10.75 ppm. MS (EI): 196 (100), 197 (56.13). HRMS (EI) for $C_{10}H_9NOF_2$ Calcd: 197.0652; Found: 197.0647. IR (KBr): ν = 2984, 2883, 1626, 1576, 1446, 1396, 1172, 1149, 1027, 917, 696 cm^{-1}.

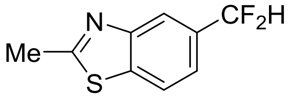

5-(Difluoromethyl)-2-methyl-1,3-benzothiazole 4am. The general procedure **C** conducted with 5-bromo-2-methyl-1,3-benzothiazole (114 mg, 0.500 mmol), [(SIPr)Ag(CF_2H)] (550 mg, 1.00 mmol), Pd(dba)$_2$ (28.0 mg, 10.0 mol%), DPEPhos (56.0 mg, 20.0 mol%) in dry toluene (2.0 mL) gave 67 mg (68%) of 5-(difluoromethyl)-2-methyl-1,3-benzothiazole as a yellow oil. ^{1}H NMR (400 MHz, $CDCl_3$) δ 8.05 (s, 1 H), 7.87 (d, J = 8.3 Hz, 1 H), 7.47 (d, J = 8.3 Hz, 1 H), 6.76 (t, J = 56.4 Hz, 1 H), 2.83 (s, 3 H); ^{19}F NMR (376 MHz, $CDCl_3$) δ -109.21 (d, J = 56.4 Hz, 2 F); ^{13}C NMR (126 MHz, $CDCl_3$) δ 168.55, 153.10, 138.07, 132.49 (t, J = 22.6 Hz), 121.90, 121.54 (t, J = 5.5 Hz), 119.84 (t, J = 6.8 Hz), 114.65 (t, J = 239.1 Hz), 20.18 ppm. MS (EI): 199 (100). HRMS (EI) for $C_9H_7NF_2S$ Calcd: 199.0267; Found: 199.0273. IR (KBr): ν = 2962, 2869, 1614, 1525, 1457, 1357, 1291, 1250, 1027, 931, 719 cm^{-1}.

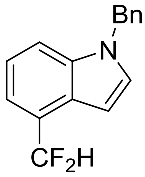

1-Benzyl-4-(difluoromethyl)indole 4an. The general procedure **C** conducted with 5-bromobenzothiophene (143 mg, 0.500 mmol), [(SIPr)Ag(CF_2H)] (550 mg, 1.00 mmol), Pd(dba)$_2$ (28.0 mg, 10.0 mol%), DPEPhos (56.0 mg, 20.0 mol%) in dry toluene (2.0 mL) gave 105 mg (82%) of 1-benzyl-4-(difluoromethyl)indole as a yellow solid. ^{1}H NMR (400 MHz, $CDCl_3$) δ 7.40 (d, J = 8.0 Hz, 1 H), 7.15-7.35 (m, 6 H), 7.09 (s, 2 H), 6.95 (t, J = 56.0 Hz, 1 H), 6.76 (s, 1 H), 5.34 (s, 2 H); ^{19}F NMR (376

MHz, $CDCl_3$) δ -110.37 (d, *J* = 56.4 Hz, 2 F); ^{13}C NMR (125 MHz, $CDCl_3$) δ 137.05, 129.56, 128.81, 127.80, 126.71, 125.91 (t, *J* = 22.5 Hz), 121.14, 117.58, 117.52, 115.68 (t, *J* = 225.0 Hz), 113.81, 112.41 (t, *J* = 2.5 Hz), 100.19, 50.31 ppm. MS (EI): 91 (100); 257 (79.1). HRMS (EI) for $C_{16}H_{13}NF_2$ Calcd: 257.1016; Found: 257.1012. IR (KBr): ν = 3098, 1951, 1716, 1606, 1556, 1453, 1337, 1312, 1146, 1078, 945, 826, 740 cm^{-1}. Mp：56.1 – 57.5 ^oC.

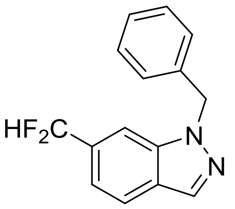

1-Benzyl-6-(difluoromethyl)indazole 4ao. The general procedure **C** conducted with 1-benzyl-6-bromo-indazole (144 mg, 0.500 mmol), [(SIPr)Ag(CF_2H)] (550 mg, 1.00 mmol), $Pd(dba)_2$ (28.0 mg, 10.0 mol%), DPEPhos (56.0 mg, 20.0 mol%) in dry toluene (2.0 mL) gave 112 mg (87%) of 1-benzyl-6-(difluoromethyl)indazole as a yellow solid. ^1H NMR (400 MHz, $CDCl_3$) δ 7.95 (s, 1 H), 7.67 (d, *J* = 8.0 Hz, 1 H), 7.38 (s, 1 H), 7.05-7.14 (m, 5 H), 7.06 (d, *J* = 8.0 Hz, 1 H), 6.59 (t, *J* = 56.0 Hz, 1 H), 5.48 (s, 2 H); ^{19}F NMR (376 MHz, $CDCl_3$) δ -109.36 (d, *J* = 56.4 Hz, 2 F); ^{13}C NMR (125 MHz, $CDCl_3$) δ 138.91, 136.39, 133.48, 132.57 (t, *J* = 27.5 Hz), 128.82, 127.94, 127.13, 125.58, 121.94, 117.76 (t, *J* = 6.25 Hz), 114.88 (t, *J* = 297.5 Hz), 106.98 (t, *J* = 8.75 Hz), 53.09 ppm. MS (EI): 91 (100), 258 (94.48). HRMS (EI) for $C_{15}H_{12}N_2F_2$ Calcd: 258.0969; Found: 258.0970. IR (KBr): ν 3107, 2988, 1538, 1512, 1455, 1398, 1328, 1188, 1074, 1017, 878, 721 cm^{-1}. Mp：78– 79.5 ^oC.

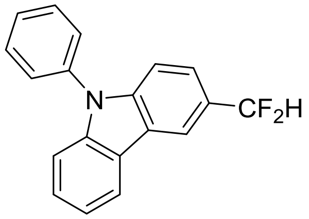

3-(Difluoromethyl)-9-phenyl-carbazole 4ap. The general procedure **C** conducted with 3-bromo-9-phenyl-carbazole (160 mg, 0.500 mmol), [(SIPr)Ag(CF_2H)] (550 mg, 1.00 mmol), $Pd(dba)_2$ (28.0 mg, 10.0 mol%), DPEPhos (56.0 mg, 20.0 mol%) in dry

toluene (2.0 mL) gave 108 mg (74%) of 3-(difluoromethyl)-9-phenyl-carbazole as a yellow oil. ^{1}H NMR (400 MHz, $CDCl_3$) δ 8.33 (s, 1 H), 8.20 (d, *J* = 7.8 Hz, 1 H), 7.64 (t, *J* = 7.7 Hz, 2 H), 7.60 – 7.43 (m, 7 H), 7.35 (t, *J* = 7.3 Hz, 1 H), 6.88 (t, *J* = 56.9 Hz, 1 H); ^{19}F NMR (376 MHz, $CDCl_3$) δ -107.39 (d, *J* = 56.8 Hz, 2 F); ^{13}C NMR (126 MHz, $CDCl_3$) δ 142.03, 141.48, 137.18, 130.01, 127.89, 127.13, 126.60, 126.13 (t, *J* = 22.5 Hz), 123.24 (t, *J* = 5.6 Hz), 122.97, 123.00, 120.48, 120.47, 118.13 (t, *J* = 6.5 Hz), 115.77 (t, *J* = 237.5 Hz), 110.09, 109.98 ppm. MS (EI): 293 (100). HRMS (EI) for $C_{19}H_{13}NF_2$ Calcd: 293.1016; Found: 293.1013. IR (KBr): ν = 3062, 2925, 2851, 1631, 1598, 1503, 1455, 1364, 1340, 1277, 1235, 907, 747 cm^{-1}.

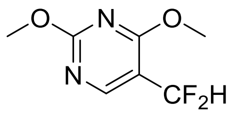

5-(Difluoromethyl)-2,4-dimethoxy-pyrimidine 5a. The general procedure **A** conducted with 5-iodo-2,4-dimethoxy-pyrimidine (133 mg, 0.500 mmol), [(SIPr)Ag(CF_2H)] (357 mg, 0.650 mmol), Pd(dba)$_2$ (14 mg, 5.0 mol%), DPEPhos (28.0 mg, 10.0 mol%) in dry toluene (2.0 mL) gave 70.3 mg (74%) of 5-(difluoromethyl)-2,4-dimethoxy-pyrimidine as a yellow solid. ^{1}H NMR (400 MHz, $CDCl_3$) δ 8.43 (s, 1 H), 6.74 (t, *J* = 56.0 Hz, 1 H), 4.03 (s, 3 H), 4.01 (s, 3 H); ^{19}F NMR (376 MHz, $CDCl_3$) δ -116.02 (d, *J* = 56.4 Hz, 2 F); ^{13}C NMR (125 MHz, $CDCl_3$) δ 168.59, 166.44, 156.72 (t, *J* = 6.25 Hz), 110.93 (t, *J* = 293.8 Hz), 109.04 (t, *J* = 30 Hz), 55.19, 54.30 ppm. MS (EI): 190 (100). HRMS (EI) for $C_7H_8N_2O_2F_2$ Calcd: 190.0554; Found: 190.0551. IR (KBr): ν = 3029, 2973, 2360, 1473, 1457, 1421, 1370, 1295, 1250, 1078, 1022, 952, 549 cm^{-1}. Mp：92.1 – 93.2 ^{o}C.

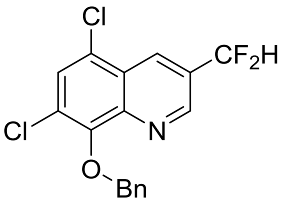

8-Benzyloxy-5,7-dichloro-3-(difluoromethyl)quinoline 5b. The general procedure **A** conducted with 8-benzyloxy-5,7-dichloro-3-iodoquinoline (215 mg, 0.500 mmol), [(SIPr)Ag(CF_2H)] (357 mg, 0.650 mmol), Pd(dba)$_2$ (14 mg, 5.0 mol%), DPEPhos

(28.0 mg, 10.0 mol%) in dry toluene (2.0 mL) gave 138 mg (78%) of 8-benzyloxy-5,7-dichloro-3-(difluoromethyl)quinoline as a yellow solid. ^{1}H NMR (400 MHz, $CDCl_3$) δ 9.13 (s, 1 H), 8.64 (s, 1 H), 7.71 (s, 1 H), 7.59 (d, *J* = 7.1 Hz, 2 H), 7.46 – 7.29 (m, 3 H), 6.93 (t, *J* = 55.6 Hz, 2 H), 5.48 (s, 2 H); ^{19}F NMR (376 MHz, $CDCl_3$) δ -112.19 (d, *J* = 55.6 Hz, 2 F); ^{13}C NMR (126 MHz, $CDCl_3$) δ 150.34, 147.50 (t, *J* = 5.3 Hz), 144.60, 136.68, 131.48 (t, *J* = 6.9 Hz), 129.00, 128.78, 128.63, 128.38, 128.33, 128.08, 126.64, 125.11, 113.30 (t, *J* = 242.0 Hz) ppm. MS (EI): 91 (100), 353 (4.33). HRMS (EI) for $C_{17}H_{11}NOF_2Cl_2$ Calcd: 353.0186; Found: 353.0180. IR (KBr): ν = 3083, 3065, 2953, 1744, 1617, 1581, 1499, 1480, 1455, 1355, 1080, 829 cm^{-1}. Mp：74.1 – 75.2 ^{o}C.

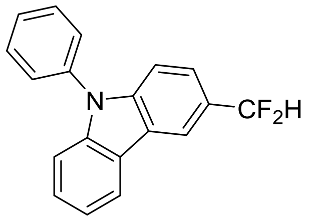

3-(Difluoromethyl)-9-phenyl-carbazole 5c. The general procedure **A** conducted with 3-iodo-9-phenyl-carbazole (184 mg, 0.500 mmol), [(SIPr)Ag(CF_2H)] (357 mg, 0.650 mmol), Pd(dba)$_2$ (14 mg, 5.0 mol%), DPEPhos (28.0 mg, 10.0 mol%) in dry toluene (2.0 mL) gave 79.1 mg (54%) of 3-(difluoromethyl)-9-phenyl-carbazole as a yellow oil. ^{1}H NMR (400 MHz, $CDCl_3$) δ 8.33 (s, 1 H), 8.20 (d, *J* = 7.8 Hz, 1 H), 7.64 (t, *J* = 7.7 Hz, 2 H), 7.60 – 7.43 (m, 7 H), 7.35 (t, *J* = 7.3 Hz, 1 H), 6.88 (t, *J* = 56.9 Hz, 1 H); ^{19}F NMR (376 MHz, $CDCl_3$) δ -107.39 (d, *J* = 56.8 Hz, 2 F); ^{13}C NMR (126 MHz, $CDCl_3$) δ 142.09, 141.51, 137.21, 130.03, 127.92, 127.15, 126.64, 126.16 (t, *J* = 22.5 Hz), 123.27 (t, *J* = 5.6 Hz), 123.20, 123.00, 120.52, 120.50, 118.16 (t, *J* = 6.5 Hz), 115.80 (t, *J* = 237.5 Hz), 110.12, 110.01 ppm. MS (EI): 293 (100). HRMS (EI) for $C_{19}H_{13}NF_2$ Calcd: 293.1016; Found: 293.1013. IR (KBr): ν = 3062, 2925, 2851, 1631, 1598, 1503, 1455, 1364, 1340, 1277, 1235, 907, 747 cm^{-1}.

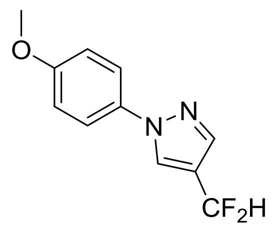

1-(4-Methoxyphenyl)-4-(difluoro)-pyrazole 5d. The general procedure **B** conducted with 1-(4-methoxyphenyl)-4-iodo-pyrazole (150 mg, 0.500 mmol), [(SIPr)Ag(CF_2H)] (357 mg, 0.650 mmol), Pd(dba)$_2$ (14 mg, 5.0 mol%), DPEPhos (28.0 mg, 10.0 mol%) in dry toluene (2.0 mL) gave 68 mg (60%) of 1-(4-methoxyphenyl)-4 -(difluoromethyl)-pyrazole as a white solid. ^{1}H NMR (400 MHz, $CDCl_3$) δ 7.97 (s, 1 H), 7.79 (s, 1 H), 7.56 (d, *J* = 9.0 Hz, 2 H), 6.97 (d, *J* = 9.0 Hz, 2 H), 6.76 (t, *J* = 56.7 Hz, 1 H), 3.83 (s, 3 H).; ^{19}F NMR (376 MHz, $CDCl_3$) δ -107.75 (d, *J* = 58.5 Hz, 2 F);^{13}C NMR (101 MHz, $CDCl_3$) δ 158.87, 138.10 (t, *J* = 4.6 Hz), 133.30, 125.77 (t, *J* = 5.7 Hz), 121.28, 118.59 (t, *J* = 26.9 Hz), 114.64, 110.89 (t, *J* = 234.3 Hz), 55.60. ppm. MS (EI): 224 (100). HRMS (EI) for $C_{11}H_{10}N_2F_2O$ Calcd: 224.0759; Found: 224.0761. IR (KBr): ν = 2964, 2869, 1724, 1596, 1519m 1439, 1344, 1303, 1214, 1172, 1084, 957, 833 cm^{-1}.

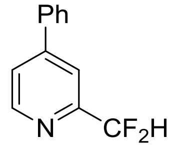

2-(Difluoromethyl)-4-phenyl-pyridine 5e. The general procedure **A** conducted with 2-chloro-4-phenyl-pyridine (94 mg, 0.50 mmol), [(SIPr)Ag(CF_2H)] (357 mg, 0.650 mmol), Pd(dba)$_2$ (14 mg, 5.0 mol%), DPEPhos (28.0 mg, 10.0 mol%) in dry toluene (2.0 mL) gave 73.8 mg (72%) of 2-(difluoromethyl)-4-phenyl-pyridine as a yellow oil. ^{1}H NMR (400 MHz, $CDCl_3$) δ 8.68 (d, *J* = 5.1 Hz, 1 H), 7.84 (s, 1 H), 7.65 (d, *J* = 7.0 Hz, 2 H), 7.60 (d, *J* = 5.0 Hz, 1 H), 7.54 – 7.43 (m, 3 H), 6.69 (t, *J* = 55.5 Hz, 1 H); ^{19}F NMR (376 MHz, $CDCl_3$) δ -115.83 (d, *J* = 55.5 Hz, 2 F); ^{13}C NMR (126 MHz, $CDCl_3$) δ 153.40 (t, *J* = 25.4 Hz), 149.98, 149.93, 137.35, 129.55, 129.26, 127.06, 123.26, 118.04 (t, *J* = 3.1 Hz), 113.96 (t, *J* = 242.0 Hz) ppm. MS (EI): 205 (100). HRMS (EI) for $C_{12}H_9F_2N$ Calcd: 205.0703; Found: 205.0701. IR (KBr): ν = 3063, 2925, 1605, 1551, 1374, 1207, 1078, 1002, 909, 852, 762 cm^{-1}.

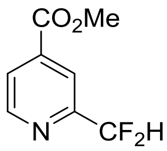

Methyl 2-(difluoromethyl)pyridine-4-carboxylate 5f. The general procedure **A** conducted with methyl 2-(difluoromethyl)pyridine-4-carboxylate (86 mg, 0.50 mmol), [(SIPr)Ag(CF_2H)] (357 mg, 0.650 mmol), Pd(dba)$_2$ (14 mg, 5.0 mol%), DPEPhos (28.0 mg, 10.0 mol%) in dry toluene (2.0 mL) gave 58 mg (62%) of methyl 2-(difluoromethyl)pyridine-4-carboxylate as a yellow oil. ^{1}H NMR (400 MHz, $CDCl_3$) δ 8.81 (d, *J* = 4.0 Hz, 1 H), 8.18 (s, 1 H), 7.97 (d, *J* = 4.0 Hz, 1 H), 6.69 (t, *J* = 56.0 Hz, 1 H), 3.98 (s, 3 H); ^{19}F NMR (376 MHz, $CDCl_3$) δ -116.09 (d, *J* = 56.4 Hz, 2 F); ^{13}C NMR (125 MHz, $CDCl_3$) δ 164.65, 153.90, 150.38, 138.86, 124.67, 119.60 (t, *J* = 3.8 Hz), 113.43 (t, *J* = 243.8 Hz), 53.02 ppm. MS (EI): 187 (100). HRMS (EI) for $C_8H_7NO_2F_2$ Calcd: 187.0445; Found: 187.0450. IR (KBr): ν = 3094, 2963, 1735, 1591, 1431, 1370, 1144, 989, 851 cm^{-1}.

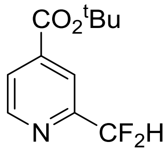

***tert*-Butyl 2-(difluoromethyl)pyridine-4-carboxylate 5g.** The general procedure **A** conducted with methyl tert-butyl 2-(difluoromethyl)pyridine-4-carboxylate (106 mg, 0.500 mmol), [(SIPr)Ag(CF_2H)] (357 mg, 0.650 mmol), Pd(dba)$_2$ (14 mg, 5.0 mol%), DPEPhos (28.0 mg, 10.0 mol%) in dry toluene (2.0 mL) gave 82.4 mg (72%) of tert-butyl 2-(difluoromethyl)pyridine-4-carboxylate as a yellow oil. ^{1}H NMR (400 MHz, $CDCl_3$) δ 8.77 (d, *J* = 5.0 Hz, 1 H), 8.10 (s, 1 H), 7.91 (d, *J* = 5.0 Hz, 1 H), 6.68 (t, *J* = 55.3 Hz, 1 H), 1.61 (s, 9 H); ^{19}F NMR (376 MHz, $CDCl_3$) δ -115.97 (d, *J* = 55.3 Hz, 2 F); ^{13}C NMR (126 MHz, $CDCl_3$) δ 163.21, 153.67 (t, *J* = 26.0 Hz), 150.19, 140.77, 124.65 (t, *J* = 1.4 Hz), 119.51 (t, *J* = 3.1 Hz), 113.58 (t, *J* = 240.8 Hz), 83.07, 27.99 ppm. MS (EI): 156 (100), 229 (2.23). HRMS (EI) for $C_{11}H_{13}F_2NO_2$ Calcd: 229.0914; Found: 229.0916. IR (KBr): ν = 3432, 2981, 2935, 1726, 1605, 1479, 1371, 1162, 1076, 995, 871, 765 cm^{-1}.

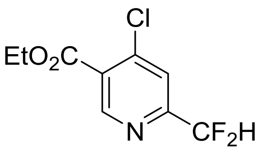

Ethyl 4-chloro-6-(difluoromethyl)pyridine-3-carboxylate 5h. The general procedure **A** conducted with ethyl 4-chloro-6-chloropyridine-3-carboxylate (110 mg, 0.500 mmol), [(SIPr)Ag(CF_2H)] (357 mg, 0.650 mmol), Pd(dba)$_2$ (14 mg, 5.0 mol%), DPEPhos (28.0 mg, 10.0 mol%) in dry toluene (2.0 mL) gave 88.1 mg (75%) of ethyl 4-chloro-6-(difluoromethyl)pyridine-3-carboxylate as a yellow oil. ^{1}H NMR (500 MHz, $CDCl_3$) δ 9.02 (s, 1 H), 7.72 (s, 1 H), 6.63 (t, *J* = 54.9 Hz, 1 H), 4.45 (q, *J* = 7.1 Hz, 2 H), 1.42 (t, *J* = 7.2 Hz, 3 H); ^{19}F NMR (376 MHz, $CDCl_3$) δ -117.19 (d, *J* = 54.9 Hz, 2 F); ^{13}C NMR (126 MHz, $CDCl_3$) δ 163.23, 155.61, 151.97, 145.42, 127.75, 122.49 (t, *J* = 3.3 Hz), 112.64 (t, *J* = 242.0 Hz), 62.35, 14.12 ppm. MS (EI): 190 (100), 235 (17.04). HRMS (EI) for $C_9H_8F_2NO_2Cl$ Calcd: 235.0212; Found: 235.0211. IR (KBr): ν = 2986, 1735, 1589, 1552, 1476, 1370, 1275, 1228, 1096, 928 cm^{-1}.

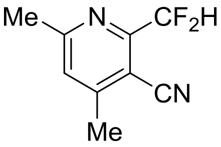

2-(Difluoromethyl)-4,6-dimethyl-pyridine-3-carbonitrile 5i. The general procedure **A** conducted with 2-chloro-4,6-dimethyl-pyridine-3-carbonitrile (83 mg, 0.50 mmol), [(SIPr)Ag(CF_2H)] (357 mg, 0.650 mmol), Pd(dba)$_2$ (14 mg, 5.0 mol%), DPEPhos (28.0 mg, 10.0 mol%) in dry toluene (2.0 mL) gave 74.6 mg (82%) of 2-(difluoromethyl)-4,6-dimethyl-pyridine-3-carbonitrile as a yellow solid. ^{1}H NMR (500 MHz, $CDCl_3$) δ 7.27 (s, 1 H), 6.72 (t, *J* = 54.0 Hz, 1 H), 2.62 (s, 3 H), 2.59 (s, 3 H); ^{19}F NMR (376 MHz, $CDCl_3$) δ -115.29 (d, *J* = 54.0 Hz, 2 F); ^{13}C NMR (126 MHz, $CDCl_3$) δ 161.96, 153.93, 152.94, 126.07, 113.58, 113.21 (t, *J* = 243.0 Hz), 105.30, 24.49, 20.25 ppm. MS (EI): 182 (100). HRMS (EI) for $C_9H_8N_2F_2$ Calcd: 182.0656; Found: 182.0660. IR (KBr): ν = 2960, 2230, 1661, 1601, 1507, 1379, 1354, 1226, 1107, 1040, 969, 779 cm^{-1}. Mp：74 – 76 ^{o}C.

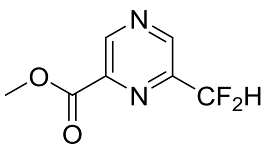

Methyl 6-(difluoromethyl)pyrazine-2-carboxylate 5j. The general procedure **A** conducted with methyl 6-chloro-pyrazine-2-carboxylate (86 mg, 0.50 mmol), [(SIPr)Ag(CF_2H)] (357 mg, 0.650 mmol), Pd(dba)$_2$ (14 mg, 5.0 mol%), DPEPhos (28.0 mg, 10.0 mol%) in dry toluene (2.0 mL) gave 80 mg (85%) of methyl 6-(difluoromethyl)pyrazine-2-carboxylate as a yellow oil. ^{1}H NMR (400 MHz, $CDCl_3$) δ 9.39 (s, 1 H), 9.09 (s, 1 H), 6.78 (t, J = 56.0 Hz, 1 H), 4.04 (s, 3 H); ^{19}F NMR (376 MHz, $CDCl_3$) δ -116.62 (d, J = 56.4 Hz, 2 F); ^{13}C NMR (125 MHz, $CDCl_3$) δ 163.59, 147.71, 147.70(t, J = 34.0 Hz), 145.01 (t, J = 3.75 Hz), 142.22, 112.90 (t, J = 240.0 Hz), 53.42 ppm. MS (EI): 191 (100). HRMS (EI) for $C_7H_6N_2O_2F_2$ Calcd: 188.0397; Found: 188.0403. IR (KBr): ν = 3446, 2958, 2920, 2850, 1734, 1653, 1457, 1313, 907, 813 cm^{-1}.

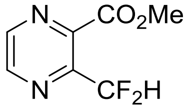

Methyl 3-(difluoromethyl)pyrazine-2-carboxylate 5k. The general procedure **A** conducted with methyl 3-chloro-pyrazine-2-carboxylate (86 mg, 0.50 mmol), [(SIPr)Ag(CF_2H)] (357 mg, 0.650 mmol), Pd(dba)$_2$ (14 mg, 5.0 mol%), DPEPhos (28.0 mg, 10.0 mol%) in dry toluene (2.0 mL) gave 61.1 mg (65%) of methyl 3-(difluoromethyl)pyrazine-2-carboxylate as a yellow oil. ^{1}H NMR (500 MHz, $CDCl_3$) δ 9.38 (s, 1 H), 9.08 (s, 1 H), 6.78 (t, J = 55.0 Hz, 1 H), 4.02 (s, 3 H); ^{19}F NMR (376 MHz, $CDCl_3$) δ -118.95 (d, J = 52.6 Hz, 2 F); ^{13}C NMR (125 MHz, $CDCl_3$) δ 163.91, 147.67 (t, J = 18.7 Hz), 14147.67, 144.98 (t, J = 2.5 Hz), 142.20 (t, J = 2.5 Hz), 112.90 (t, J = 240 Hz), 55.39 ppm. MS (EI): 188 (13.62), 130 (100). HRMS (EI) for $C_7H_6N_2O_2F_2$ Calcd: 188.0395; Found: 188.0397. IR (KBr): ν = 2959, 1733, 1568, 1445, 1426, 1368, 1301, 1207, 1065, 734 cm^{-1}.

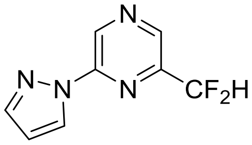

2-(Difluoromethyl)-6-pyrazol-1-yl-pyrazine 5l. The general procedure **A** conducted with 2-chloro-6-pyrazol-1-yl-pyrazine (90 mg, 0.50 mmol), [(SIPr)Ag(CF_2H)] (357 mg, 0.650 mmol), Pd(dba)$_2$ (14 mg, 5.0 mol%), DPEPhos (28.0 mg, 10.0 mol%) in dry toluene (2.0 mL) gave 74.4 mg (76%) of 2-(difluoromethyl)-6-pyrazol-1-yl-pyrazine as a yellow solid. ^{1}H NMR (400 MHz, $CDCl_3$) δ 9.47 (s, 1 H), 8.78 (s, 1 H), 8.52 (s, 1 H), 7.82 (s, 1 H), 6.91 – 6.45 (m, 2 H); ^{19}F NMR (376 MHz, $CDCl_3$) δ -117.72 (d, J = 54.6 Hz, 2 F); ^{13}C NMR (101 MHz, $CDCl_3$) δ 146.54, 145.06 (t, J = 26.5 Hz), 143.68, 138.29 (t, J = 4.1 Hz), 137.61, 127.72, 112.65 (t, J = 241.3 Hz), 109.17 ppm. MS (EI): 196 (100). HRMS (EI) for $C_8H_6F_2N_4$ Calcd: 196.0561; Found: 196.0555. IR (KBr): ν = 3140, 2359, 1897, 1758, 1591, 1471, 1364, 1288, 1124, 1041, 908, 770 cm^{-1}. Mp：80.1 – 81.2 ^{o}C.

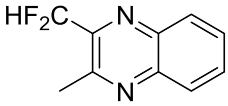

2-(Difluoromethyl)-3-methyl-quinoxaline 5m. The general procedure **A** conducted with 2-chloro-3-methyl-quinoxaline (89 mg, 0.50 mmol), [(SIPr)Ag(CF_2H)] (357 mg, 0.650 mmol), Pd(dba)$_2$ (14 mg, 5.0 mol%), DPEPhos (28.0 mg, 10.0 mol%) in dry toluene (2.0 mL) gave 70 mg (72%) of 2-(difluoromethyl)-3-methyl-quinoxaline as a yellow solid. ^{1}H NMR (400 MHz, $CDCl_3$) δ 8.04 (t, J = 8 Hz, 2 H), 7.79 (t, J = 7.5 Hz, 1 H), 7.73 (t, J = 7.5 Hz, 1 H), 6.80 (t, J = 52.0 Hz, 1 H), 2.93 (s, 3 H); ^{19}F NMR (376 MHz, $CDCl_3$) δ -115.05 (d, J = 52.6 Hz, 2 F); ^{13}C NMR (125 MHz, $CDCl_3$) δ 152.16, 145.77, 142.38, 139.23, 131.49, 129.76, 129.35, 128.51, 116.56 (t, J = 240 Hz), 21.88 ppm. MS (EI): 194 (100). HRMS (EI) for $C_{10}H_8N_2F_2$ Calcd: 194.0656; Found: 194.0655. IR (KBr): ν = 3002, 2679, 1566, 1491, 1378, 1360, 1186, 1126, 1077, 771 cm^{-1}. Mp：123 – 125 ^{o}C.

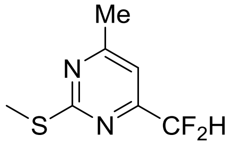

4-(Difluoromethyl)-6-methyl-2-methylsulfanyl-pyrimidine 5n. The general procedure **A** conducted with 4-chloro-6,7-dimethoxy-quinazoline (87 mg, 0.50 mmol), [(SIPr)Ag(CF_2H)] (357 mg, 0.650 mmol), Pd(dba)$_2$ (14 mg, 5.0 mol%), DPEPhos

(28.0 mg, 10.0 mol%) in dry toluene (2.0 mL) gave 81.7 mg (86%) of 4-(difluoromethyl)-6-methyl-2-methylsulfanyl-pyrimidine as a yellow oil. ^{1}H NMR (400 MHz, $CDCl_3$) δ 7.08 (s, 1 H), 6.42 (t, *J* = 54.9 Hz, 1 H), 2.56 (s, 3 H), 2.52 (s, 3 H); ^{19}F NMR (376 MHz,$CDCl_3$) δ -119.78 (d, *J* = 54.9 Hz, 2 F); ^{13}C NMR (126 MHz, $CDCl_3$) δ 172.94, 169.43, 159.93 (t, *J* = 26.4 Hz), 112.57 (t, *J* = 241.9 Hz), 111.17 (t, *J* = 3.1 Hz), 24.36, 14.06 ppm. MS (EI): 190 (100). HRMS (EI) for $C_7H_8F_2N_2S$ Calcd: 190.0376; Found: 190.0381. IR (KBr): ν = 2930, 1584, 1553, 1407, 1393, 1261, 1106, 1057, 910, 733 cm^{-1}.

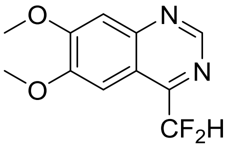

4-(Difluoromethyl)-6,7-dimethoxy-quinazoline 5o. The general procedure **A** conducted with 4-chloro-6,7-dimethoxy-quinazoline (112 mg, 0.500 mmol), [(SIPr)Ag(CF_2H)] (357 mg, 0.650 mmol), $Pd(dba)_2$ (14 mg, 5.0 mol%), DPEPhos (28.0 mg, 10.0 mol%) in dry toluene (2.0 mL) gave 104.4 mg (87%) of 4-(difluoromethyl)-6,7-dimethoxy-quinazoline as a yellow solid. ^{1}H NMR (400 MHz, $CDCl_3$) δ 9.16 (s, 1 H), 7.55 (s, 1 H), 7.40 (s, 1 H), 6.84 (t, *J* = 54.1 Hz, 1 H), 4.09 (s, 3 H), 4.07 (s, 3 H); ^{19}F NMR (376 MHz, $CDCl_3$) δ -112.65 (d, *J* = 54.2 Hz, 2 F); ^{13}C NMR (126 MHz, $CDCl_3$) δ 156.61, 152.83, 151.15, 150.06, 148.93, 117.08, 116.95 (t, *J* = 243.2 Hz), 107.07, 101.51 (t, *J* = 3.7 Hz), 56.49, 56.31 ppm. MS (EI): 240 (100). HRMS (EI) for $C_{11}H_{10}F_2N_2O_2$ Calcd: 240.0710; Found: 240.0714. IR (KBr): ν = 3008, 1616, 1473, 1381, 1308, 1182, 1083, 992, 906, 732, 655 cm^{-1}. Mp：168.1 – 169.5 ^{o}C.

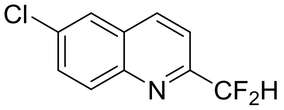

6-Chloro-2-(difluoromethyl)quinoline 5p. The general procedure **A** conducted with 6-chloro-2-chloroquinoline (99 mg, 0.50 mmol), [(SIPr)Ag(CF_2H)] (357 mg, 0.650 mmol), $Pd(dba)_2$ (14 mg, 5.0 mol%), DPEPhos (28.0 mg, 10.0 mol%) in dry toluene (2.0 mL) gave 80 mg (75%) of 6-chloro-2-(difluoromethyl)quinoline as a yellow solid. ^{1}H NMR (400 MHz, $CDCl_3$) δ 8.24 (d, *J* = 12.0 Hz, 1 H), 8.08 (d, *J* = 8.0 Hz, 1 H),

7.87 (s, 1 H), 7.76-7.71 (m, 2 H), 6.76 (t, *J* = 56.0 Hz, 1 H); ^{19}F NMR (376 MHz, $CDCl_3$) δ -114.39 (d, *J* = 56.4 Hz, 2 F); ^{13}C NMR (125 MHz, $CDCl_3$) δ 152.97 (t, *J* = 26.3 Hz), 145.48, 136.88, 133.85, 131.33, 131.26, 129.23 (t, *J* = 101.3 Hz), 126.38, 117.73 (t, *J* = 1.3 Hz), 114.36 (t, *J* = 237.5 Hz) ppm. MS (EI): 213 (100). HRMS (EI) for $C_{10}H_6NF_2Cl$ Calcd: 213.0157; Found: 213.0155. IR (KBr): ν = 3011, 1802, 1751, 1598, 1495, 1360, 1188, 1152, 1096, 1127, 839, 806, 778 cm^{-1}. Mp：132 – 133 ^{o}C.

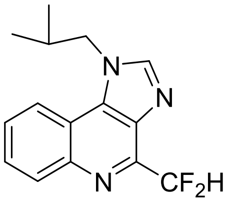

4-(Difluoromethyl)-1-isopentyl-1*H*-imidazo[4,5-*c*]quinolone 6a. The general procedure **A** conducted with 4-bromo-1-isopentyl-1H-imidazo[4,5-c]quinolone (152 mg, 0.500 mmol), [(SIPr)Ag(CF_2H)] (357 mg, 0.650 mmol), Pd(dba)$_2$ (14 mg, 5.0 mol%), DPEPhos (28.0 mg, 10.0 mol%) in dry toluene (2.0 mL) gave 127 mg (93%) of 4-(difluoromethyl)-1-isopentyl-1*H*-imidazo[4,5-c]quinolone as a yellow solid. ^{1}H NMR (400 MHz, $CDCl_3$) δ 8.37 (d, *J* = 8.0 Hz, 1 H), 8.17 (d, *J* = 8.0 Hz, 1 H), 8.00 (s, 1 H), 7.22 (t, *J* = 52.0 Hz, 1 H), 4.41 (d, *J* = 8.0 Hz, 2 H), 2.31 – 2.43 (m, 1 H), 1.07 (d, *J* = 8.0 Hz, 6 H); ^{19}F NMR (376 MHz, $CDCl_3$) δ -116.28 (d, *J* = 52.6 Hz, 2 F); ^{13}C NMR (125 MHz, $CDCl_3$) δ 145.82, 144.74, 143.54, 135.12, 134.25, 131.56, 127.88, 127.77, 120.01, 118.59, 113.73 (t, *J* = 241.3 Hz), 55.07, 28.82, 19.76 ppm. MS (EI): 275 (100). HRMS (EI) for $C_{15}H_{15}N_3F_2$ Calcd: 275.1234; Found: 275.1235. IR (KBr): ν = 2964, 2874, 1735, 1578, 1523m 1472, 1391, 1372, 1216, 1125, 1001, 913, 823 cm^{-1}. Mp：142 – 145 ^{o}C.

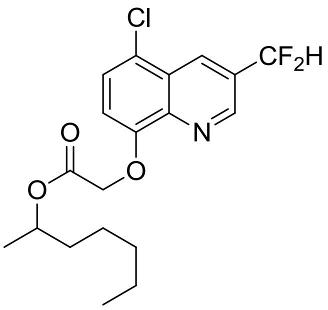

Heptan-2-yl 2-((5-chloro-3-(difluoromethyl) quinolin-8-yl)oxy)acetate 6b. The general procedure **B** conducted with heptan-2-yl 2-((5-chloro-3-iodo-quinolin-8-yl)oxy)acetate (231 mg, 0.500 mmol), [(SIPr)Ag(CF_2H)] (357 mg, 0.650 mmol), $Pd(dba)_2$ (28 mg, 10.0 mol%), DPEPhos (56.0 mg, 20.0 mol%) in dry toluene (2.0 mL) gave 113 mg (58%) of heptan-2-yl 2-((5-chloro-3-(difluoromethyl) quinolin-8-yl)oxy)acetate as a yellow oil. ^{1}H NMR (400 MHz, $CDCl_3$) δ 9.06 (s, 1 H), 8.63 (s, 1 H), 7.53 (d, *J* = 8.4 Hz, 1 H), 6.95 (d, *J* = 8.4 Hz, 1 H), 6.90 (t, *J* = 55.6 Hz, 1 H), 5.12 – 4.73 (m, 2 H), 1.44 (ddd, *J* = 23.6, 15.9, 8.9 Hz, 2 H), 1.34 – 0.98 (m, 7 H), 0.80 (t, *J* = 6.5 Hz, 2 H); ^{19}F NMR (376 MHz, $CDCl_3$) δ -112.17 (d, *J* = 55.5 Hz, 2 F);^{13}C NMR (101 MHz, $CDCl_3$) δ 167.84, 152.86, 146.57 (t, *J* = 5.3 Hz), 141.29, 130.95 (t, *J* = 6.7 Hz), 128.53 (t, *J* = 23.1 Hz), 127.09, 125.94, 124.54, 123.71, 113.33 (t, *J* = 240.1 Hz), 110.85, 74.29, 66.37, 35.60, 31.35, 24.82, 22.37, 19.67, 13.81 ppm. MS (EI): 242 (100). HRMS (EI) for $C_{19}H_{22}ClNO_3F_2$ Calcd: 385.1263; Found: 385.1256. IR (KBr): ν = 2957, 2861, 1751, 1571, 1498, 1461, 1367, 1339, 1214, 1185, 1037, 924, 817 cm^{-1}.

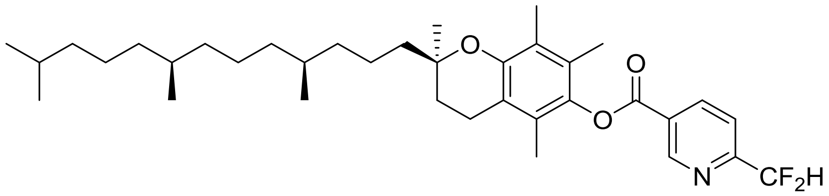

(*S*)-2,5,7,8-Tetramethyl-2-((4*R*,8*R*)-4,8,12-trimethyltridecyl)chroman-6-yl-6-(difluoromethyl)nicotinate 6c. The general procedure **A** conducted with (S)-2,5,7,8-tetramethyl-2-((4R,8R)-4,8,12-trimethyltridecyl)chroman-6-yl-6-bromo-nicotinate (307 mg, 0.5 mmol), [(SIPr)Ag(CF_2H)] (357 mg, 0.650 mmol), $Pd(dba)_2$ (14 mg, 5.0 mol%), DPEPhos (28.0 mg, 10.0 mol%) in dry toluene (2.0 mL) gave 269 mg (92%) of S)-2,5,7,8-tetramethyl-2-((4R,8R)-4,8,12-trimethyltridecyl)chroman-6-yl-6-(difluoromethyl)nicotinate as a yellow oil. ^{1}H NMR (400 MHz, $CDCl_3$) δ 9.49 (s, 1 H), 8.67 (dd, *J* = 8.0 Hz, 1 H), 7.83 (d, *J* = 8.0 Hz, 1 H), 6.74 (t, *J* = 56.0 Hz, 1 H), 2.66 (t, *J* = 4.0 Hz, 2 H), 2.17 (s, 3 H), 2.09 (s, 3 H), 2.05 (s, 3 H), 0.8 - 2.0 (m, 38 H); ^{19}F NMR (376 MHz, $CDCl_3$) δ -116.43 (d, *J* = 52.6 Hz, 2 F); ^{13}C NMR (125 MHz, $CDCl_3$) δ 163.08, 156.56 (t, *J* = 25.0 Hz), 150.99, 149.84, 140.26, 139.07, 127.19,

126.60, 124.89, 123.40, 120.03, 117.67, 115.31, 113.39, 111.47, 75.27, 39.41, 37.59, 37.56, 37.50, 37.45, 37.43, 37.33, 32.80, 32.71, 27.94, 24.83, 24.48, 22.74, 22.65, 21.07, 20.66, 19.78, 19.72, 13.07, 12.22, 11.88 ppm. MS (EI): 57 (100), 585 (3.1). HRMS (EI) for $C_{36}H_{53}NO_3F_2$ Calcd: 585.3994; Found: 585.3988. IR (KBr): ν =2926, 2867, 1741, 1600, 1462, 1414, 1279, 1240, 1100, 1054, 909, 909, 734 cm^{-1}.

Stoicihiometric reaction of [(Xantphos) Pd(3-Py)(Br)] with [(SIPr)Ag(CF_2H)]

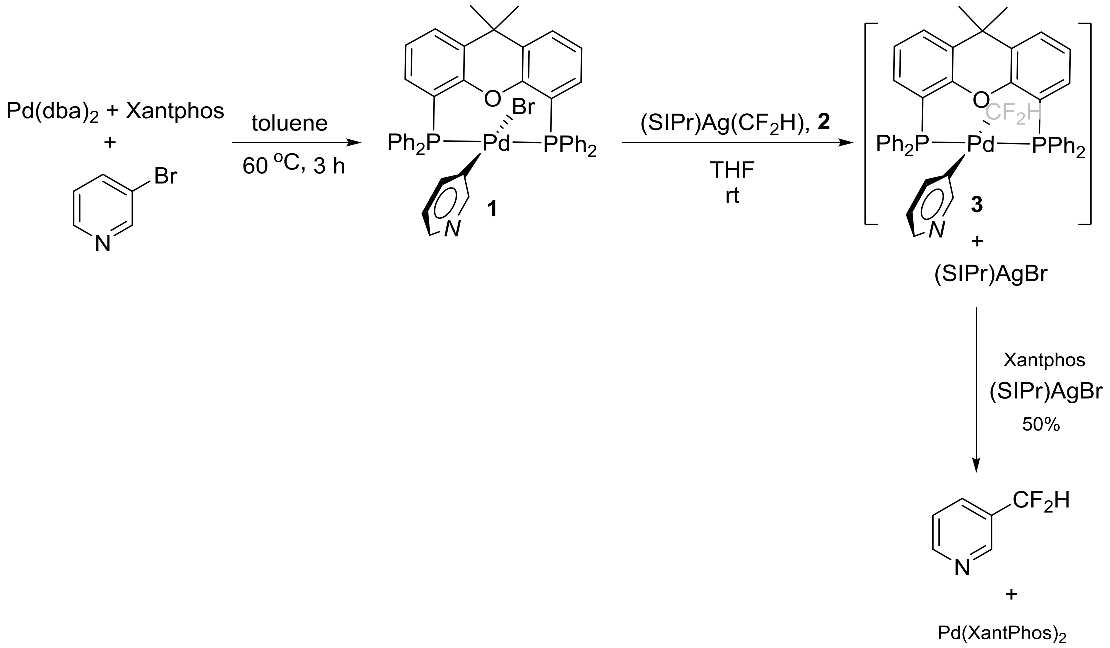

A Schlenk tube was charged with Pd(dba)2 (287 mg, 0.500 mmol) and Xantphos (289 mg, 0.500 mmol), briefly evacuated and backfilled with argon. 3-Bromopyridine (785 mg, 5.00 mmol, 10.0 eq) was added and toluene was used as solvent. The Schlenk tube was sealed with a Teflon valve and the reaction was stirred at 60 ℃ for 3 ~4 h when the mixture turned into green. The mixture was cooled to room temperature and flitered through a short plug with Celite and washed with dichloromethane .The solvent was evaporated under vacuum until 20 % ~30 % solvent was left. Hexane was added to give a yellow solid. The solid was recrystallized from CH_2Cl_2/petane to give the final product [(Xantphos) Pd(3-Py)(Br)] as a yellow solid (327 mg, 75%).

A Schlenk tube was charged with [(Xantphos) Pd(3-Py)(Br)] (21.0 mg, 0.250 mmol) and [(SIPr)Ag(CF_2H)] (137 mg, 0.250 mmol), briefly evacuated and backfilled with argon, then dry THF (2.0 mL) was added as solvent. The mixture was stirred at room temperature for 1 h, and monitored by ^{19}F NMR spectrum. The formation of 3-difluoromethylpyridine was observed in 50% yield as determined by ^{19}F NMR spectrum using benzotrifluoride as the internal standard.

Reference

[1] Y. Gu, Q. Shen, *Nat. Commun*. **2014**, *5*, 5405, doi: 10.1038/ncomms6405.
[2] C. -H. Wang, X. -T. Liu, X. -H. Chao, *Synthesis*, **1982**, *10*, 858.
[3] C. N. Johnson, S. F. Moss, D. R. Witty, WO2005030724A1, **2005**.
[4] E. Kiselev, K. Agama, Y. Pommier, M. Cushman, *J. Med. Chem.* **2012**, *55*,1682.

Spectrum of the Products

^{1}H NMR (400 MHz, $CDCl_3$) ethyl 5-(difluoromethyl)pyridine-3-carboxylate 4a

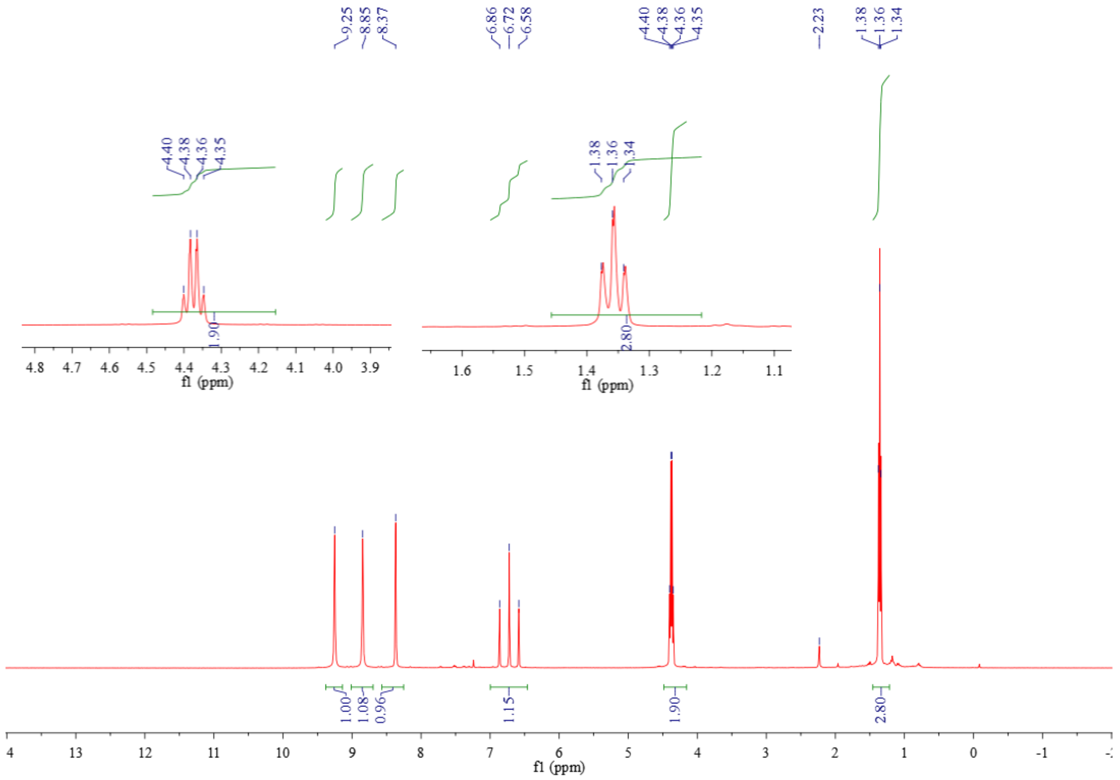

^{19}F NMR (376 MHz, $CDCl_3$) ethyl 5-(difluoromethyl)pyridine-3-carboxylate 4a

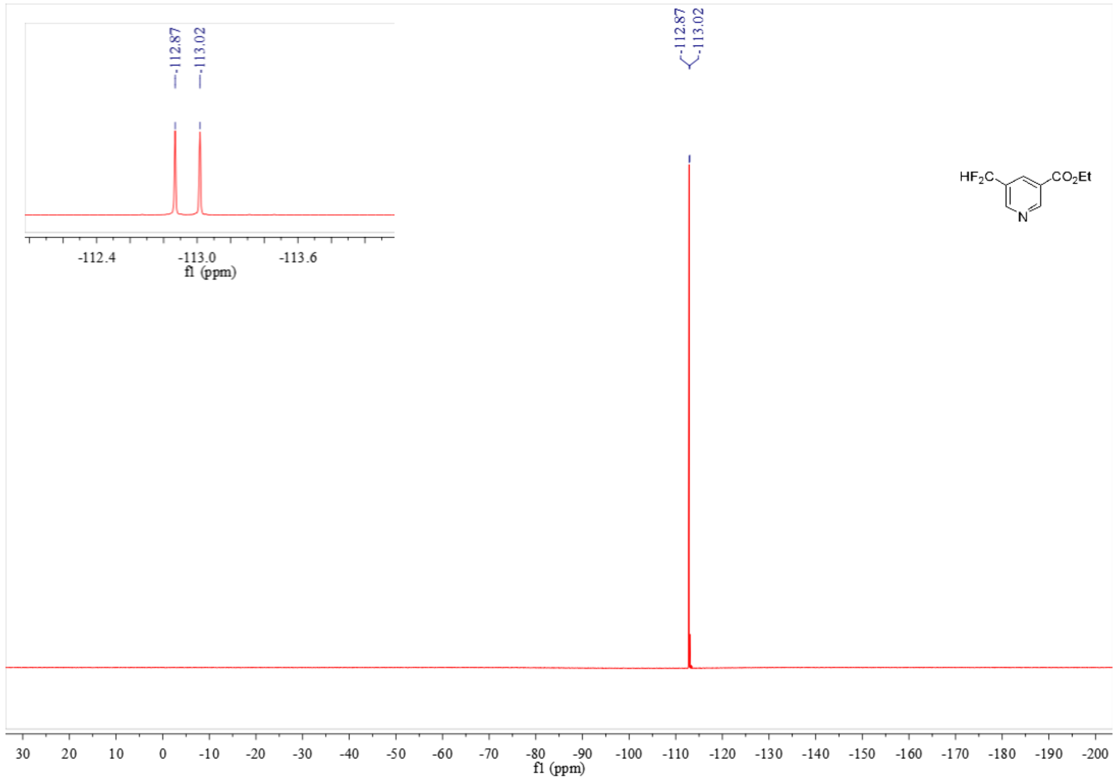

^{13}C NMR (101 MHz, $CDCl_3$) ethyl 5-(difluoromethyl)pyridine-3-carboxylate 4a

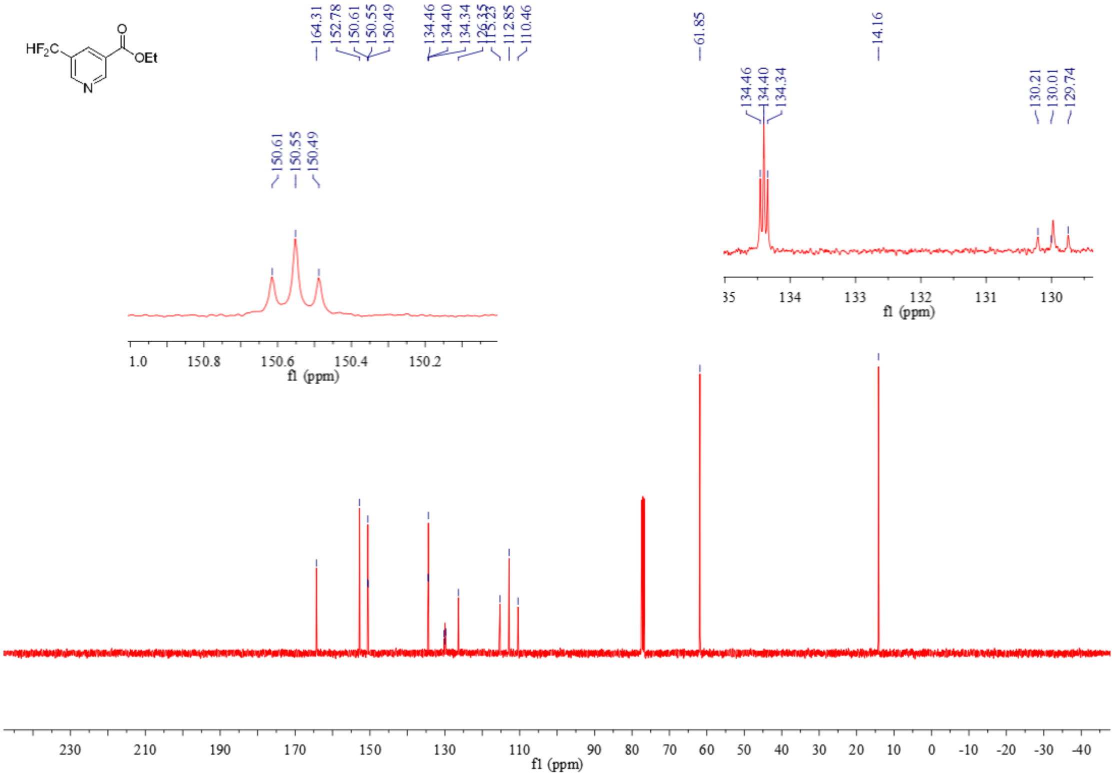

^{1}H NMR (400 MHz, $CDCl_3$) 5-(difluoromethyl)pyridine-3-carbonitrile 4b

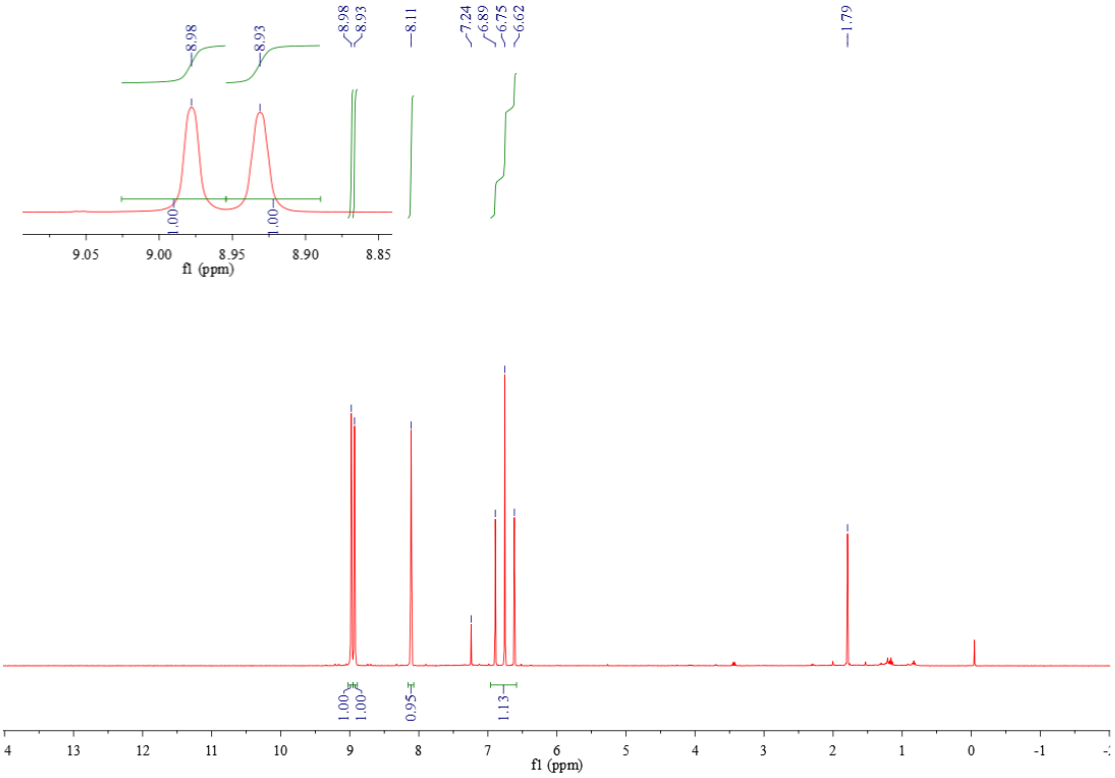

^{19}F NMR (376 MHz, $CDCl_3$) 5-(difluoromethyl)pyridine-3-carbonitrile 4b

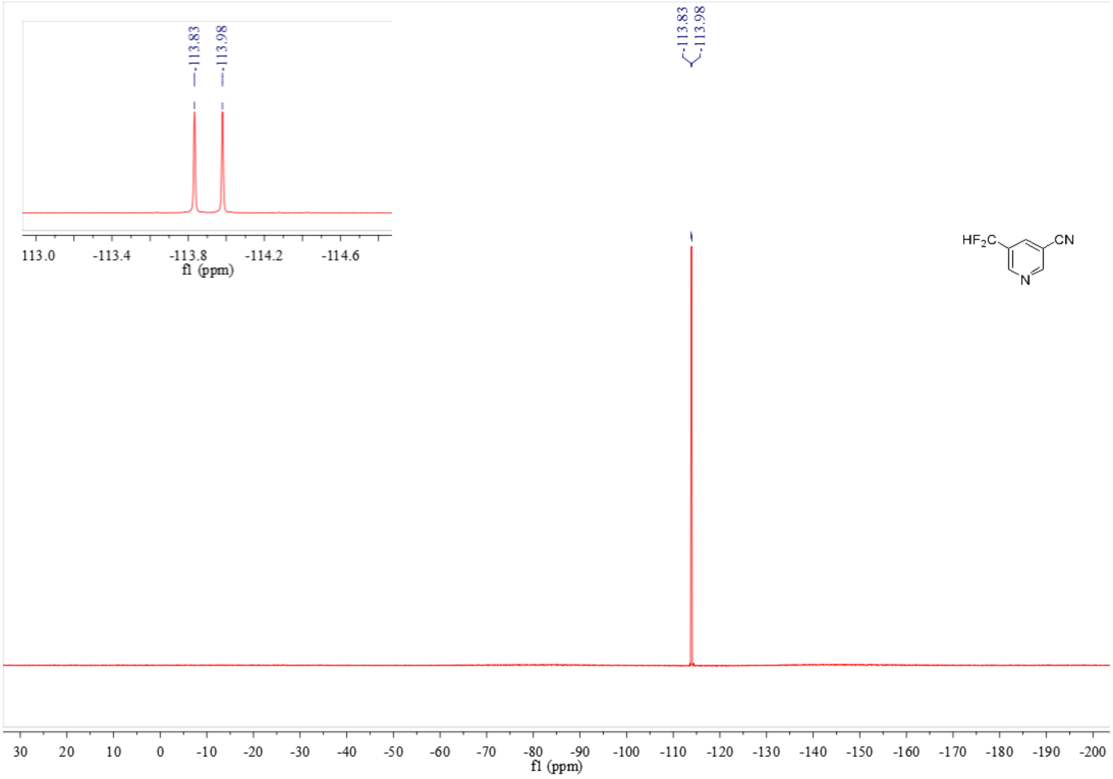

^{13}C NMR (101 MHz, $CDCl_3$) 5-(difluoromethyl)pyridine-3-carbonitrile 4b

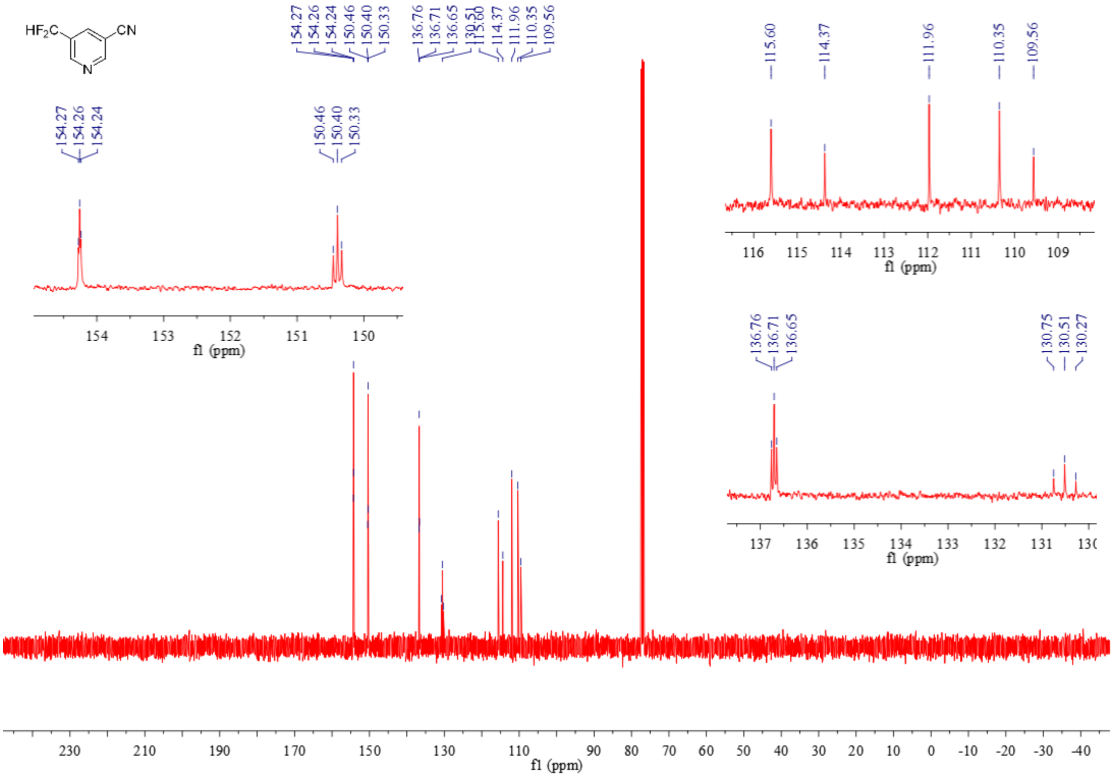

^{1}H NMR (400 MHz, $CDCl_3$) 3-benzyloxy-5-(difluoromethyl)pyridine 4c

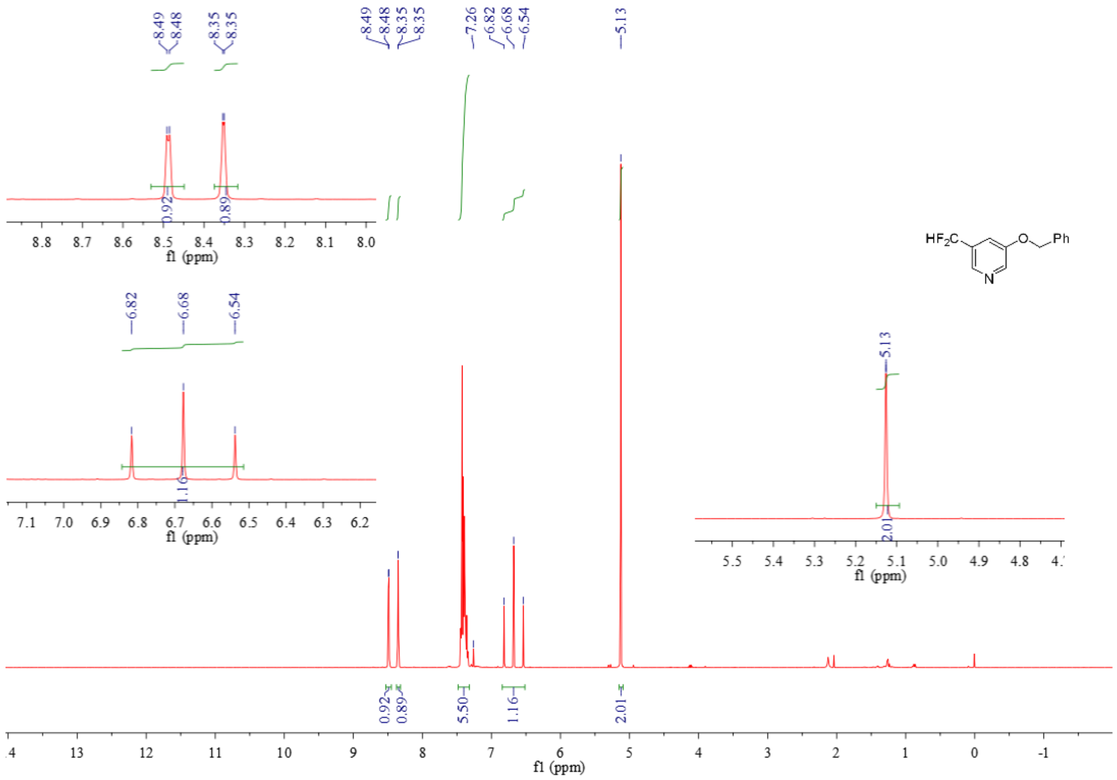

^{19}F NMR (376 MHz, $CDCl_3$) 3-benzyloxy-5-(difluoromethyl)pyridine 4c

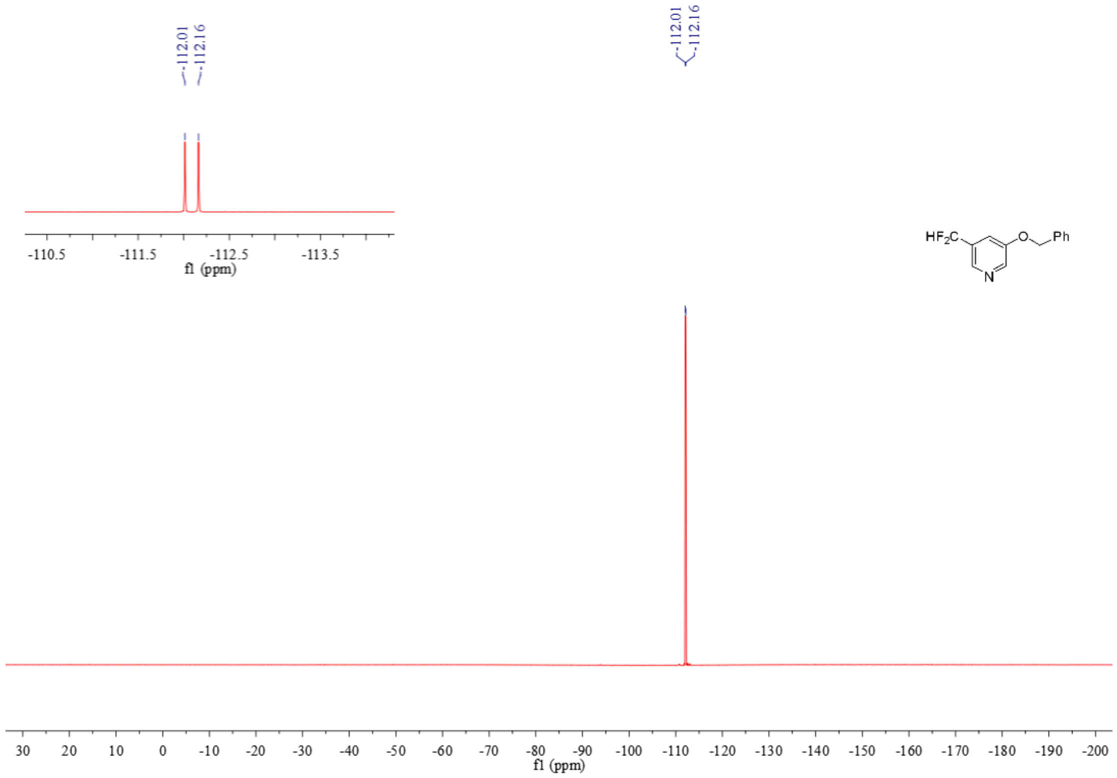

^{13}C NMR (101 MHz, $CDCl_3$) 3-benzyloxy-5-(difluoromethyl)pyridine 4c

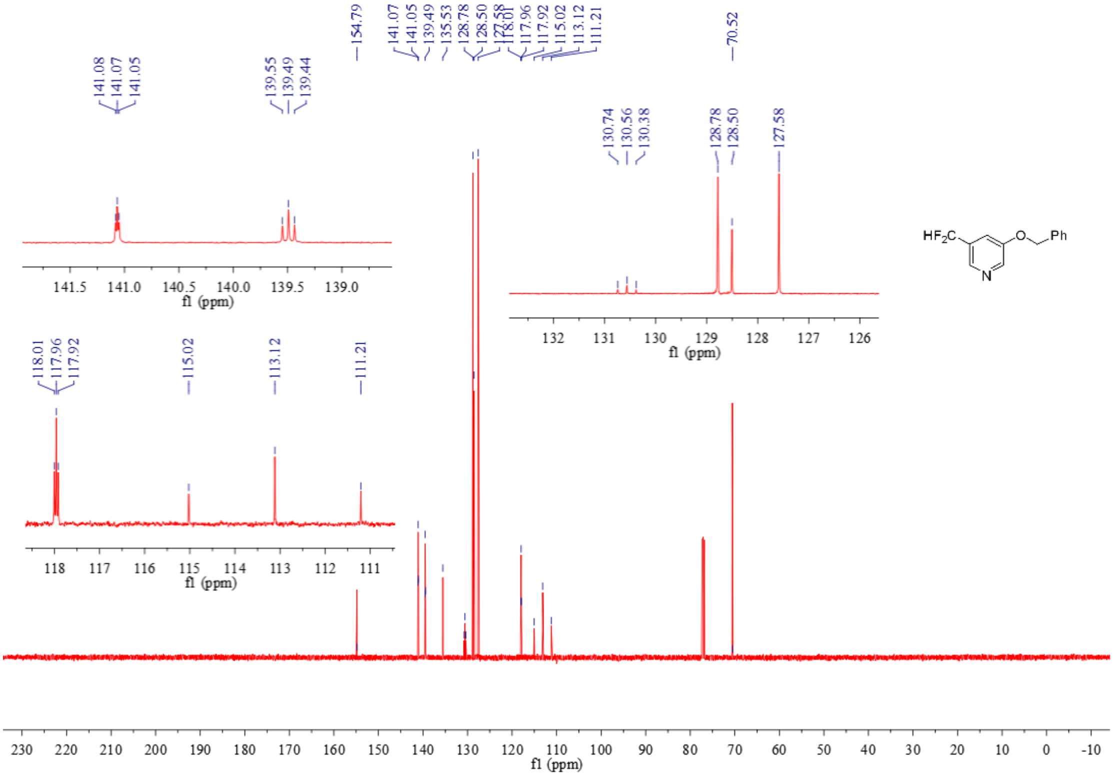

^{1}H NMR (400 MHz, $CDCl_3$) 3-(difluoromethyl)-5-(1,3-dioxolan-2-yl)pyridine 4d

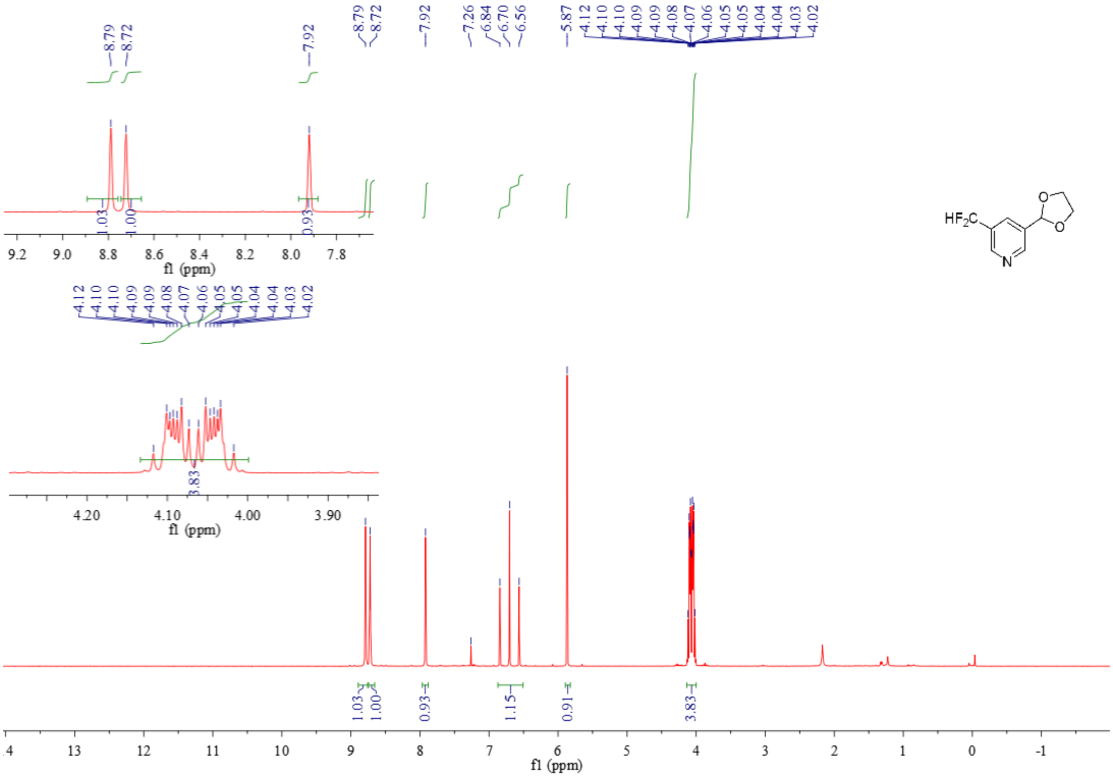

^{19}F NMR (376 MHz, $CDCl_3$) 3-(difluoromethyl)-5-(1,3-dioxolan-2-yl)pyridine 4d

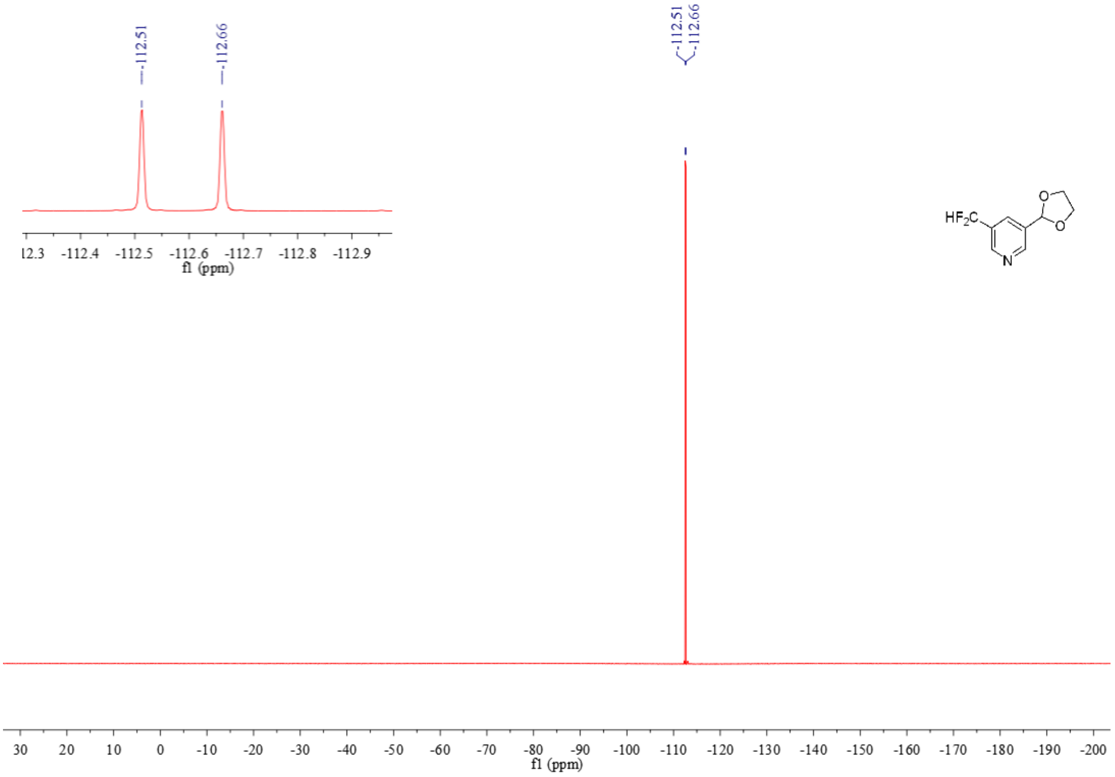

^{13}C NMR (101 MHz, $CDCl_3$) 3-(difluoromethyl)-5-(1,3-dioxolan-2-yl)pyridine 4d

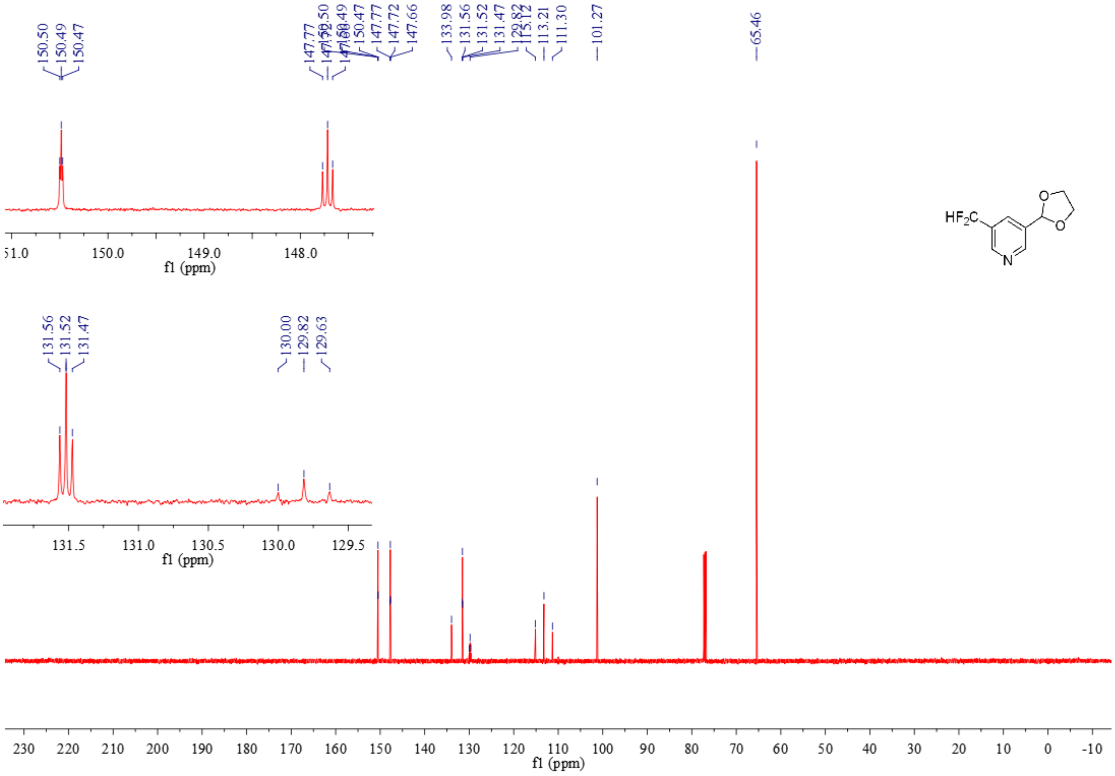

^{1}H NMR (400 MHz, $CDCl_3$) 5-(difluoromethyl)-2-nitro-pyridine 4e

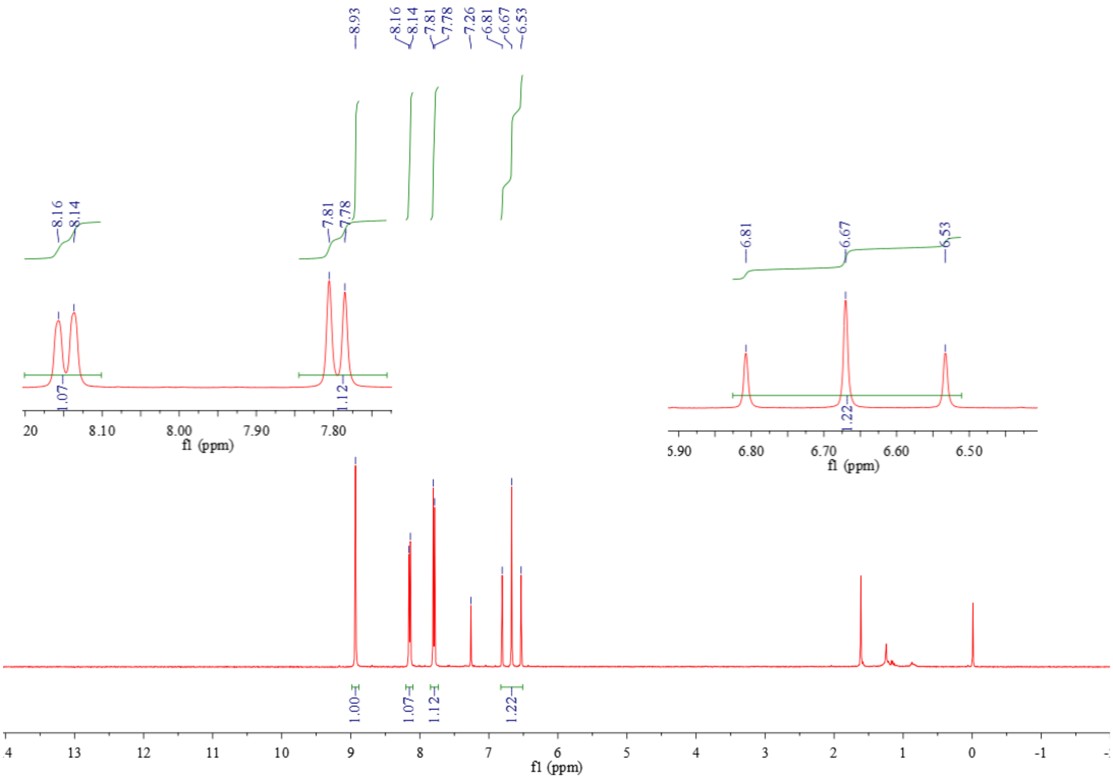

^{19}F NMR (376 MHz, $CDCl_3$) 5-(difluoromethyl)-2-nitro-pyridine 4e

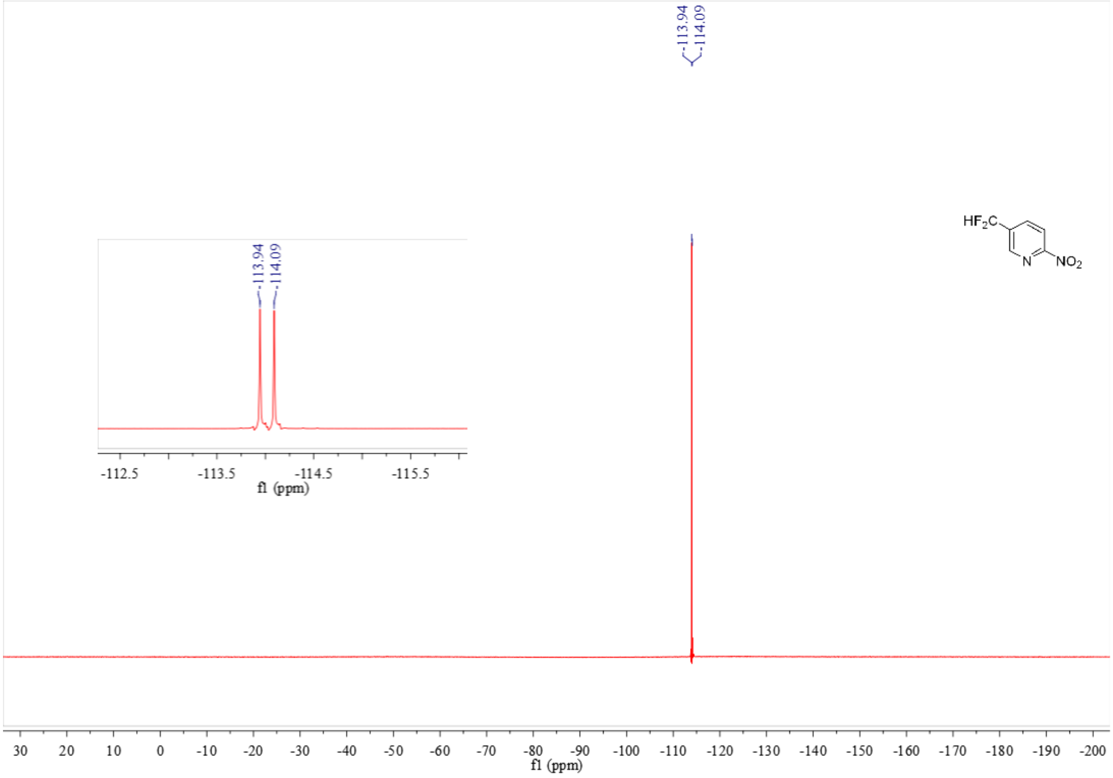

^{13}C NMR (101 MHz, $CDCl_3$) 5-(difluoromethyl)-2-nitro-pyridine 4e

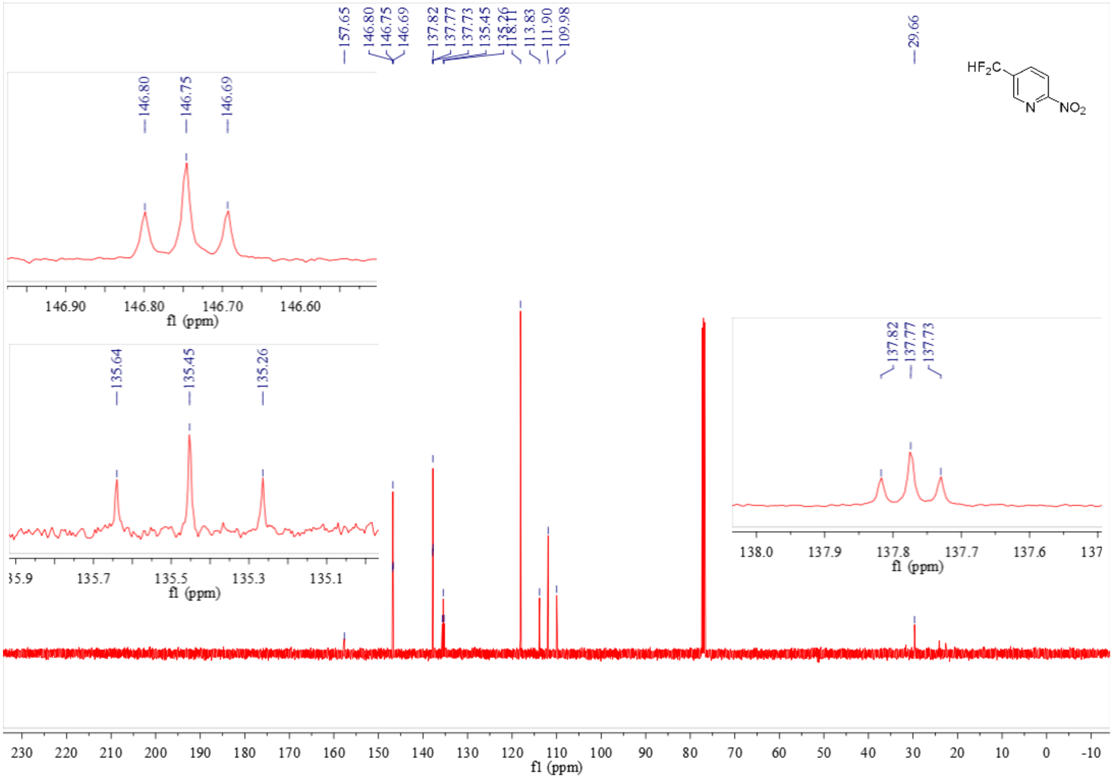

^{1}H NMR (400 MHz, $CDCl_3$) 6-(difluoromethyl)pyridine-3-carbonitrile 4f

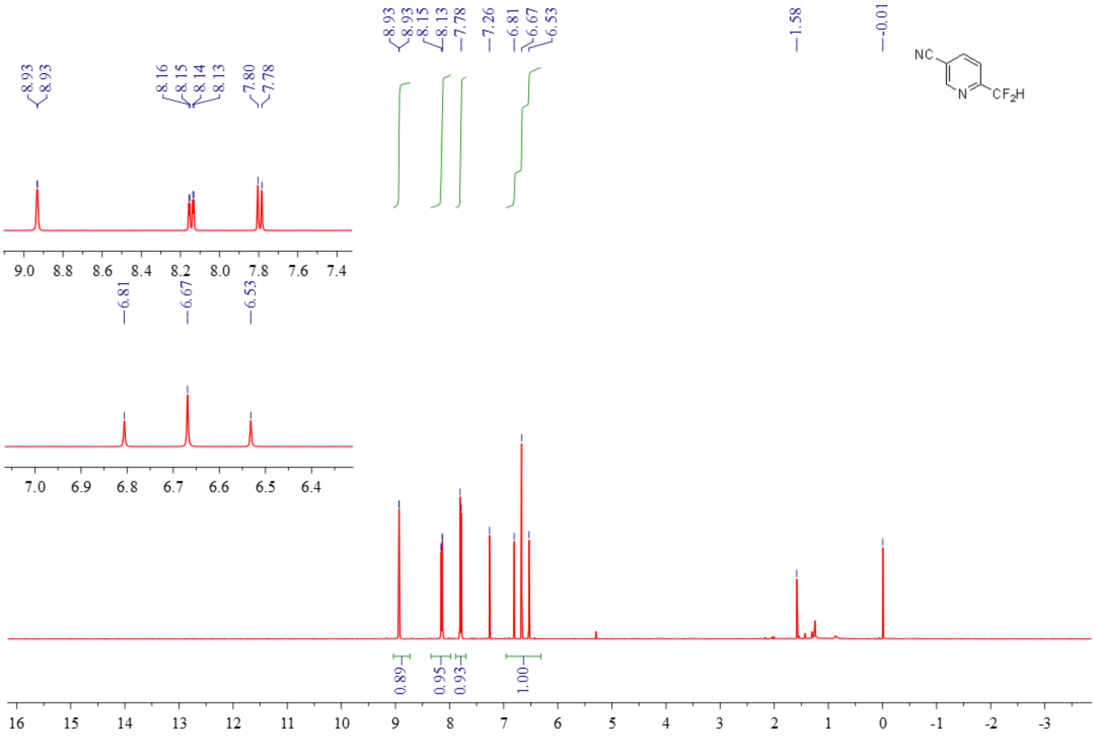

^{19}F NMR (376 MHz, $CDCl_3$) 6-(difluoromethyl)pyridine-3-carbonitrile 4f

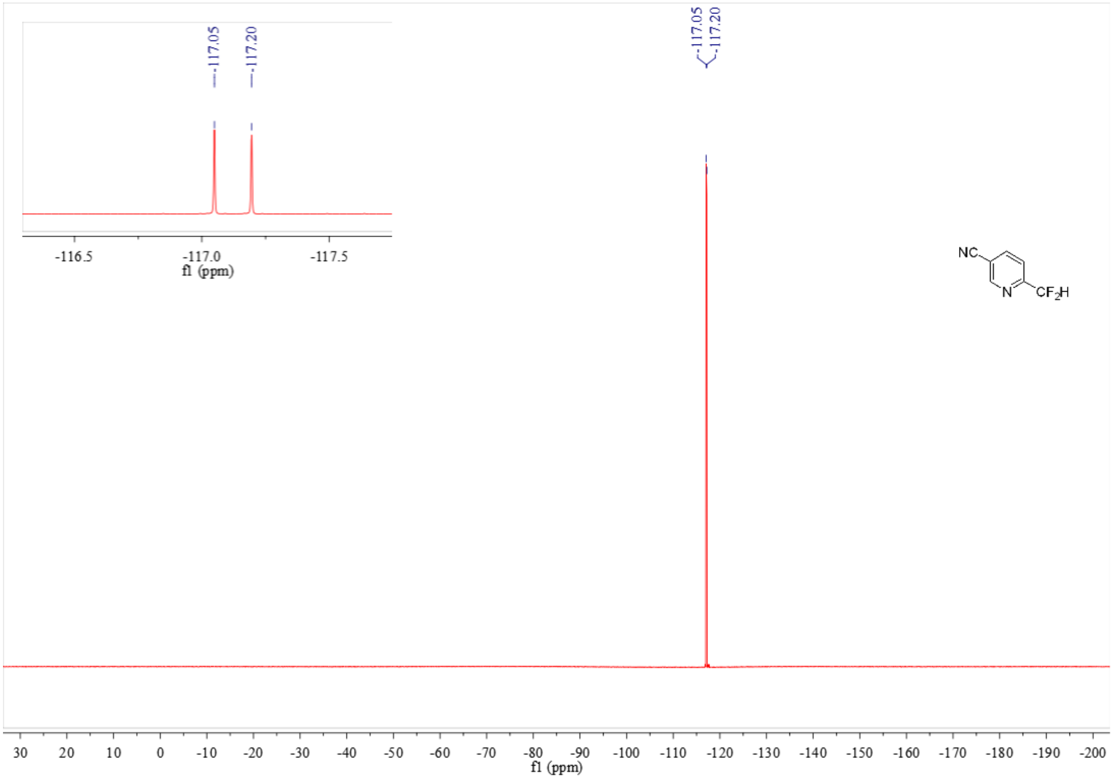

^{13}C NMR (101 MHz, $CDCl_3$) 6-(difluoromethyl)pyridine-3-carbonitrile 4f

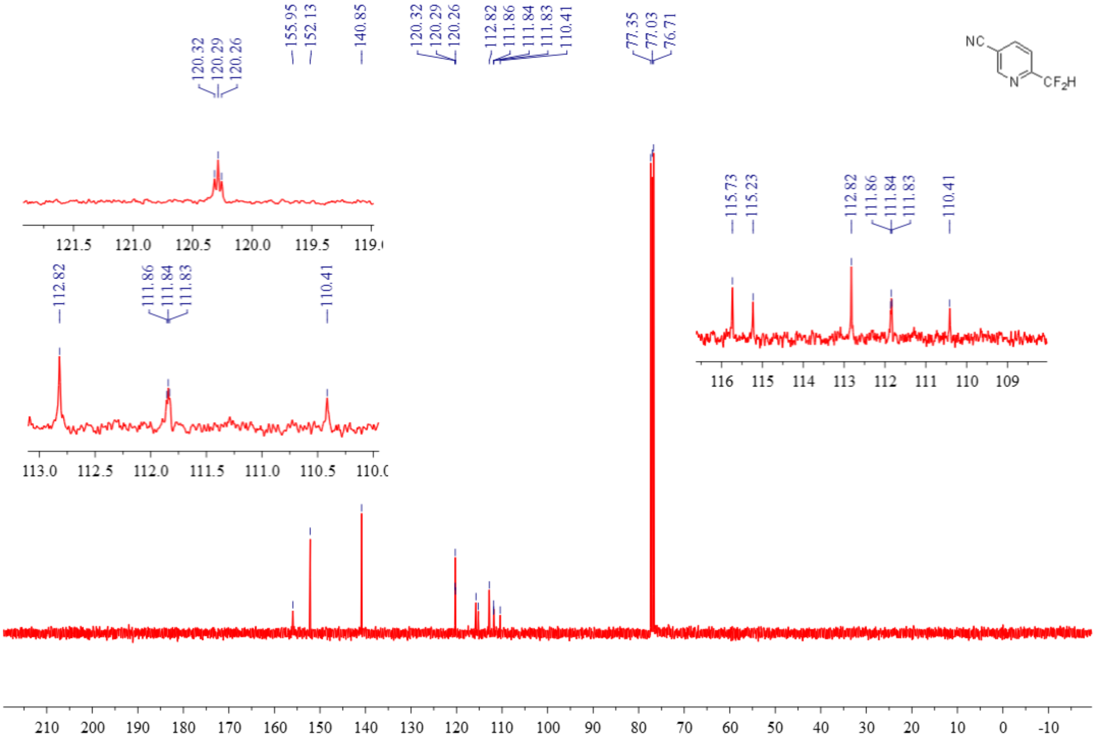

^{1}H NMR (400 MHz, $CDCl_3$) 2-(difluoromethyl)-5-nitro-pyridine 4g

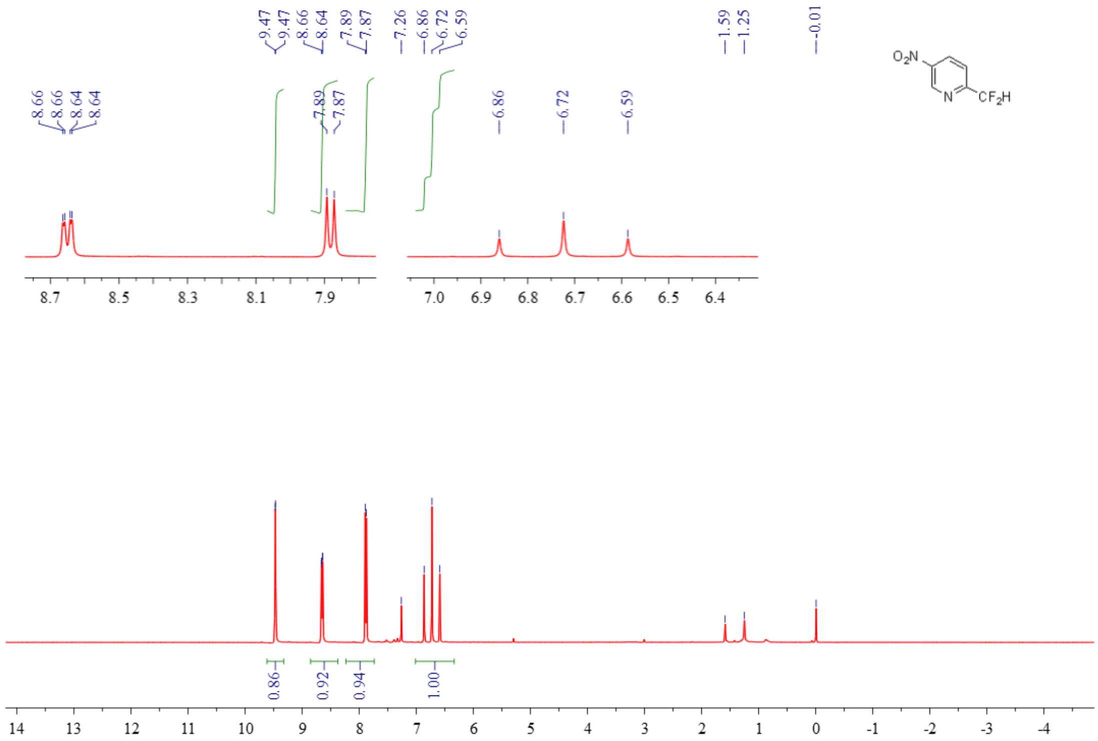

^{19}F NMR (376 MHz, $CDCl_3$) 2-(difluoromethyl)-5-nitro-pyridine 4g

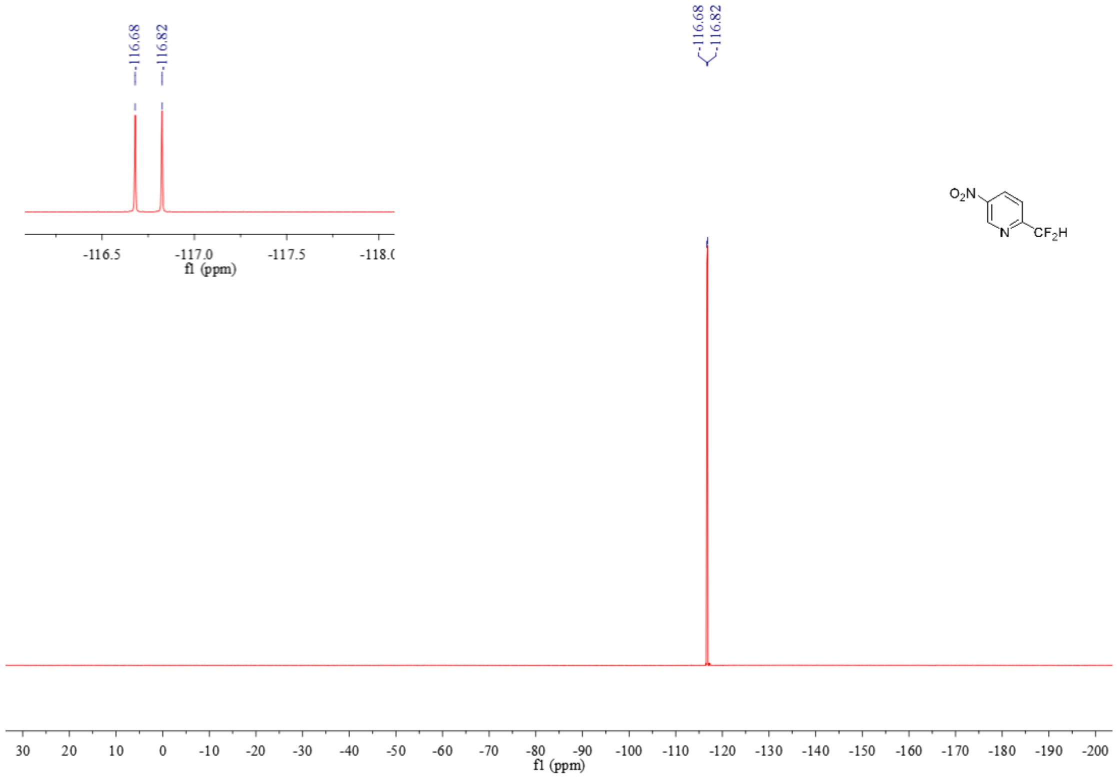

^{13}C NMR (101 MHz, $CDCl_3$) 2-(difluoromethyl)-5-nitro-pyridine 4g

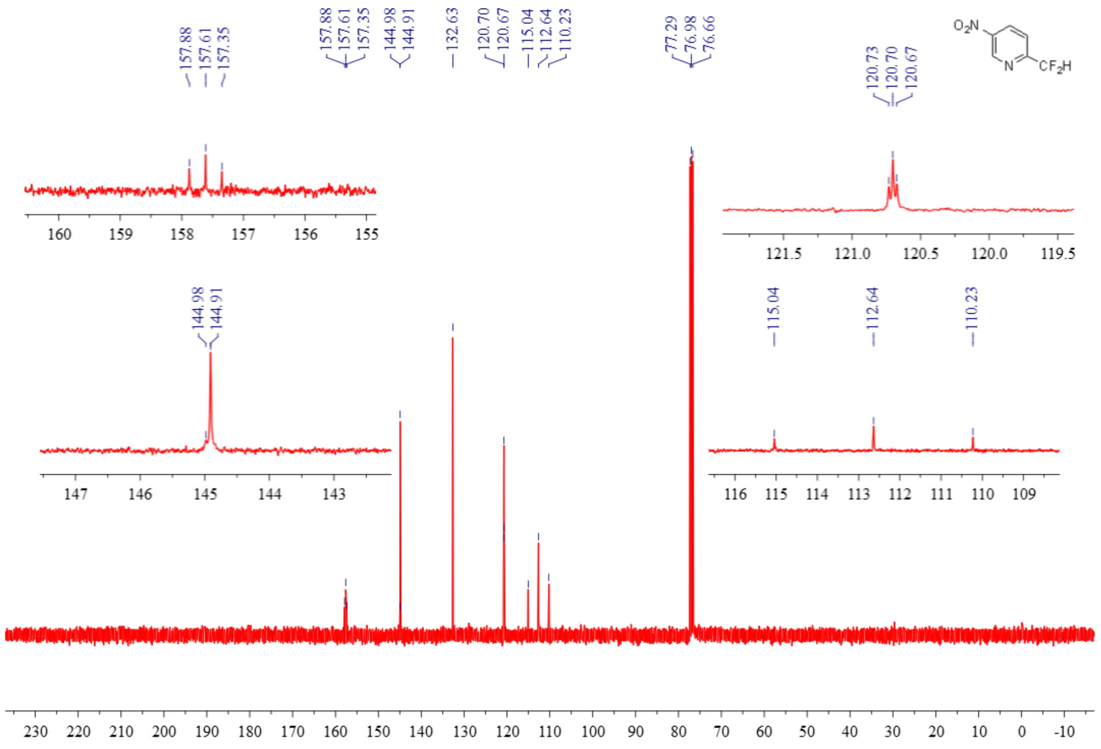

^{1}H NMR (400 MHz, $CDCl_3$) 6-(difluoromethyl)pyridine-2-carbonitrile 4h

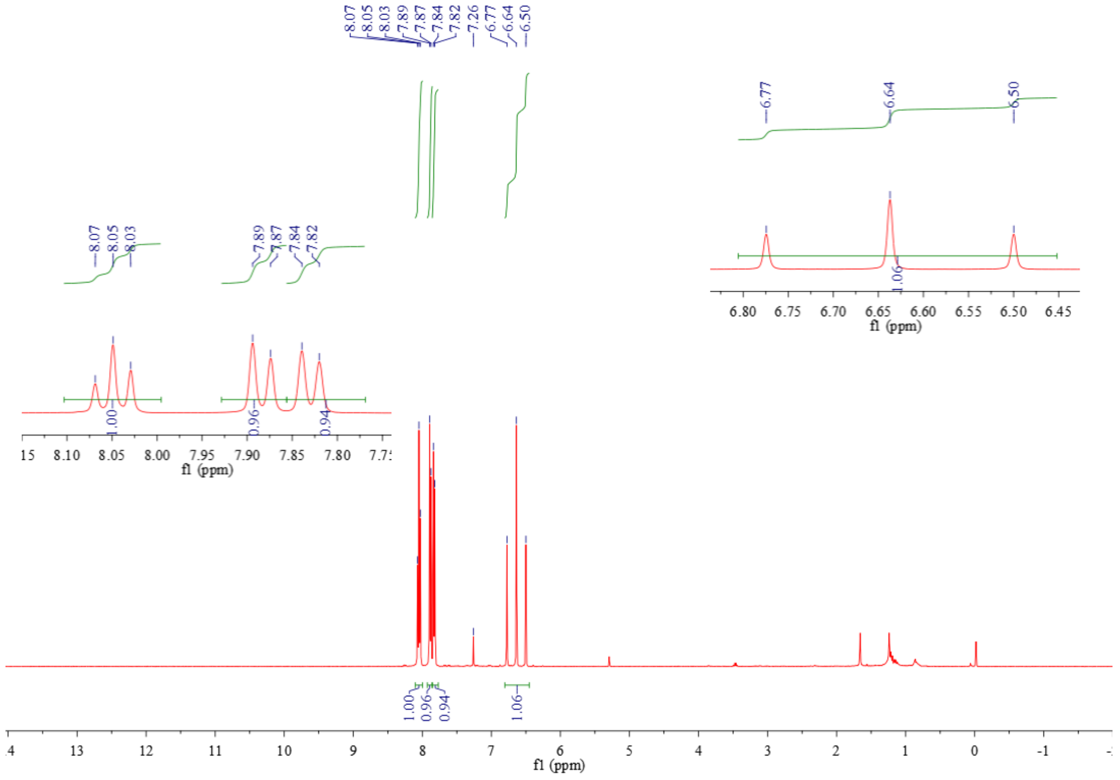

^{19}F NMR (376 MHz, $CDCl_3$) 6-(difluoromethyl)pyridine-2-carbonitrile 4h

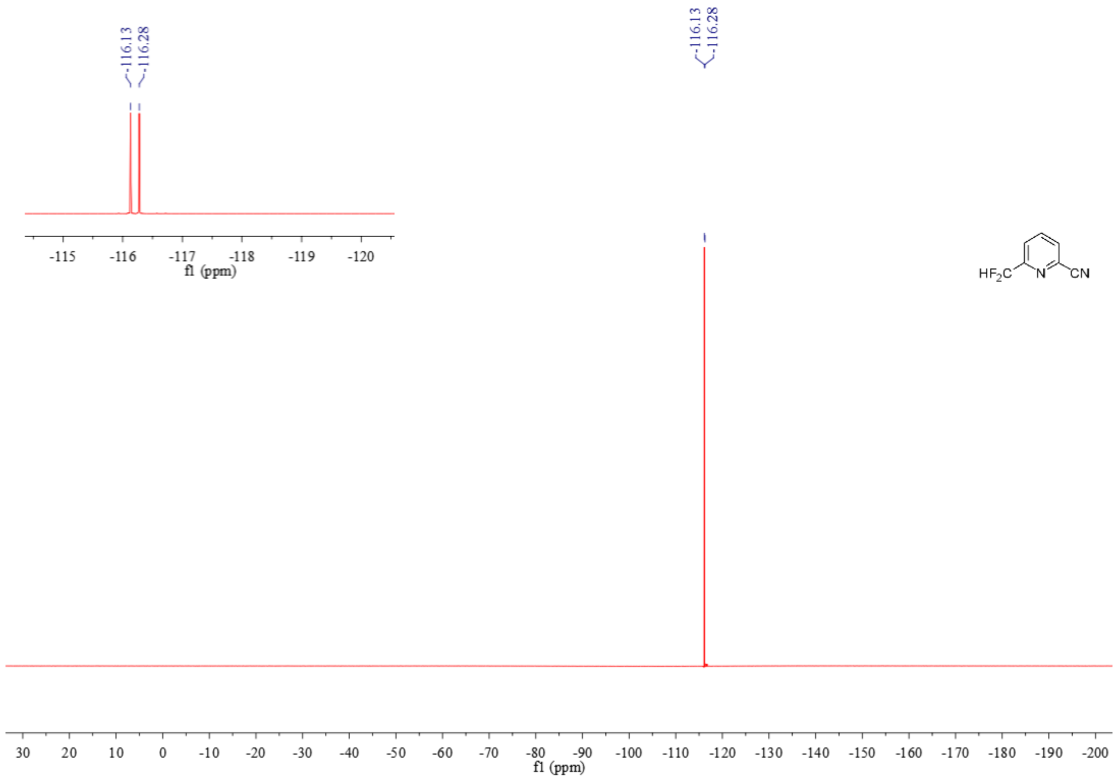

^{13}C NMR (101 MHz, $CDCl_3$) 6-(difluoromethyl)pyridine-2-carbonitrile 4h

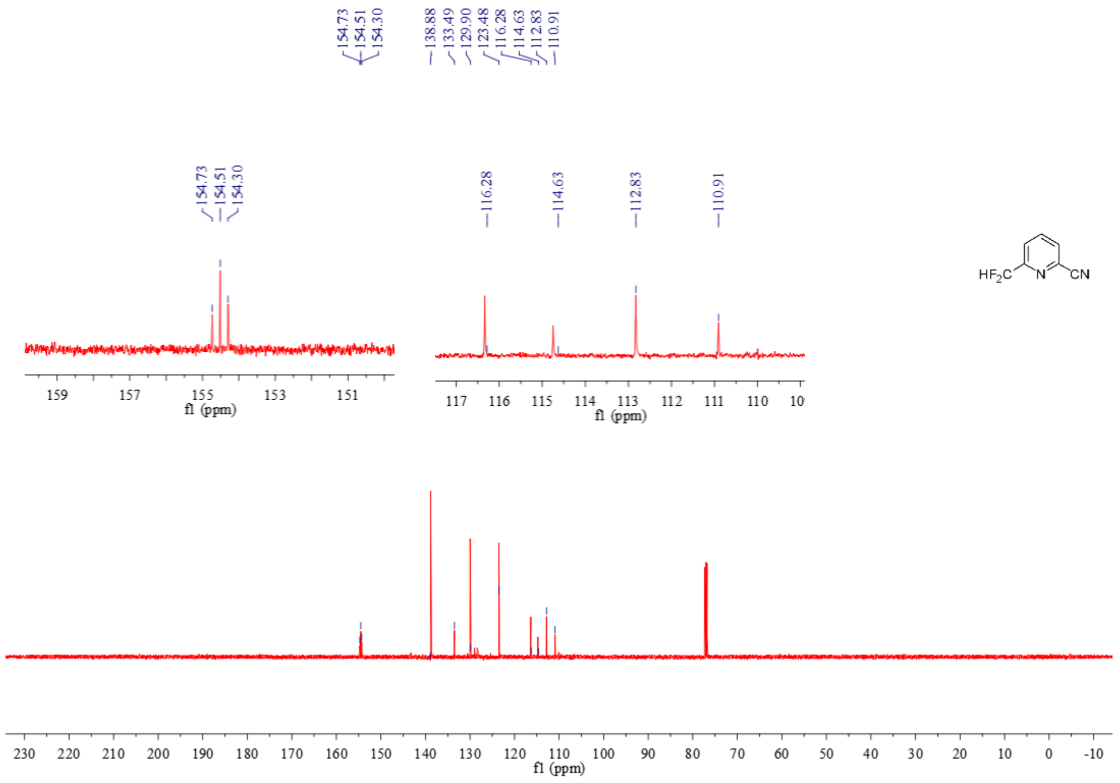

^{1}H NMR (400 MHz, $CDCl_3$) 2-(difluoromethyl)-3-methoxy-pyridine 4i

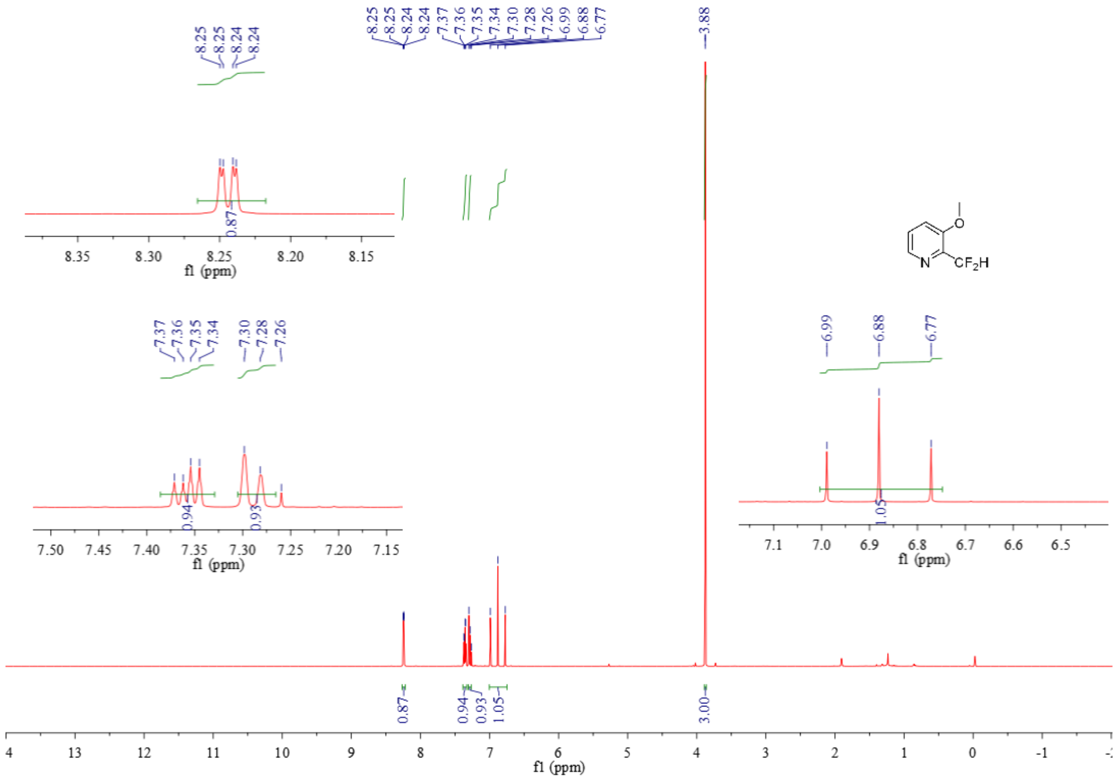

^{19}F NMR (376 MHz, $CDCl_3$) 2-(difluoromethyl)-3-methoxy-pyridine 4i

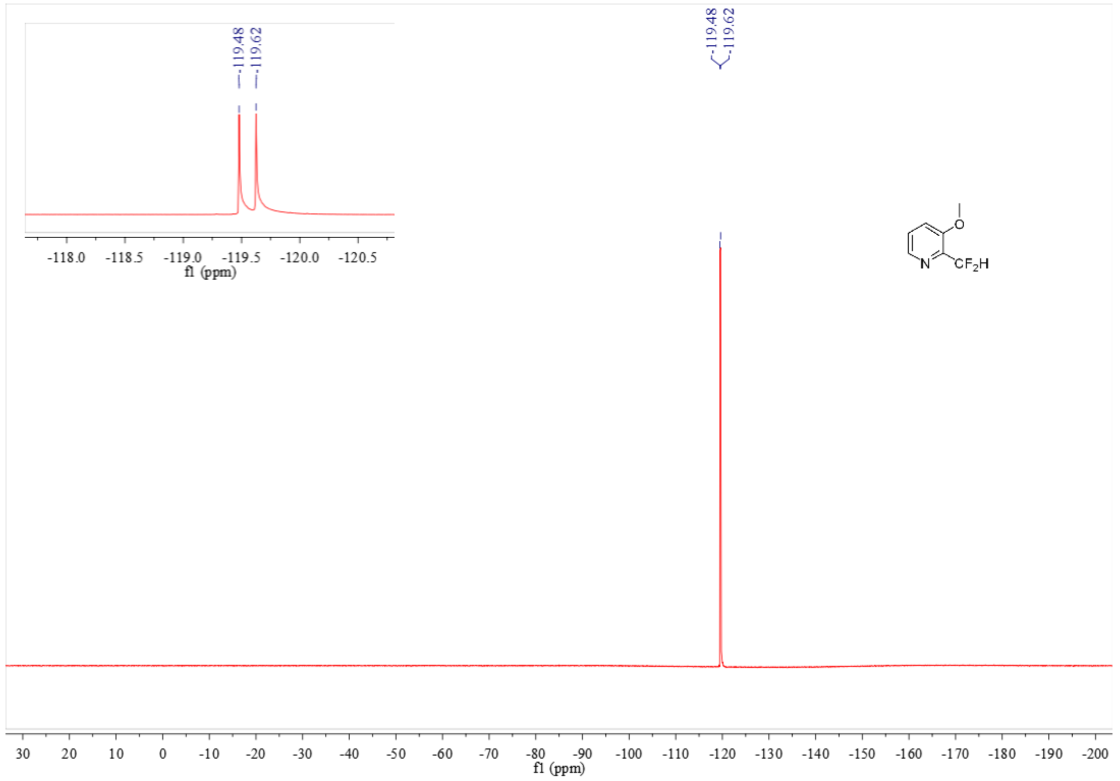

^{13}C NMR (101 MHz, $CDCl_3$) 2-(difluoromethyl)-3-methoxy-pyridine 4i

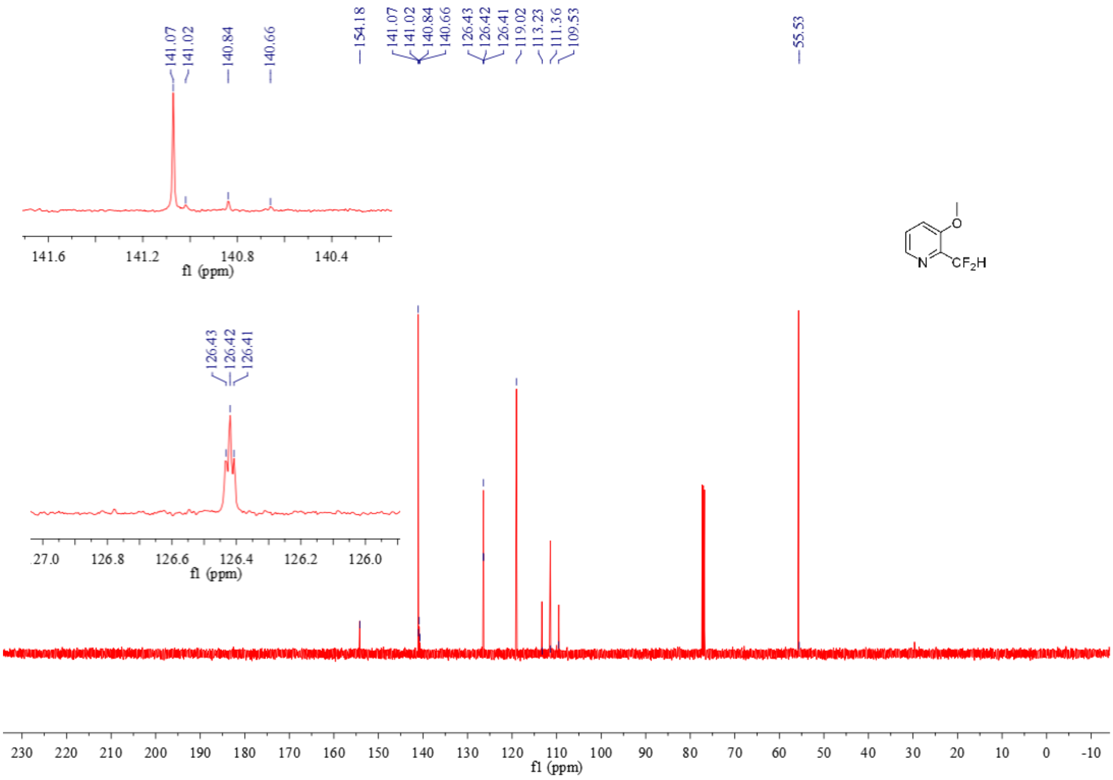

^{1}H NMR (400 MHz, $CDCl_3$) 1-[6-(difluoromethyl)-2-pyridyl]ethanone 4j

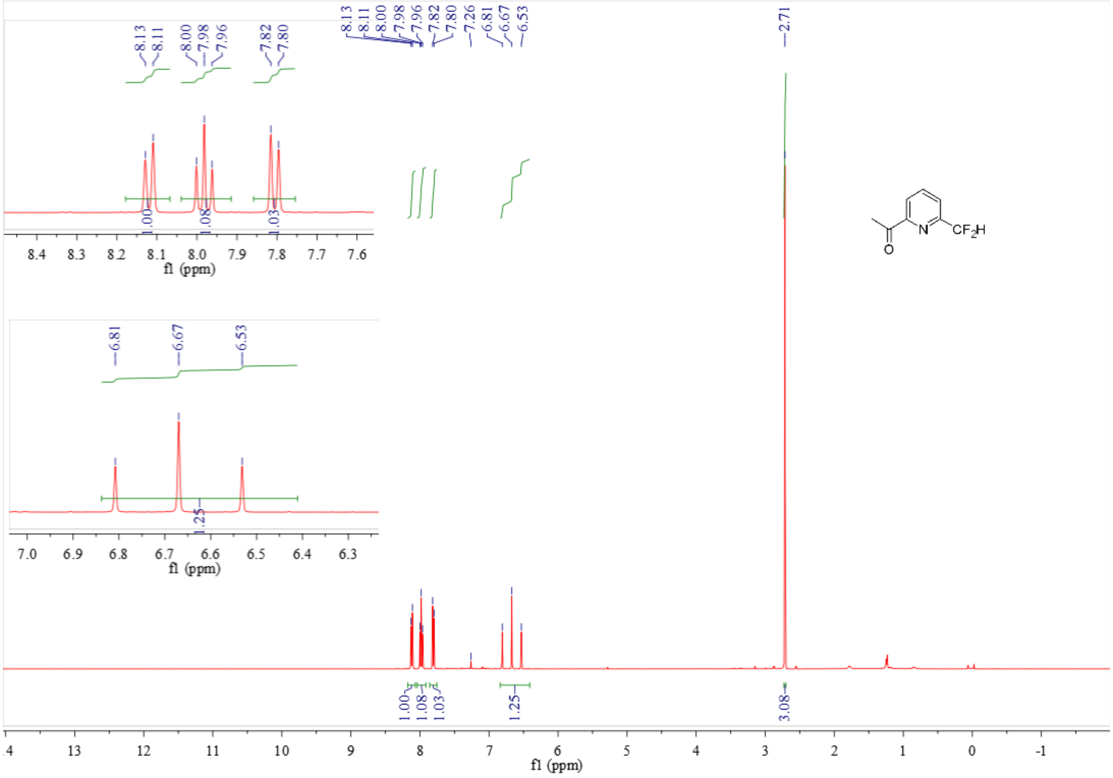

^{19}F NMR (376 MHz, $CDCl_3$) 1-[6-(difluoromethyl)-2-pyridyl]ethanone 4j

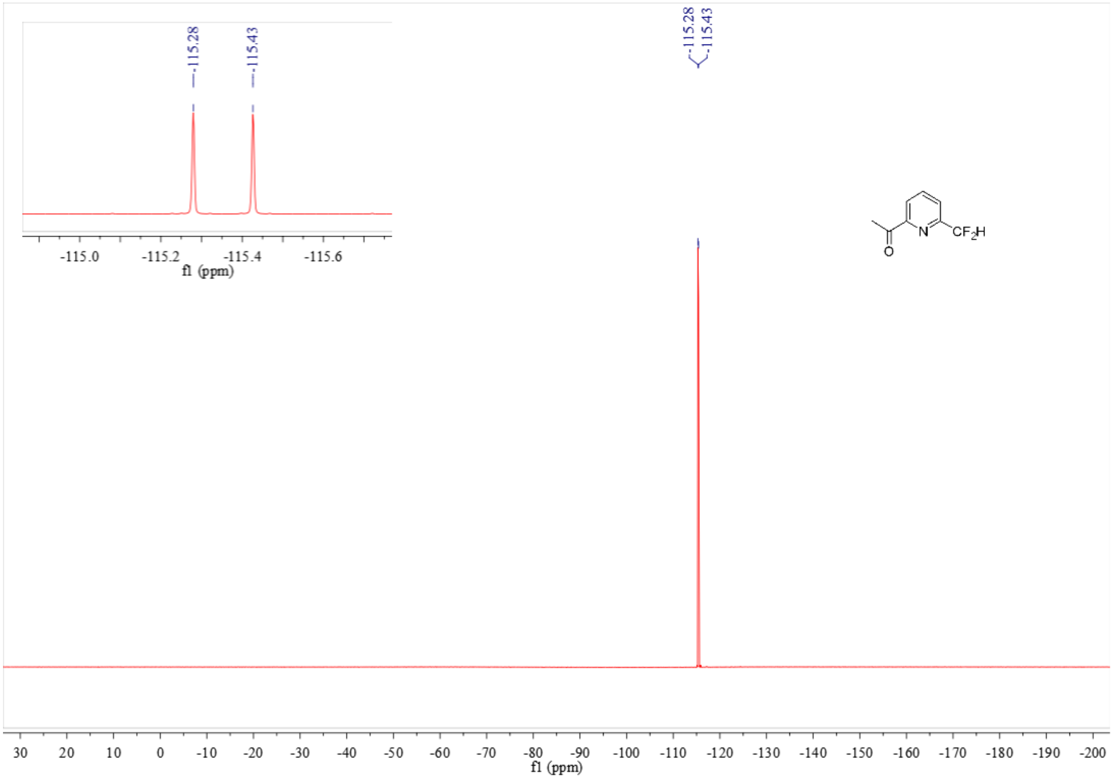

^{13}C NMR (101 MHz, $CDCl_3$) 1-[6-(difluoromethyl)-2-pyridyl]ethanone 4j

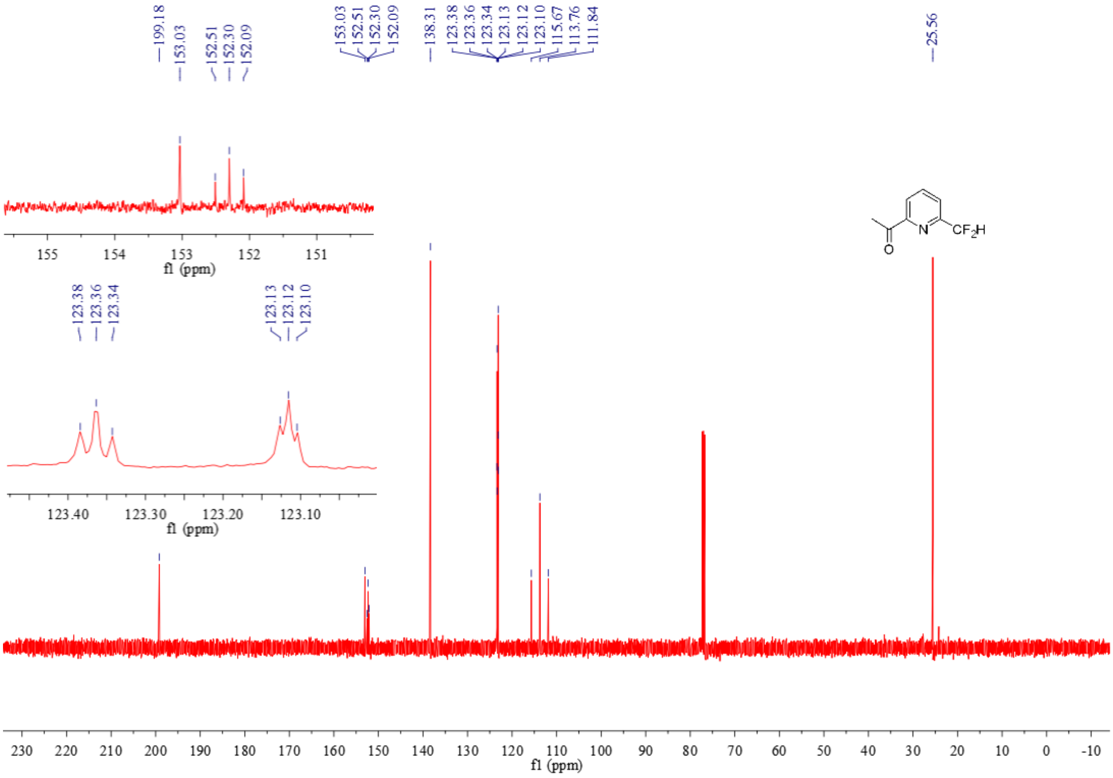

^{1}H NMR (400 MHz, $CDCl_3$) *tert*-butyl *N*-benzyl-*N*-[6-(difluoromethyl) 2-pyridyl]carbamate 4k

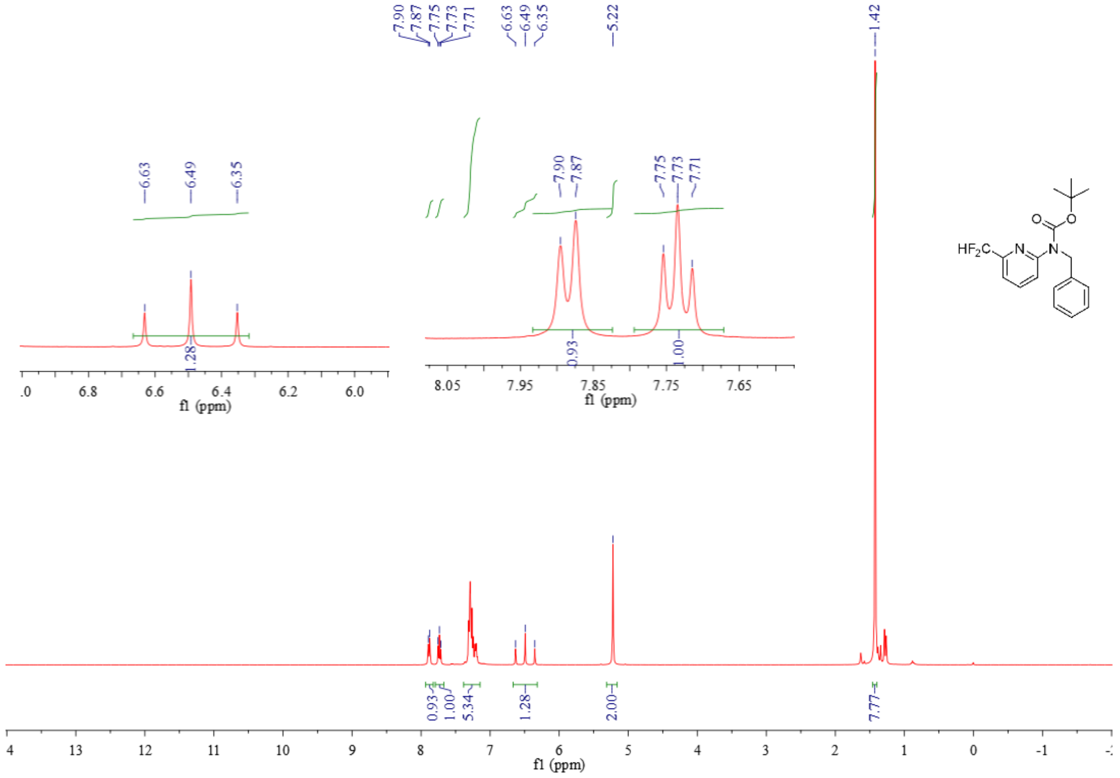

^{19}F NMR (376 MHz, $CDCl_3$) *tert*-butyl *N*-benzyl-*N*-[6-(difluoromethyl) 2-pyridyl]carbamate 4k

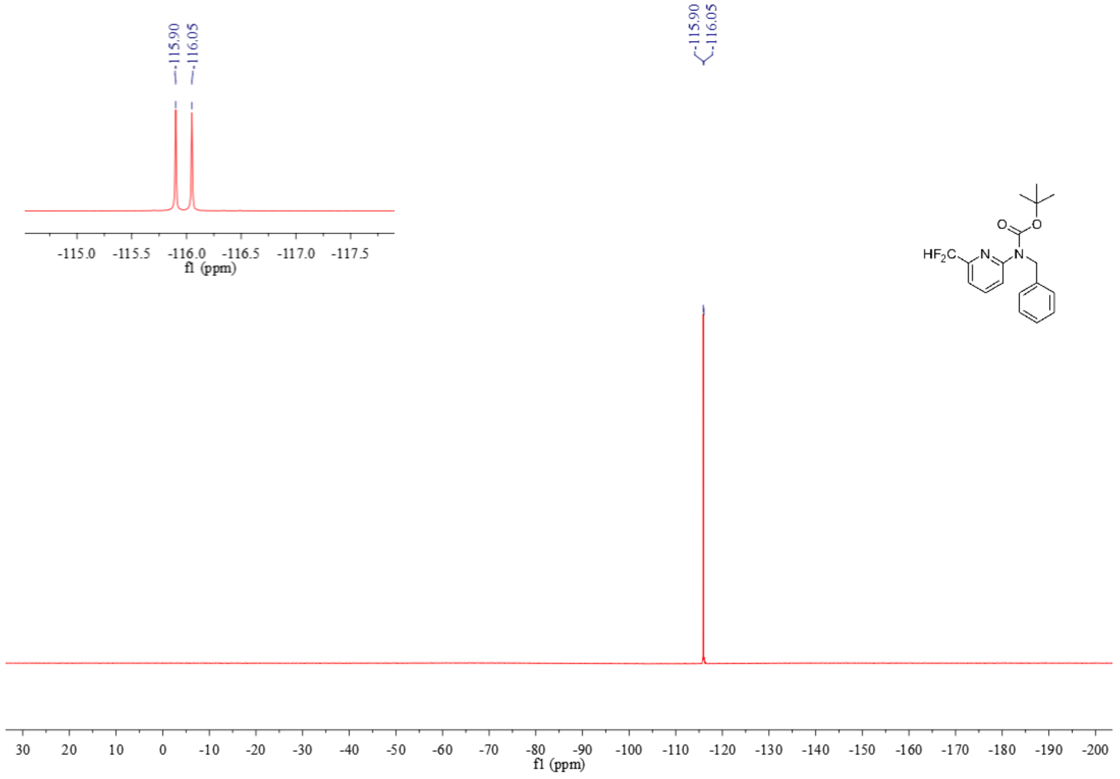

^{13}C NMR (101 MHz, $CDCl_3$) *tert*-butyl *N*-benzyl-*N*-[6-(difluoromethyl) 2-pyridyl]carbamate 4k

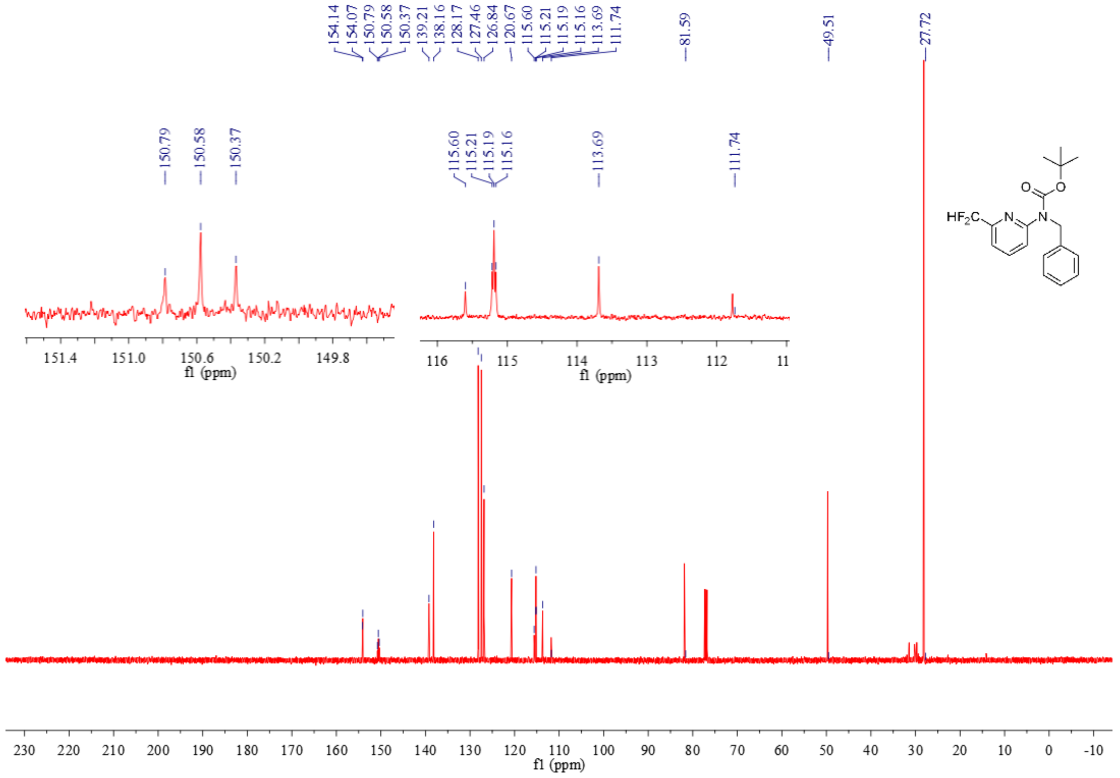

^{1}H NMR (400 MHz, $CDCl_3$) 5-(difluoromethyl)pyrimidine-2-carbonitrile 4l

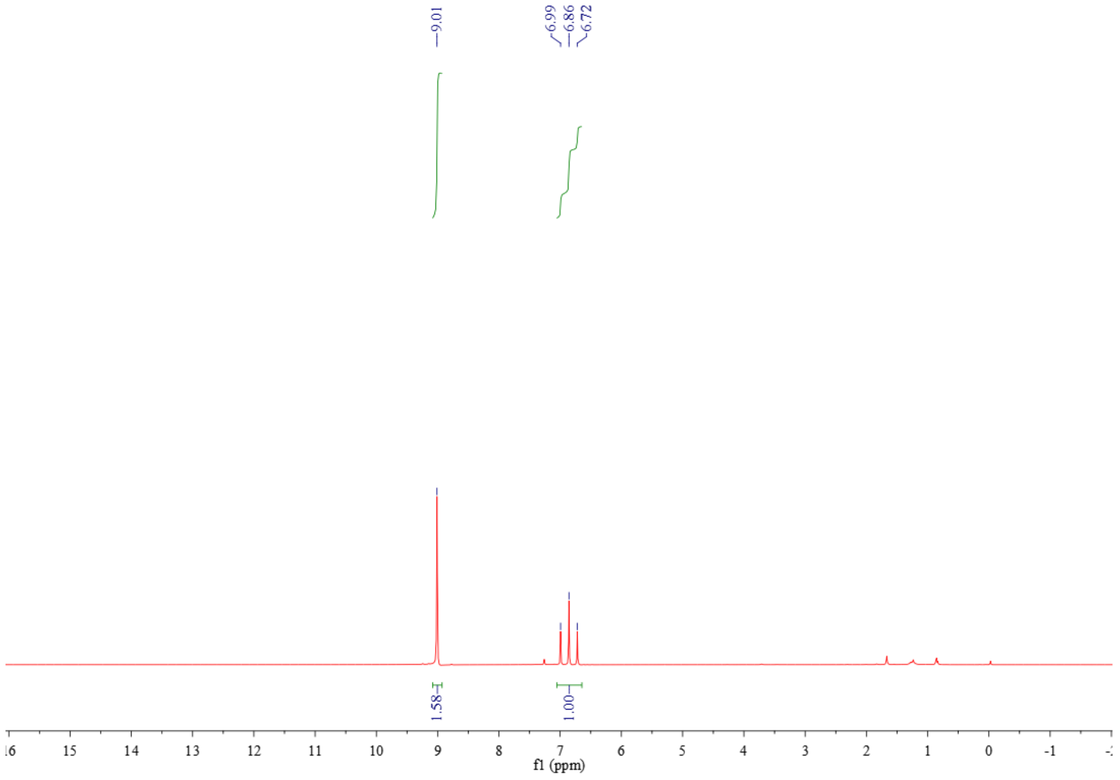

^{19}F NMR (376 MHz, $CDCl_3$) 5-(difluoromethyl)pyrimidine-2-carbonitrile 4l

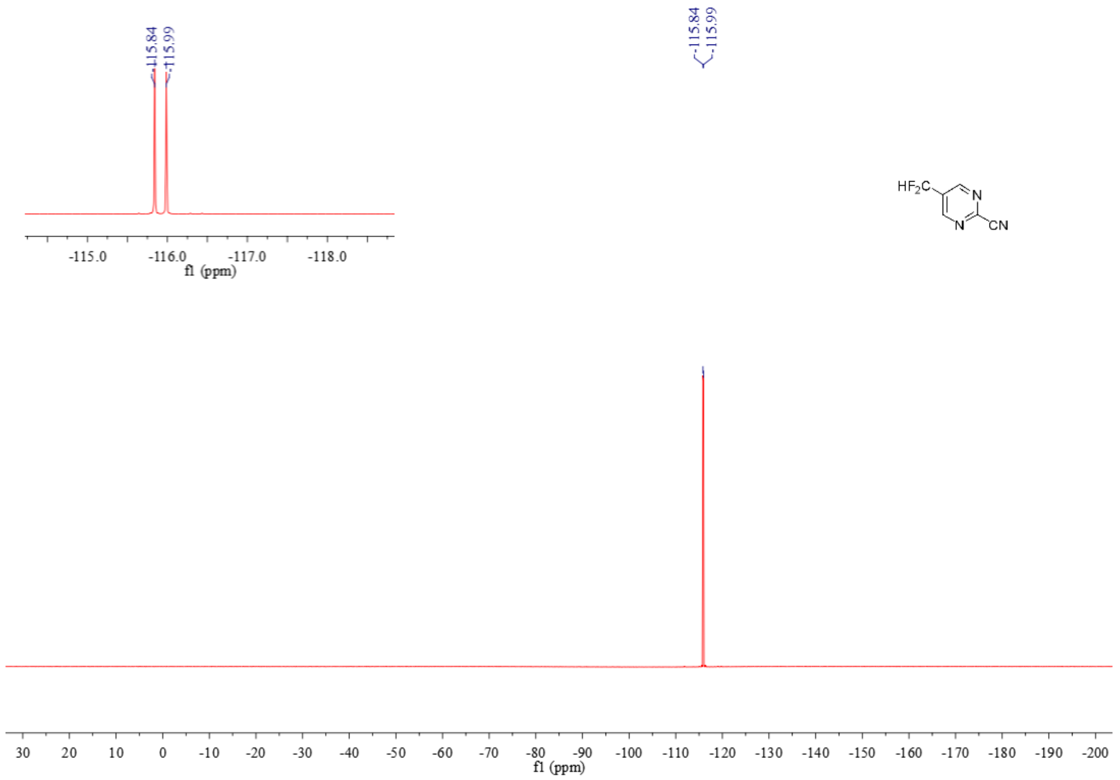

^{13}C NMR (101 MHz, $CDCl_3$) 5-(difluoromethyl)pyrimidine-2-carbonitrile 4l

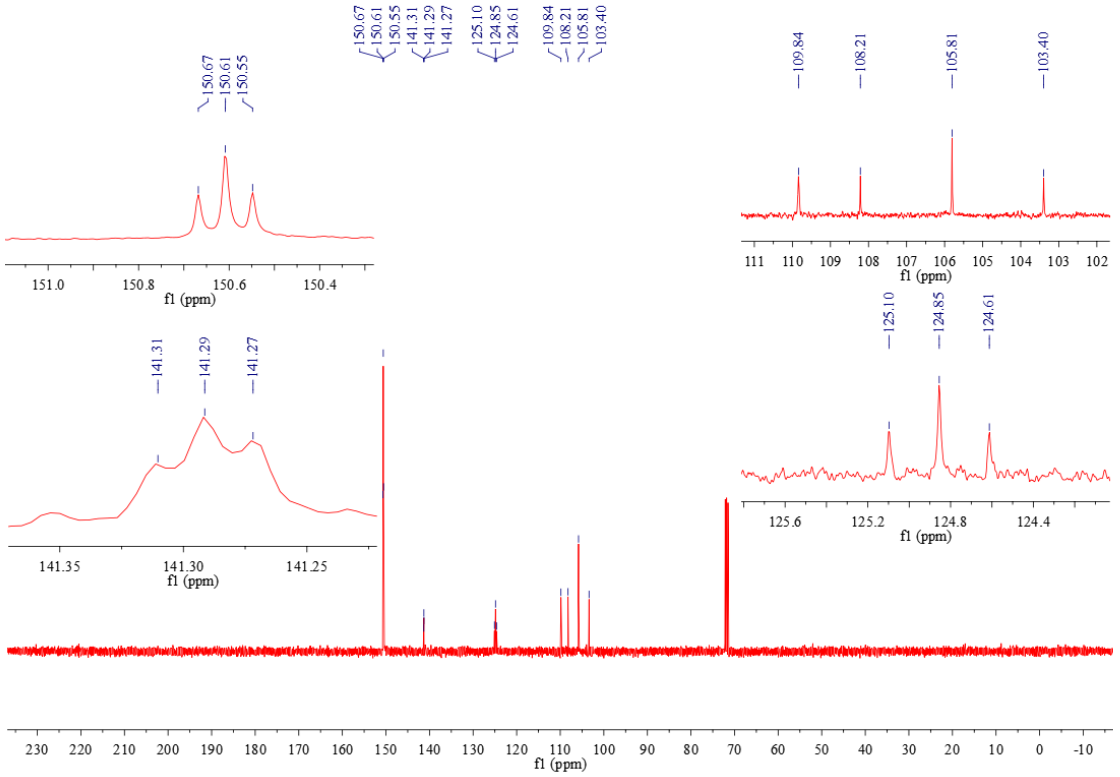

^{1}H NMR (400 MHz, $CDCl_3$) *tert*-butyl-*N*-*tert*-butoxycarbonyl *N*-[5-(difluoromethyl)pyrimidin-2-yl]carbamate 4m

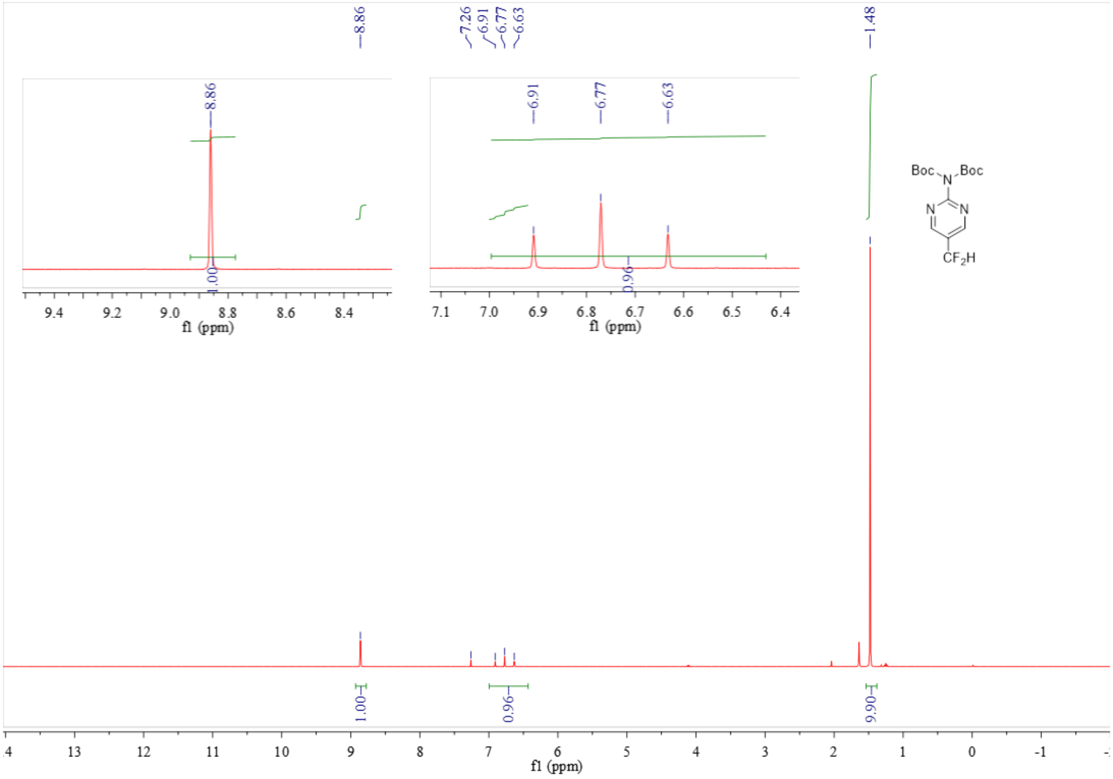

^{19}F NMR (376 MHz, $CDCl_3$) *tert*-butyl-*N*-*tert*-butoxycarbonyl *N*-[5-(difluoromethyl)pyrimidin-2-yl]carbamate 4m

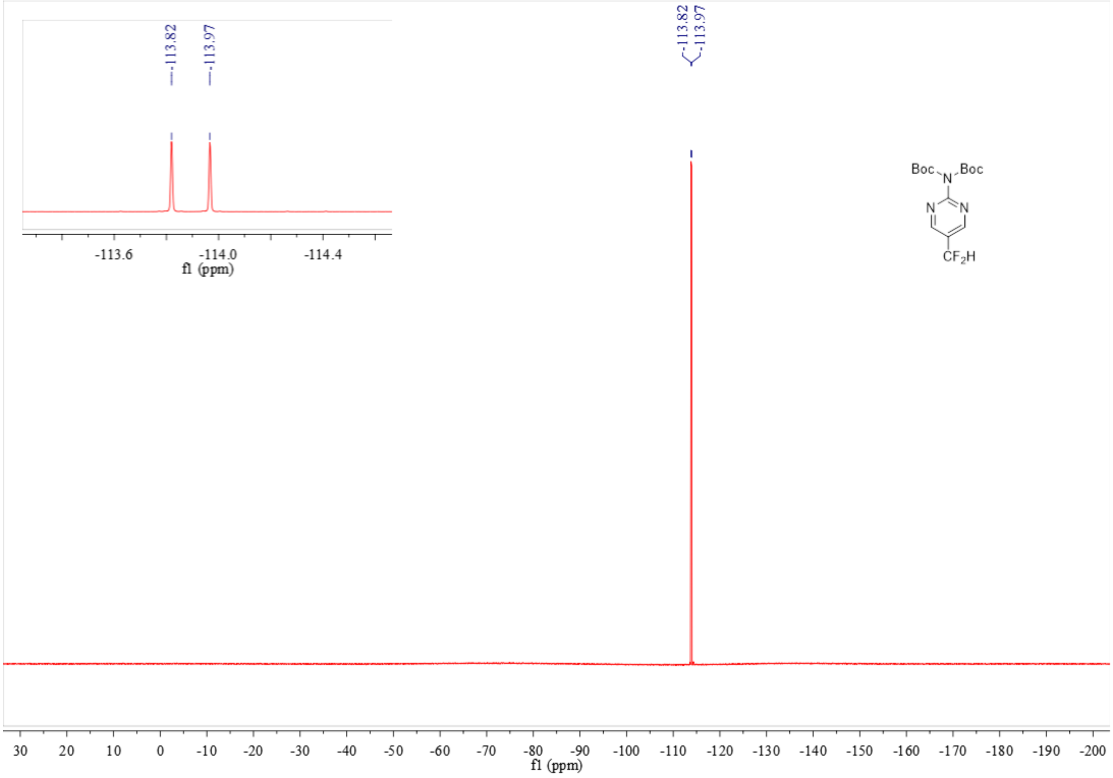

^{13}C NMR (101 MHz, $CDCl_3$) *tert*-butyl-*N*-*tert*-butoxycarbonyl *N*-[5-(difluoromethyl)pyrimidin-2-yl]carbamate 4m

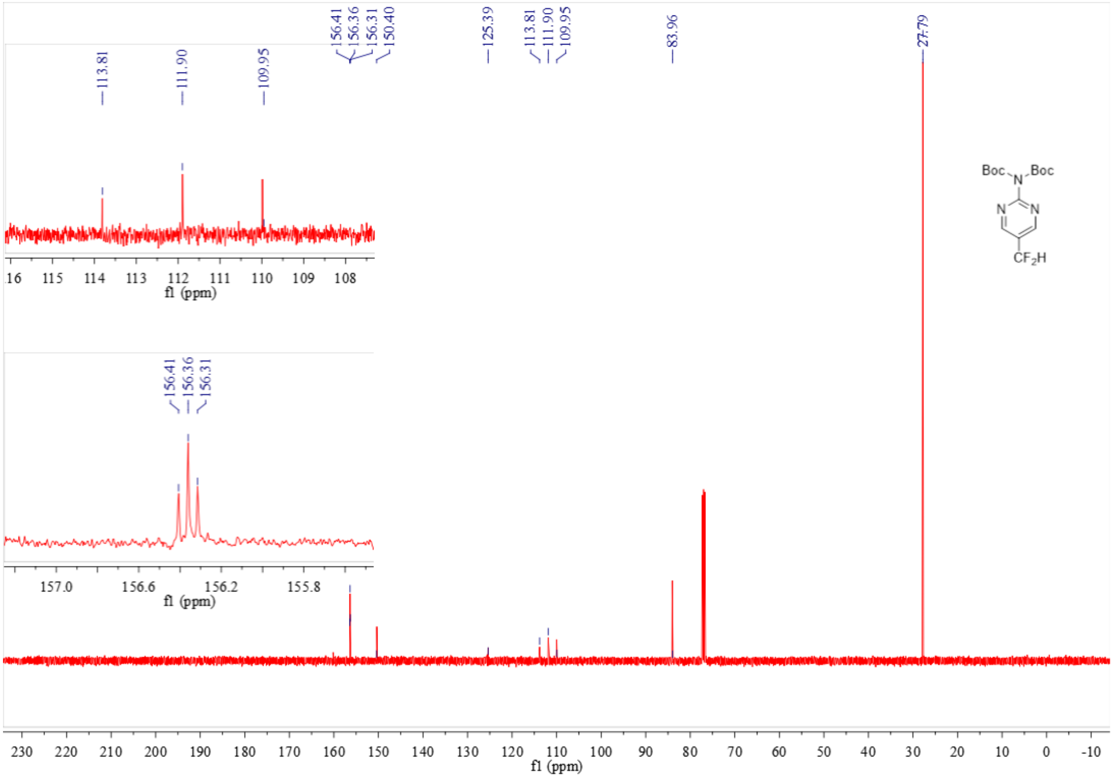

^{1}H NMR (400 MHz, $CDCl_3$) 5-(difluoromethyl)-2-methoxy-pyrimidine 4n

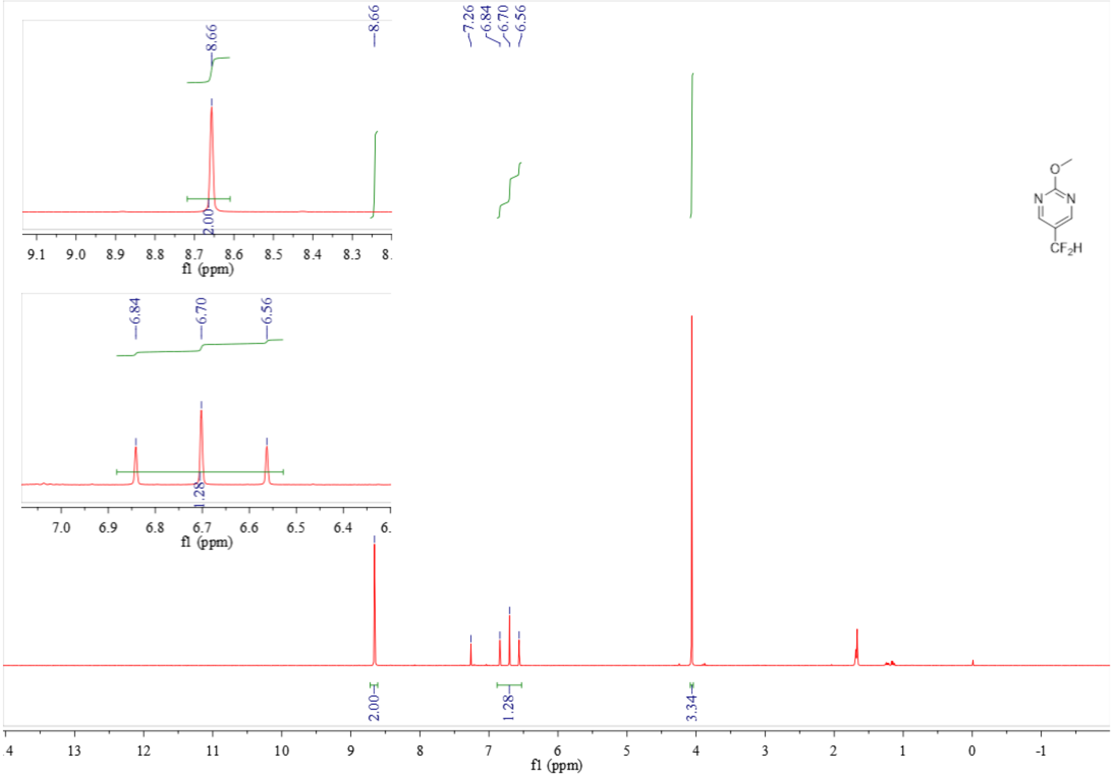

^{19}F NMR (376 MHz, $CDCl_3$) 5-(difluoromethyl)-2-methoxy-pyrimidine 4n

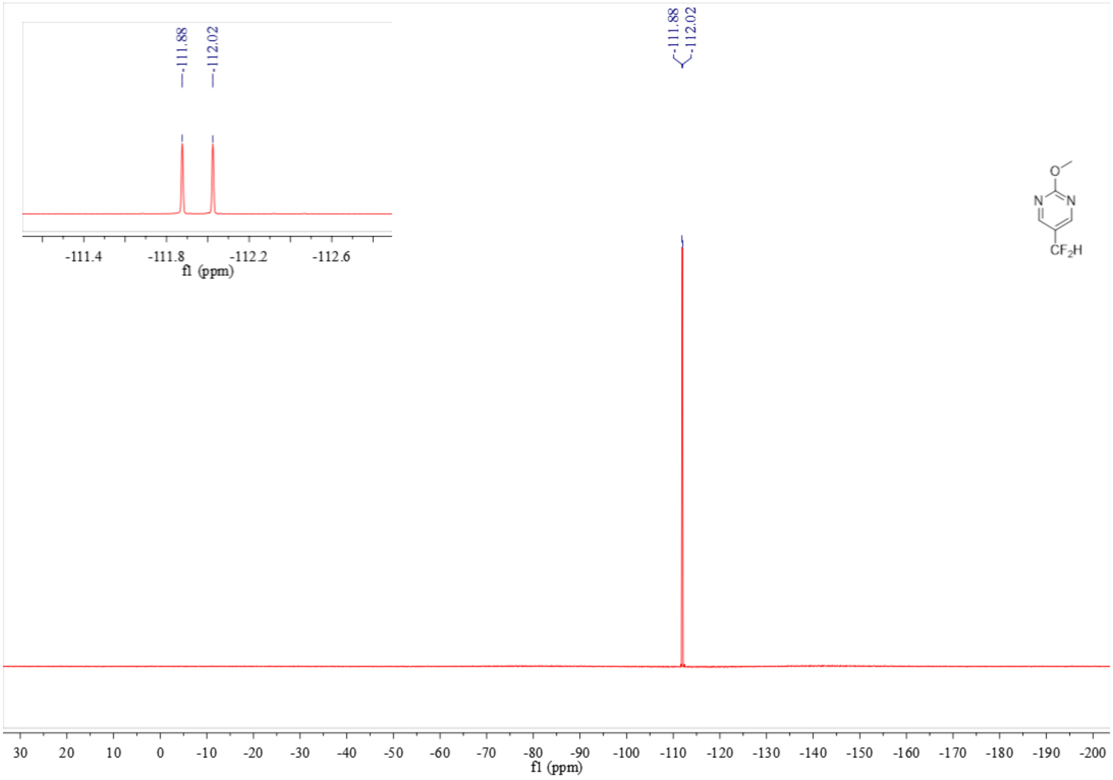

^{13}C NMR (101 MHz, $CDCl_3$) 5-(difluoromethyl)-2-methoxy-pyrimidine 4n

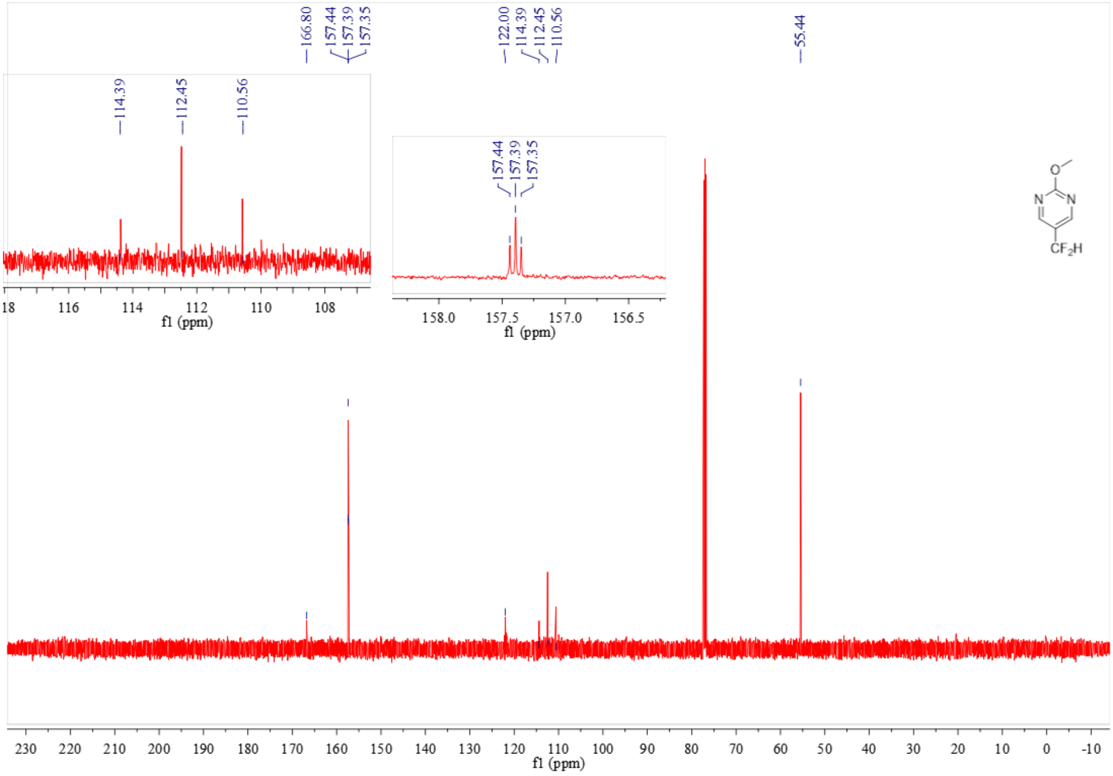

^{1}H NMR (400 MHz, $CDCl_3$) 5-(difluoromethyl)-2-pyrrolidin-1-yl-pyrimidine 4o

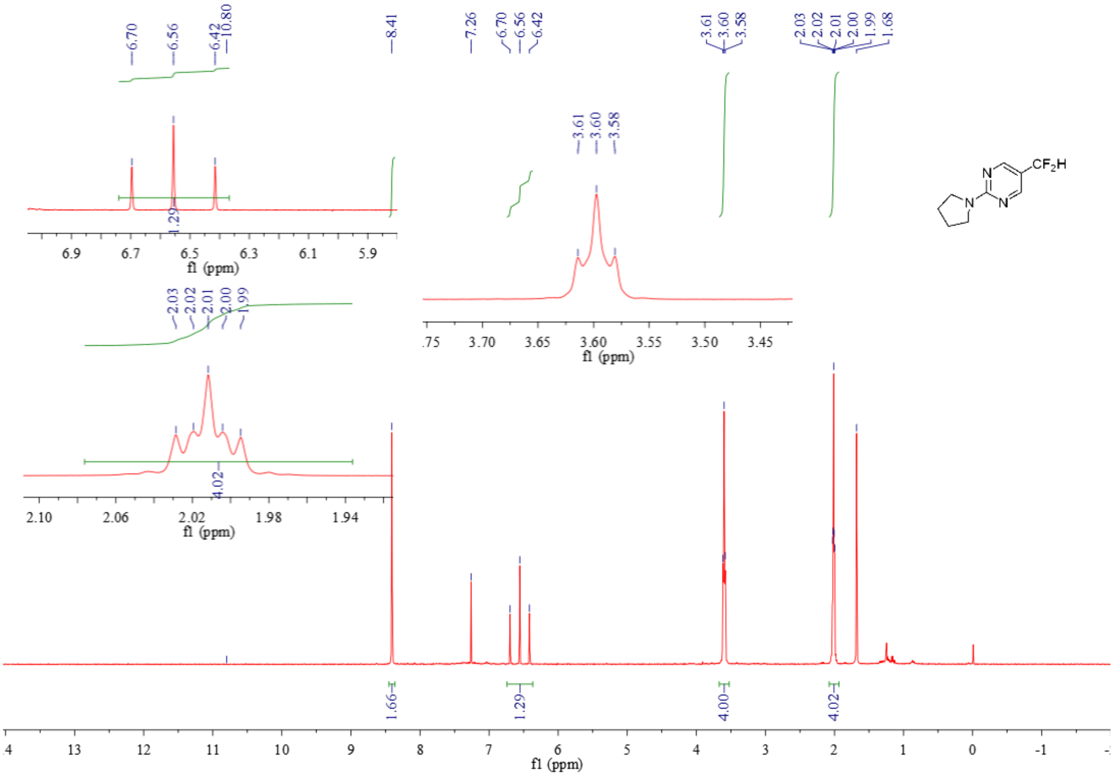

^{19}F NMR (376 MHz, $CDCl_3$) 5-(difluoromethyl)-2-pyrrolidin-1-yl-pyrimidine 4o

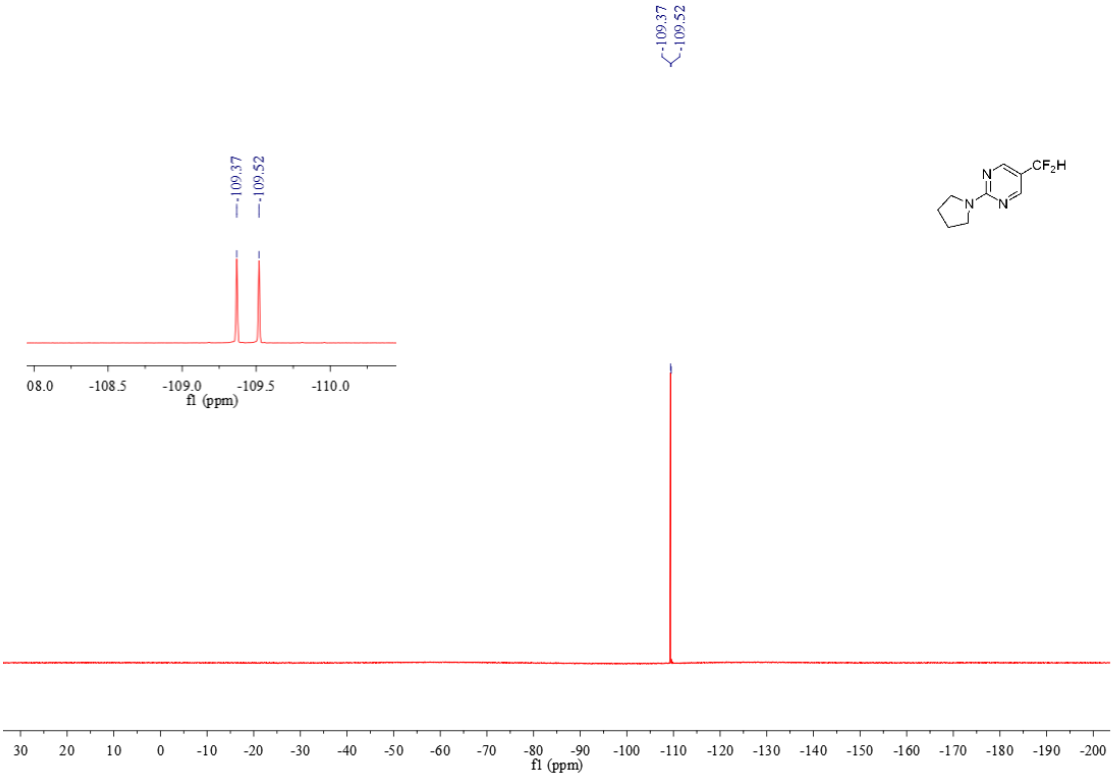

^{13}C NMR (101 MHz, $CDCl_3$) 5-(difluoromethyl)-2-pyrrolidin-1-yl-pyrimidine 4o

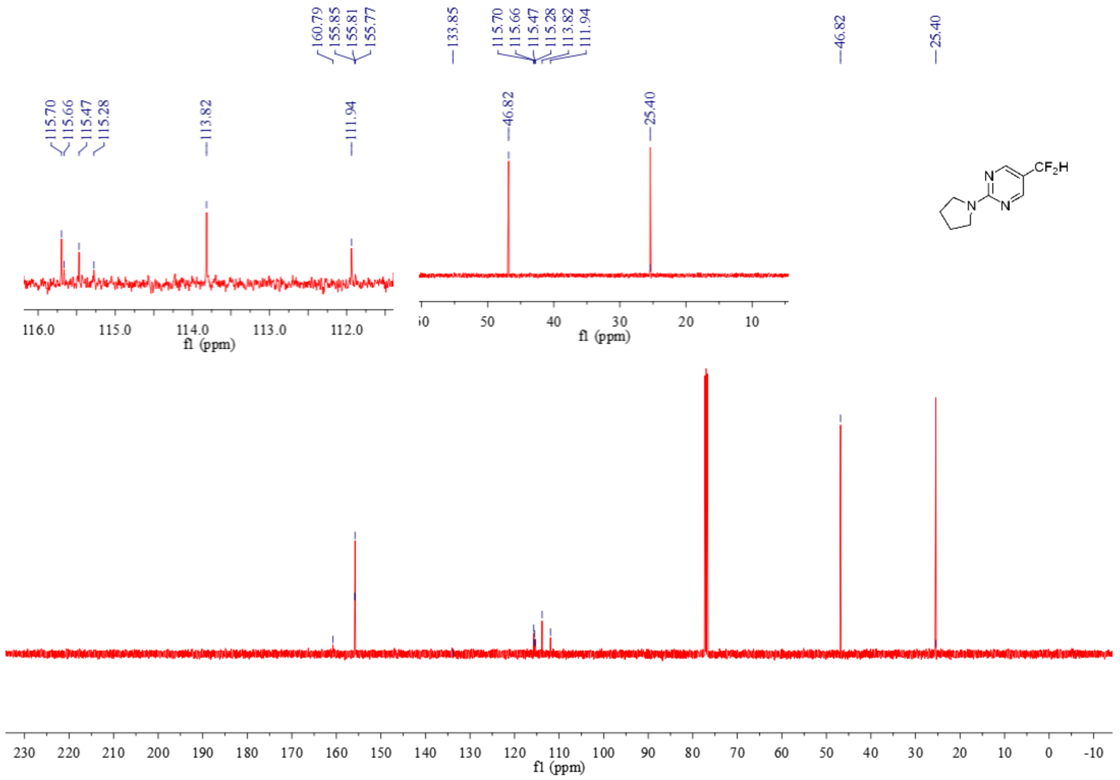

^{1}H NMR (400 MHz, $CDCl_3$) methyl 3-(difluoromethyl)pyrazine-2-carboxylate 4p

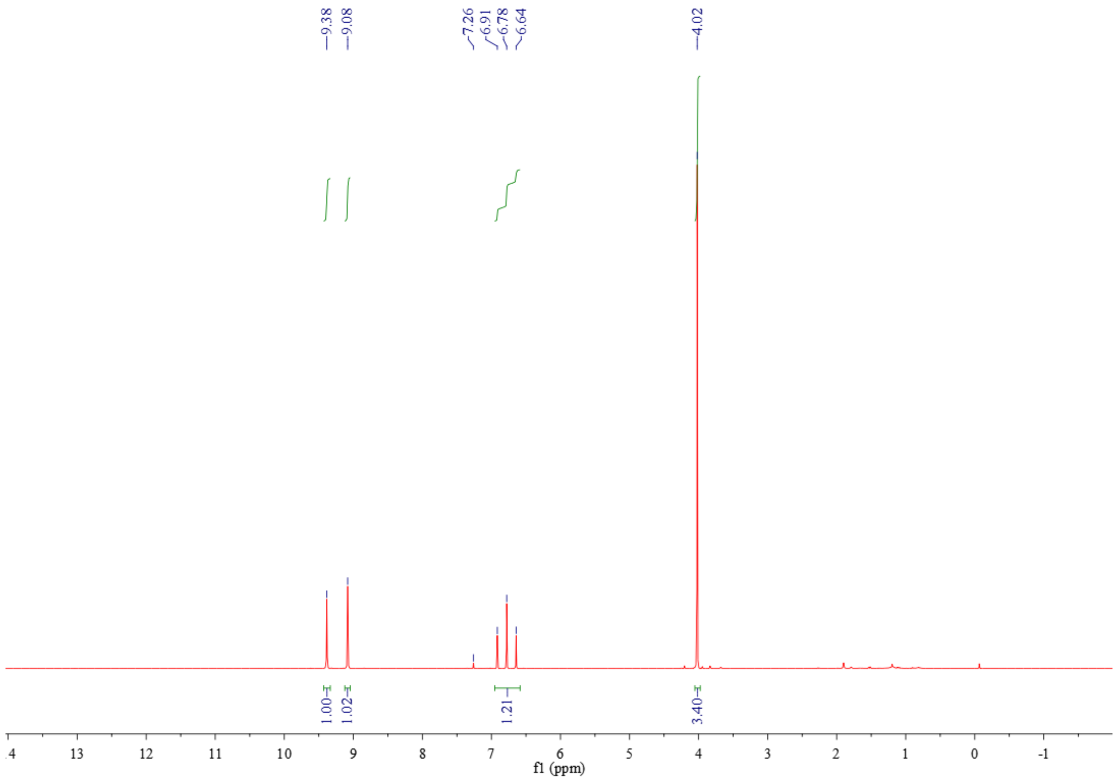

^{19}F NMR (376 MHz, $CDCl_3$) methyl 3-(difluoromethyl)pyrazine-2-carboxylate 4p

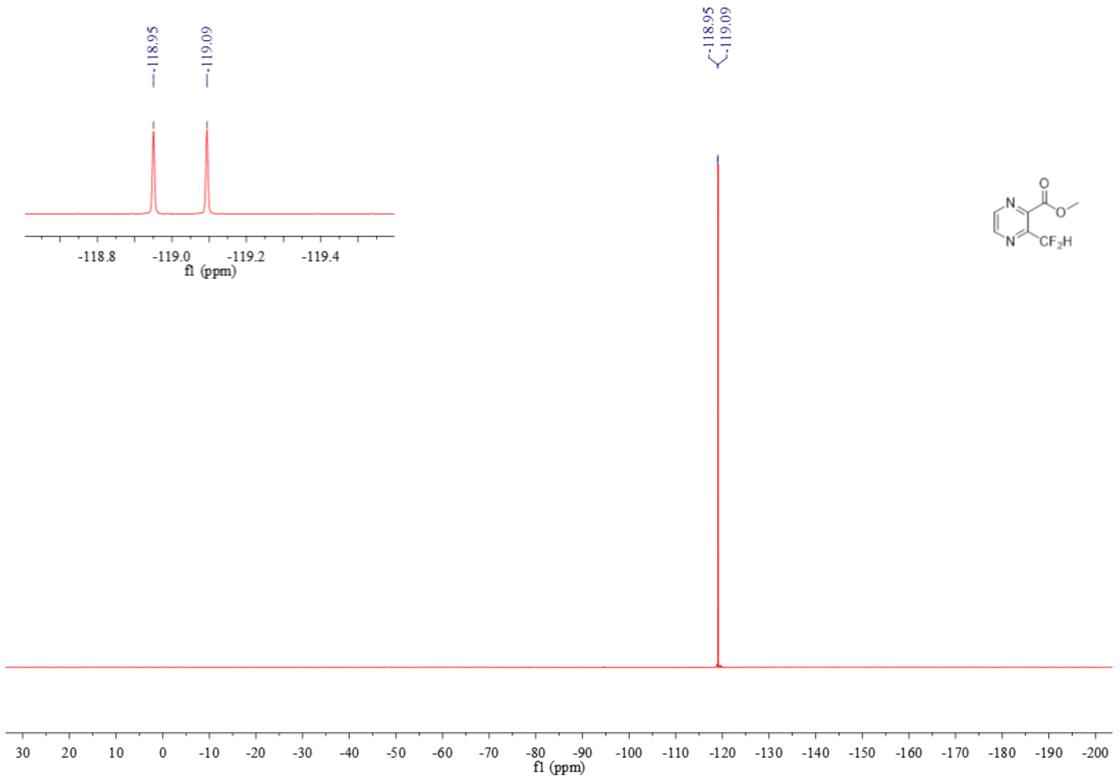

^{13}C NMR (101 MHz, $CDCl_3$) methyl 3-(difluoromethyl) pyrazine-2-carboxylate 4p

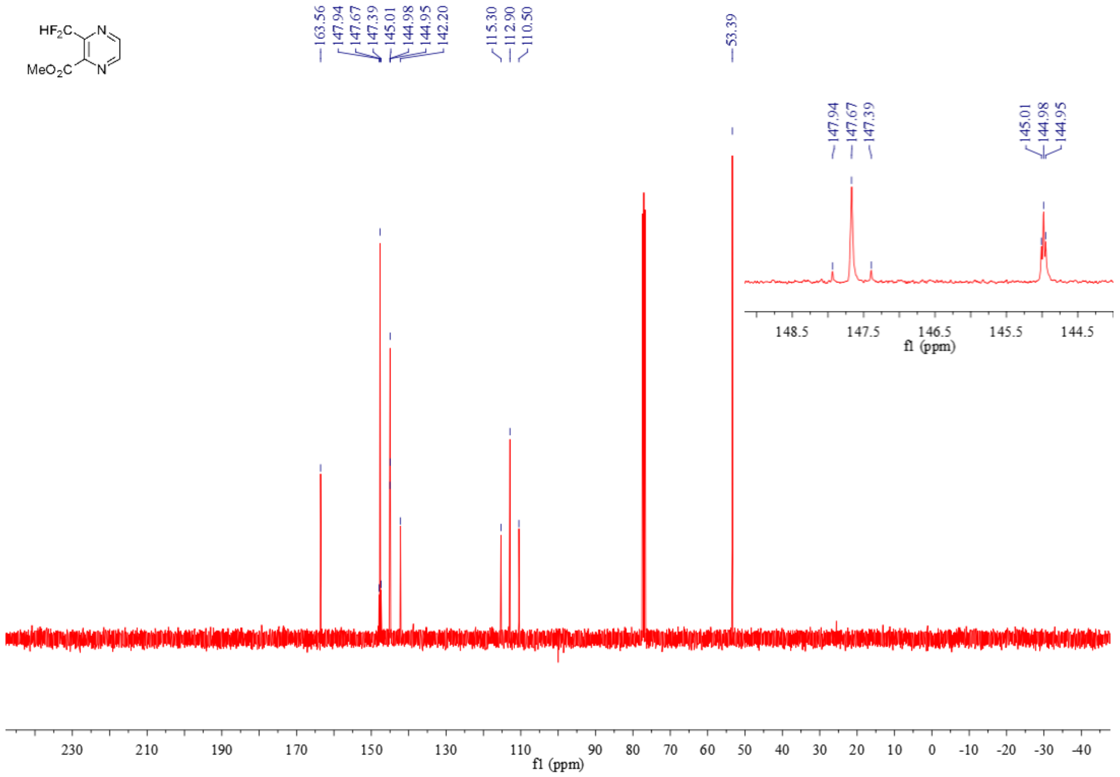

^{1}H NMR (400 MHz, $CDCl_3$) 2-(difluoromethyl)-5-methylsulfanyl-pyrazine 4q

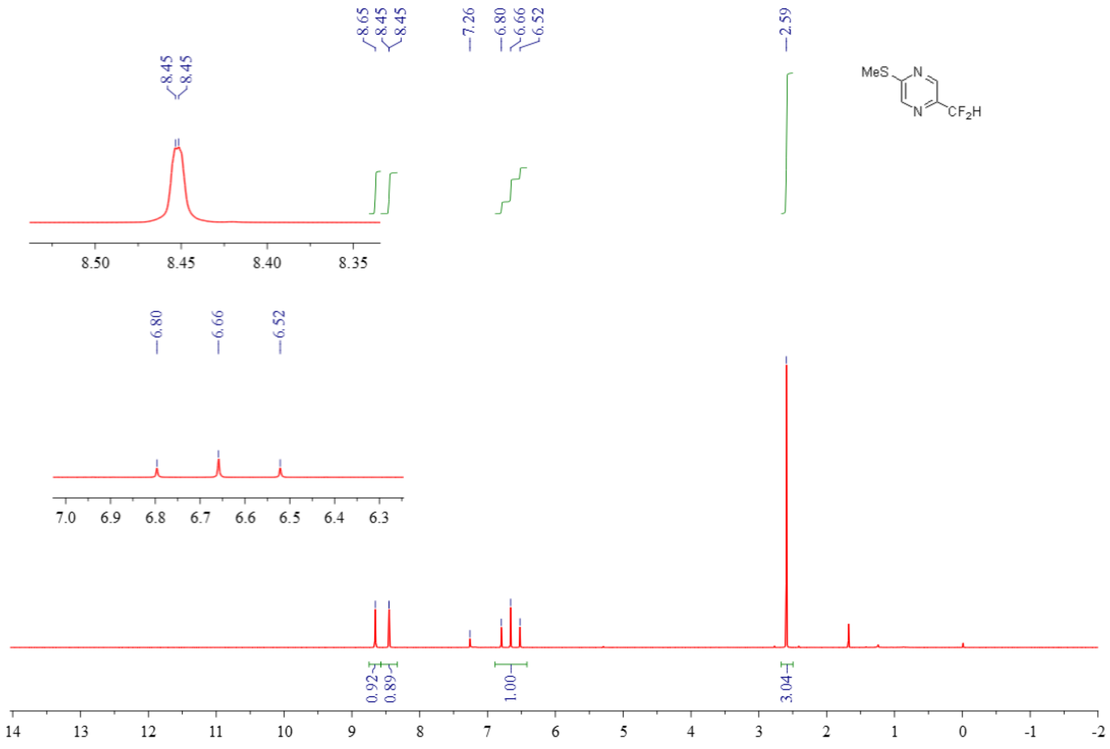

^{19}F NMR (376 MHz, $CDCl_3$) 2-(difluoromethyl)-5-methylsulfanyl-pyrazine 4q

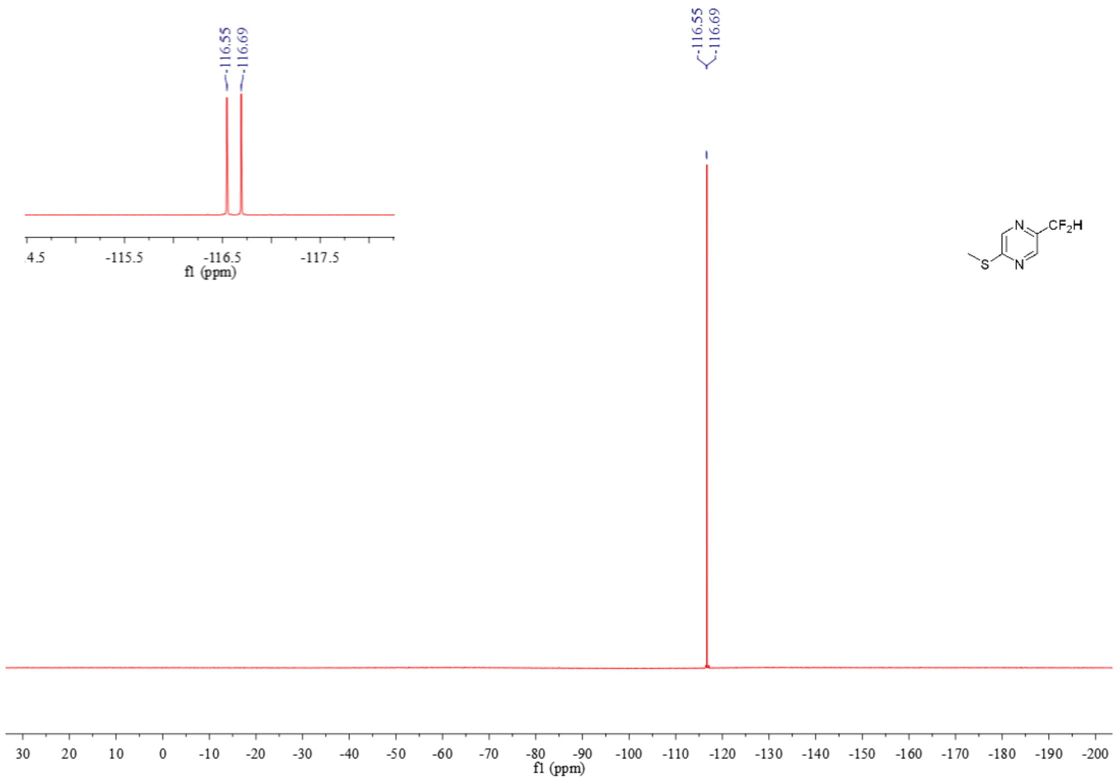

^{13}C NMR (101 MHz, $CDCl_3$) methyl 2-(difluoromethyl) -5-methylsulfanyl-pyrazine 4q

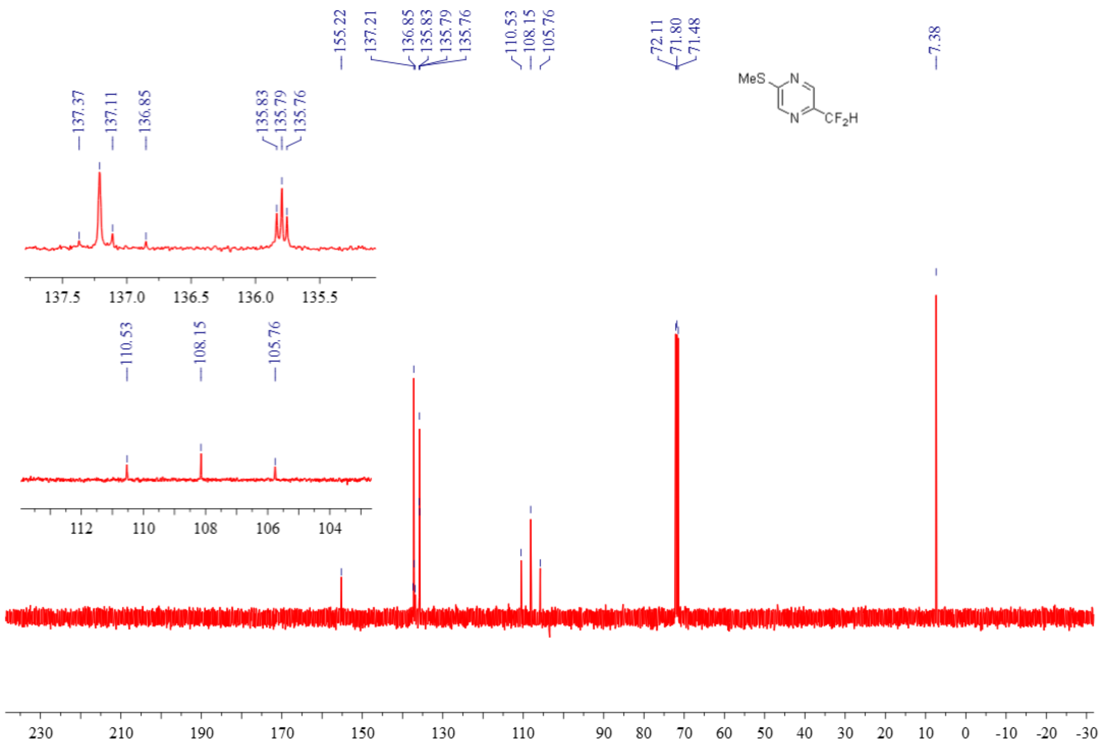

^{1}H NMR (400 MHz, $CDCl_3$) 4-(difluoromethyl)quinolone 4r

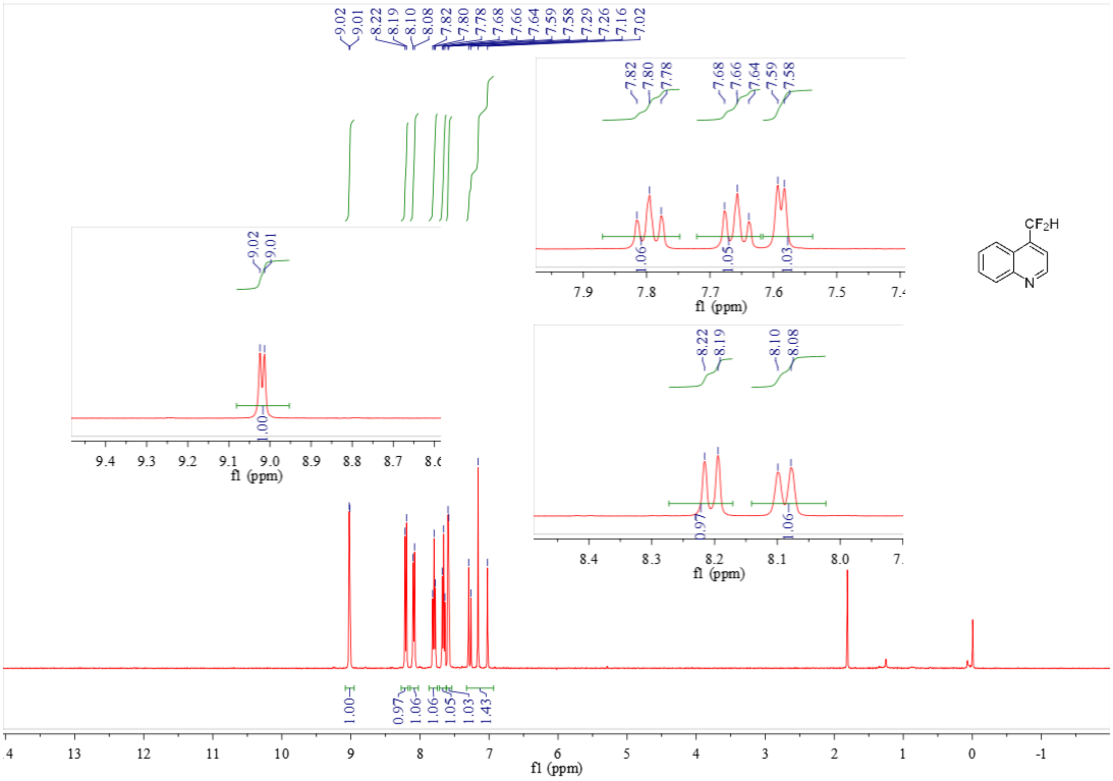

^{19}F NMR (376 MHz, $CDCl_3$) 4-(difluoromethyl)quinolone 4r

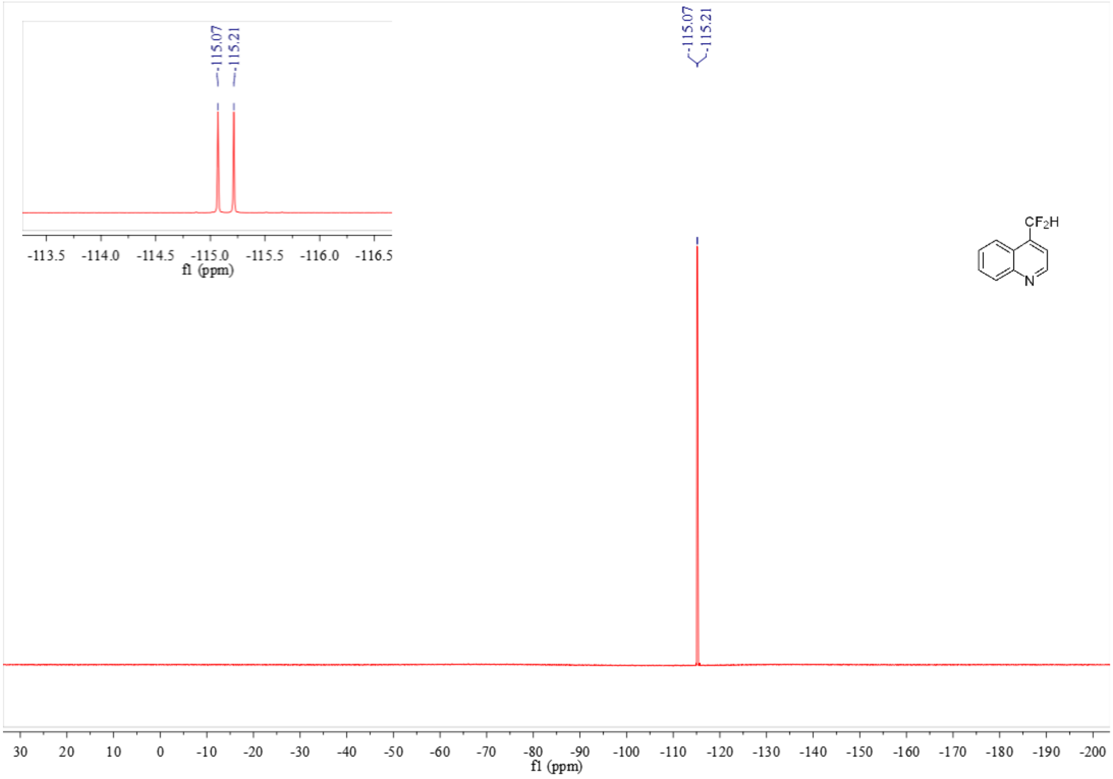

^{13}C NMR (101 MHz, $CDCl_3$) 4-(difluoromethyl)quinolone 4r

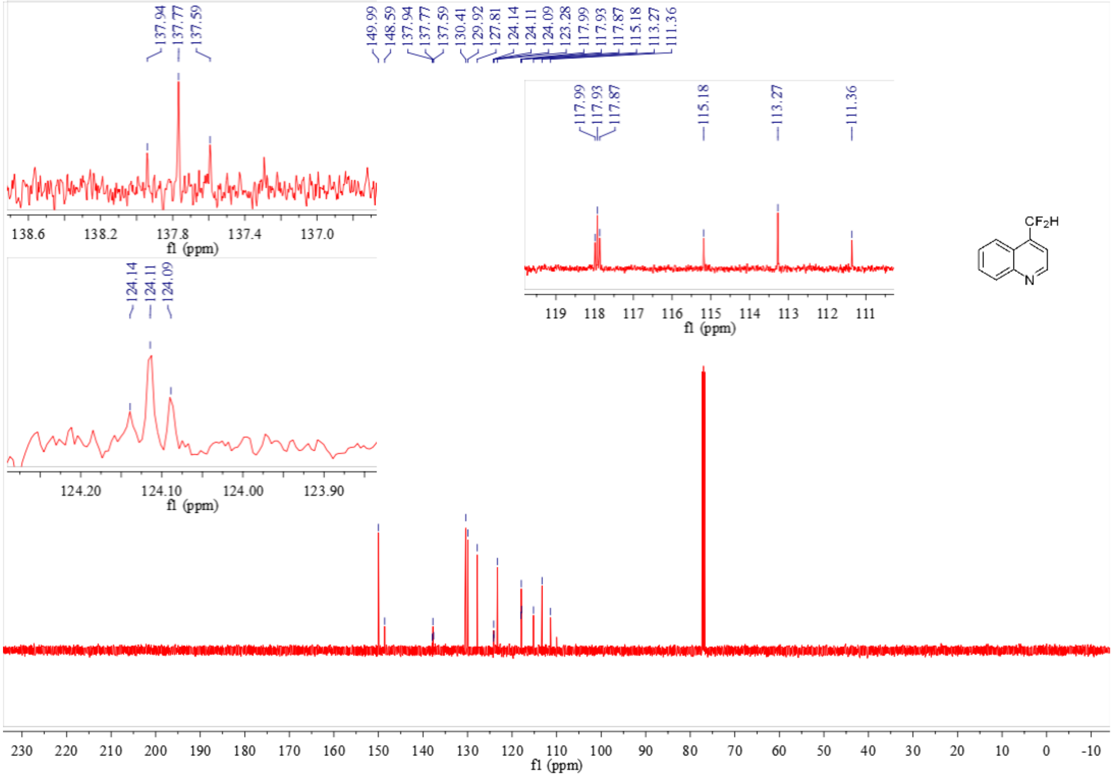

^{1}H NMR (400 MHz, $CDCl_3$) 3-(difluoromethyl)quinolone 4s

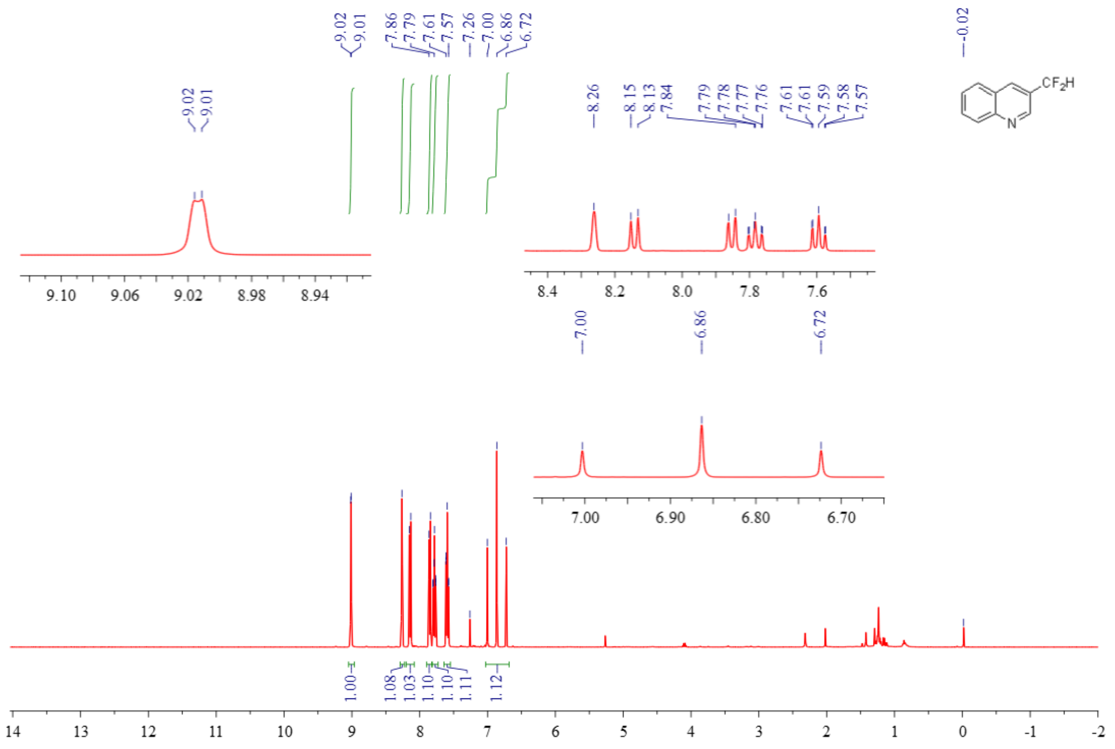

^{19}F NMR (376 MHz, $CDCl_3$) 3-(difluoromethyl)quinolone 4s

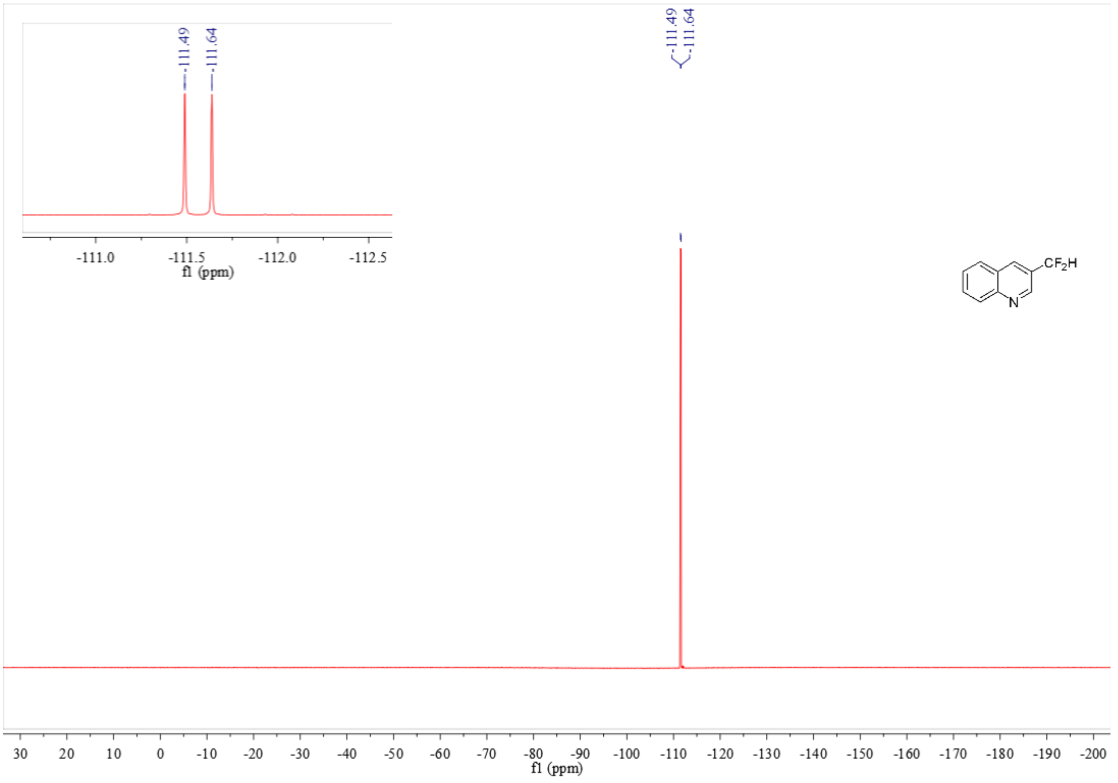

^{13}C NMR (101 MHz, $CDCl_3$) 3-(difluoromethyl)quinolone 4s

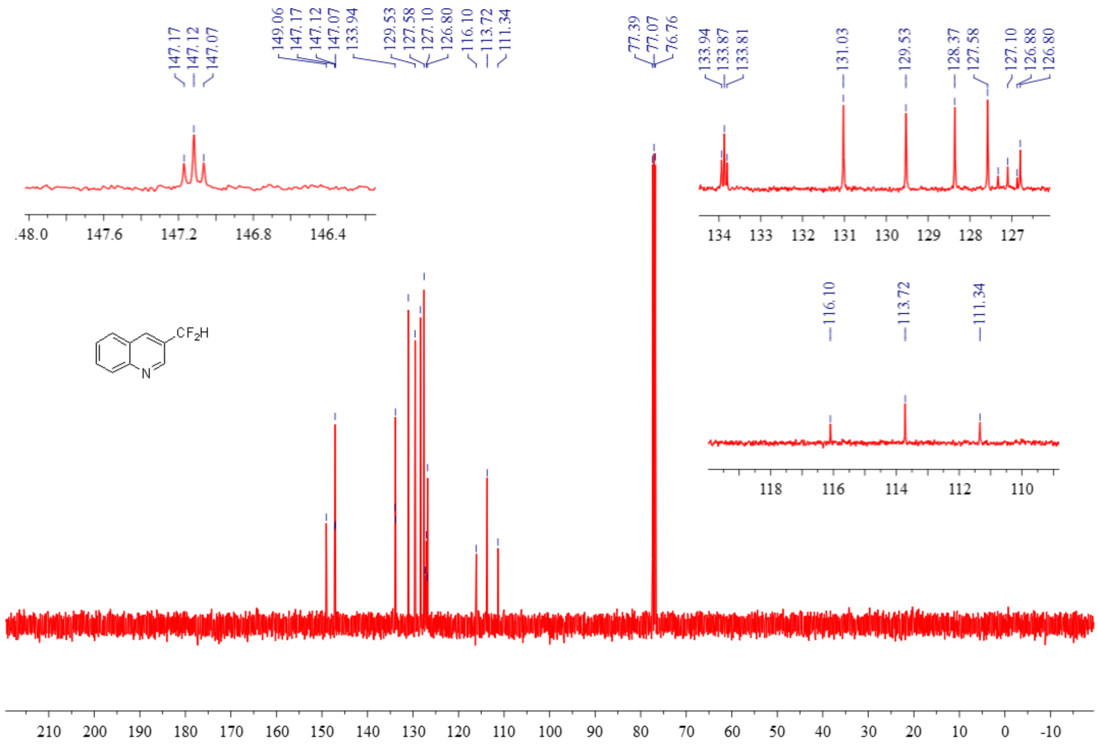

^{1}H NMR (400 MHz, $CDCl_3$) 2-(difluoromethyl)quinolone 4t

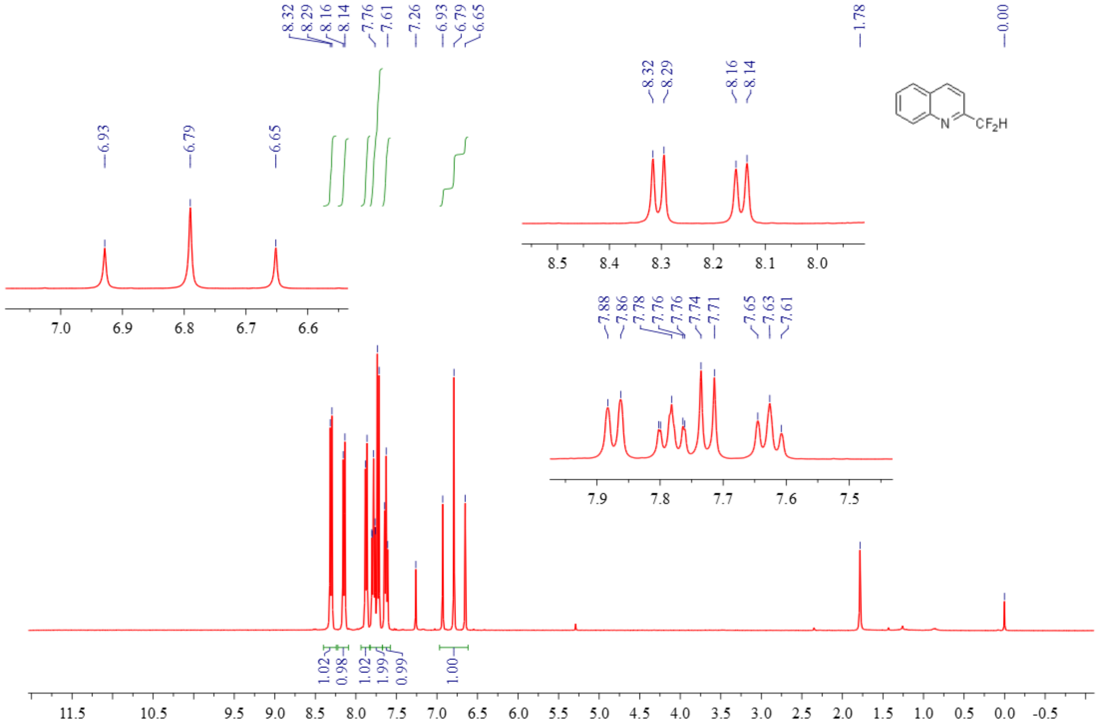

^{19}F NMR (376 MHz, $CDCl_3$) 2-(difluoromethyl)quinolone 4t

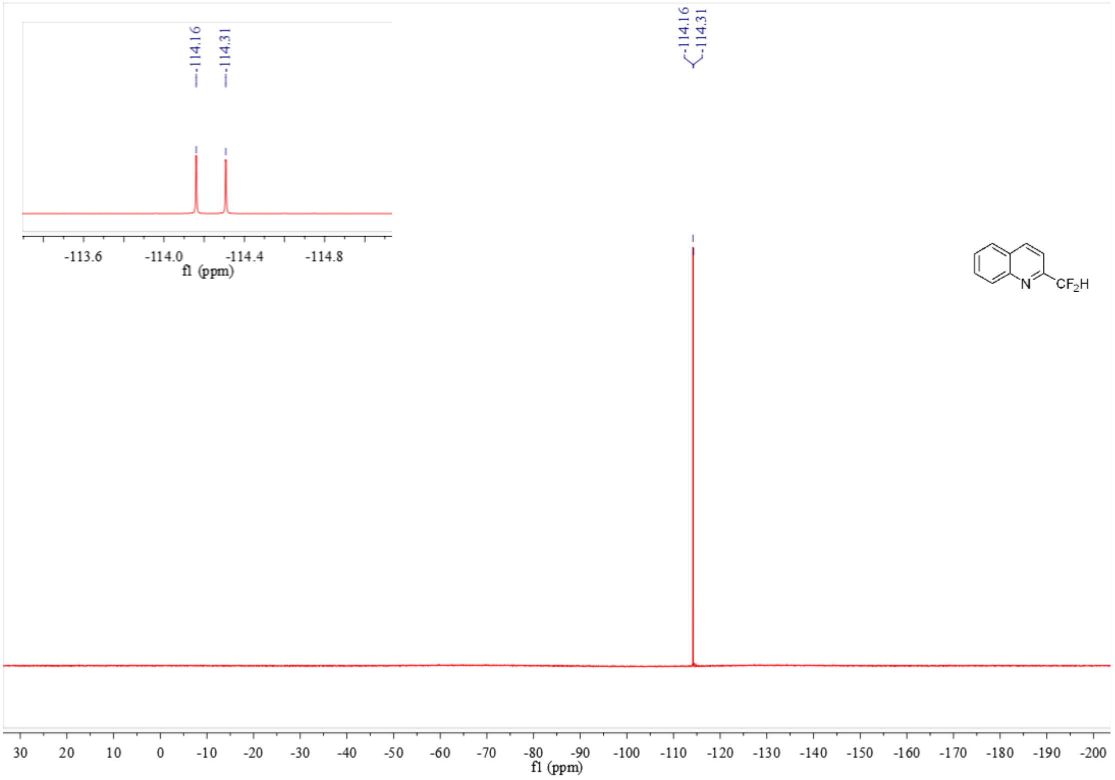

^{13}C NMR (101 MHz, $CDCl_3$) 2-(difluoromethyl)quinolone 4t

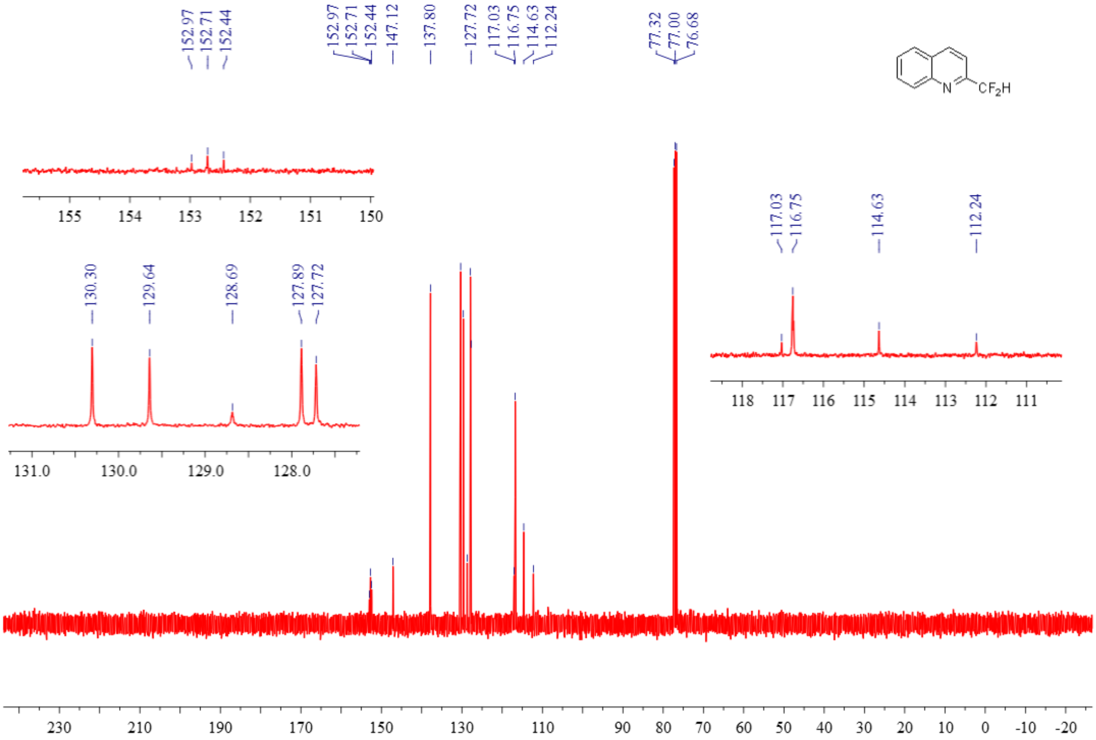

^{1}H NMR (400 MHz, $CDCl_3$) 4-(difluoromethyl)isoquinoline 4u

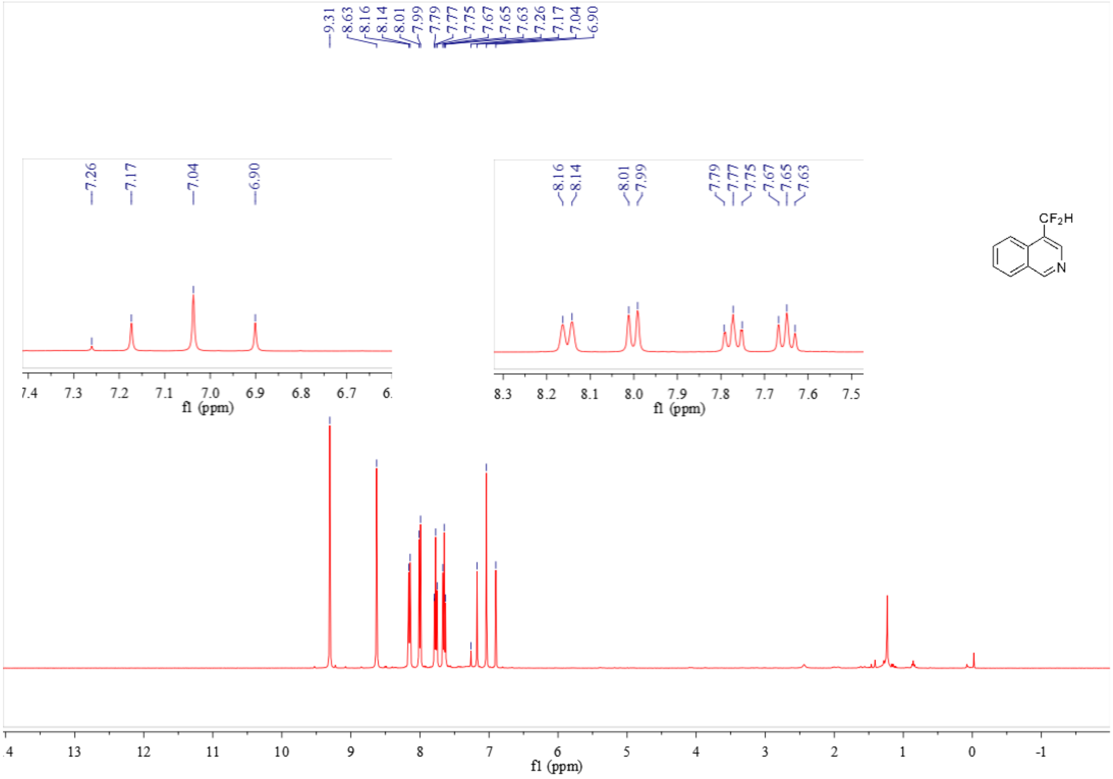

^{19}F NMR (376 MHz, $CDCl_3$) 4-(difluoromethyl)isoquinoline 4u

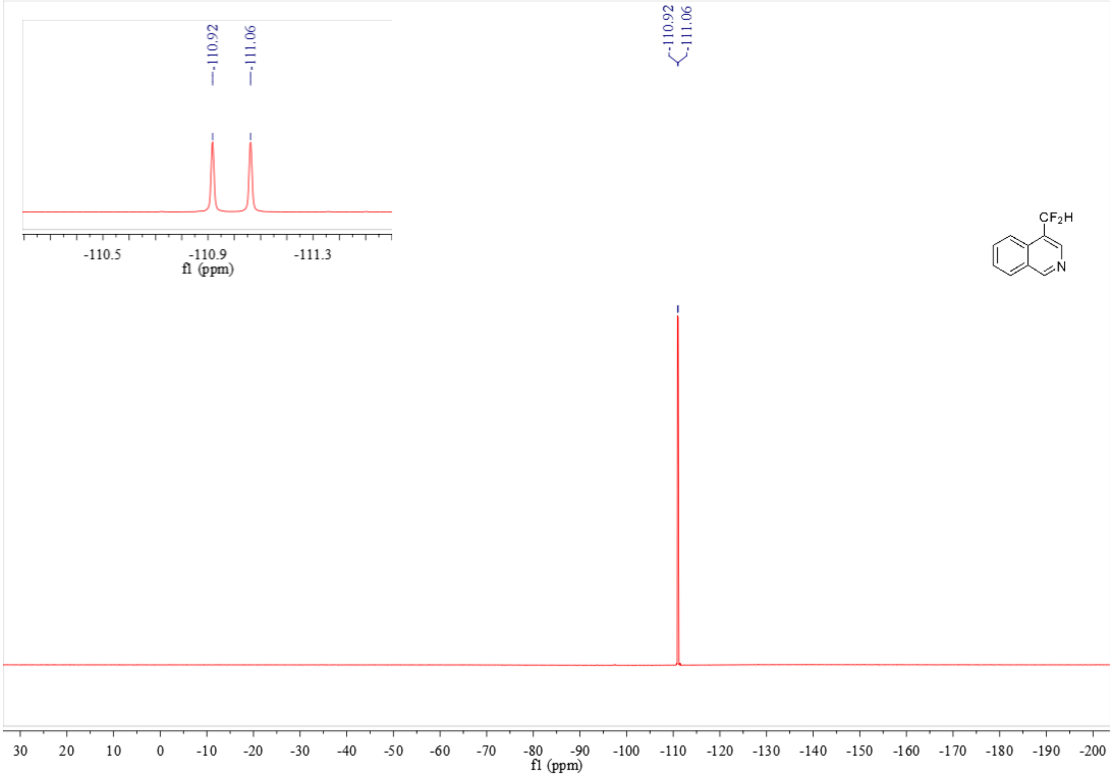

^{13}C NMR (101 MHz, $CDCl_3$) 4-(difluoromethyl)isoquinoline 4u

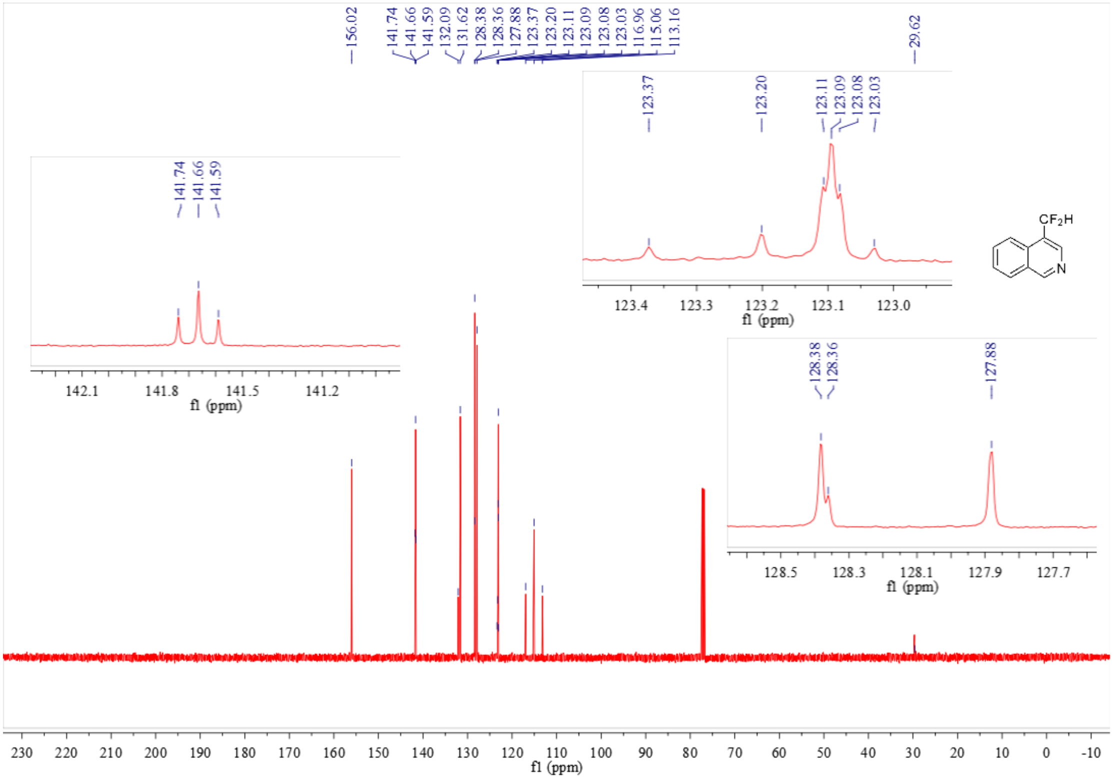

^{1}H NMR (400 MHz, $CDCl_3$) 3-(difluoromethyl)isoquinoline 4v

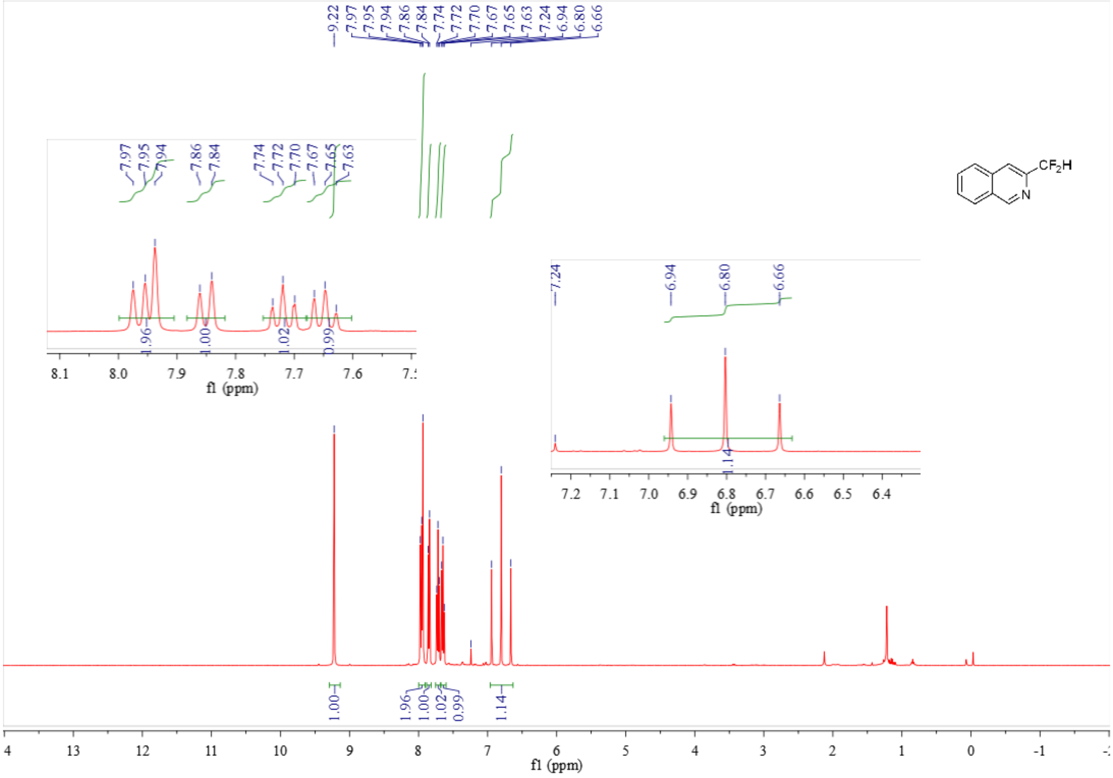

^{19}F NMR (376 MHz, $CDCl_3$) 3-(difluoromethyl)isoquinoline 4v

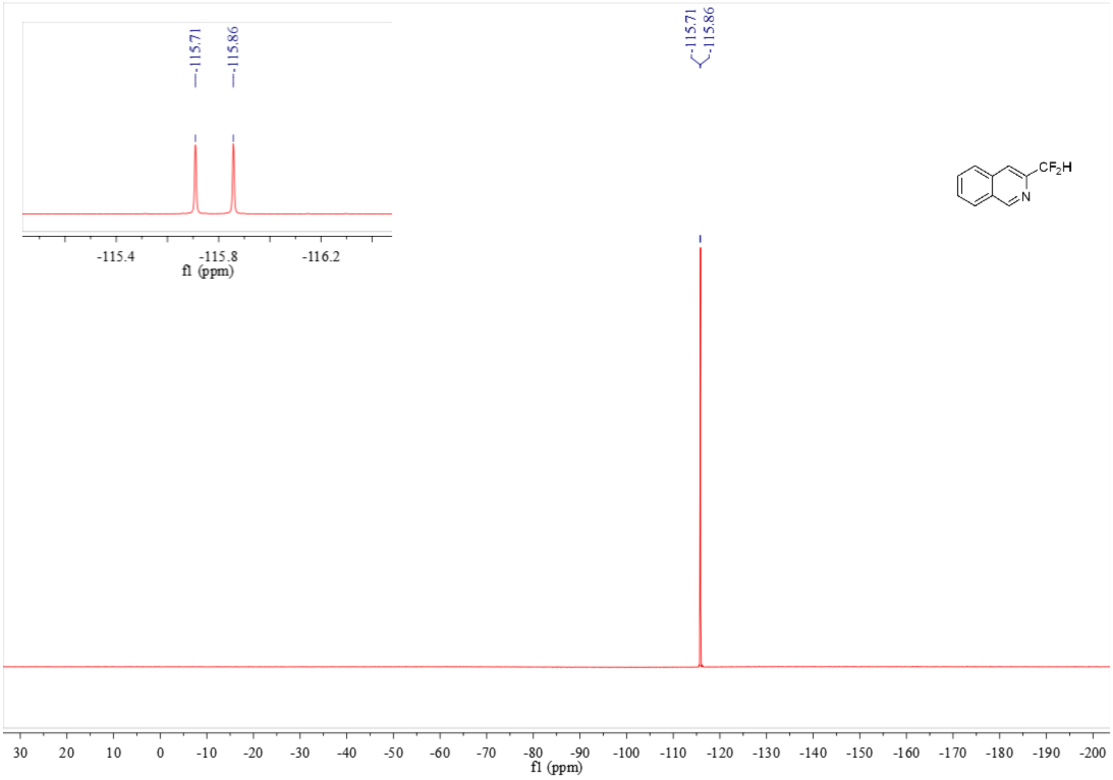

^{13}C NMR (101 MHz, $CDCl_3$) 3-(difluoromethyl)isoquinoline 4v

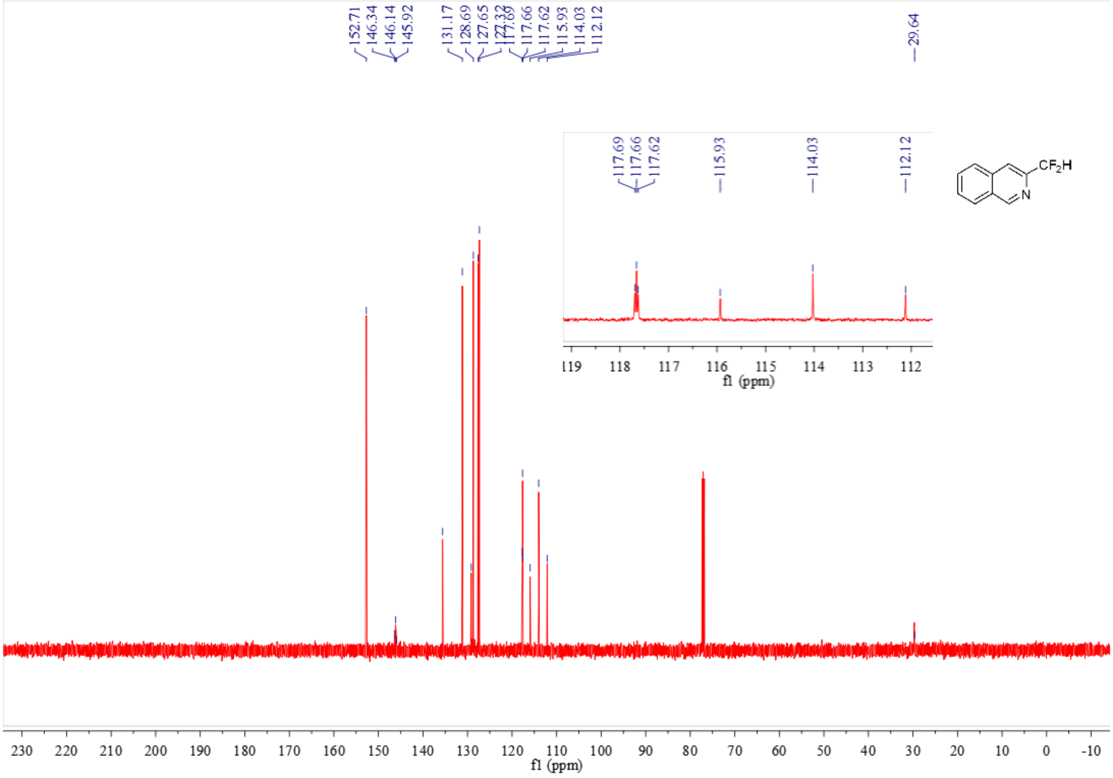

^{1}H NMR (400 MHz, $CDCl_3$) 1-(difluoromethyl)isoquinoline 4w

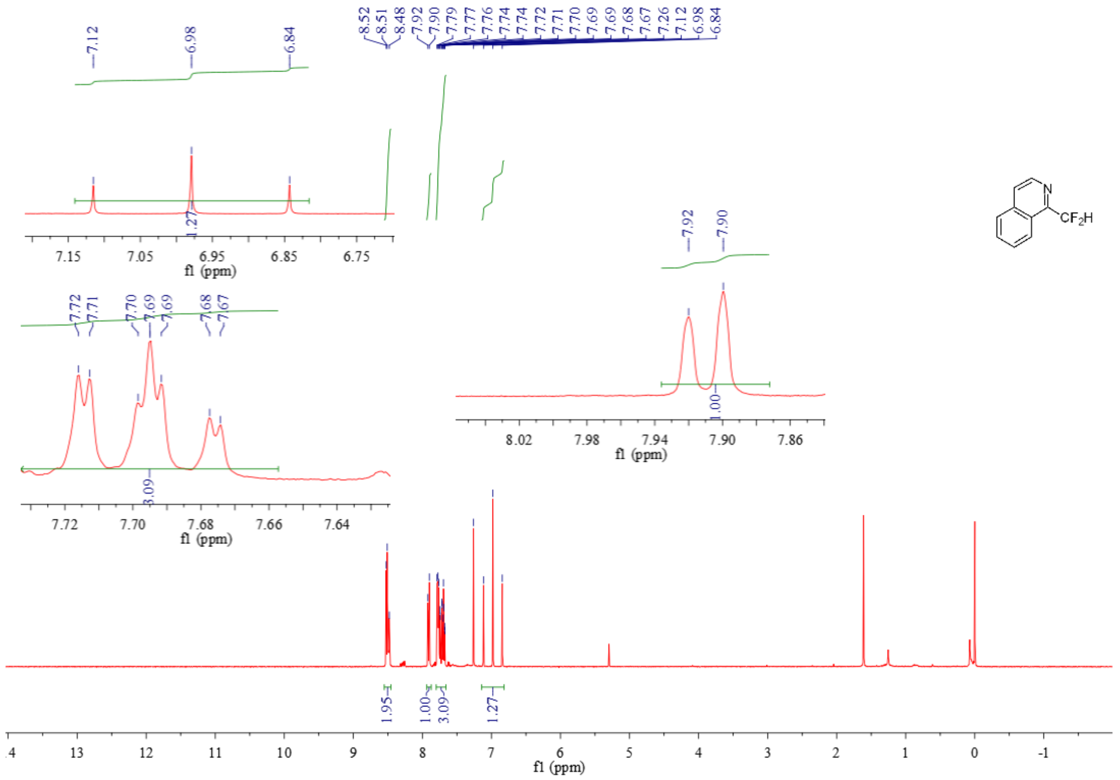

^{19}F NMR (376 MHz, $CDCl_3$) 1-(difluoromethyl)isoquinoline 4w

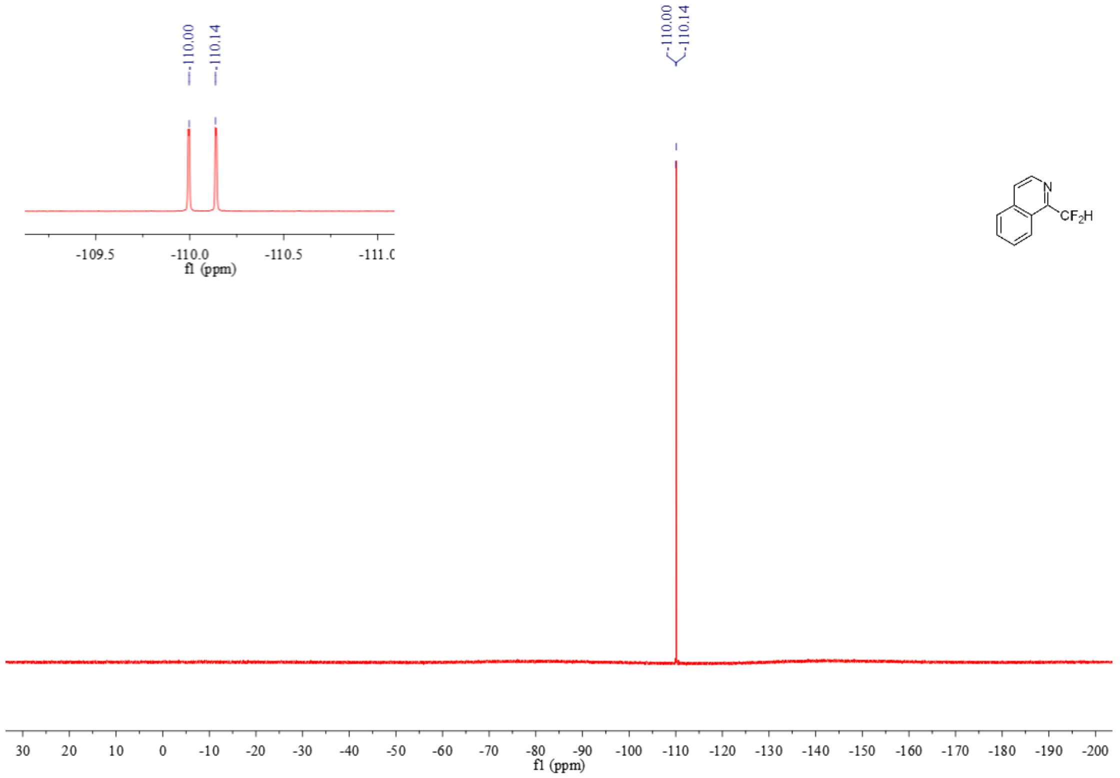

^{13}C NMR (101 MHz, $CDCl_3$) 1-(difluoromethyl)isoquinoline 4w

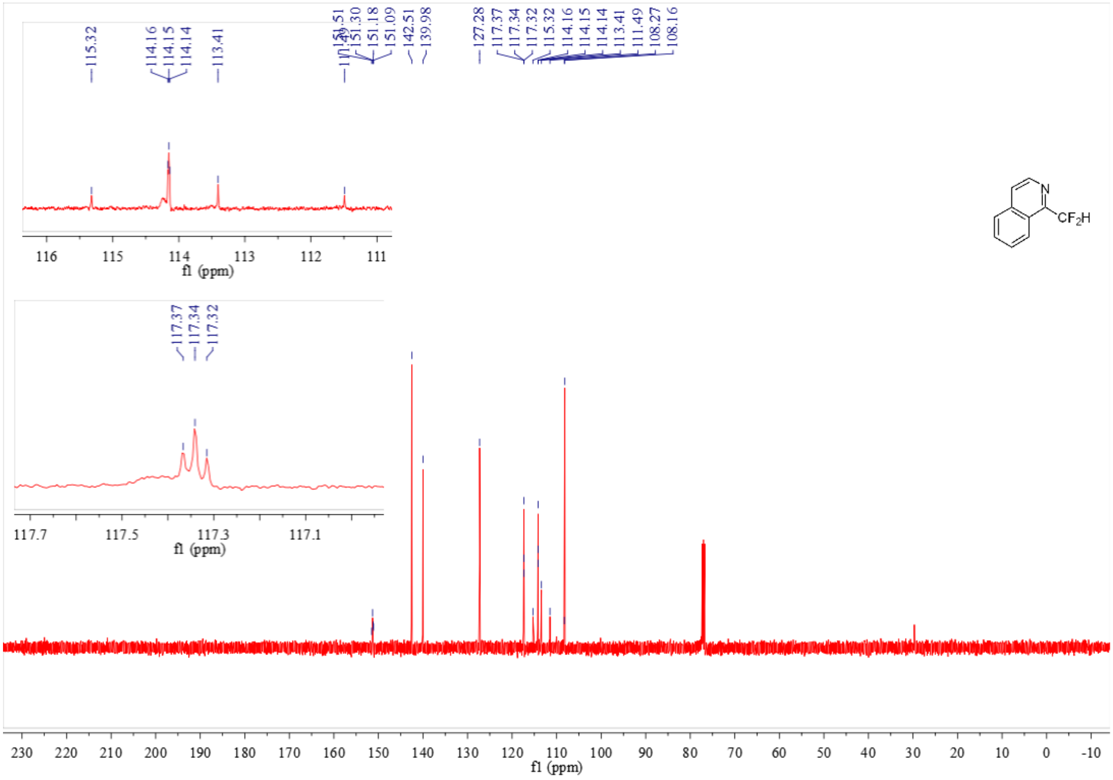

^{1}H NMR (400 MHz, $CDCl_3$) *tert*-butyl 6-(difluoromethyl)pyrrolo[2,3-b]pyridine-1-carboxylate 4x

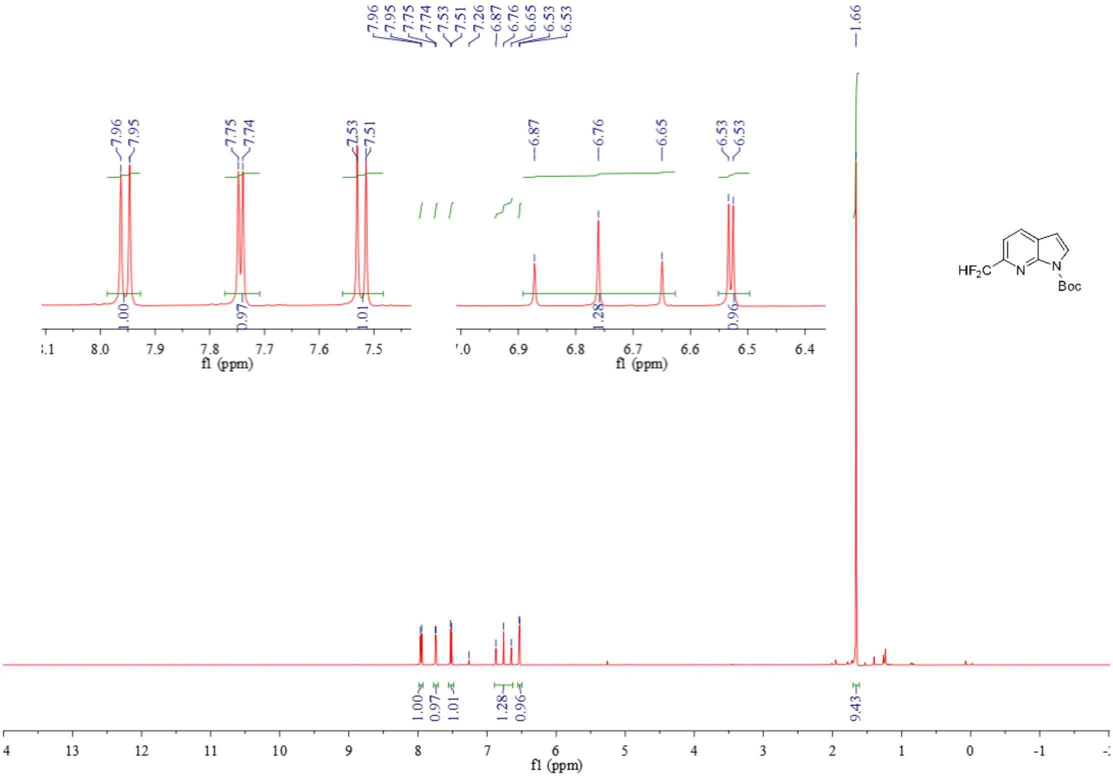

^{19}F NMR (376 MHz, $CDCl_3$) *tert*-butyl 6-(difluoromethyl)pyrrolo[2,3-b]pyridine-1-carboxylate 4x

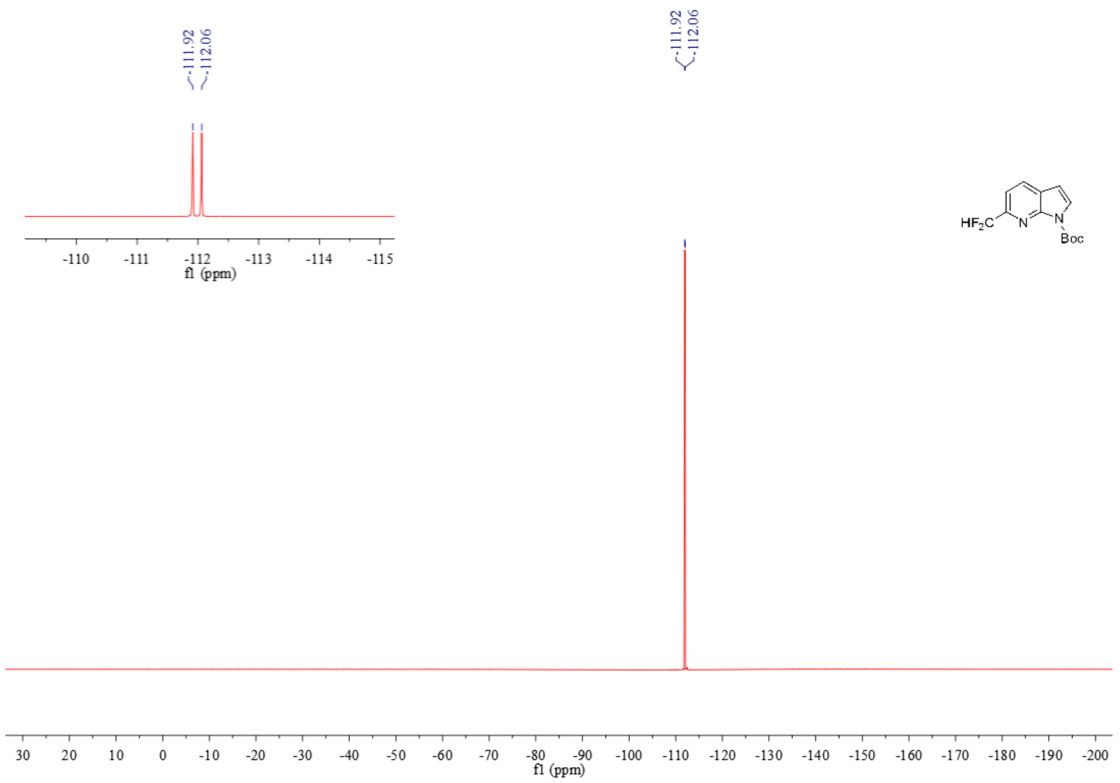

^{13}C NMR (101 MHz, $CDCl_3$) *tert*-butyl 6-(difluoromethyl)pyrrolo[2,3-b]pyridine-1-carboxylate 4x

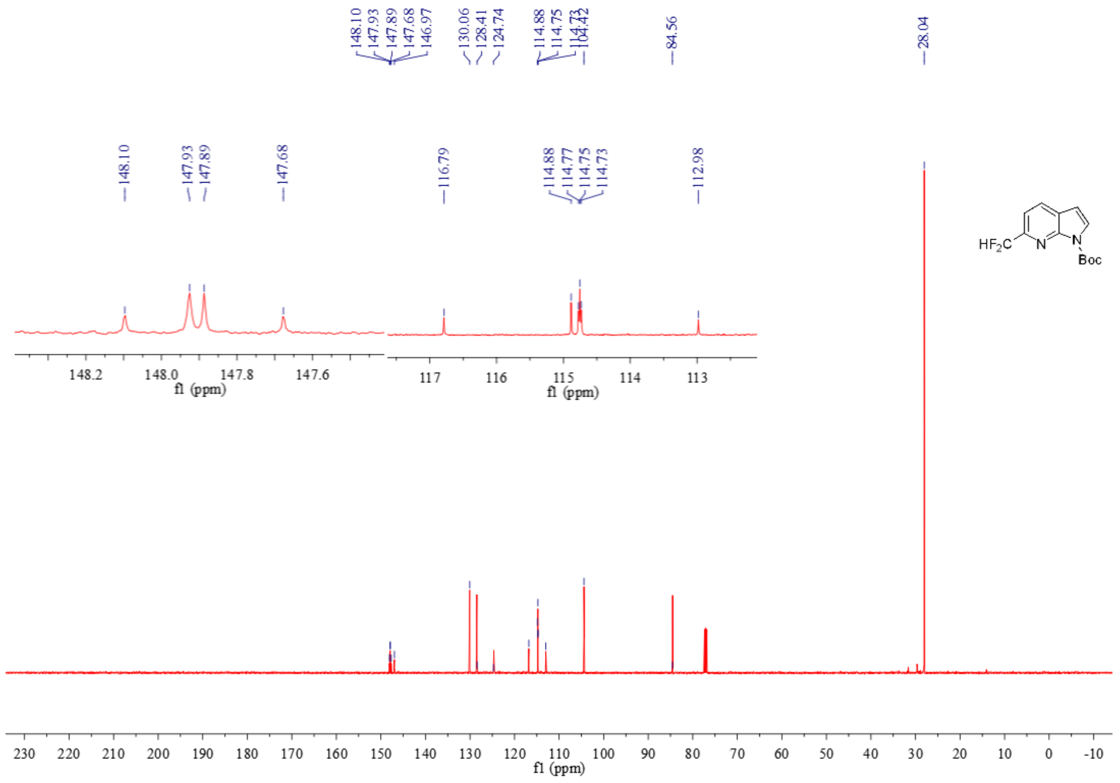

^{1}H NMR (400 MHz, $CDCl_3$) 2-(difluoromethyl)quinoxaline 4y

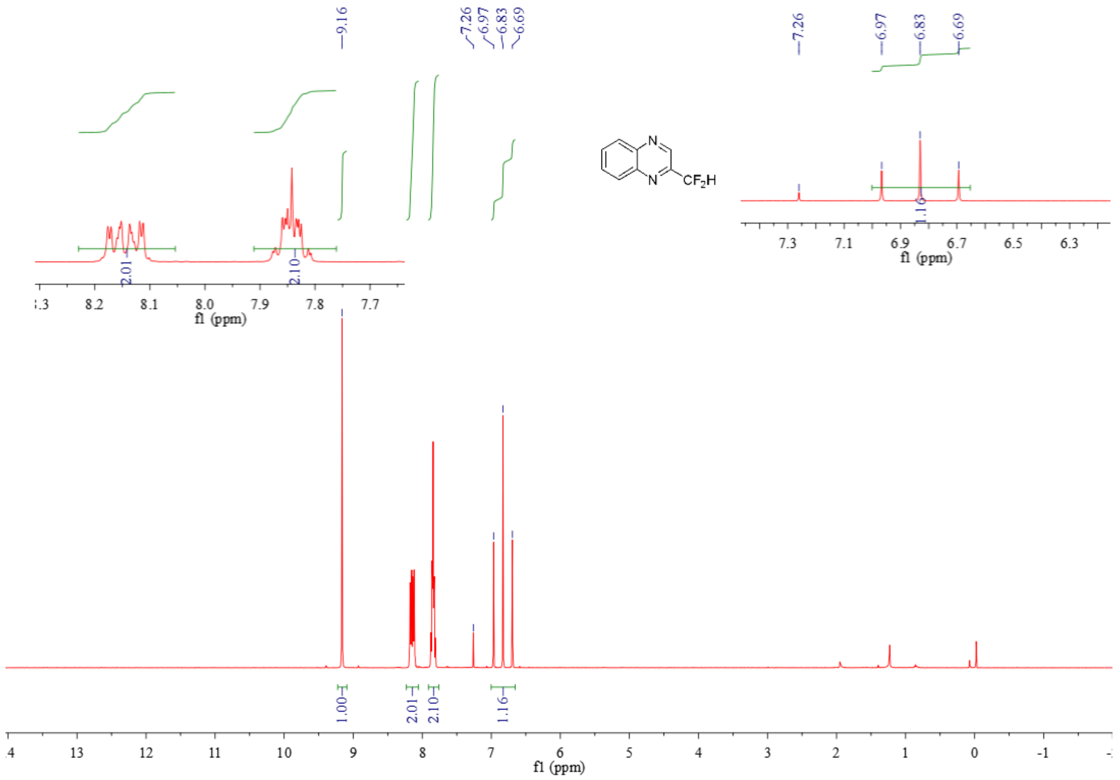

^{19}F NMR (376 MHz, $CDCl_3$) 2-(difluoromethyl)quinoxaline 4y

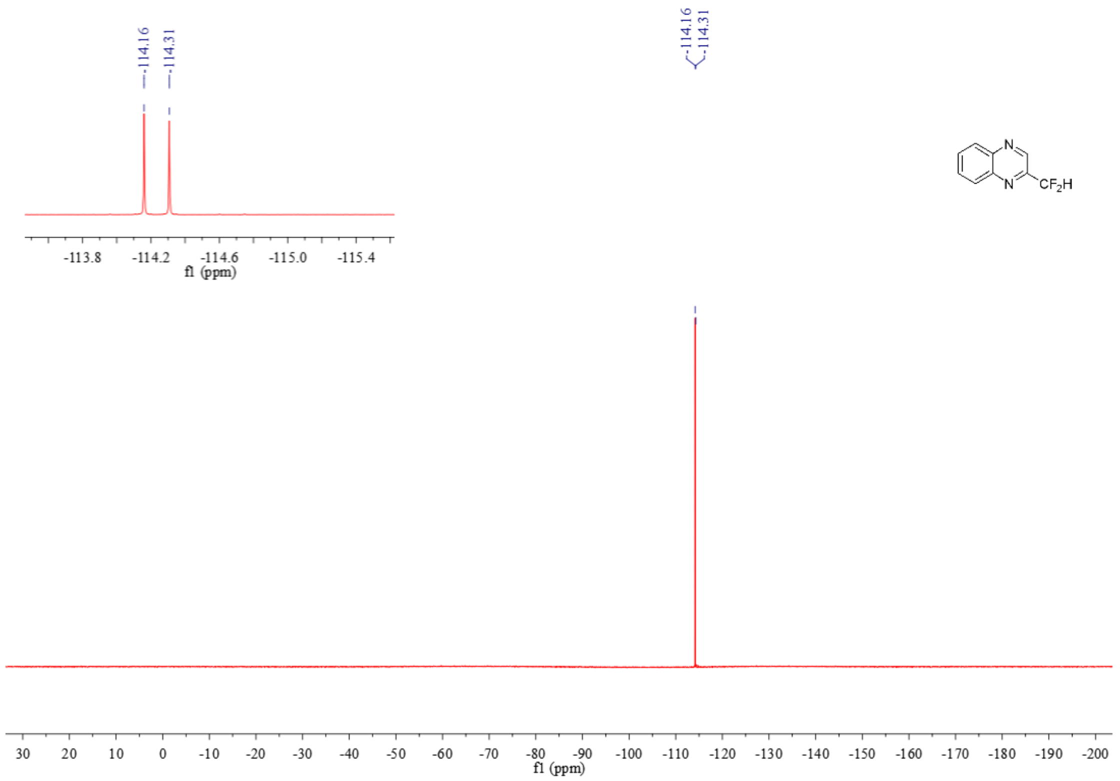

^{13}C NMR (101 MHz, $CDCl_3$) 2-(difluoromethyl)quinoxaline 4y

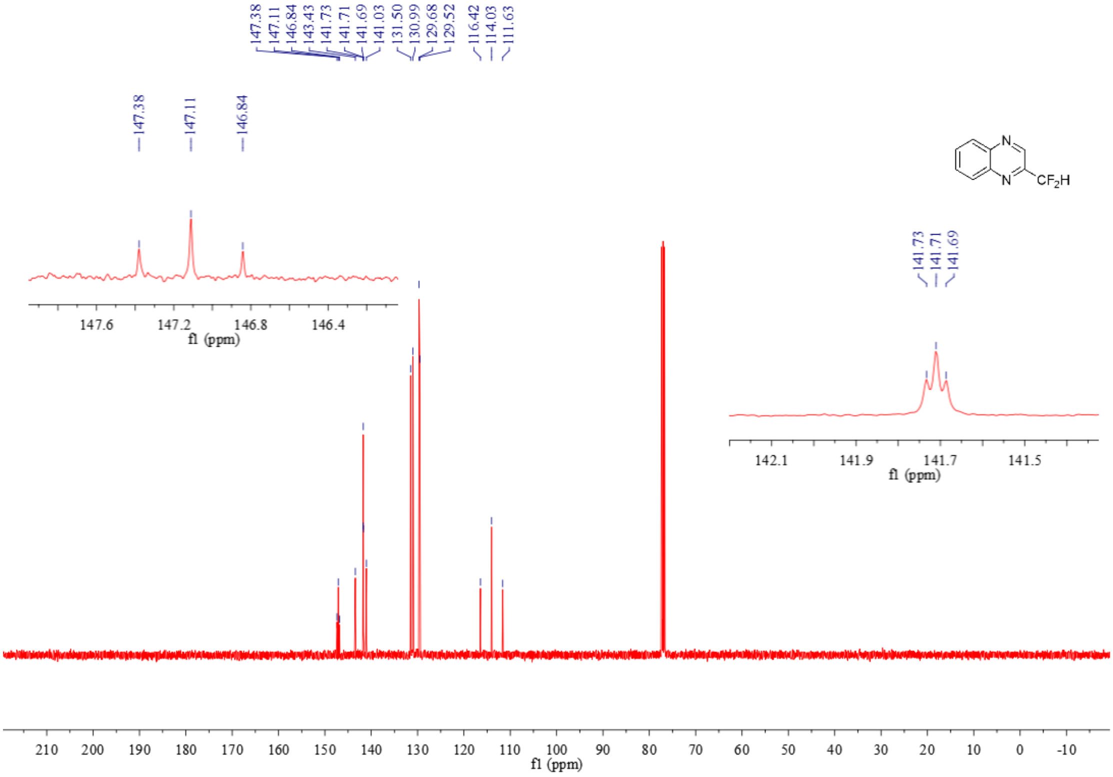

^{1}H NMR (400 MHz, $CDCl_3$) 6-(difluoromethyl)imidazo[1,2-a]pyridine 4z

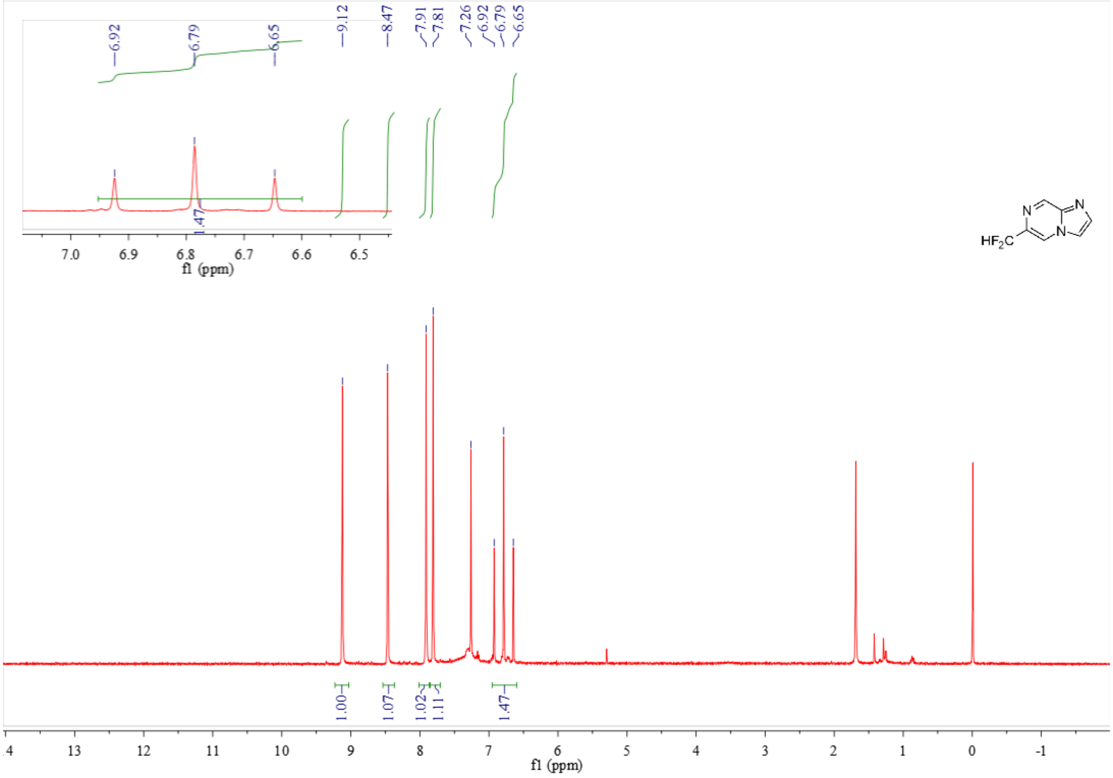

^{19}F NMR (376 MHz, $CDCl_3$) 6-(difluoromethyl)imidazo[1,2-a]pyridine 4z

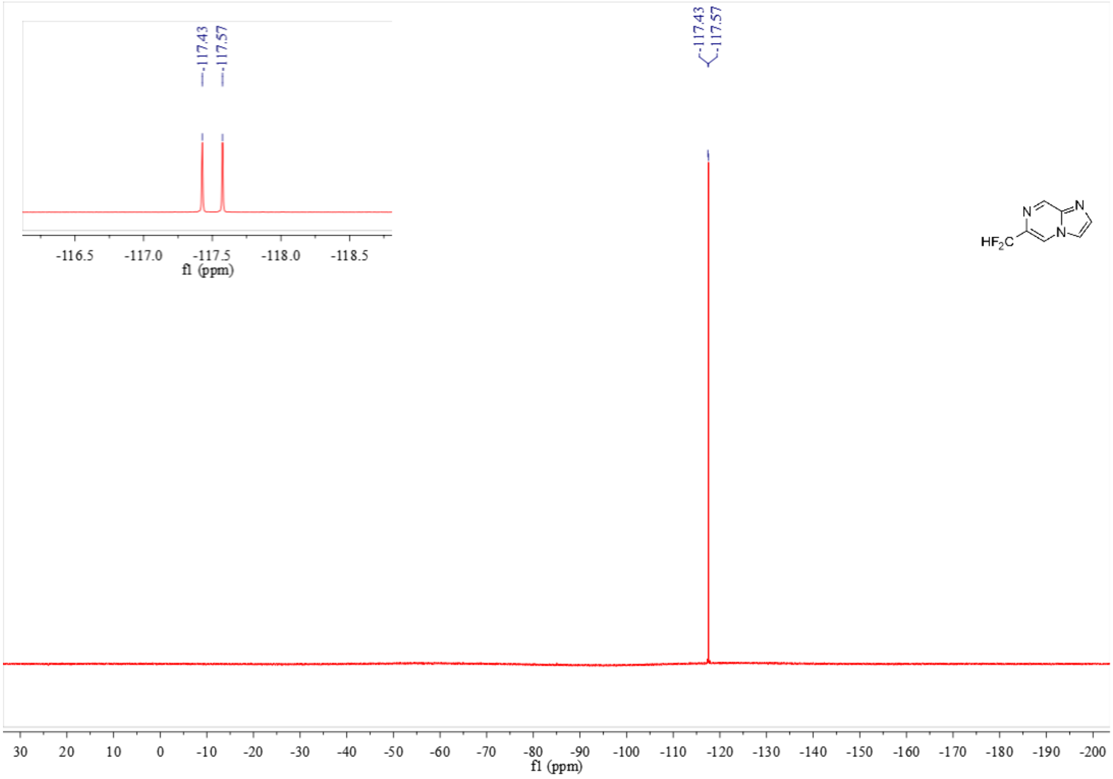

^{13}C NMR (101 MHz, $CDCl_3$) 2-(difluoromethyl)quinoxaline 4z

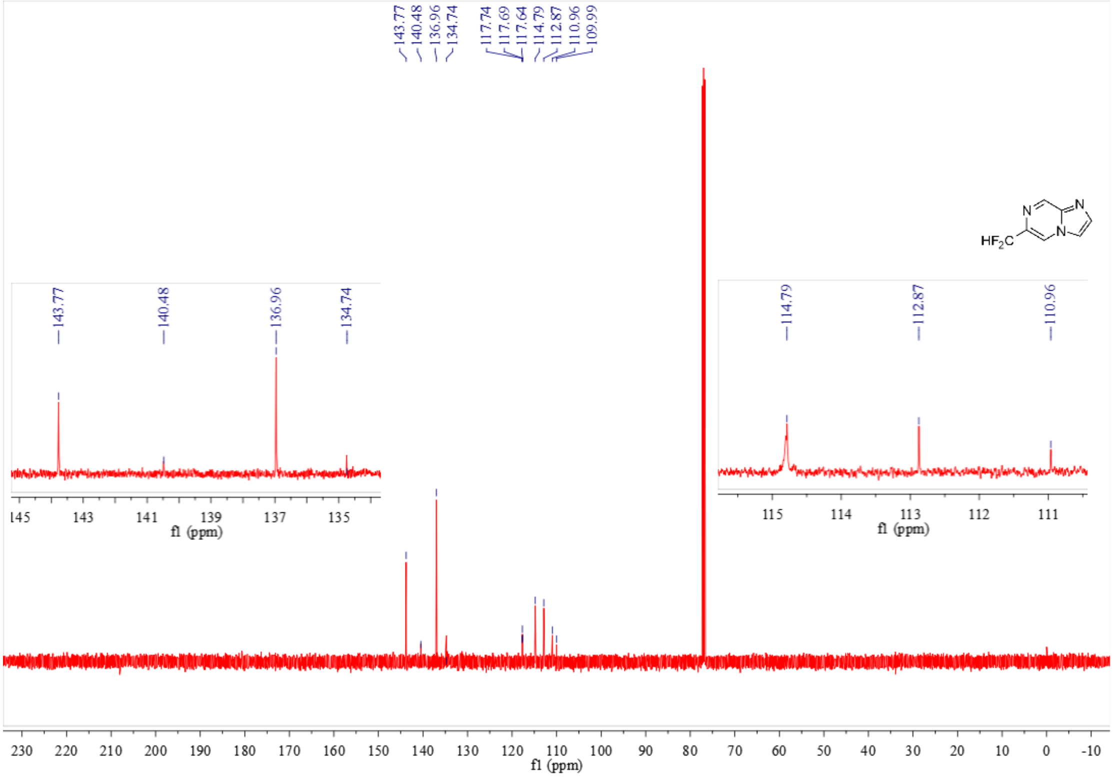

^{1}H NMR (400 MHz, $CDCl_3$) 7-(difluoromethyl)pyrido[2,3-b]pyrazine 4aa

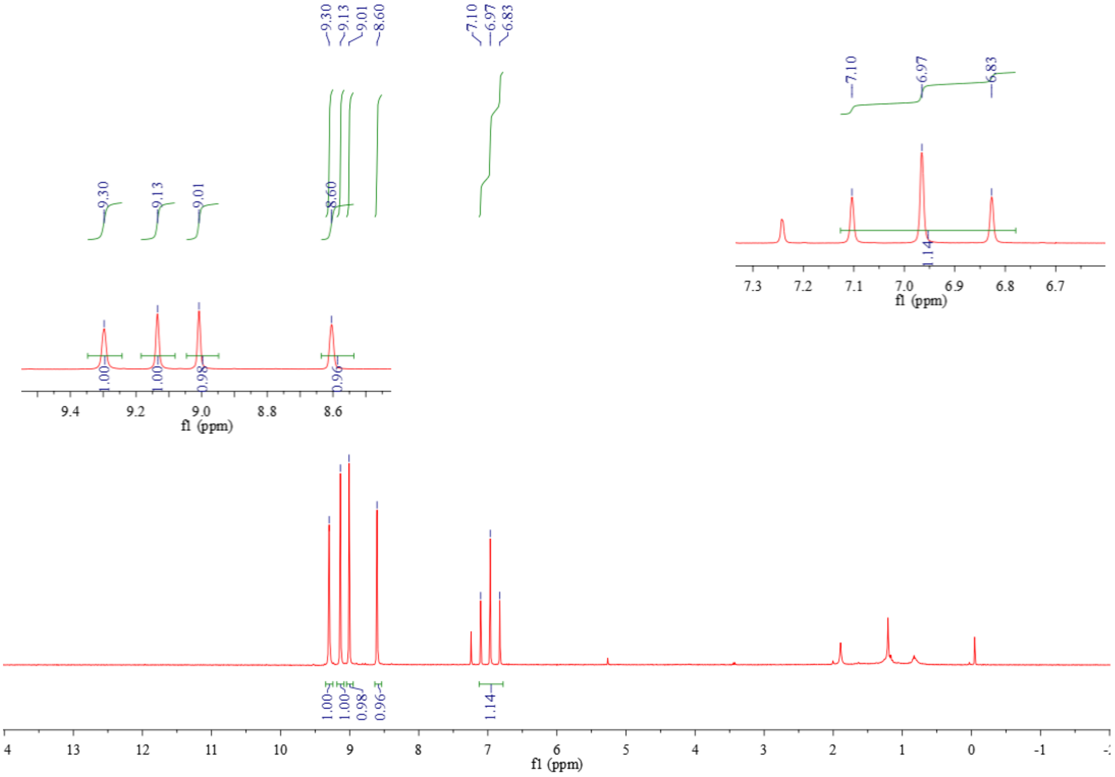

^{19}F NMR (376 MHz, $CDCl_3$) 7-(difluoromethyl)pyrido[2,3-b]pyrazine 4aa

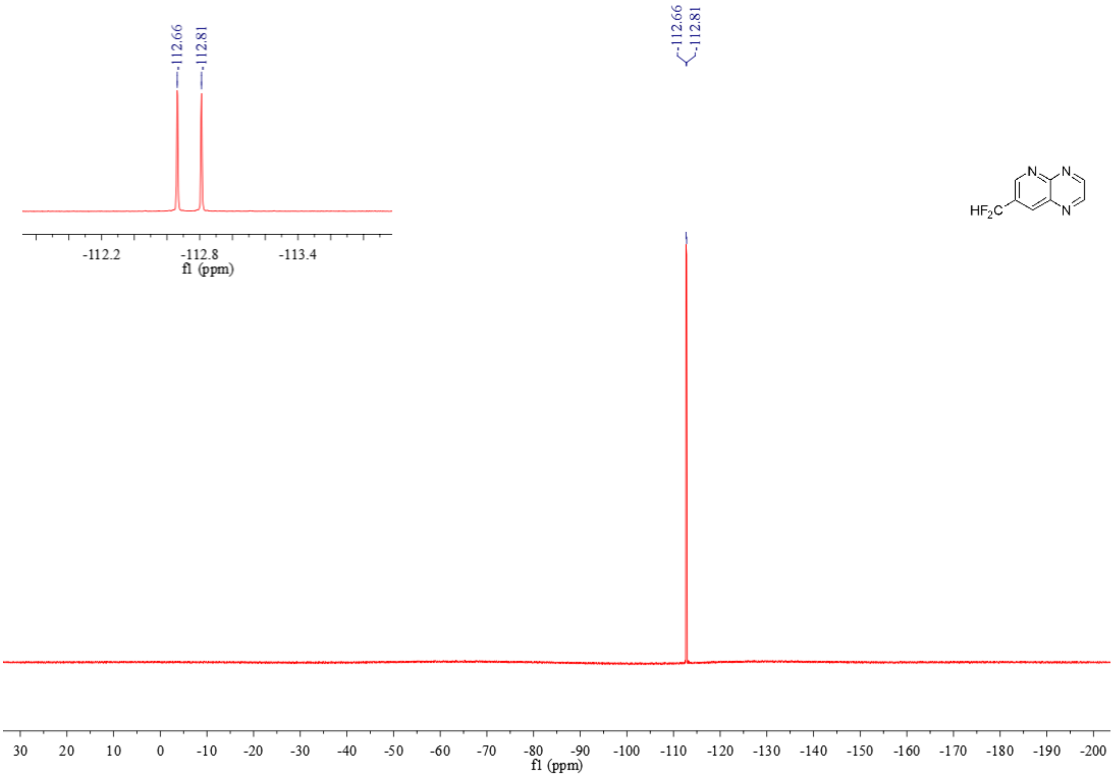

^{13}C NMR (101 MHz, $CDCl_3$) 7-(difluoromethyl)pyrido[2,3-b]pyrazine 4aa

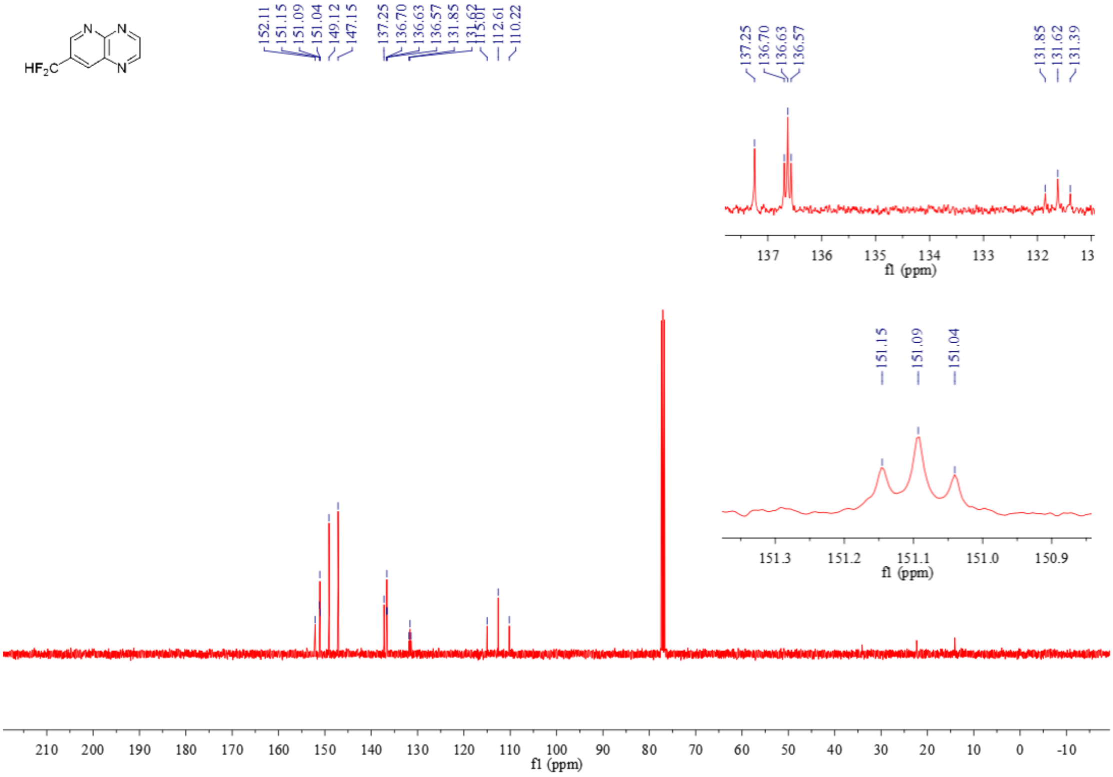

^{1}H NMR (400 MHz, $CDCl_3$) 1-[5-(difluoromethyl)-2-thienyl]ethanone 4ab

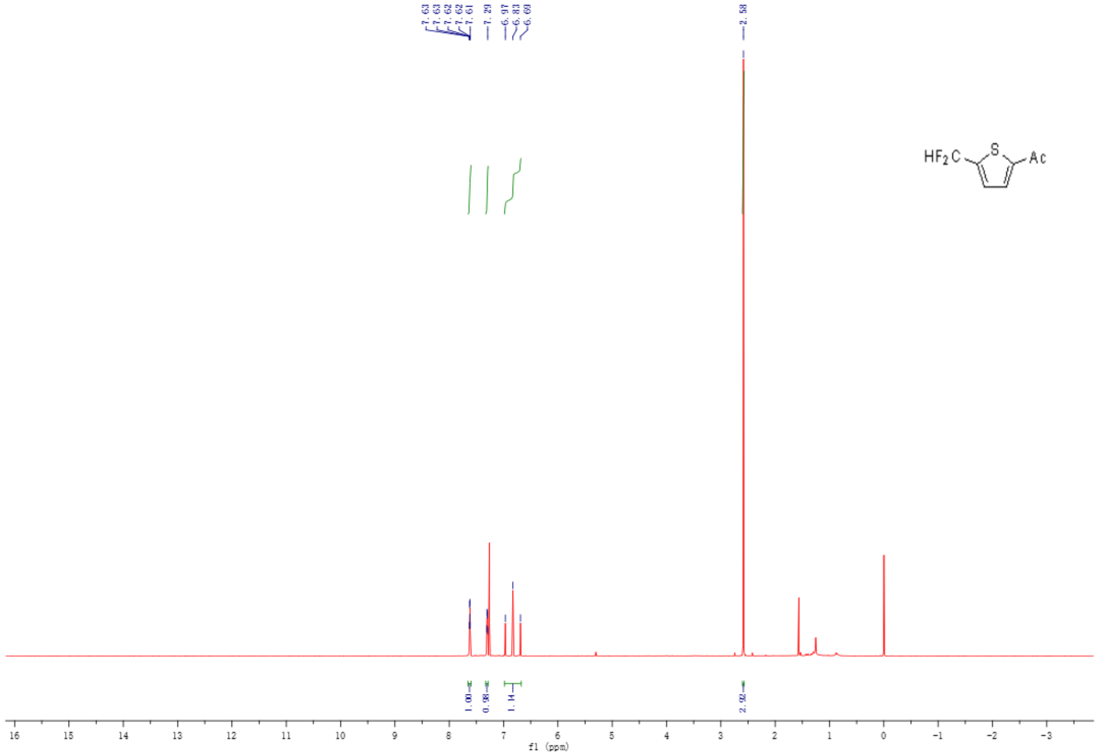

^{19}F NMR (376 MHz, $CDCl_3$) 1-[5-(difluoromethyl)-2-thienyl]ethanone 4ab

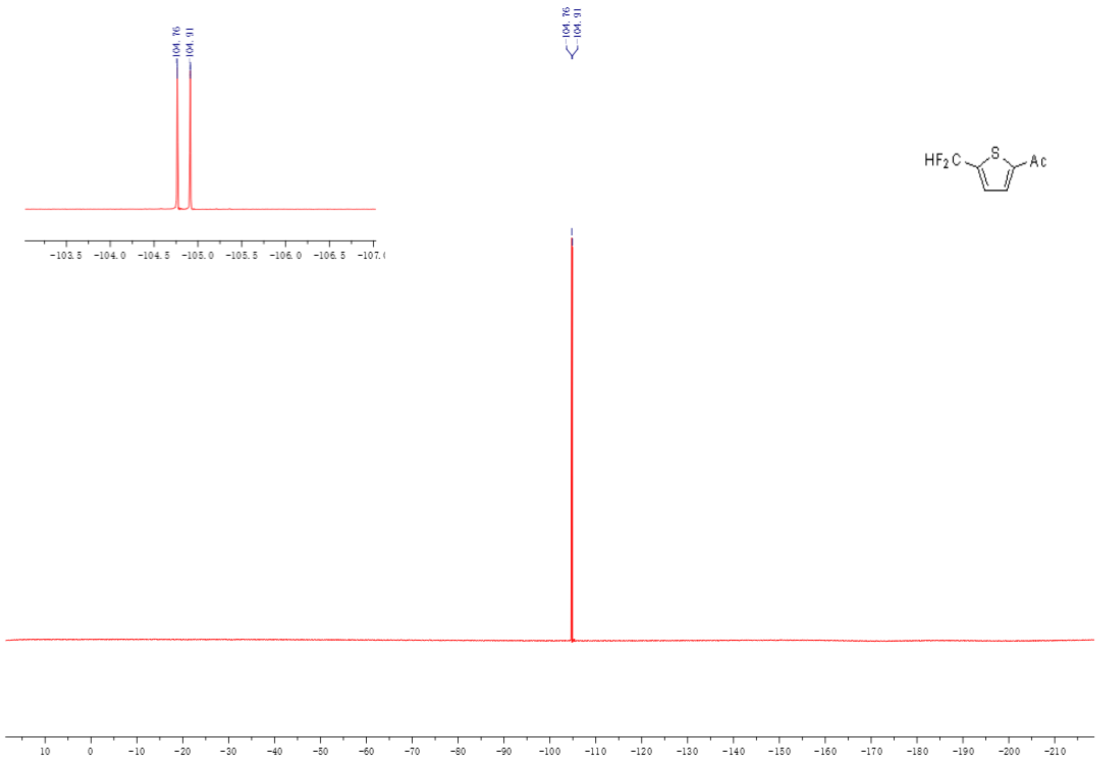

^{13}C NMR (101 MHz, $CDCl_3$) 1-[5-(difluoromethyl)-2-thienyl]ethanone 4ab

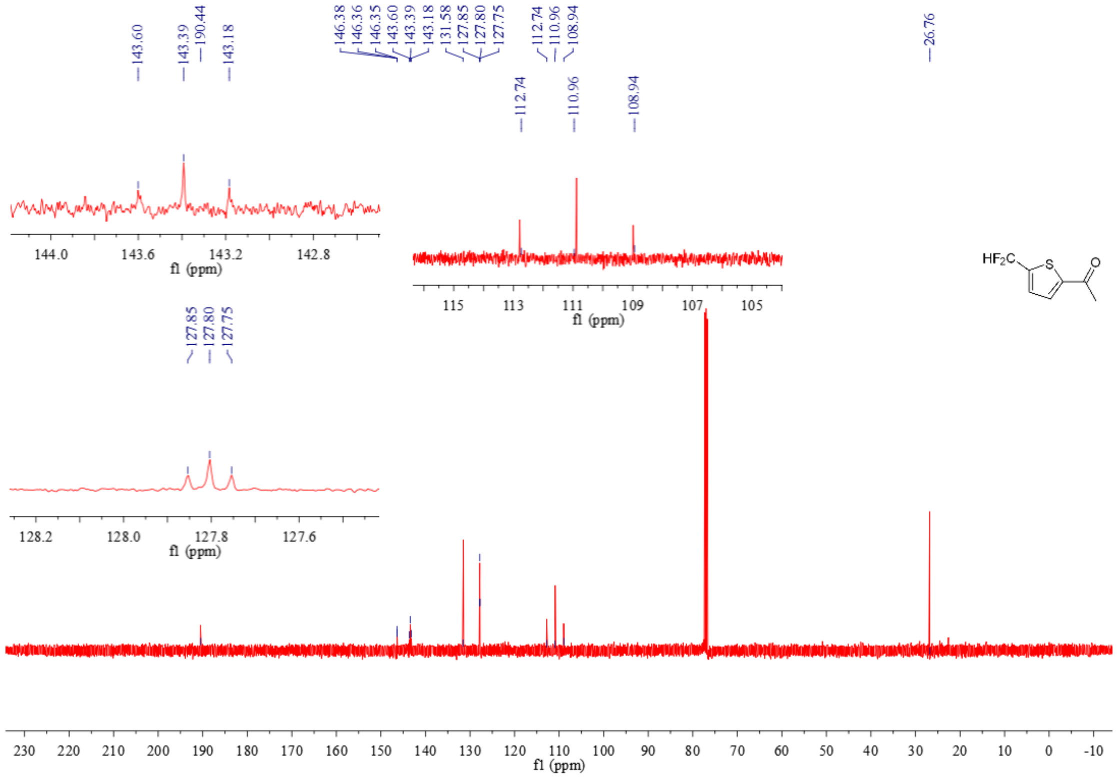

^{1}H NMR (400 MHz, $CDCl_3$) 2-[5-(difluoromethyl)-2-thienyl]pyridine 4ac

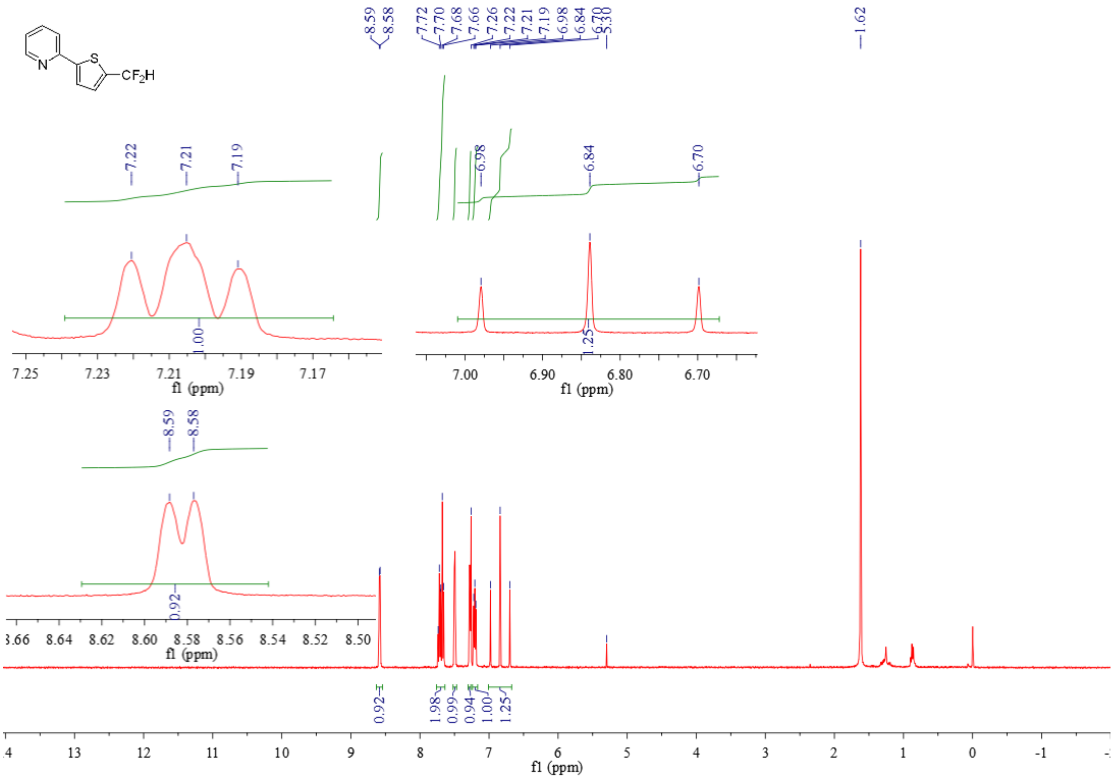

^{19}F NMR (376 MHz, $CDCl_3$) 2-[5-(difluoromethyl)-2-thienyl]pyridine 4ac

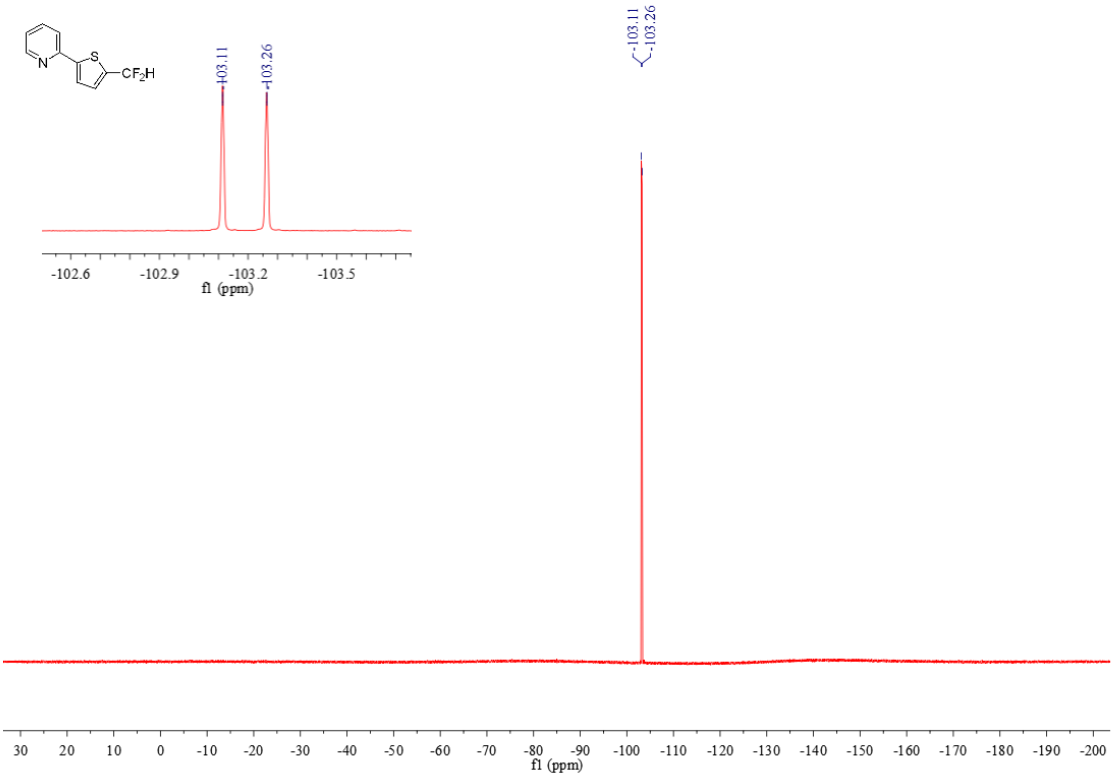

^{13}C NMR (101 MHz, $CDCl_3$) 2-[5-(difluoromethyl)-2-thienyl]pyridine 4ac

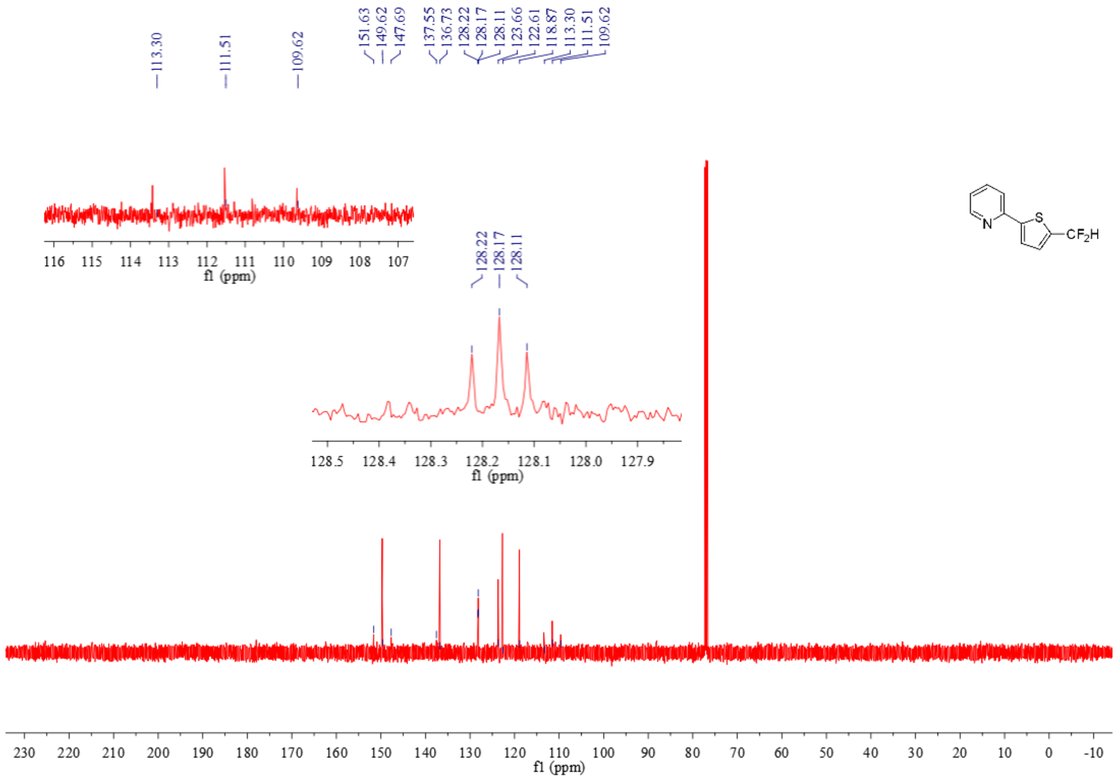

^{1}H NMR (400 MHz, $CDCl_3$) 2-(difluoromethyl)-5-methyl-thiophene-3-carbonitrile 4ad

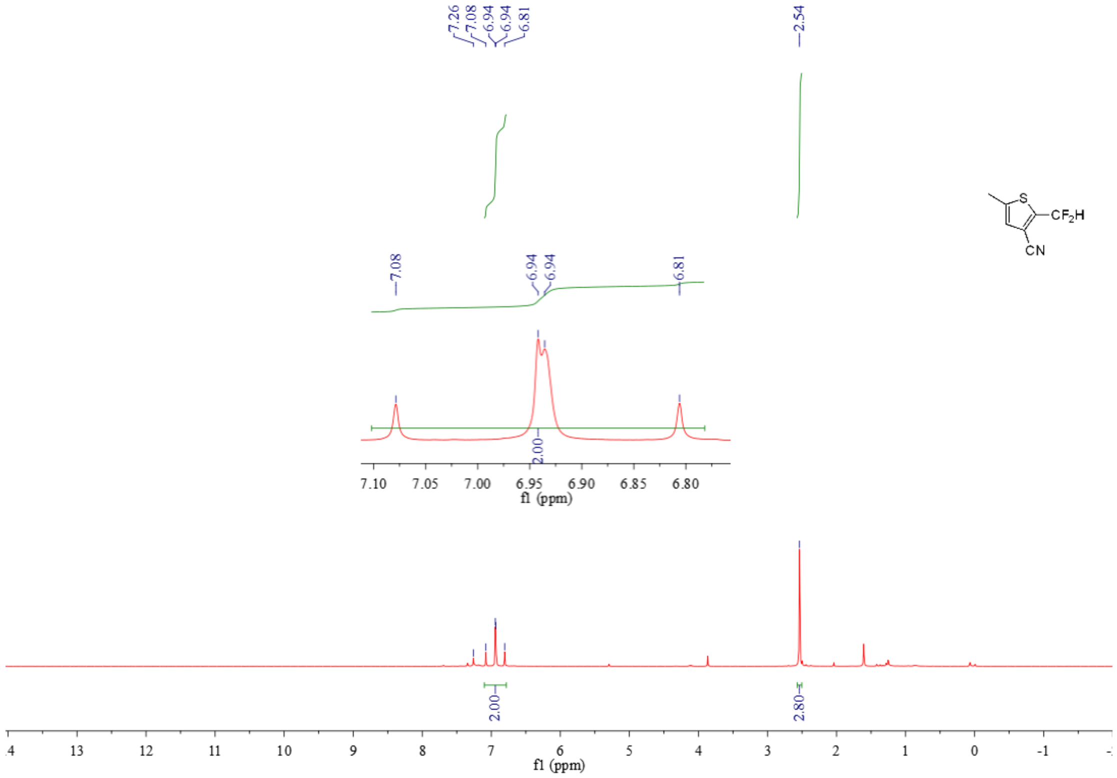

^{19}F NMR (376 MHz, $CDCl_3$) 2-(difluoromethyl)-5-methyl-thiophene-3-carbonitrile 4ad

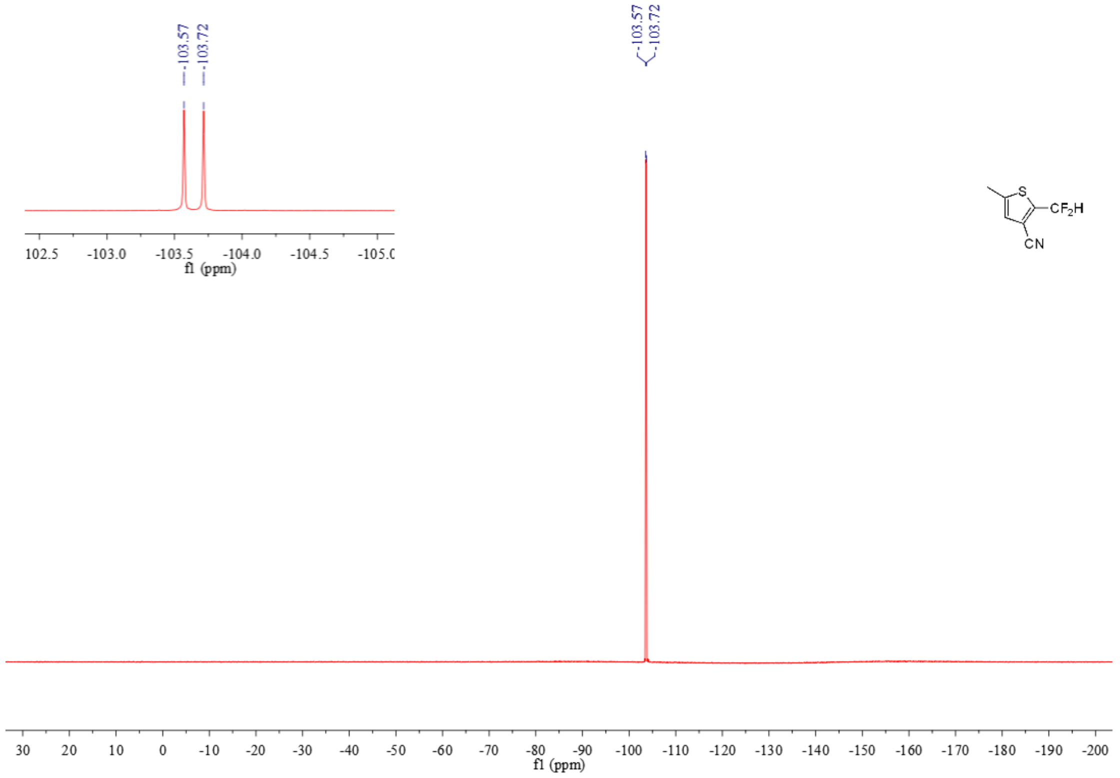

^{13}C NMR (101 MHz, $CDCl_3$) 2-(difluoromethyl)-5-methyl-thiophene-3-carbonitrile 4ad

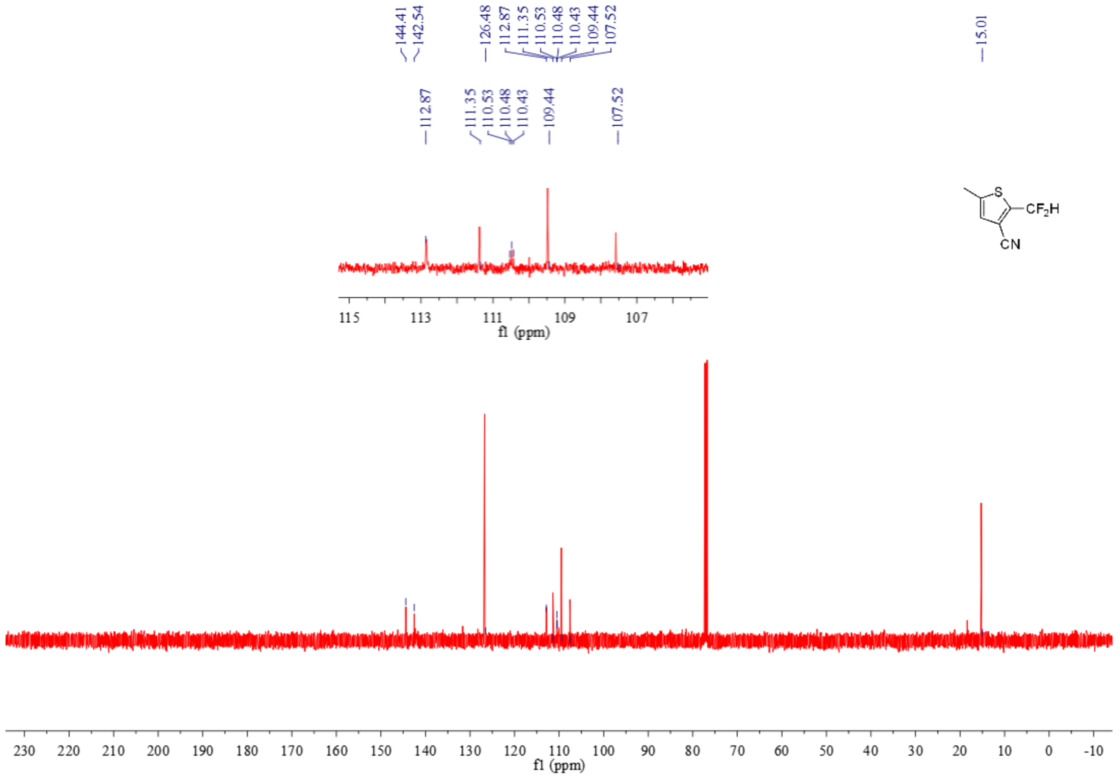

^{1}H NMR (400 MHz, $CDCl_3$) 2-(difluoromethyl)benzothiophene 4ae

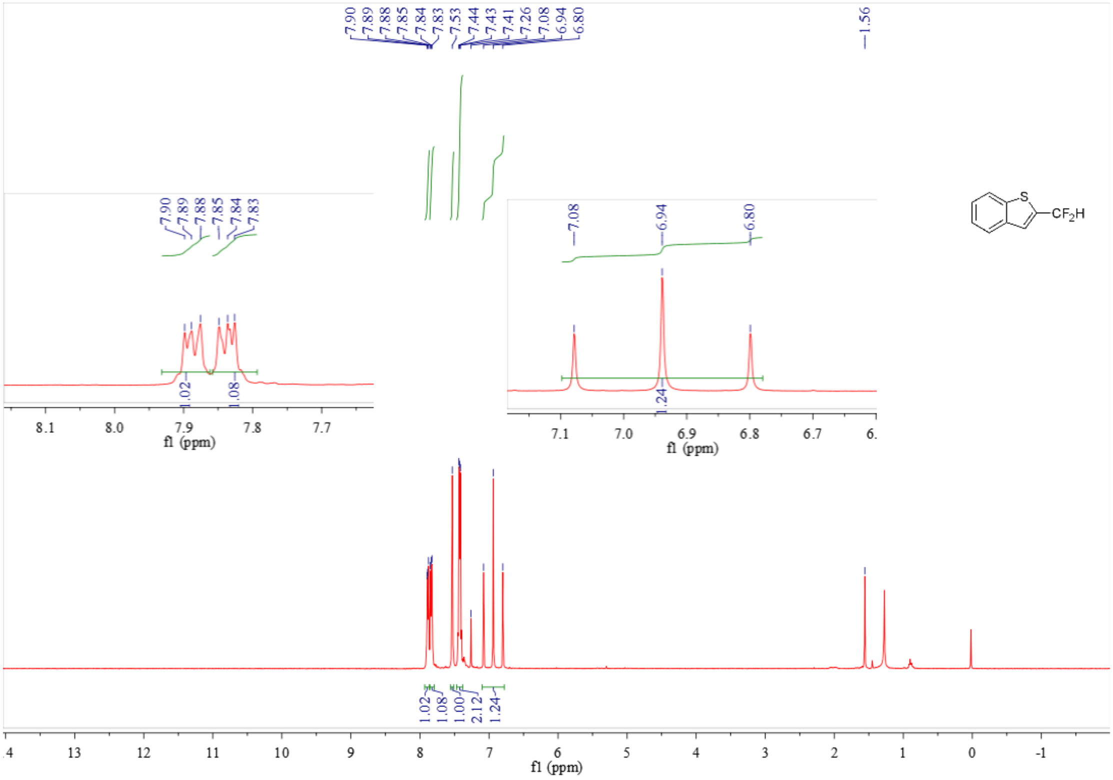

^{19}F NMR (376 MHz, $CDCl_3$) 2-(difluoromethyl)benzothiophene 4ae.

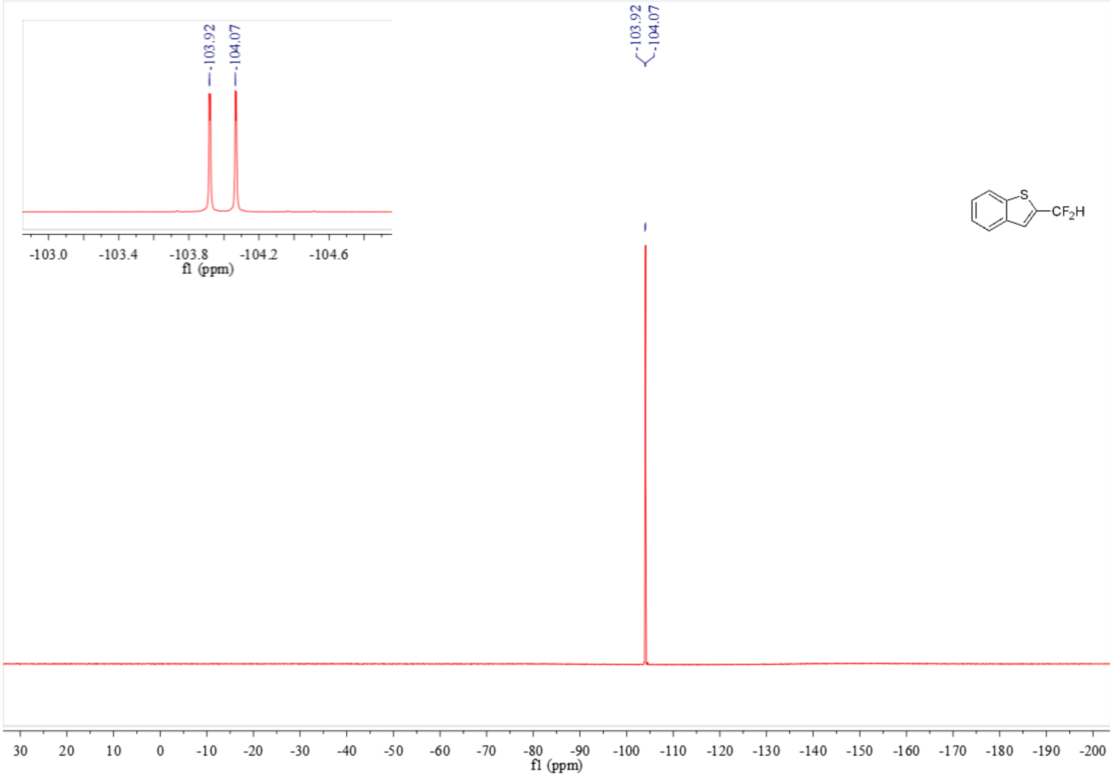

^{13}C NMR (101 MHz, $CDCl_3$) 2-(difluoromethyl)benzothiophene 4ae

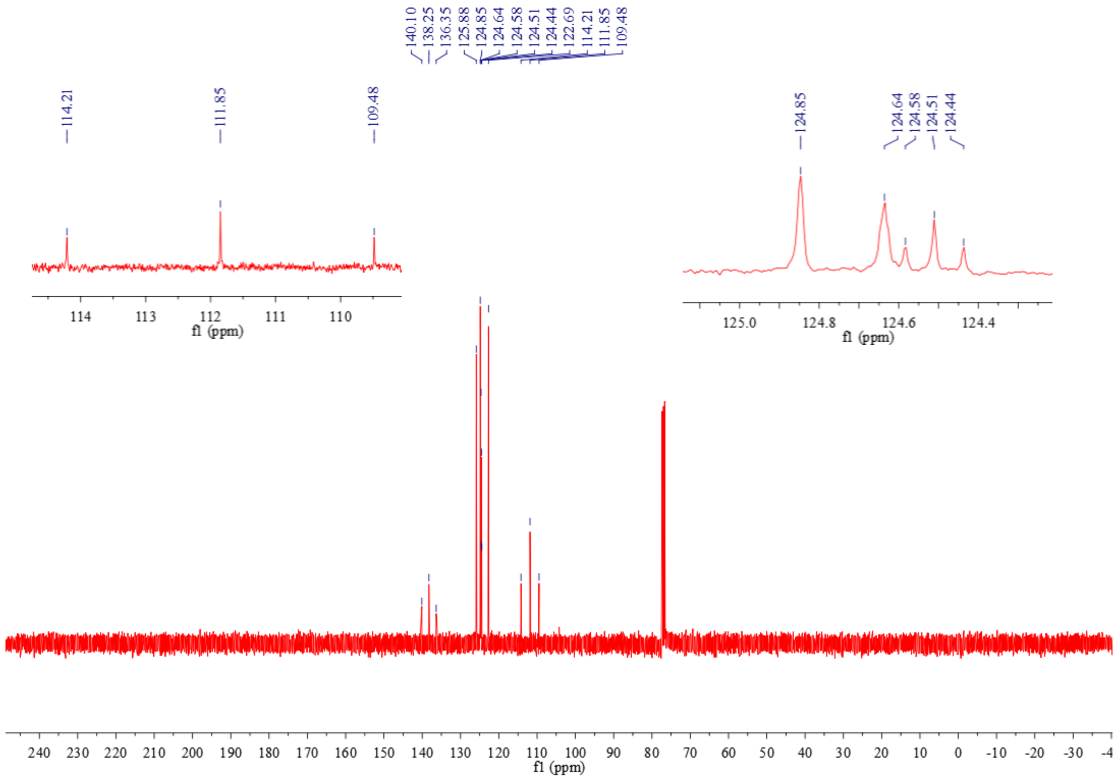

^{1}H NMR (400 MHz, $CDCl_3$) 3-(difluoromethyl)benzothiophene 4af

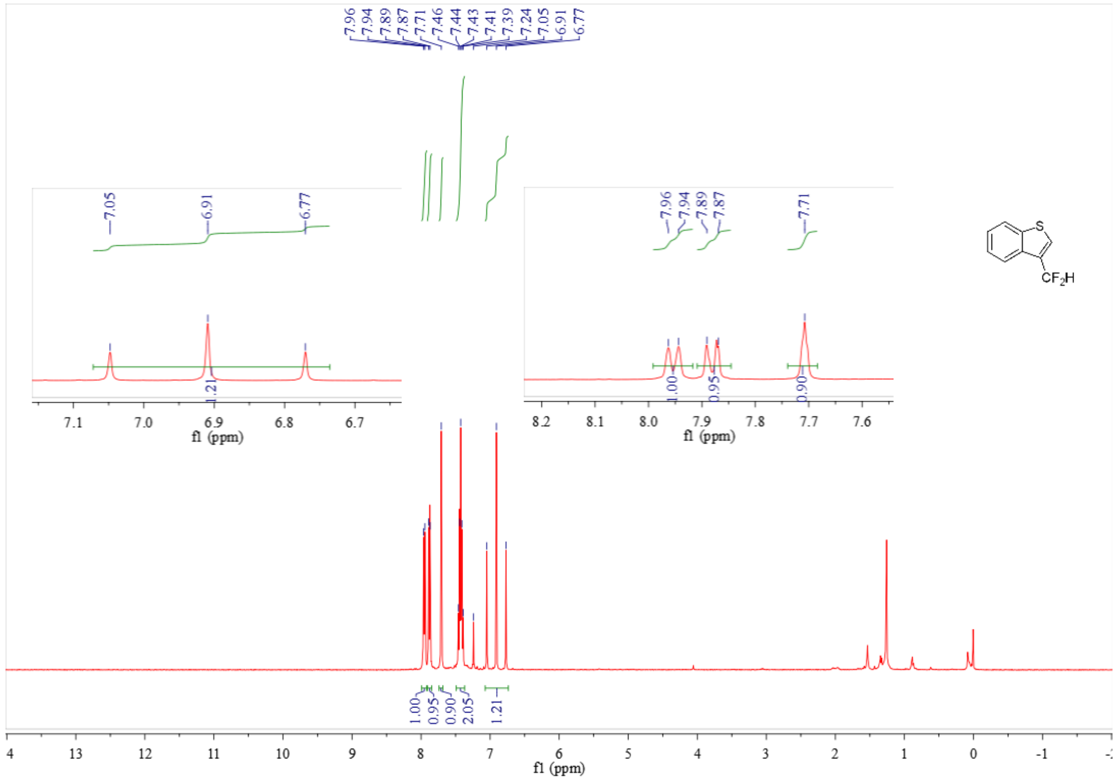

^{19}F NMR (376 MHz, $CDCl_3$) 3-(difluoromethyl)benzothiophene 4af

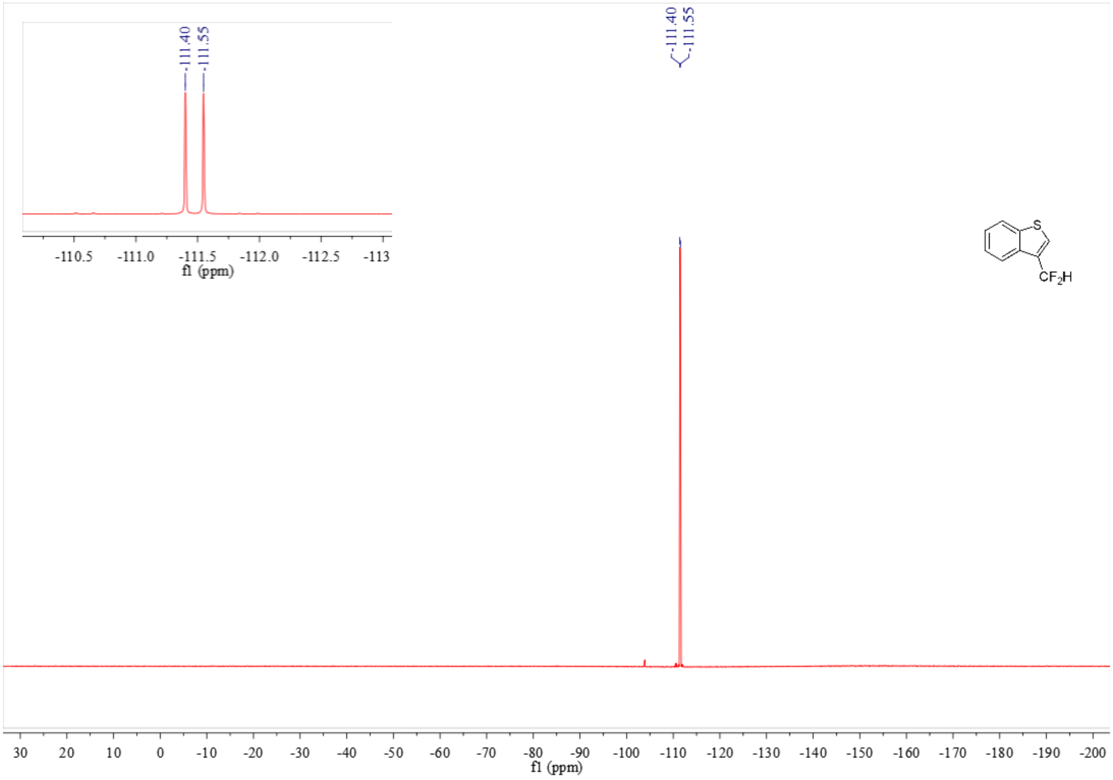

^{13}C NMR (101 MHz, $CDCl_3$) 3-(difluoromethyl)benzothiophene 4af

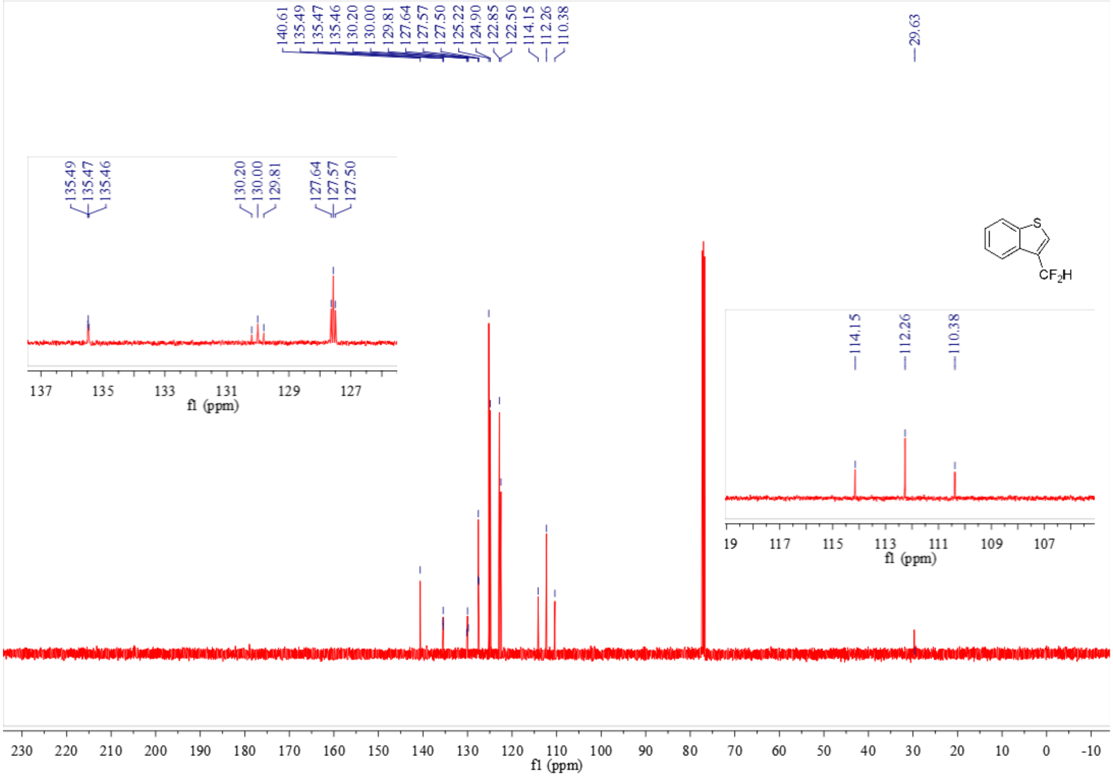

^{1}H NMR (400 MHz, $CDCl_3$) methyl 5-(difluoromethyl)furan-2-carboxylate 4ag

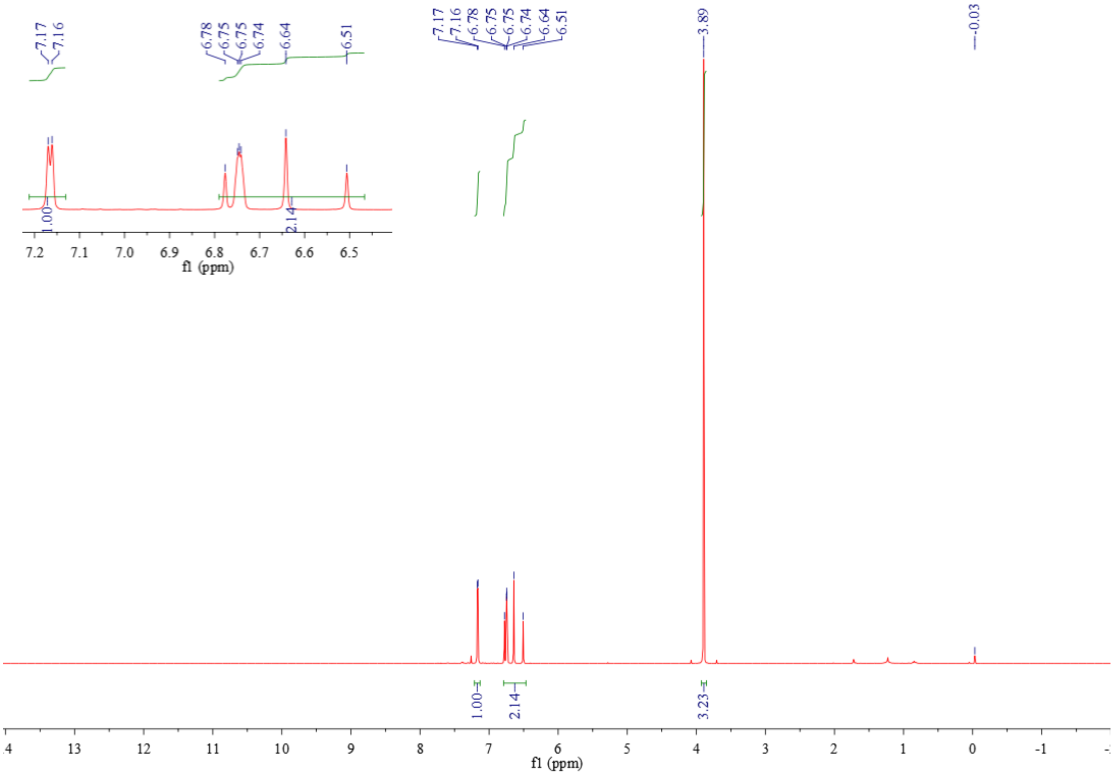

^{19}F NMR (376 MHz, $CDCl_3$) methyl 5-(difluoromethyl)furan-2-carboxylate 4ag

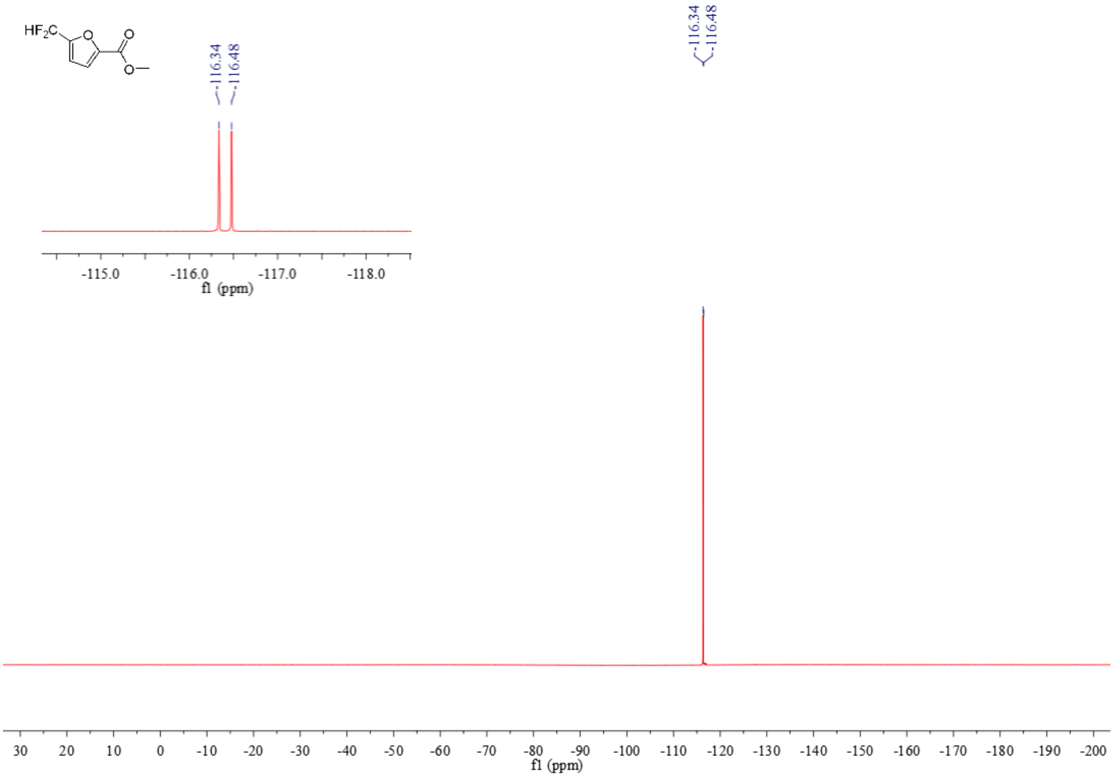

^{13}C NMR (101 MHz, $CDCl_3$) methyl 5-(difluoromethyl)furan-2-carboxylate 4ag

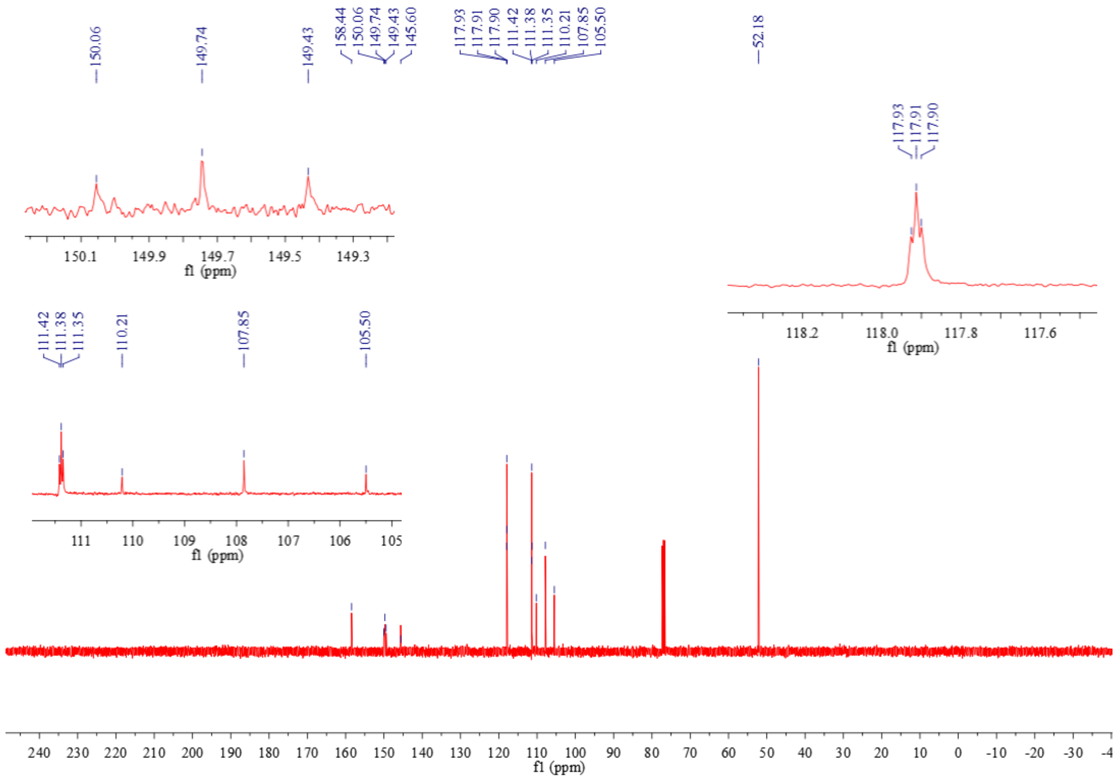

^{1}H NMR (400 MHz, $CDCl_3$) 2-(difluoromethyl)dibenzofuran 4ah

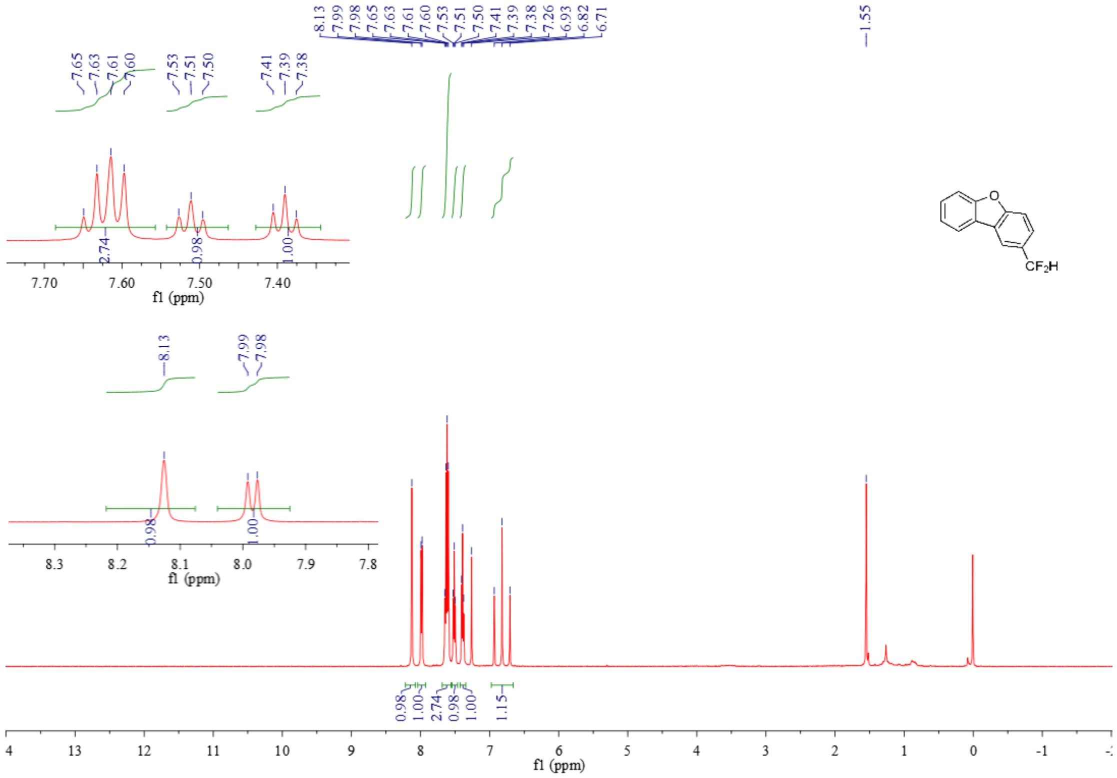

^{19}F NMR (376 MHz, $CDCl_3$) 2-(difluoromethyl)dibenzofuran 4ah

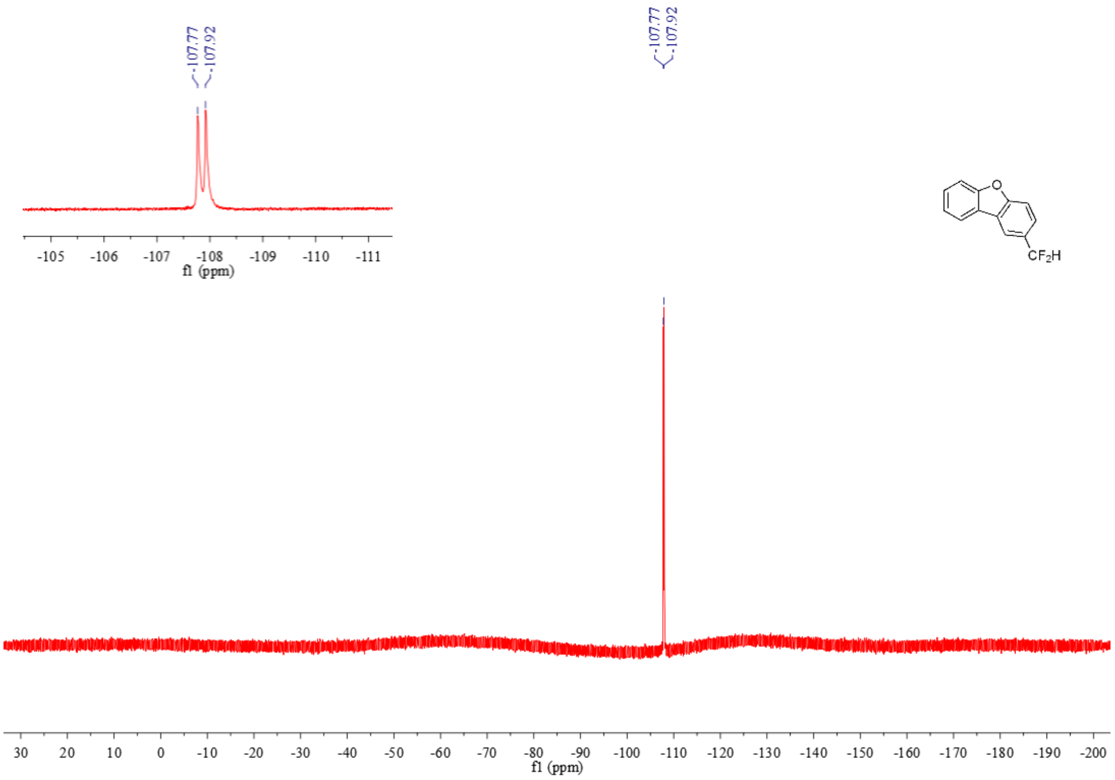

^{13}C NMR (101 MHz, $CDCl_3$) 2-(difluoromethyl)dibenzofuran 4ah

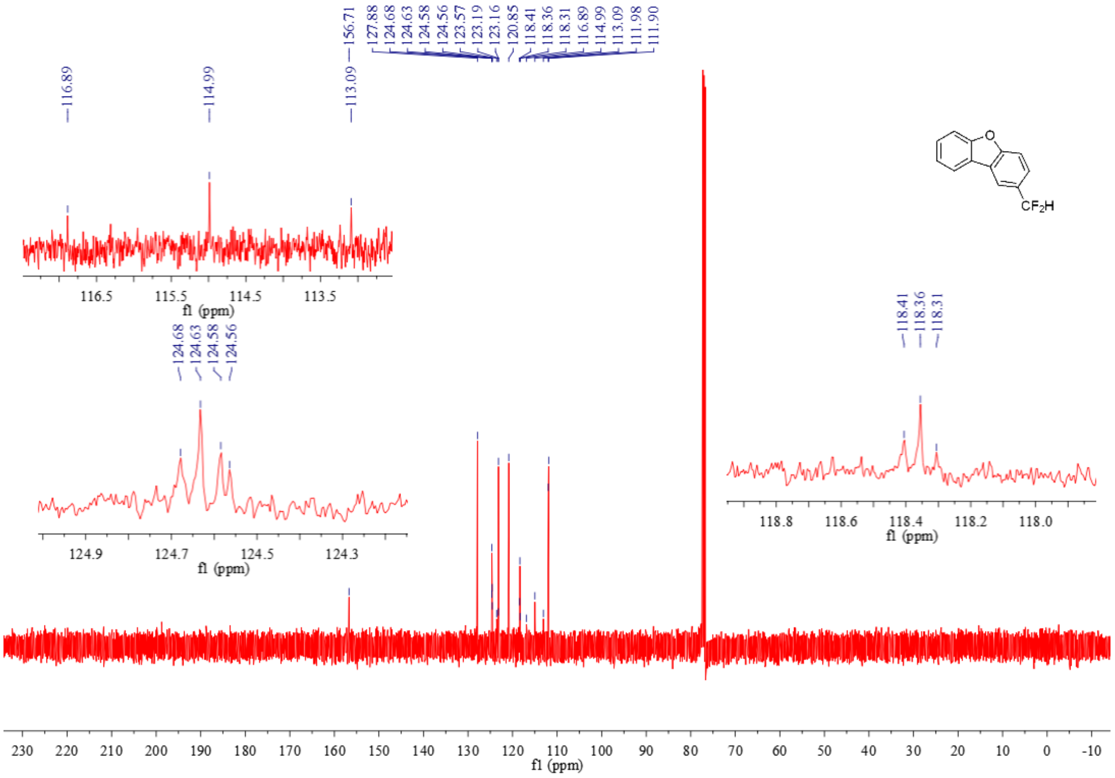

^{1}H NMR (400 MHz, $CDCl_3$) ethyl 5-(difluoromethyl)benzofuran -2-carboxylate 4ai

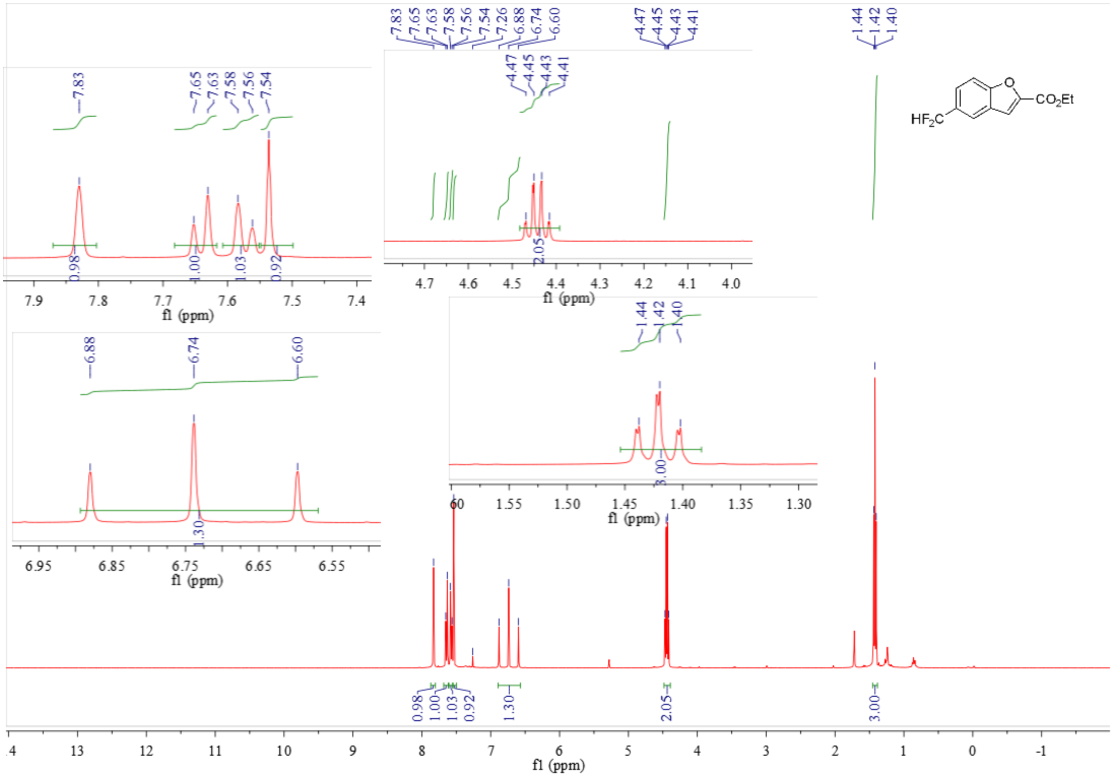

^{19}F NMR (376 MHz, $CDCl_3$) ethyl 5-(difluoromethyl)benzofuran -2-carboxylate 4ai

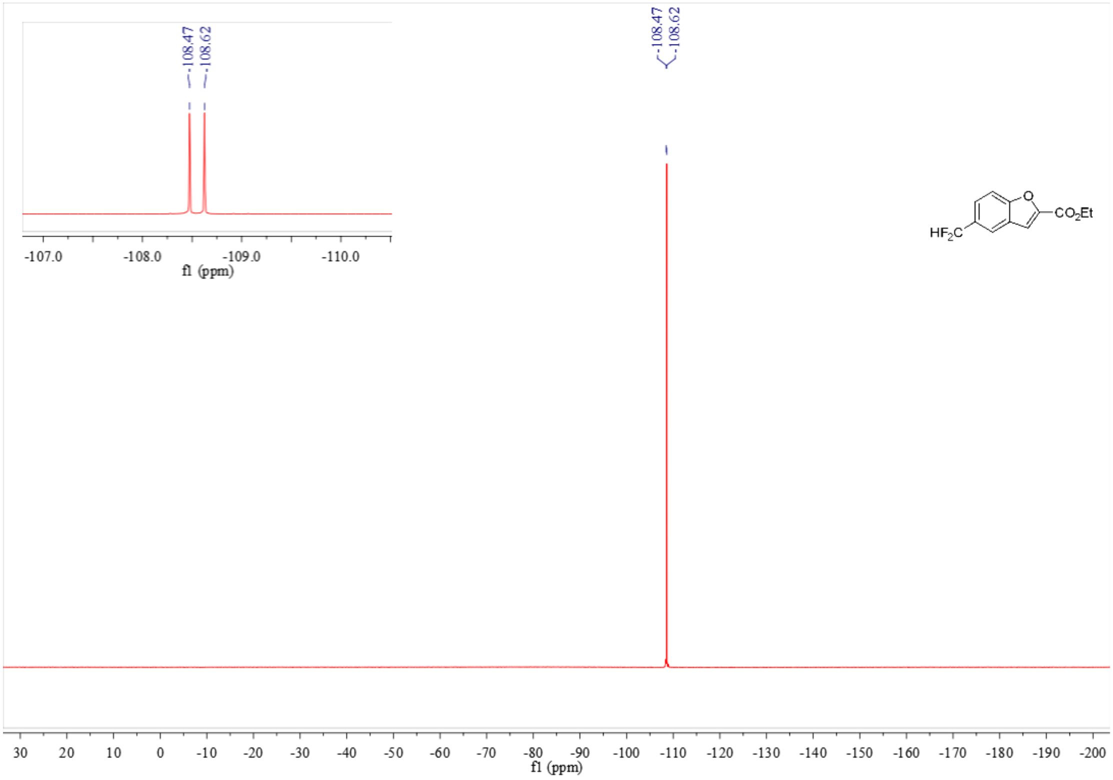

^{13}C NMR (101 MHz, $CDCl_3$) ethyl 5-(difluoromethyl)benzofuran -2-carboxylate 4ai

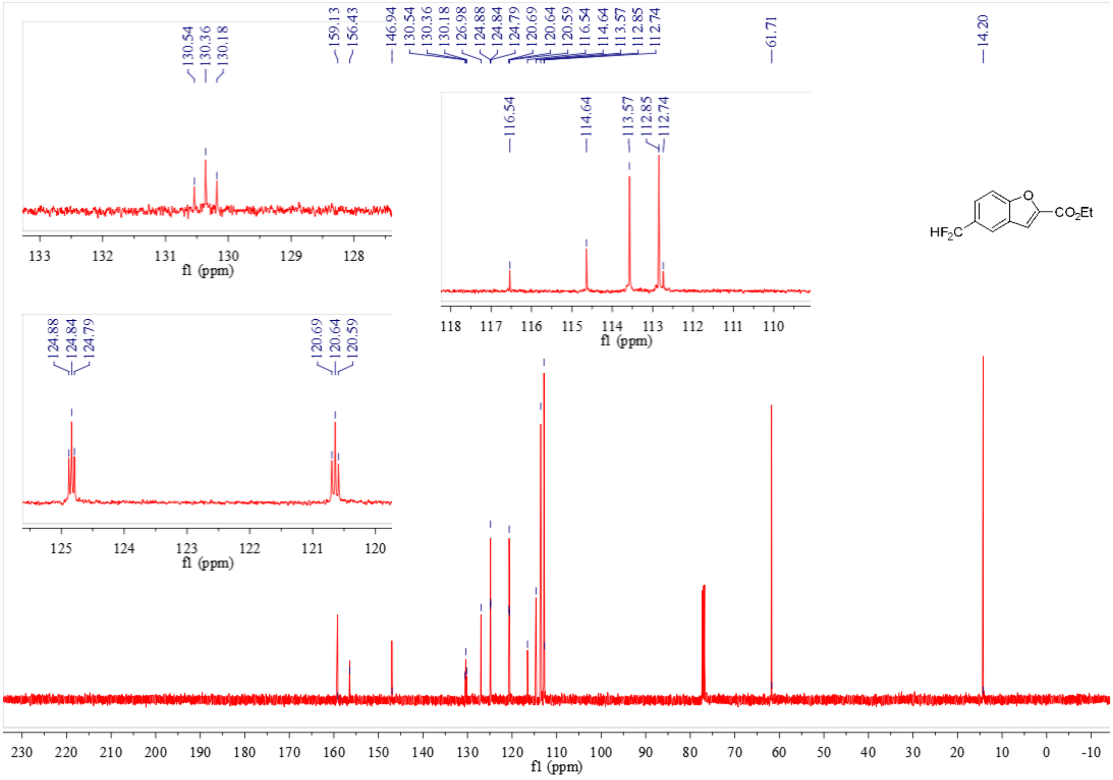

^{1}H NMR (400 MHz, $CDCl_3$) 1-[5-(difluoromethyl)benzofuran-2-yl]ethanone 4aj

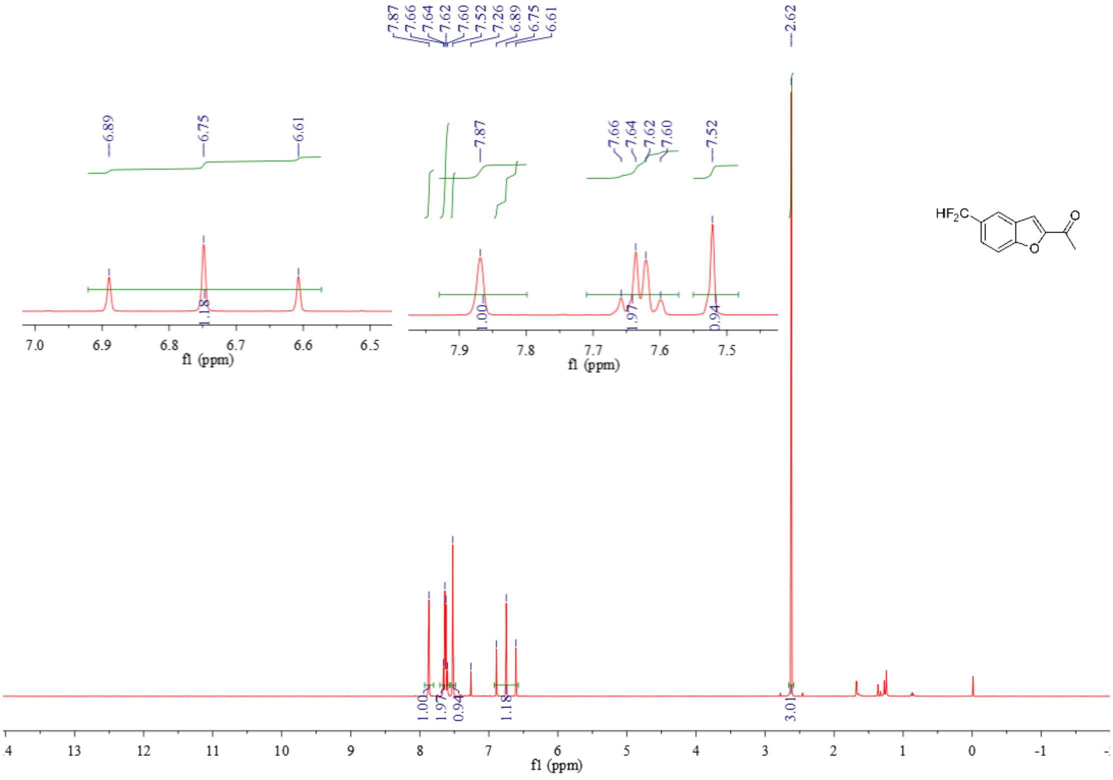

^{19}F NMR (376 MHz, $CDCl_3$) 1-[5-(difluoromethyl)benzofuran-2-yl]ethanone 4aj

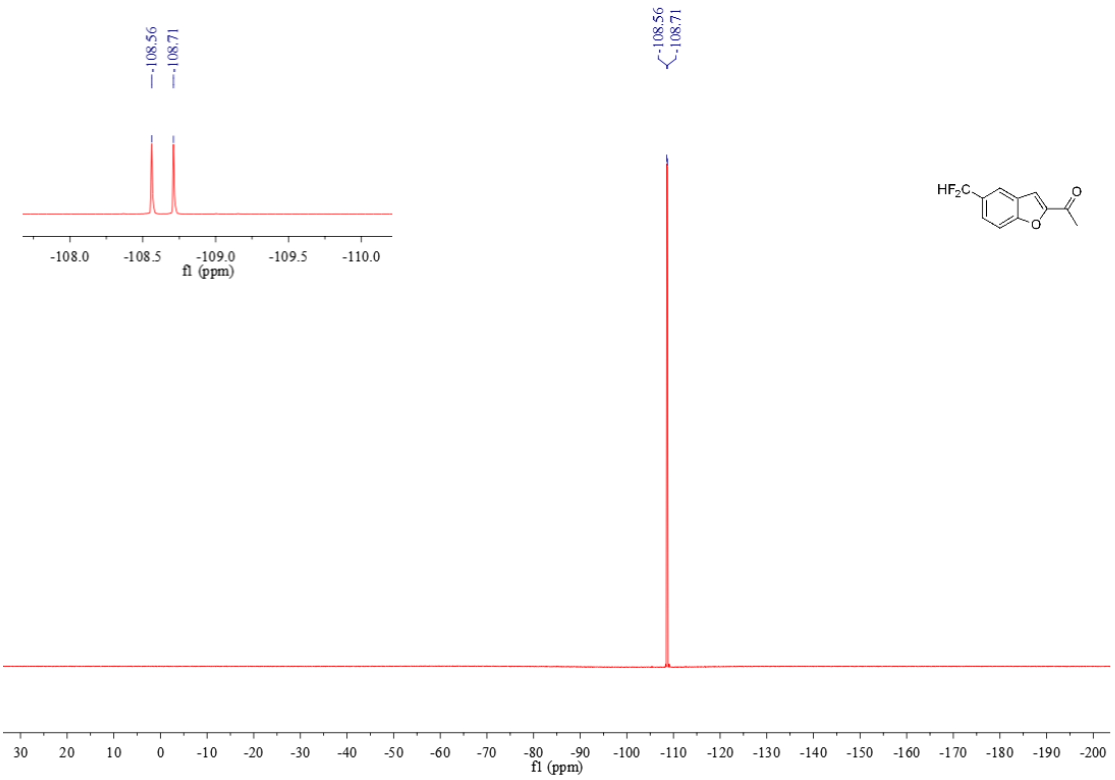

^{13}C NMR (101 MHz, $CDCl_3$) 1-[5-(difluoromethyl)benzofuran-2-yl]ethanone 4aj

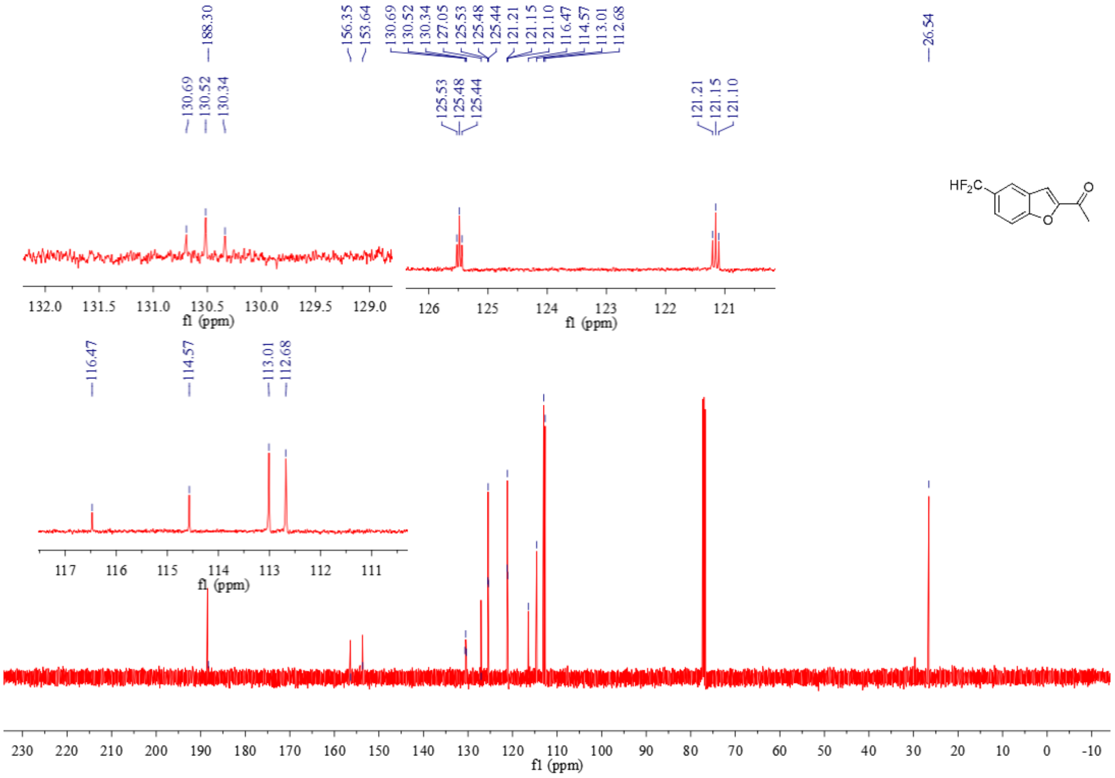

^{1}H NMR (400 MHz, $CDCl_3$) 5-(difluoromethyl)benzothiophene 4ak

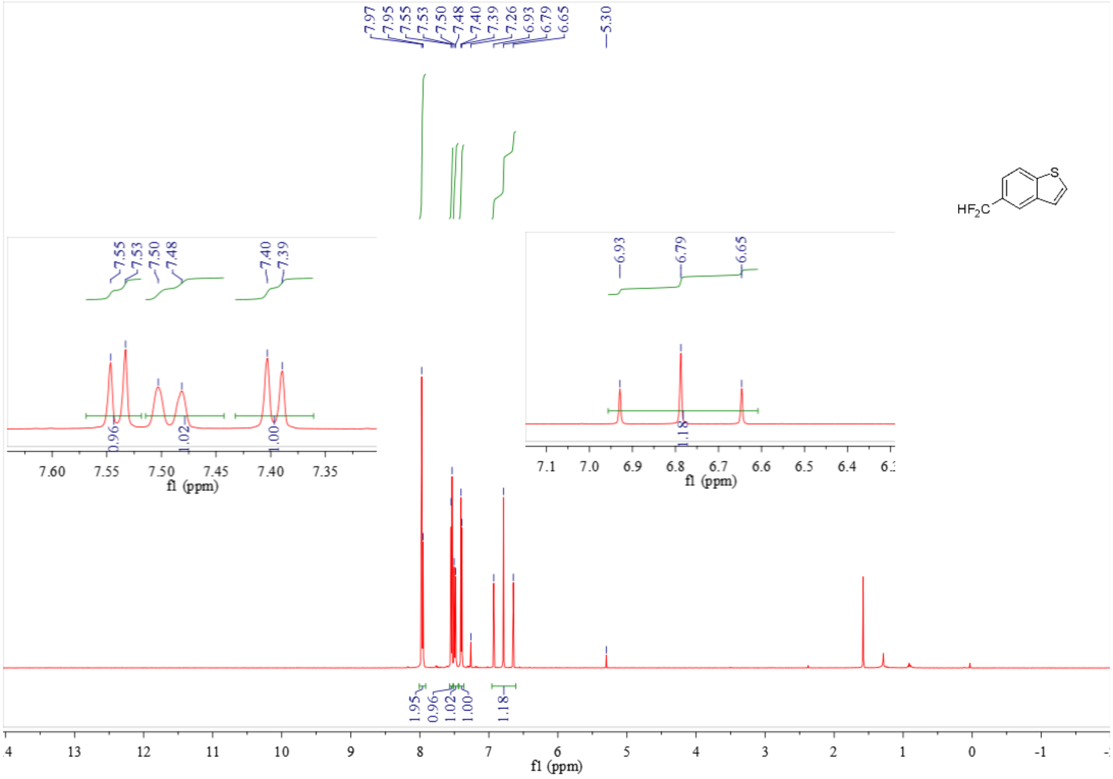

^{19}F NMR (376 MHz, $CDCl_3$) 5-(difluoromethyl)benzothiophene 4ak

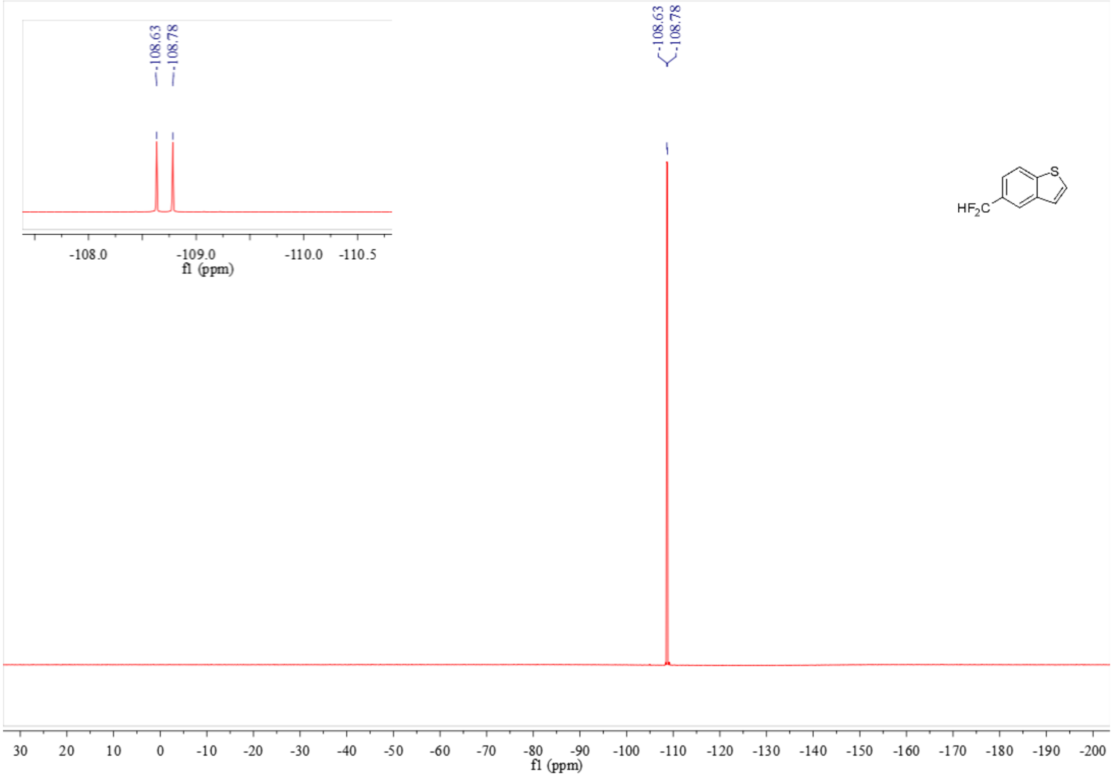

^{13}C NMR (101 MHz, $CDCl_3$) 5-(difluoromethyl)benzothiophene 4ak

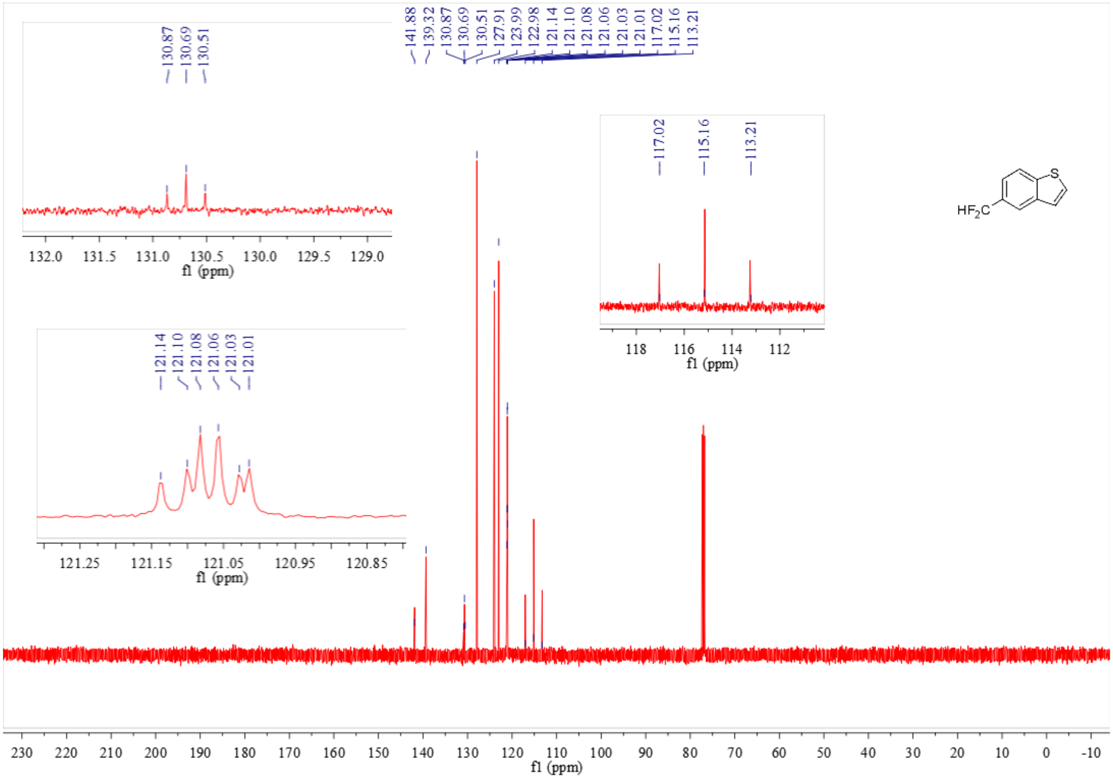

^{1}H NMR (400 MHz, $CDCl_3$) 5-(difluoromethyl)-2-ethyl-1,3-benzoxazole 4al

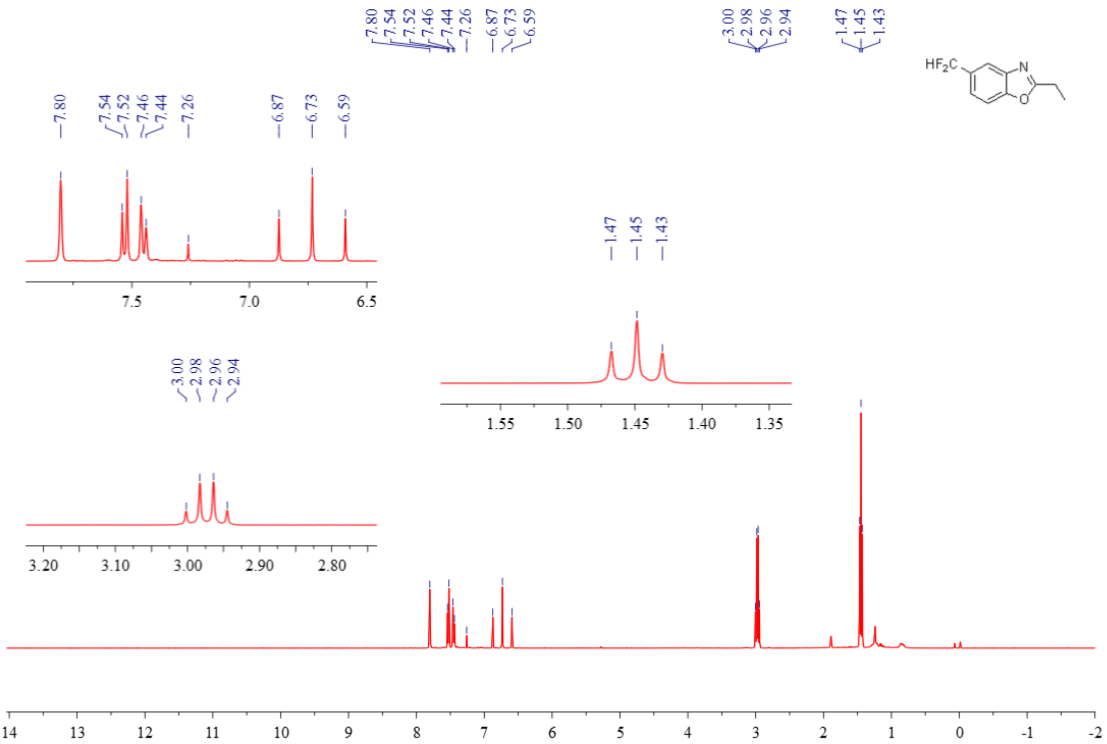

^{19}F NMR (376 MHz, $CDCl_3$) 5-(difluoromethyl)-2-ethyl-1,3-benzoxazole 4al

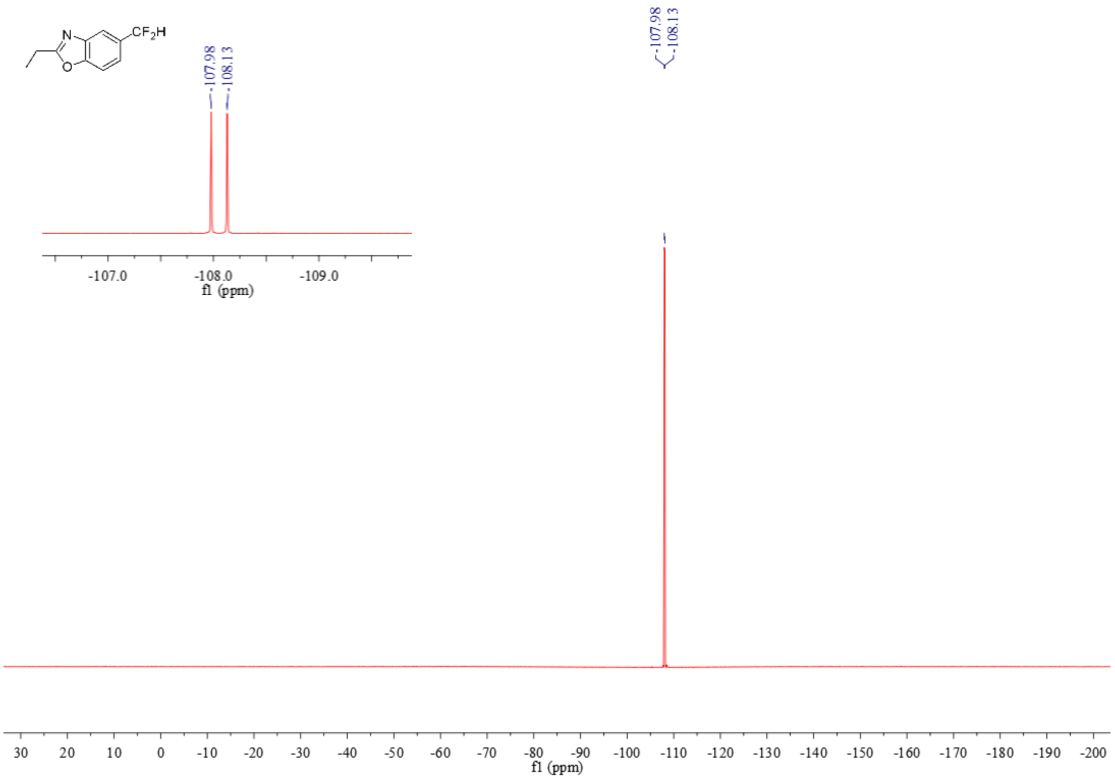

^{13}C NMR (101 MHz, $CDCl_3$) 5-(difluoromethyl)-2-ethyl-1,3-benzoxazole 4al

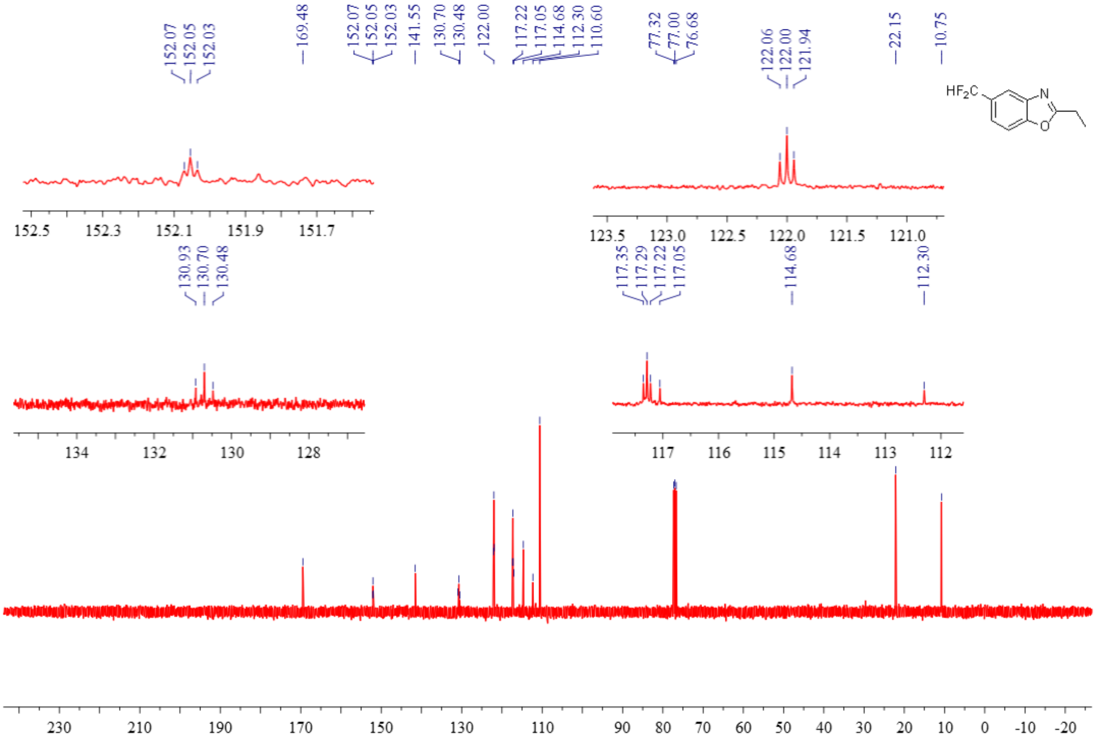

^{1}H NMR (400 MHz, $CDCl_3$) 5-(difluoromethyl)-2-methyl-1,3-benzothiazole 4am

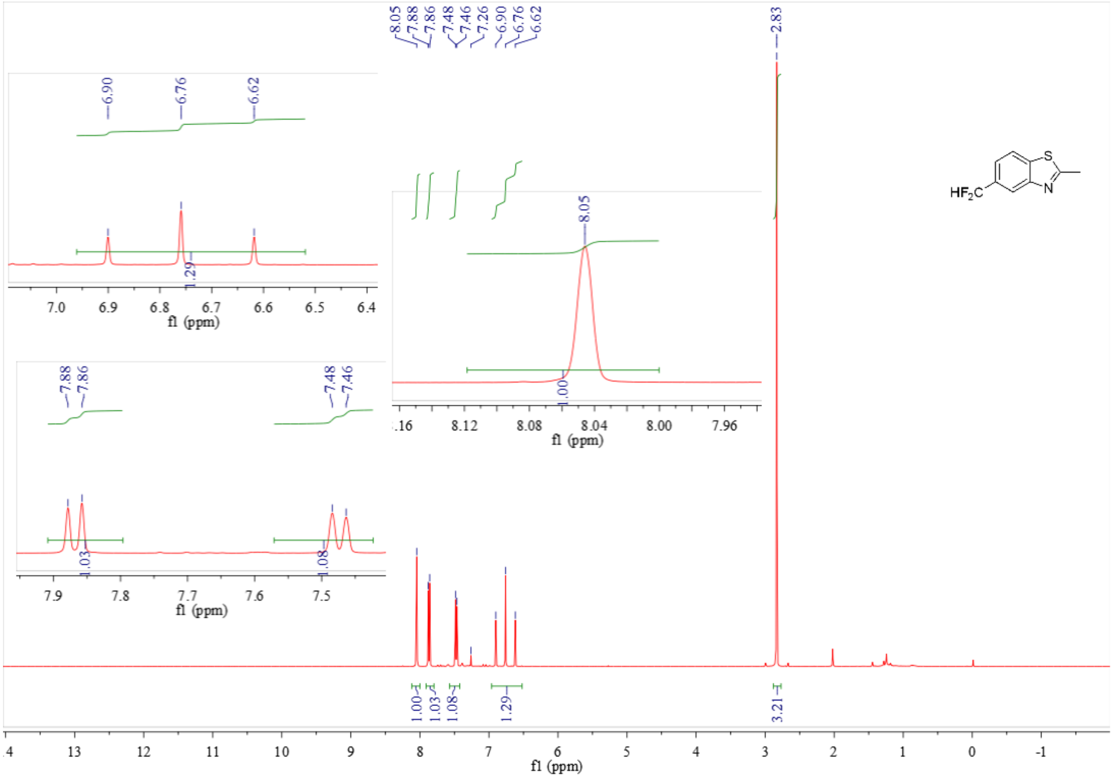

^{19}F NMR (376 MHz, $CDCl_3$) 5-(difluoromethyl)-2-methyl-1,3-benzothiazole 4am

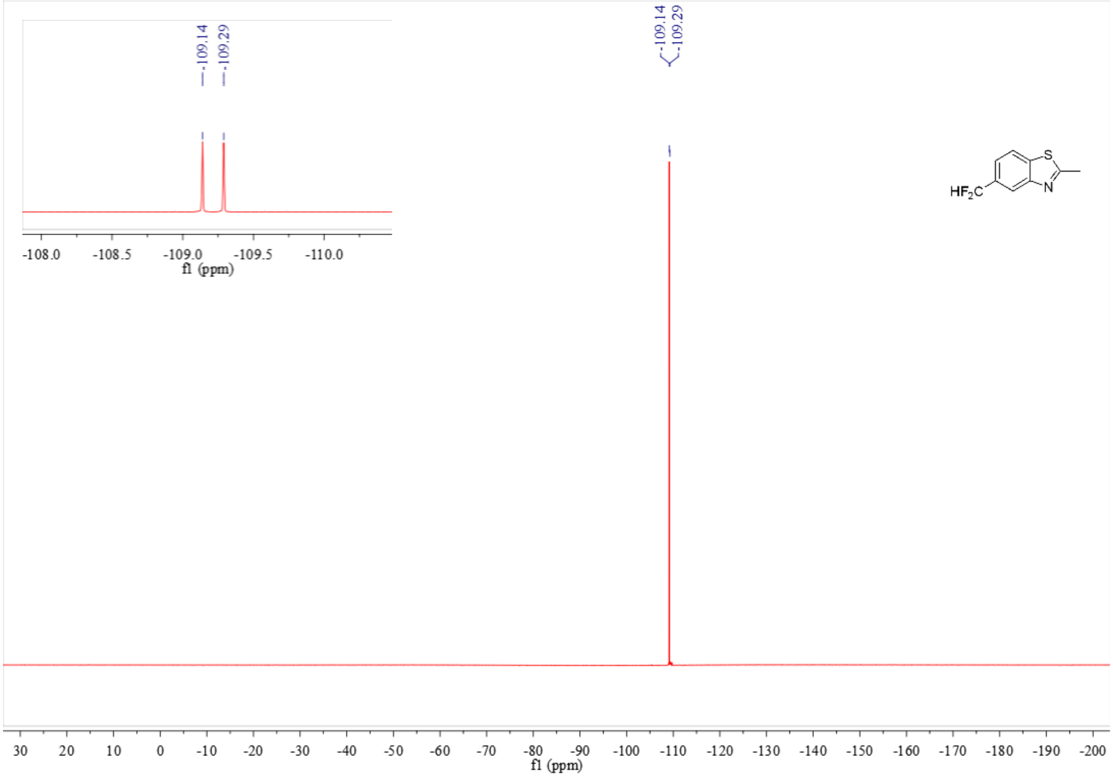

^{13}C NMR (101 MHz, $CDCl_3$) 5-(difluoromethyl)-2-methyl-1,3-benzothiazole 4am

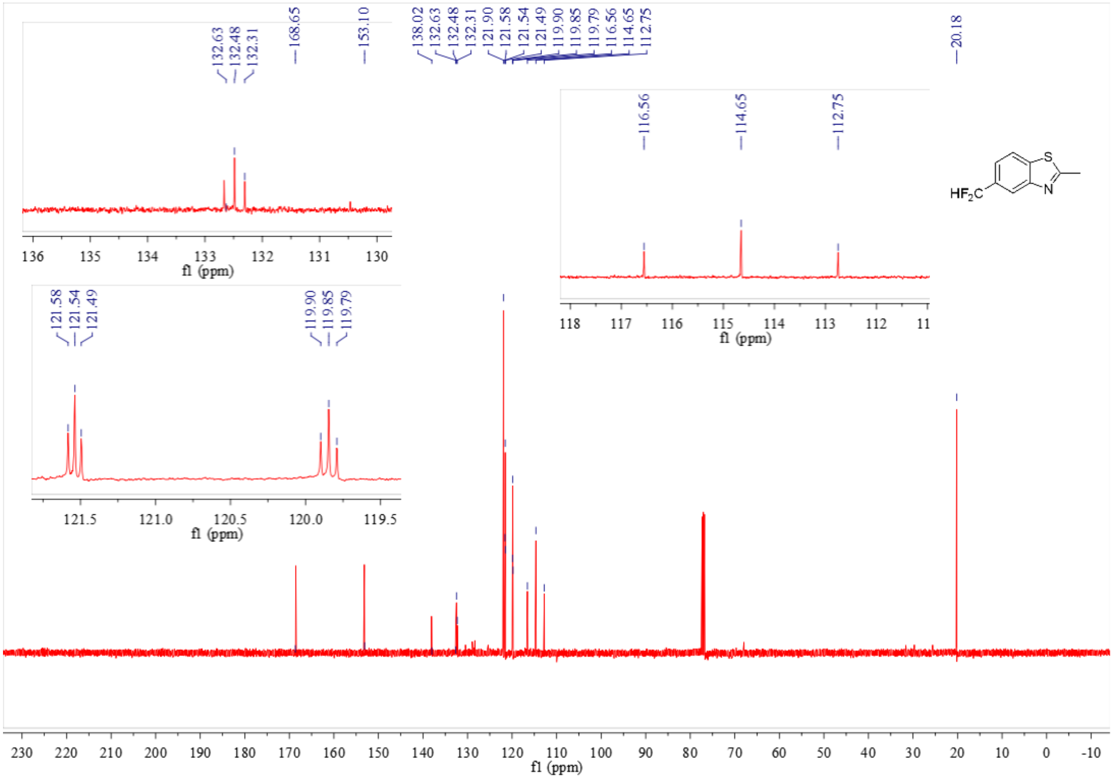

^{1}H NMR (400 MHz, $CDCl_3$) 1-benzyl-4-(difluoromethyl)indole 4an

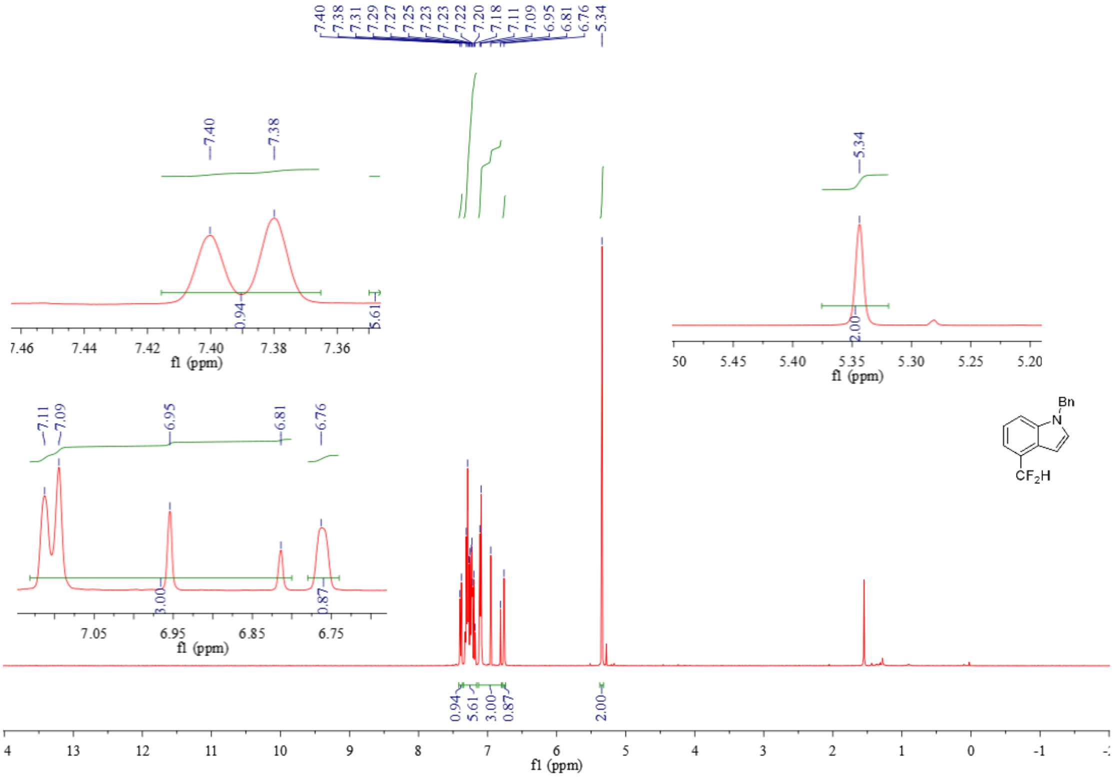

^{19}F NMR (376 MHz, $CDCl_3$) 1-benzyl-4-(difluoromethyl)indole 4an

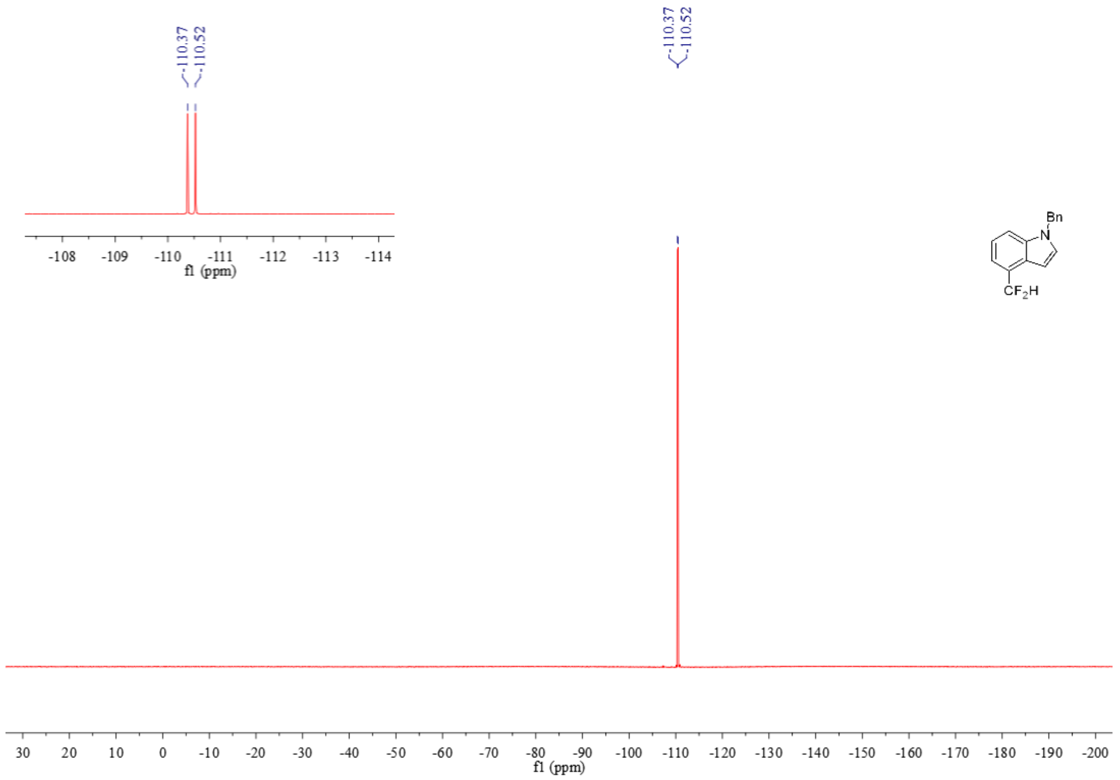

^{13}C NMR (101 MHz, $CDCl_3$) 1-benzyl-4-(difluoromethyl)indole 4an

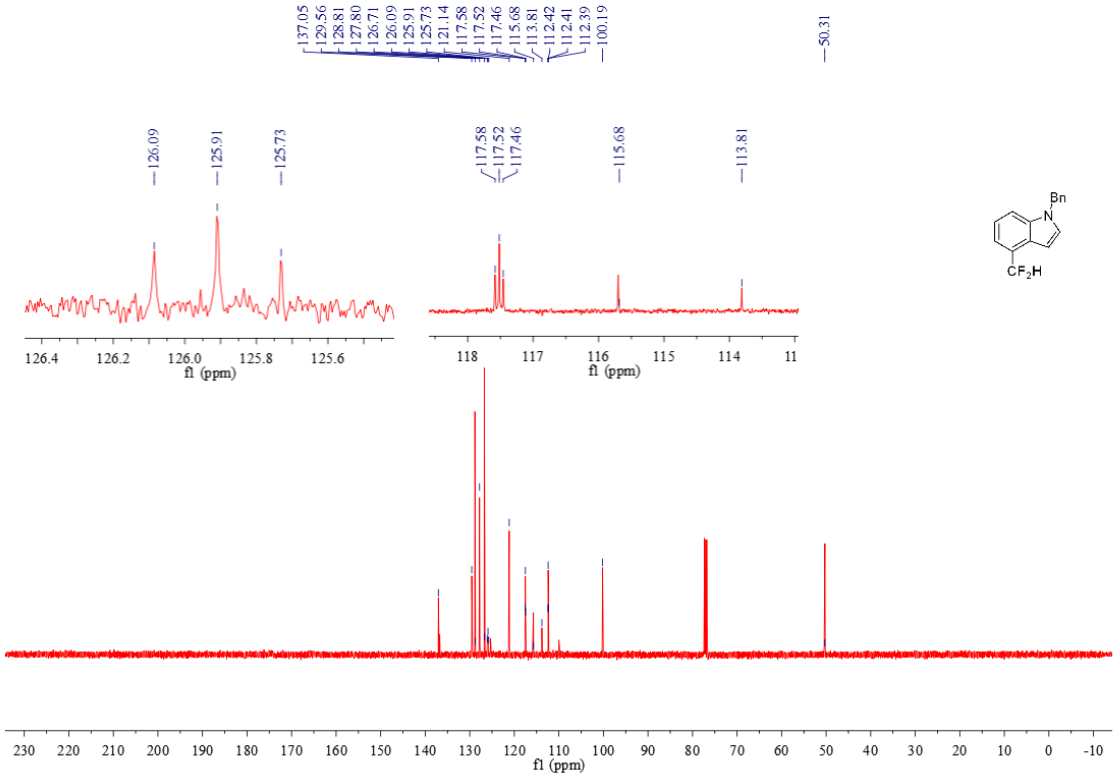

^{1}H NMR (400 MHz, $CDCl_3$) 1-benzyl-6-(difluoromethyl)indazole 4ao

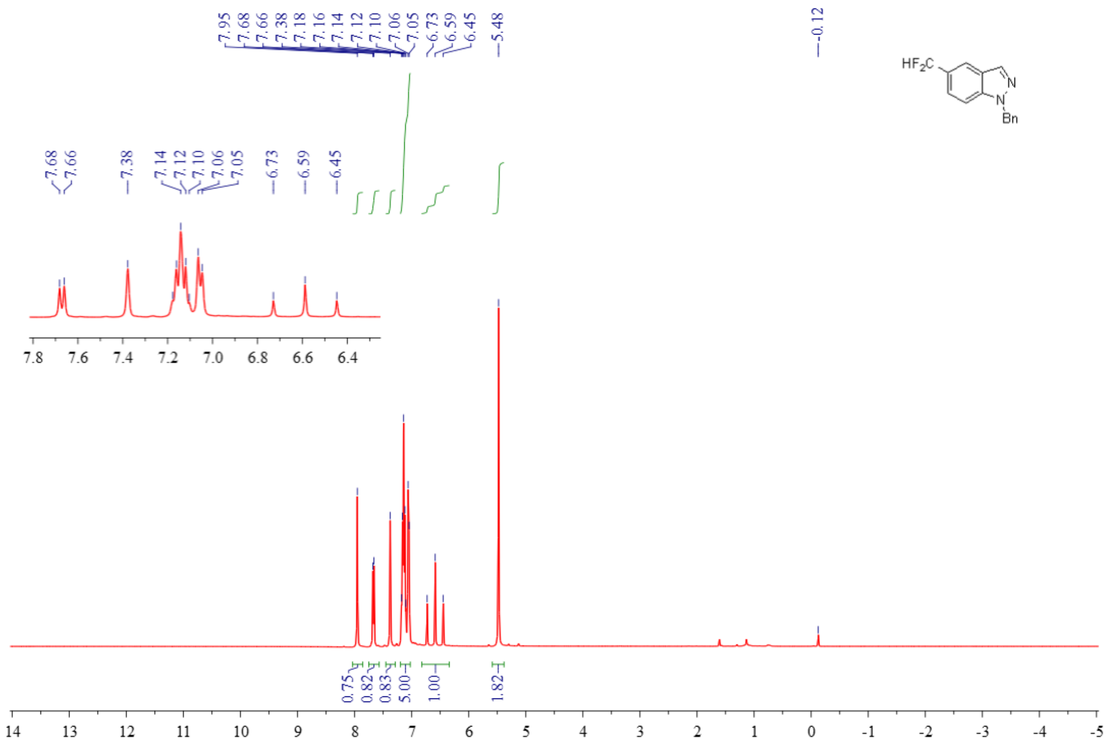

^{19}F NMR (376 MHz, $CDCl_3$) 1-benzyl-6-(difluoromethyl)indazole 4ao

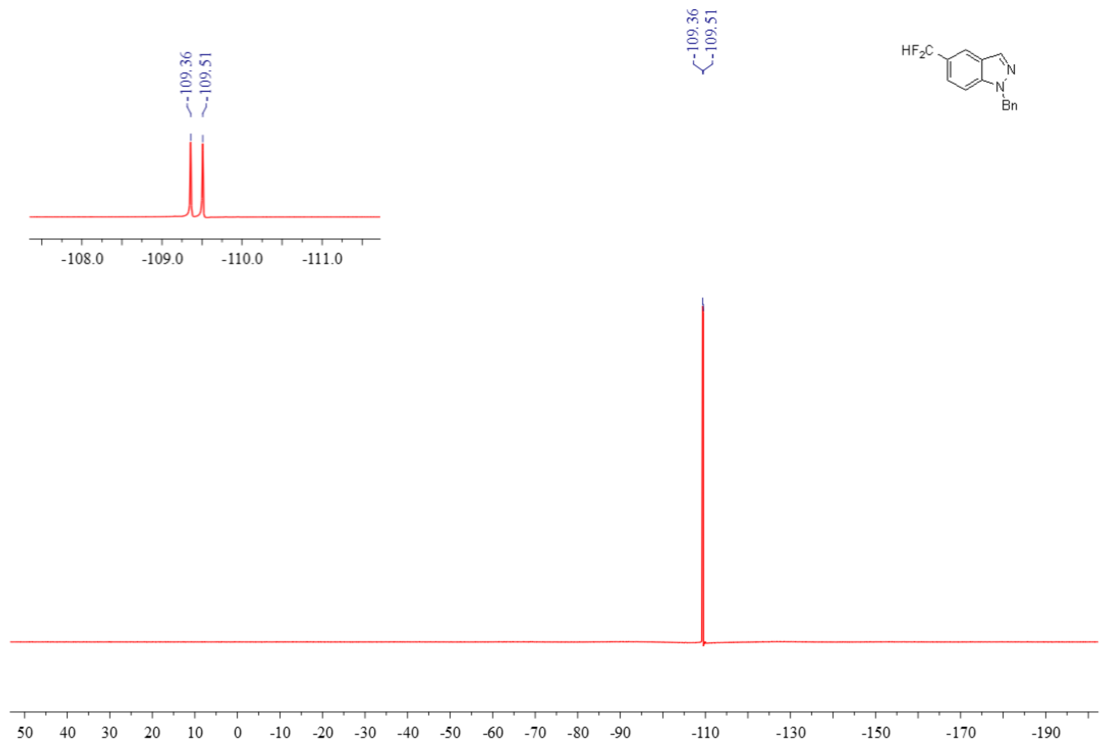

^{13}C NMR (101 MHz, $CDCl_3$) 1-benzyl-6-(difluoromethyl)indazole 4ao

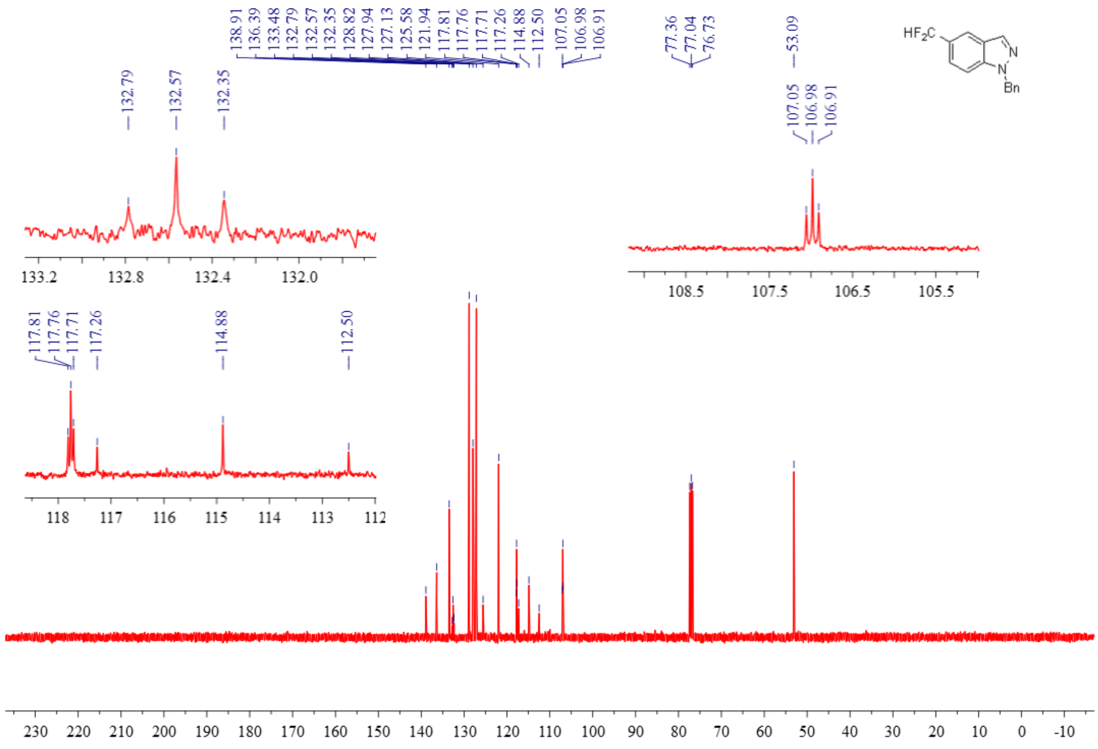

^{1}H NMR (400 MHz, $CDCl_3$) 3-(difluoromethyl)-9-phenyl-carbazole 4ap

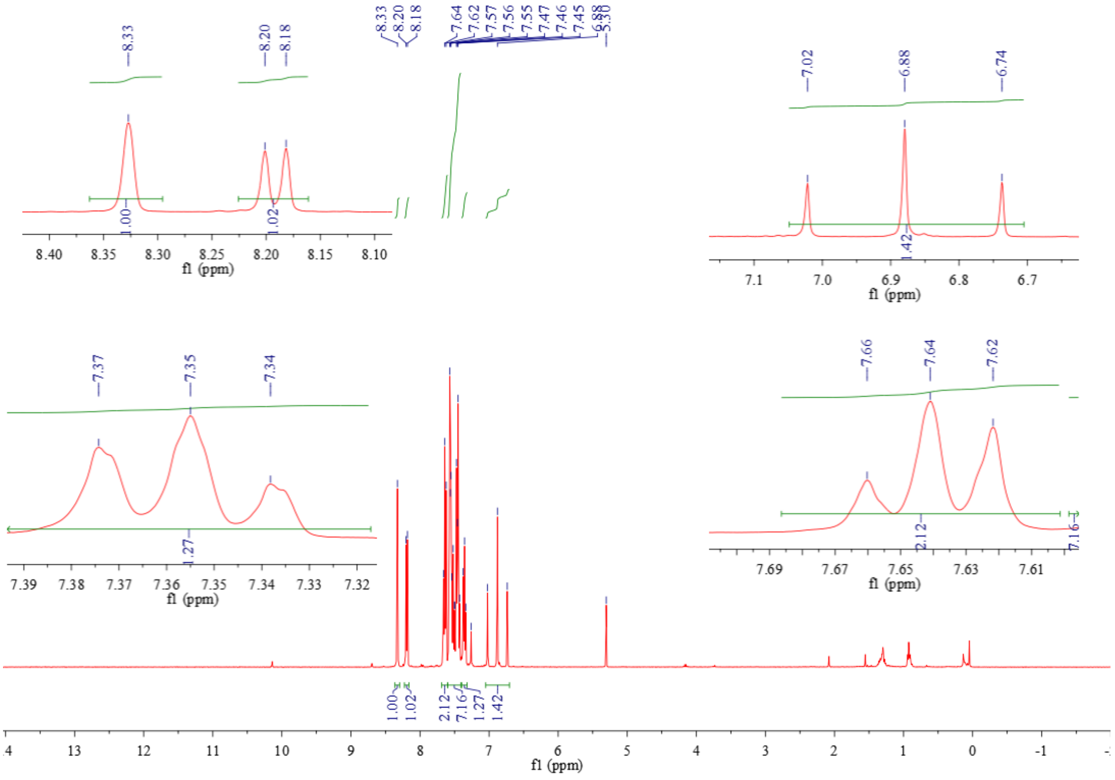

^{19}F NMR (376 MHz, $CDCl_3$) 3-(difluoromethyl)-9-phenyl-carbazole 4ap

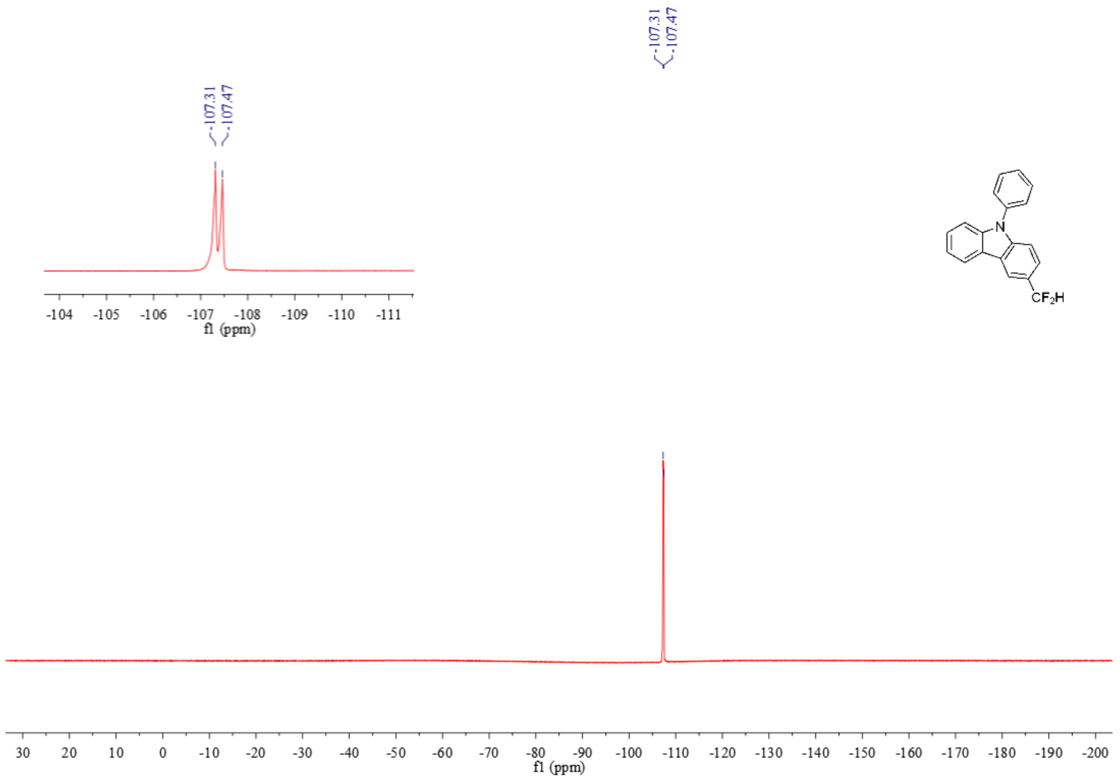

^{13}C NMR (101 MHz, $CDCl_3$) 3-(difluoromethyl)-9-phenyl-carbazole 4ap

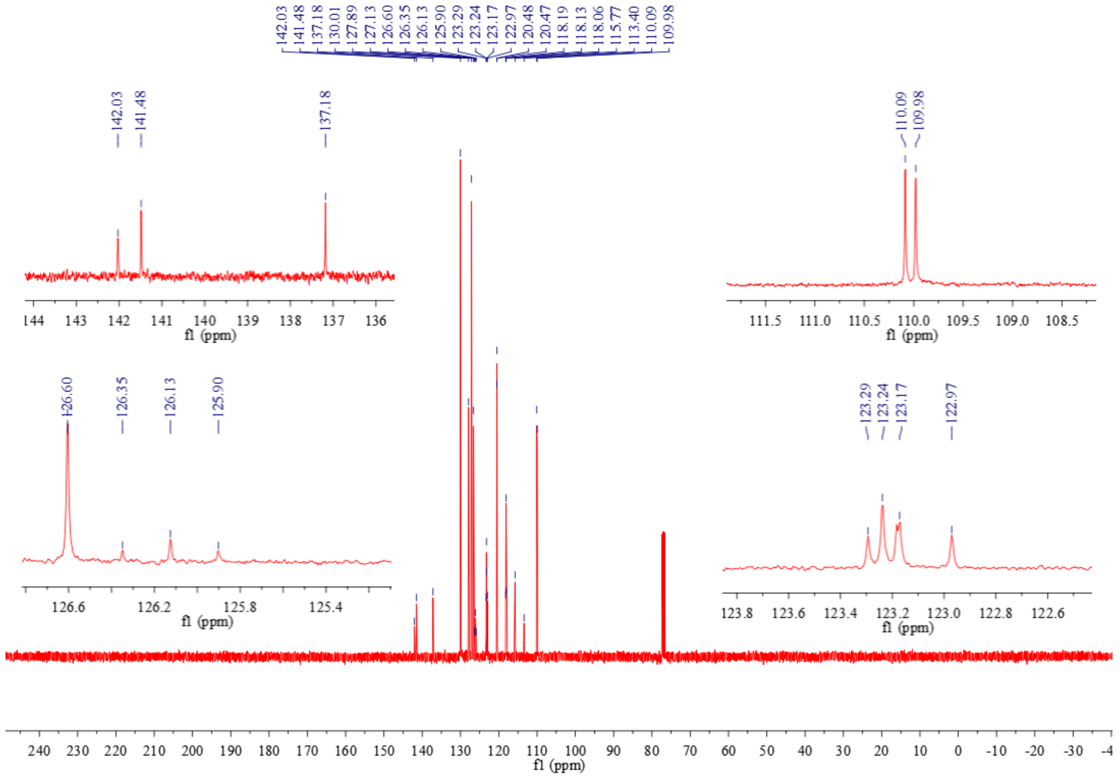

^{1}H NMR (400 MHz, $CDCl_3$) 5-(difluoromethyl)-2,4-dimethoxy-pyrimidine 5a

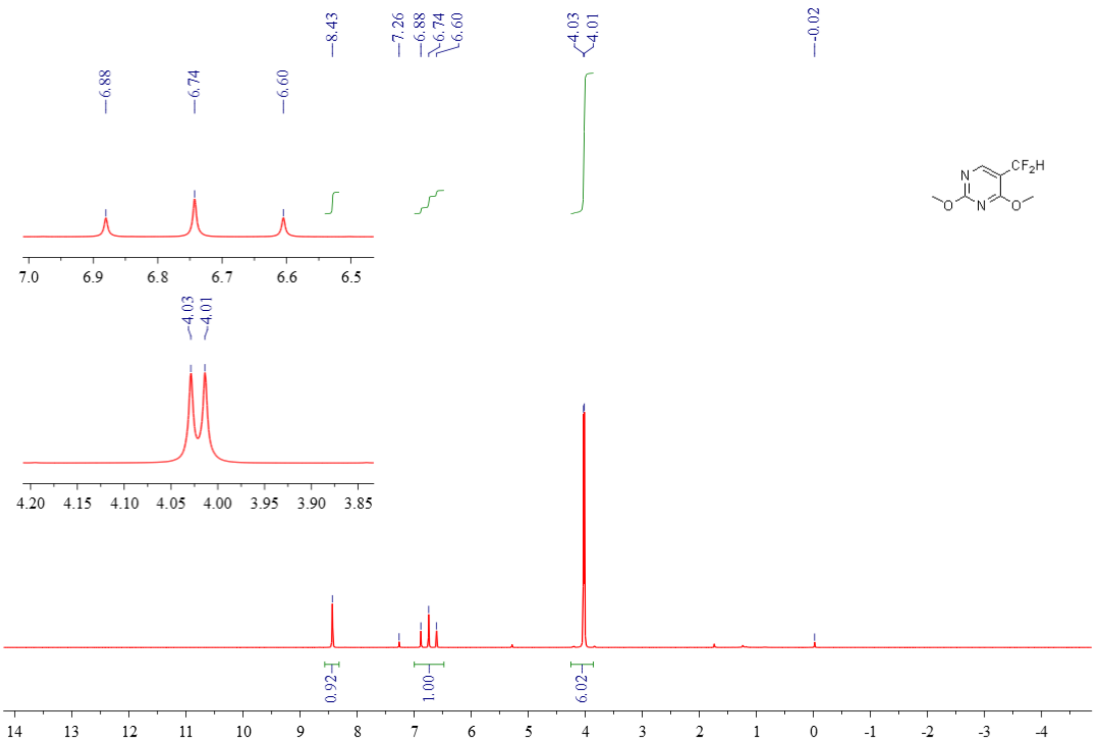

^{19}F NMR (376 MHz, $CDCl_3$) 5-(difluoromethyl)-2,4-dimethoxy-pyrimidine 5a

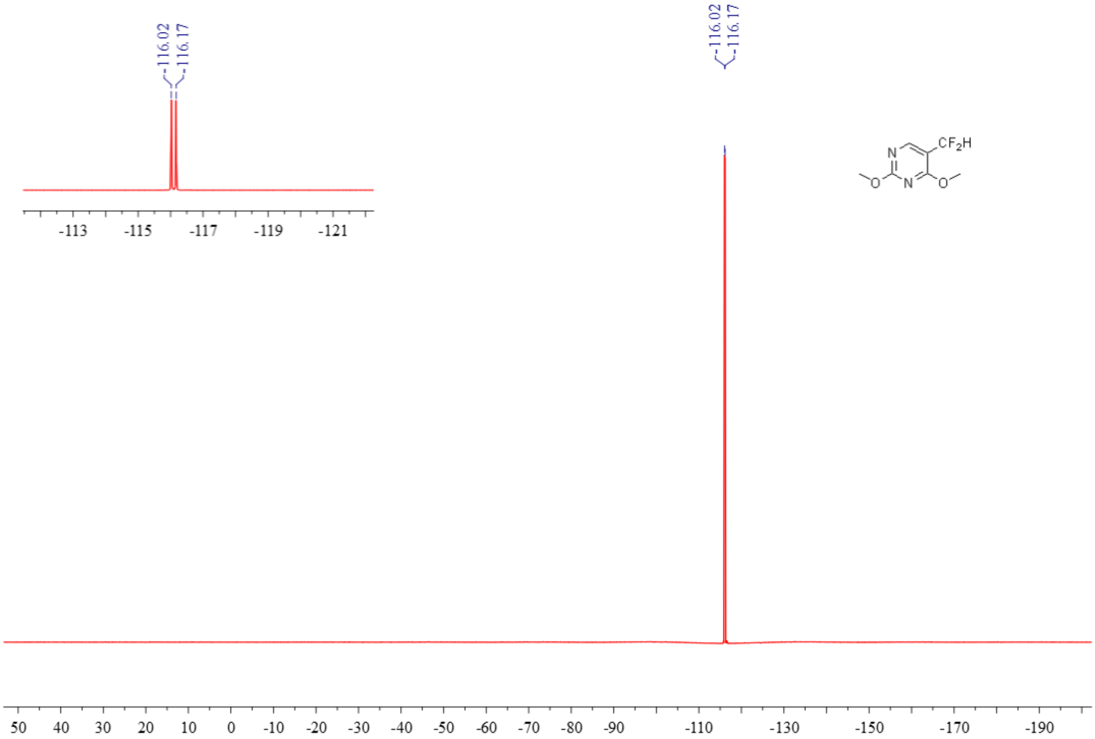

^{13}C NMR (101 MHz, $CDCl_3$) 5-(difluoromethyl)-2,4-dimethoxy-pyrimidine 5a

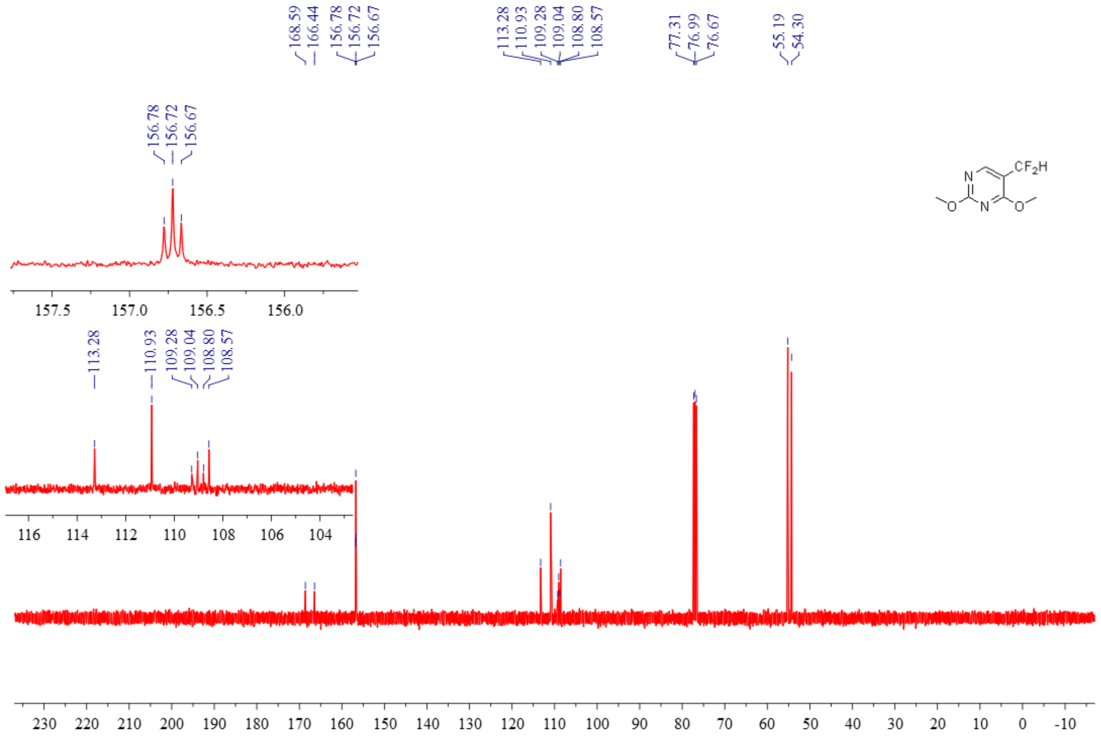

^{1}H NMR (400 MHz, $CDCl_3$) 8-benzyloxy-5,7-dichloro -3-(difluoromethyl) quinolone 5b

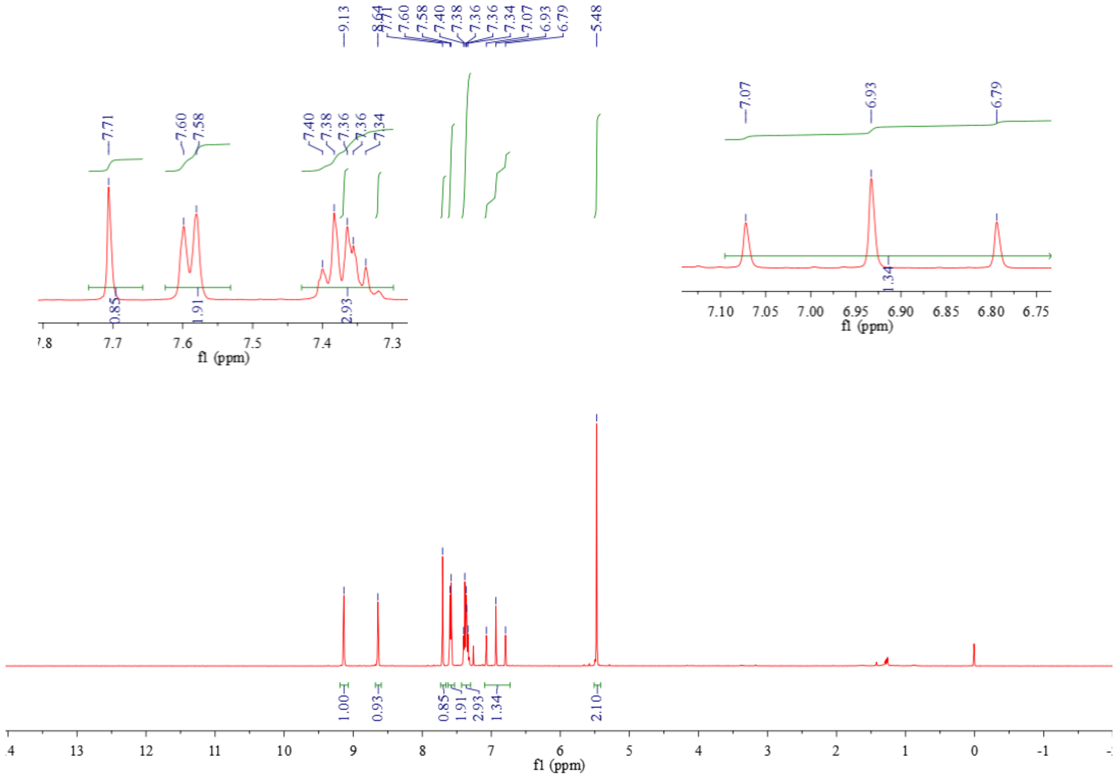

^{19}F NMR (376 MHz, CDCl$_3$) 8-benzyloxy-5,7-dichloro -3-(difluoromethyl) quinolone 5b

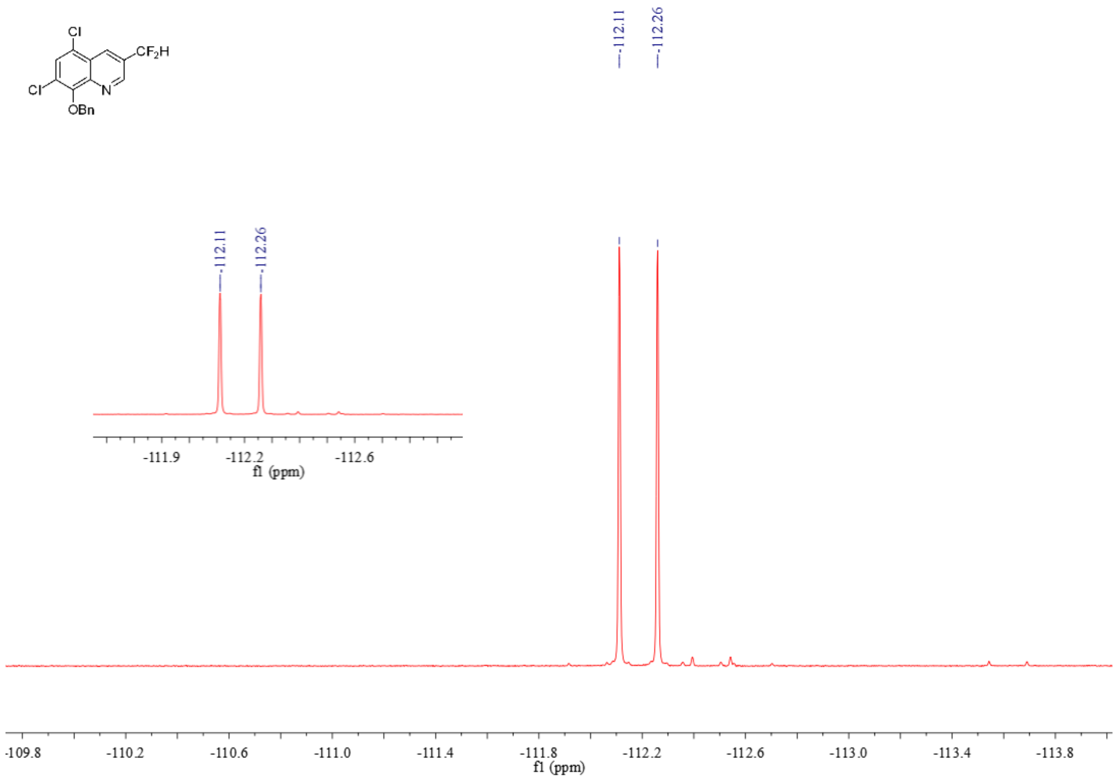

^{13}C NMR (101 MHz, CDCl$_3$) 8-benzyloxy-5,7-dichloro -3-(difluoromethyl) quinolone 5b

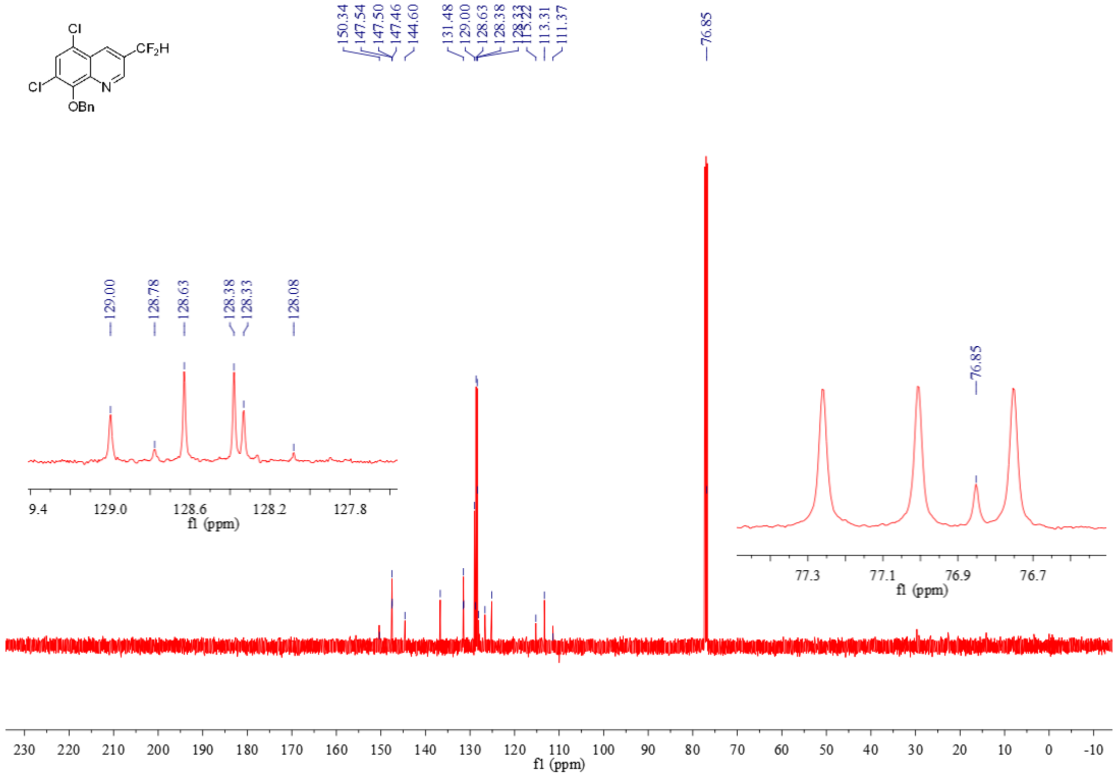

^{1}H NMR (400 MHz, $CDCl_3$) 4-iodo-1-(4-methoxyphenyl)-1*H*-pyrazole

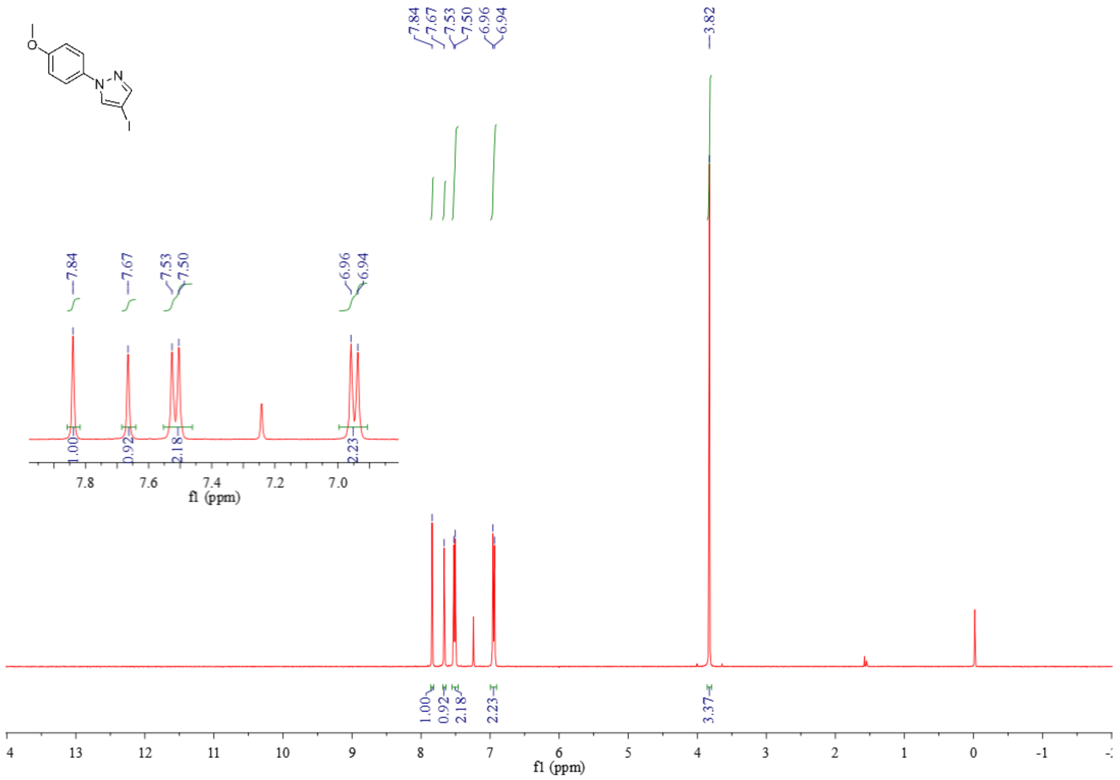

^{13}C NMR (101 MHz, $CDCl_3$) 4-iodo-1-(4-methoxyphenyl)-1*H*-pyrazole

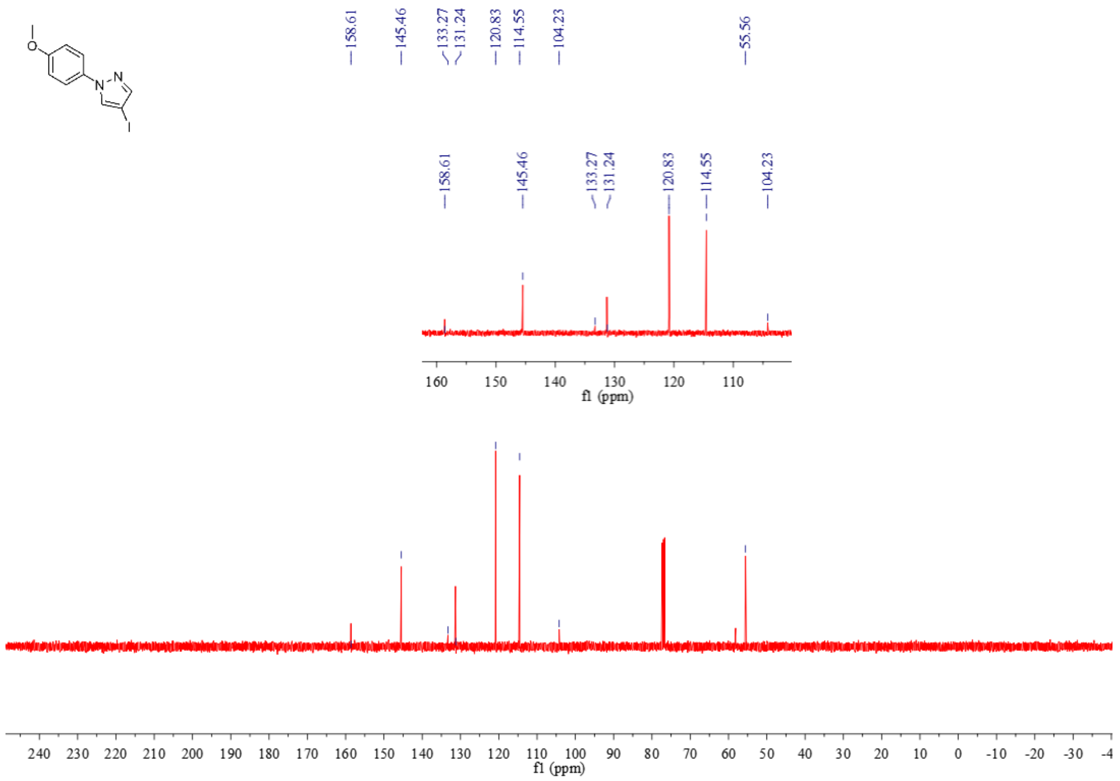

^{1}H NMR (400 MHz, $CDCl_3$)

4-(difluoromethyl)-1-(4-methoxyphenyl)-1*H*-pyrazole 4aq

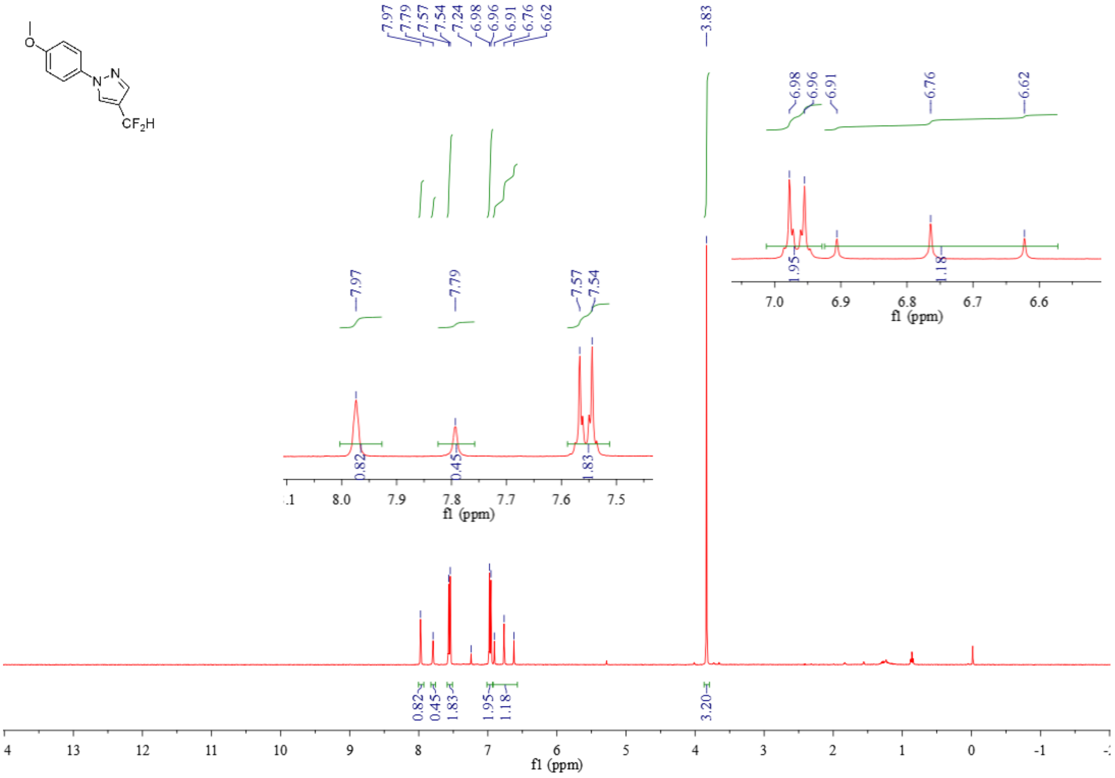

^{19}F NMR (376 MHz, $CDCl_3$)

4-(difluoromethyl)-1-(4-methoxyphenyl)-1*H*-pyrazole 5d

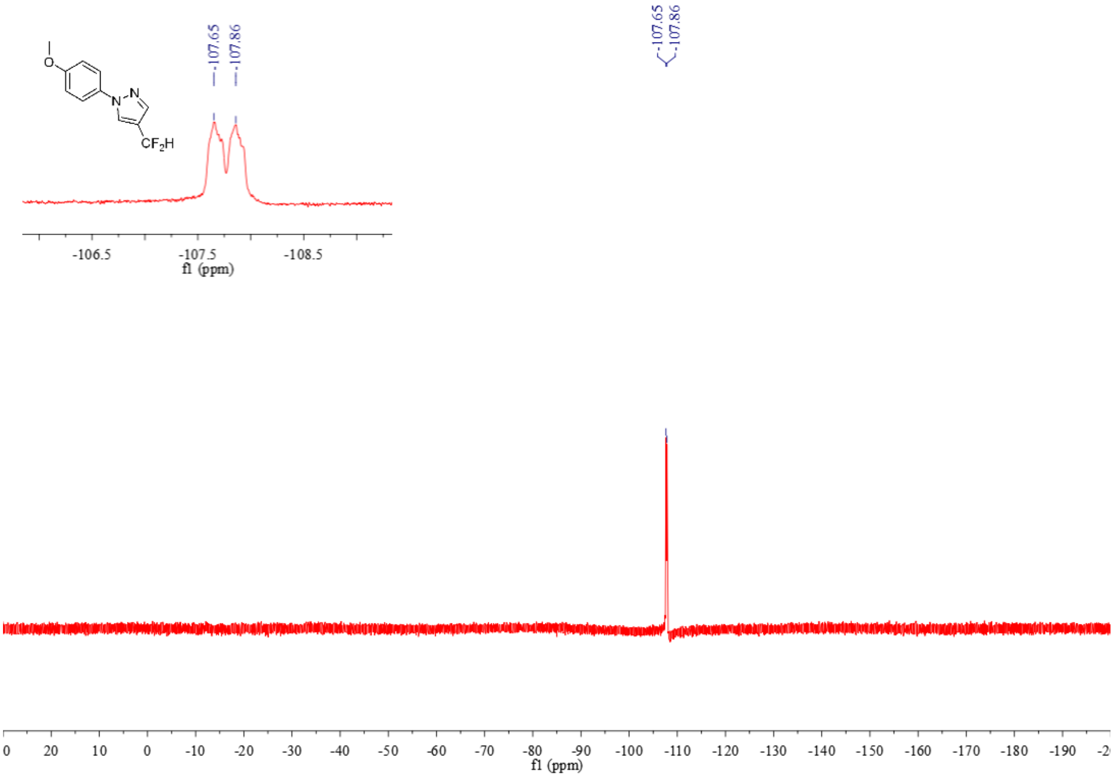

^{13}C NMR (101 MHz, $CDCl_3$)
4-(difluoromethyl)-1-(4-methoxyphenyl)-1*H*-pyrazole 5d

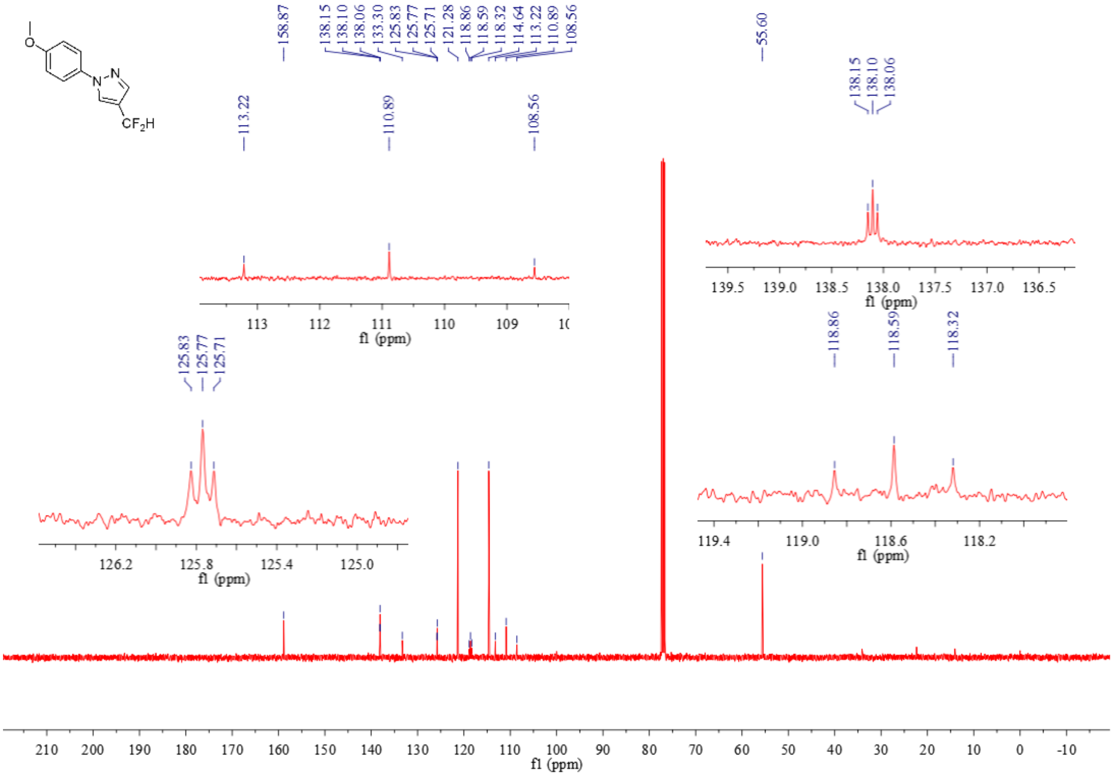

^{1}H NMR (400 MHz, $CDCl_3$) 2-(difluoromethyl)-4-phenyl-pyridine 5e

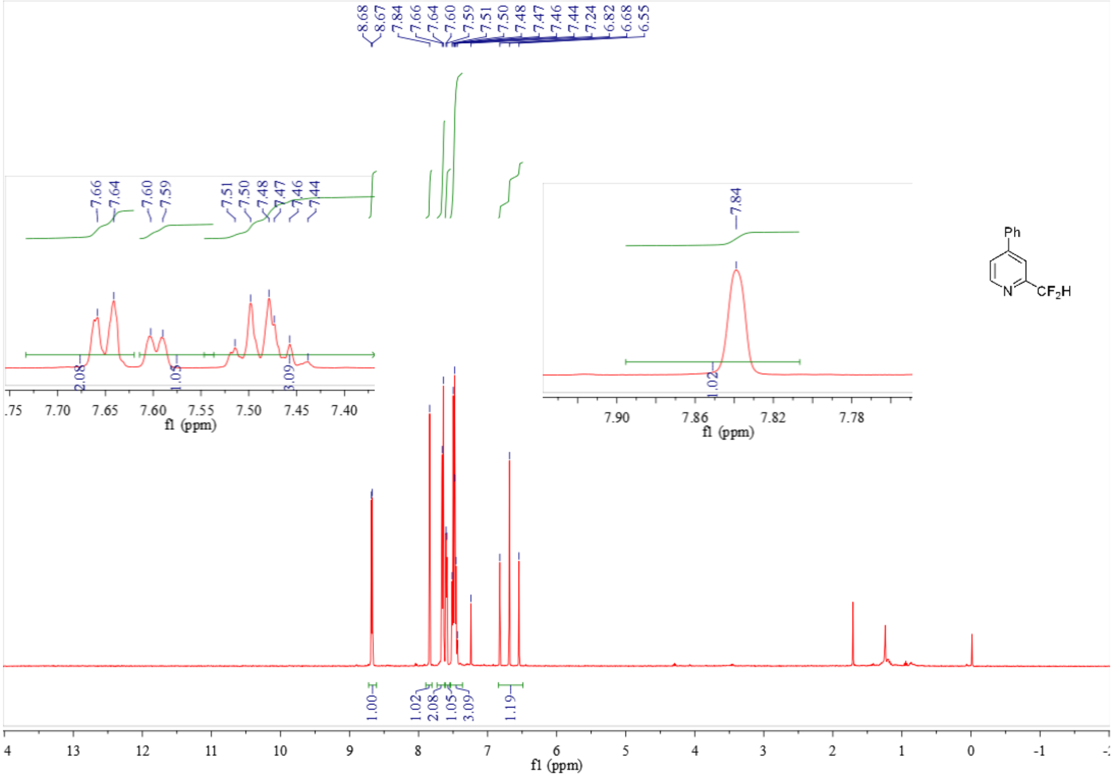

^{19}F NMR (376 MHz, $CDCl_3$) 2-(difluoromethyl)-4-phenyl-pyridine 5e

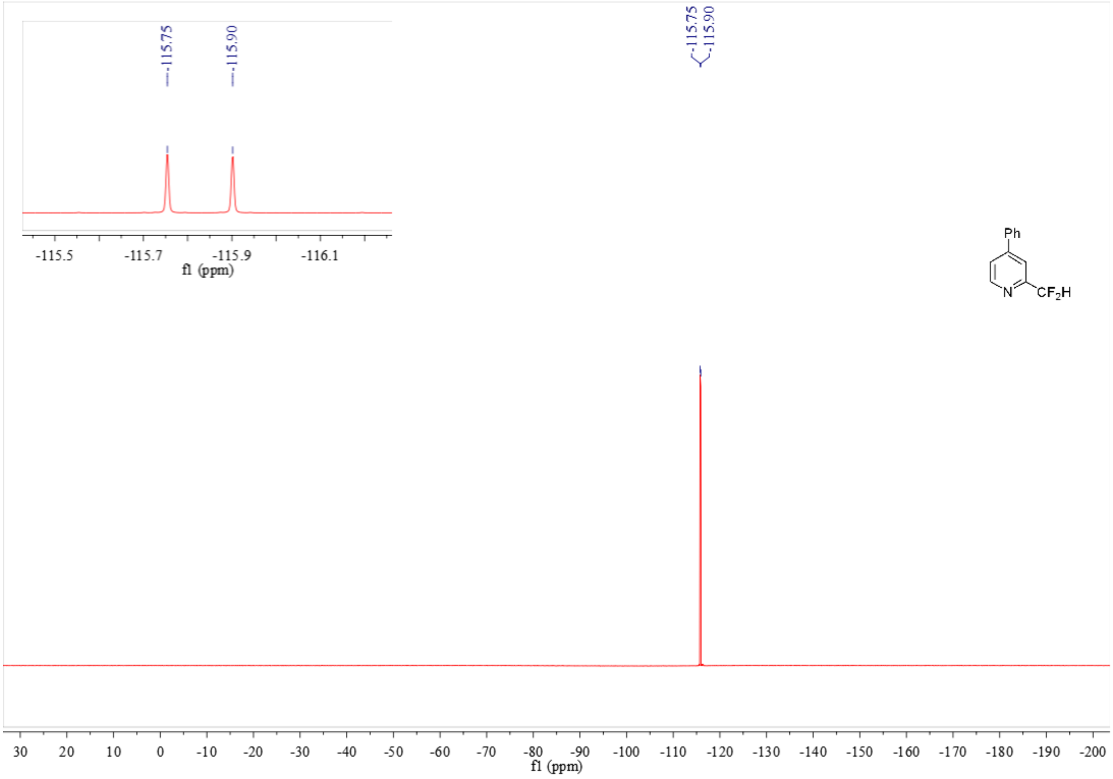

^{13}C NMR (101 MHz, $CDCl_3$) 2-(difluoromethyl)-4-phenyl-pyridine 5e

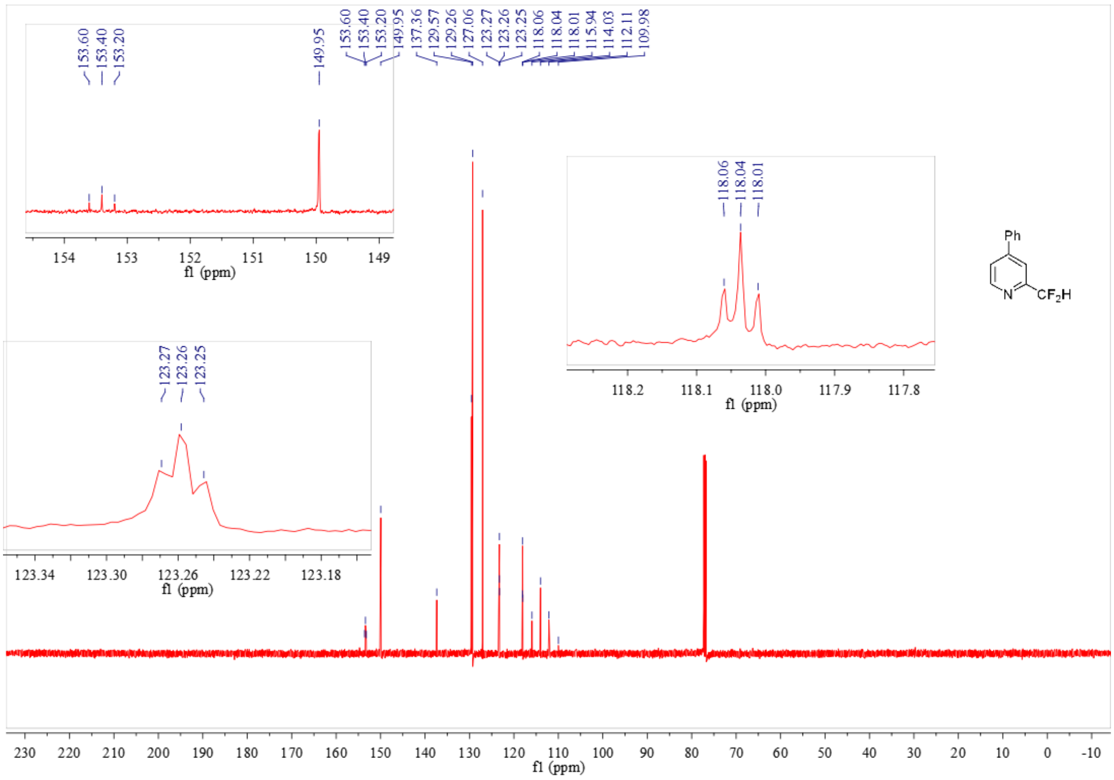

^{1}H NMR (400 MHz, $CDCl_3$) methyl 2-(difluoromethyl)pyridine-4-carboxylate 5f

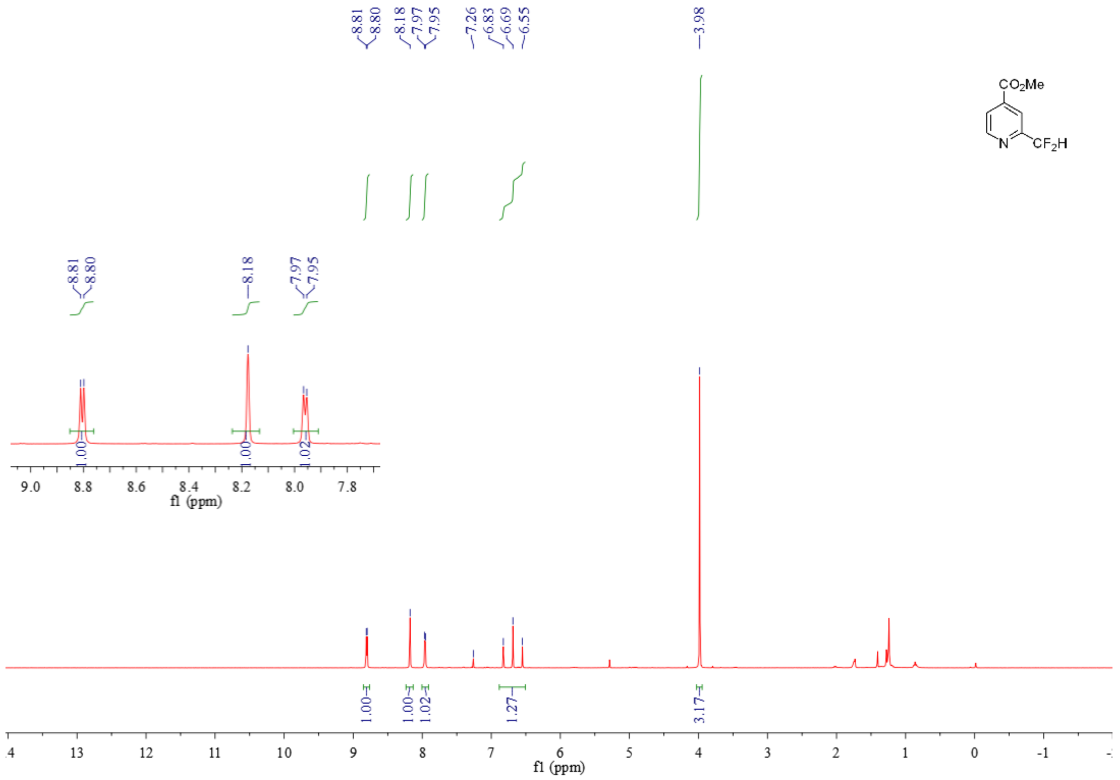

^{19}F NMR (376 MHz, $CDCl_3$) methyl 2-(difluoromethyl)pyridine-4-carboxylate 5f

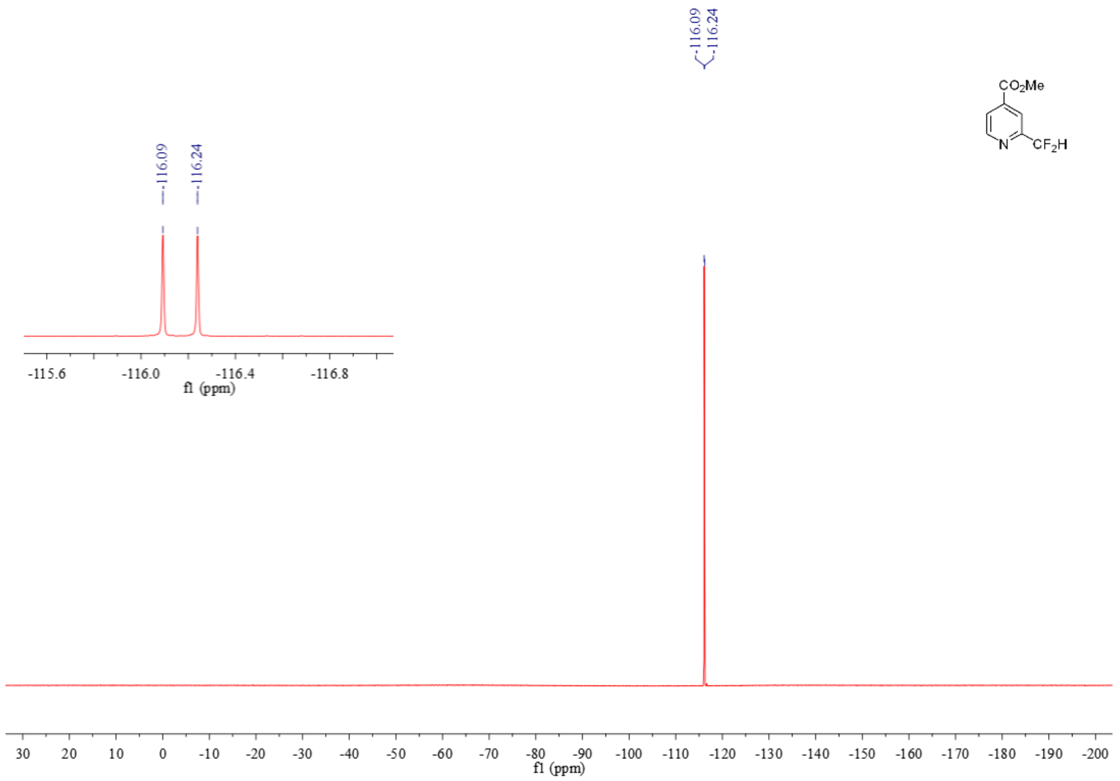

^{13}C NMR (101 MHz, $CDCl_3$) methyl 2-(difluoromethyl)pyridine-4-carboxylate 5f

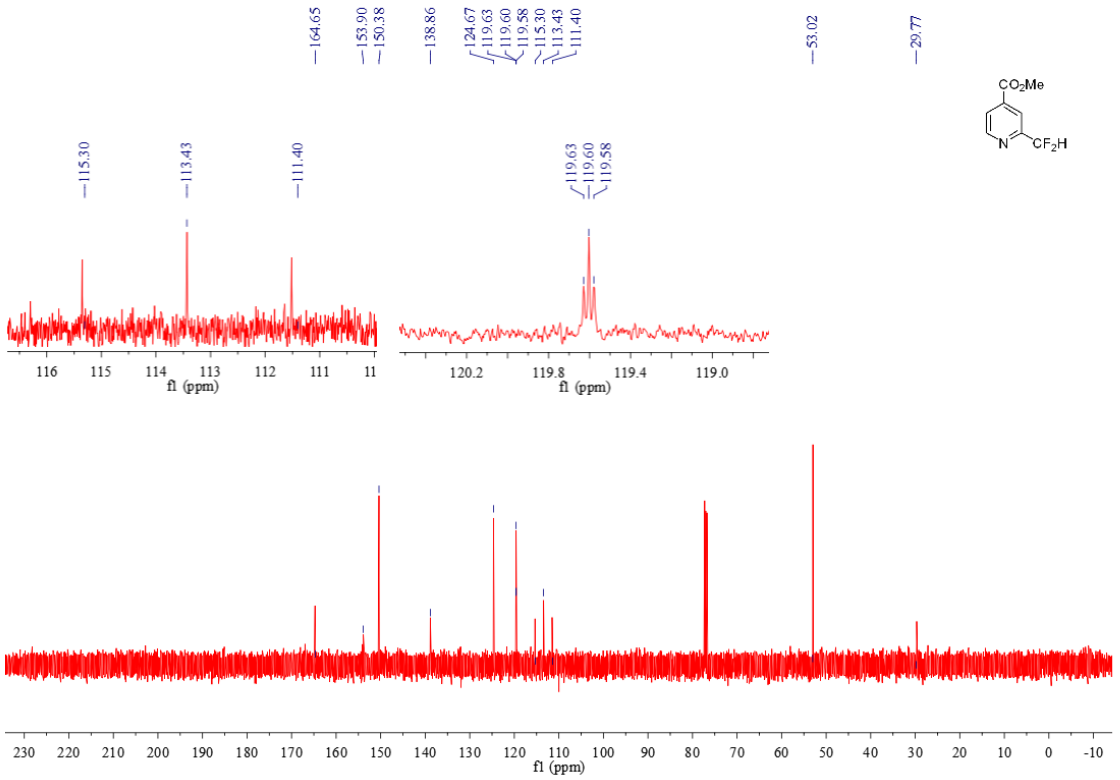

^{1}H NMR (400 MHz, $CDCl_3$) *tert*-butyl 2-(difluoromethyl)pyridine -4-carboxylate 5g

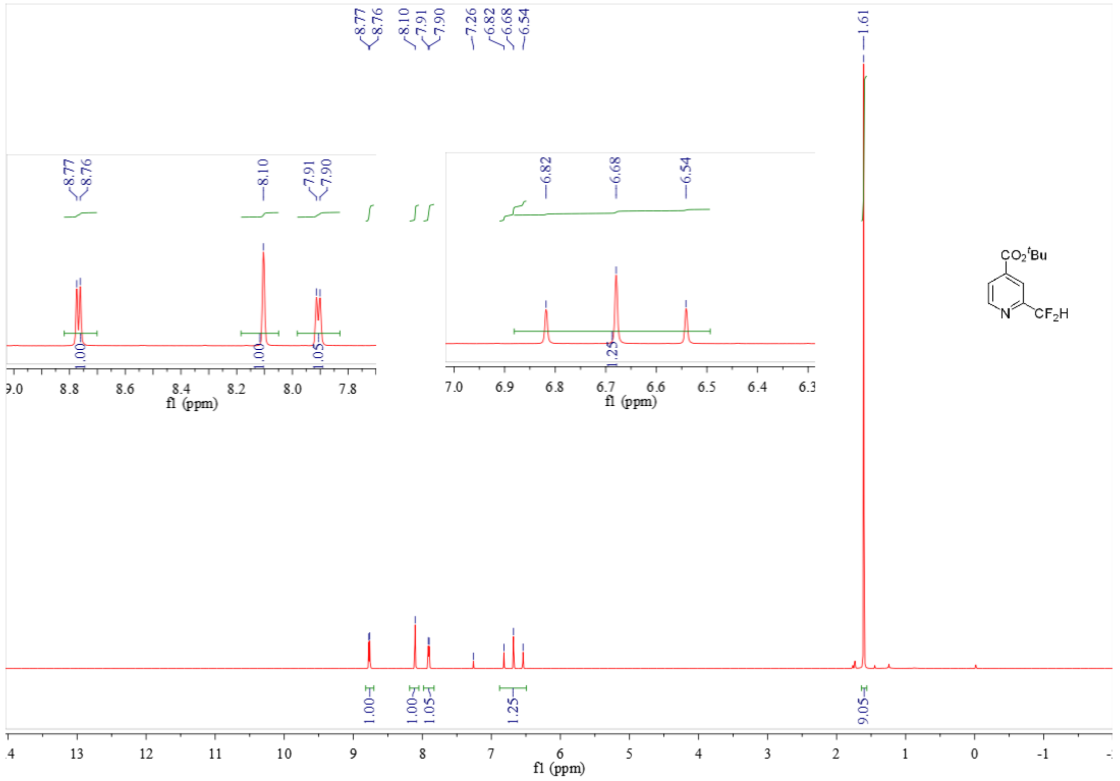

^{19}F NMR (376 MHz, $CDCl_3$) *tert*-butyl 2-(difluoromethyl) pyridine-4-carboxylate 5g

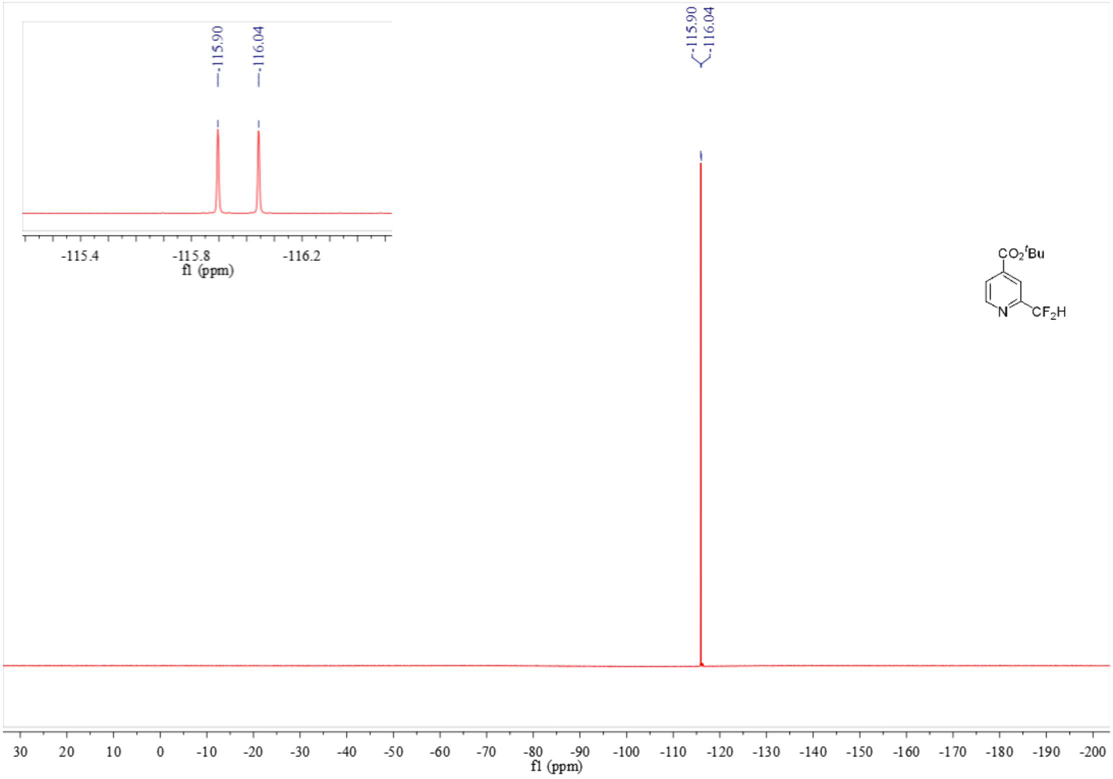

^{13}C NMR (101 MHz, $CDCl_3$) *tert*-butyl 2-(difluoromethyl) pyridine-4-carboxylate 5g

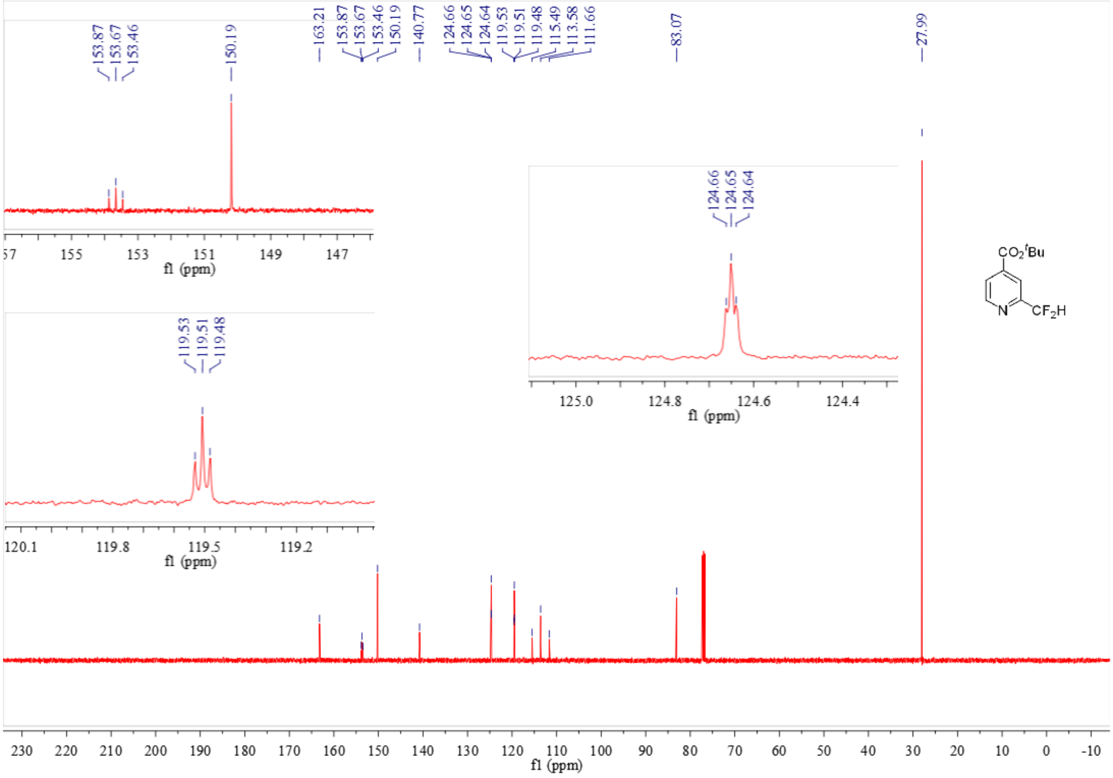

^{1}H NMR (400 MHz, $CDCl_3$) ethyl 4-chloro-6-(difluoromethyl) pyridine-3-carboxylate 5h

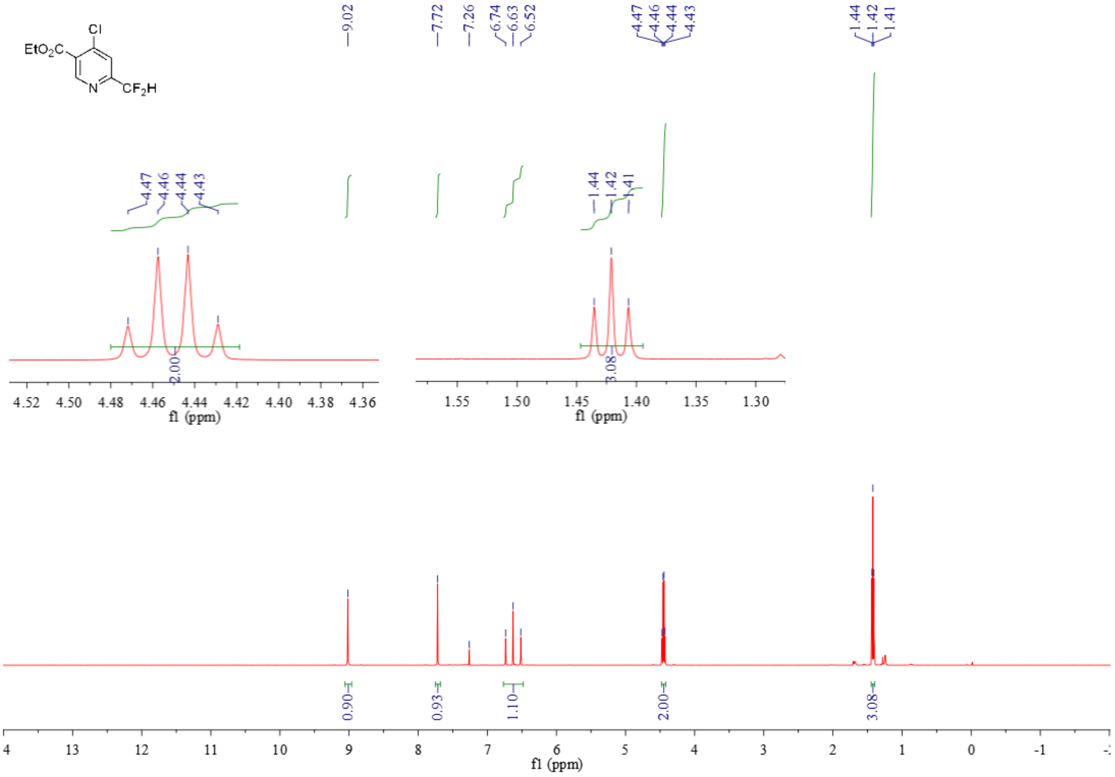

^{19}F NMR (376 MHz, $CDCl_3$) ethyl 4-chloro-6-(difluoromethyl) pyridine-3-carboxylate 5h

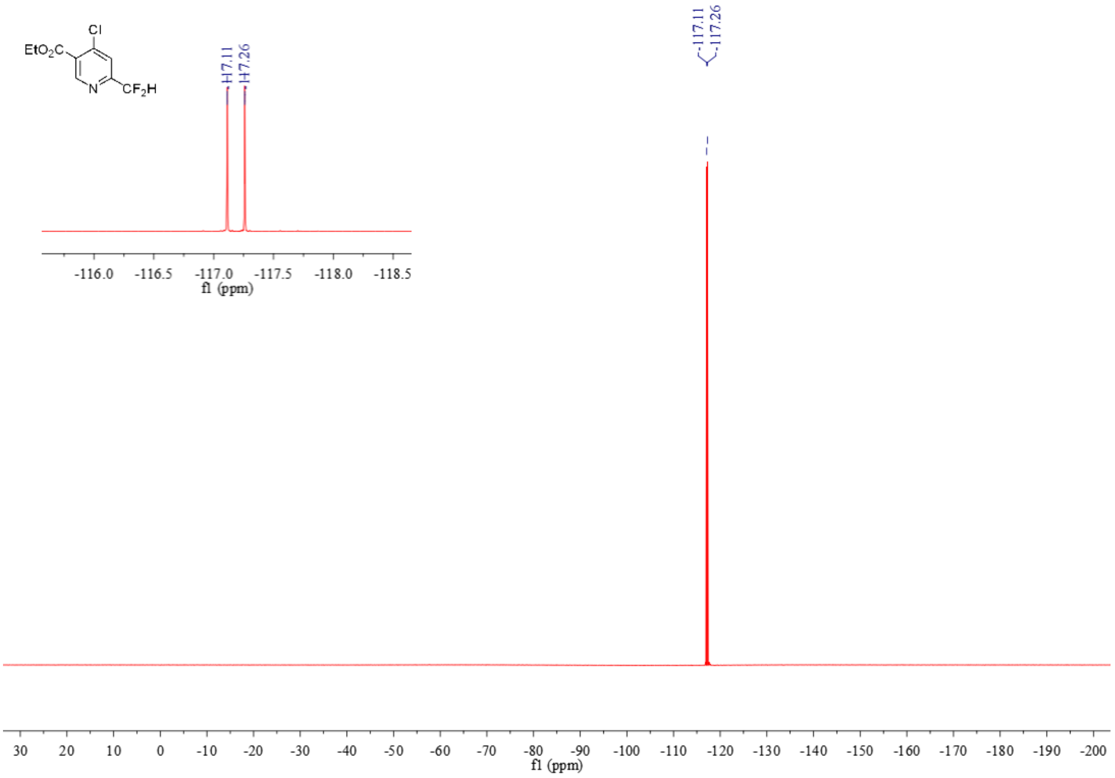

^{13}C NMR (101 MHz, $CDCl_3$) ethyl 4-chloro-6-(difluoromethyl) pyridine-3-carboxylate 5h

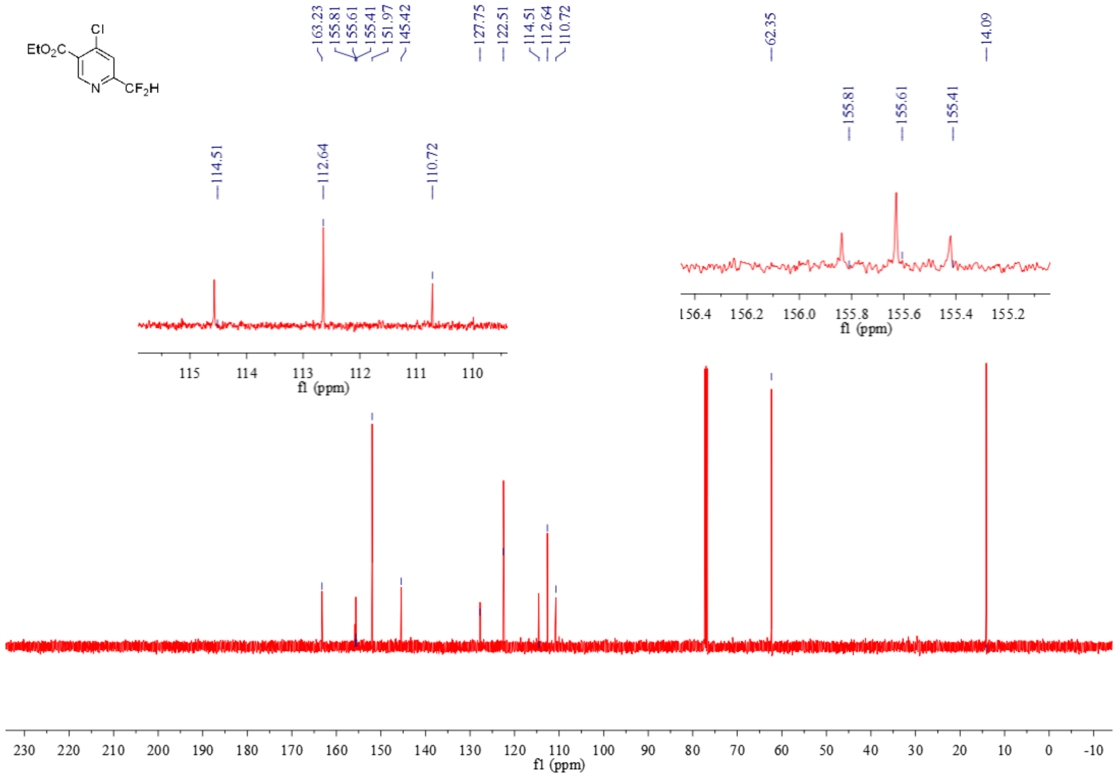

^{1}H NMR (400 MHz, $CDCl_3$) 2-(difluoromethyl)-4,6-dimethyl-pyridine-3-carbonitrile 5i

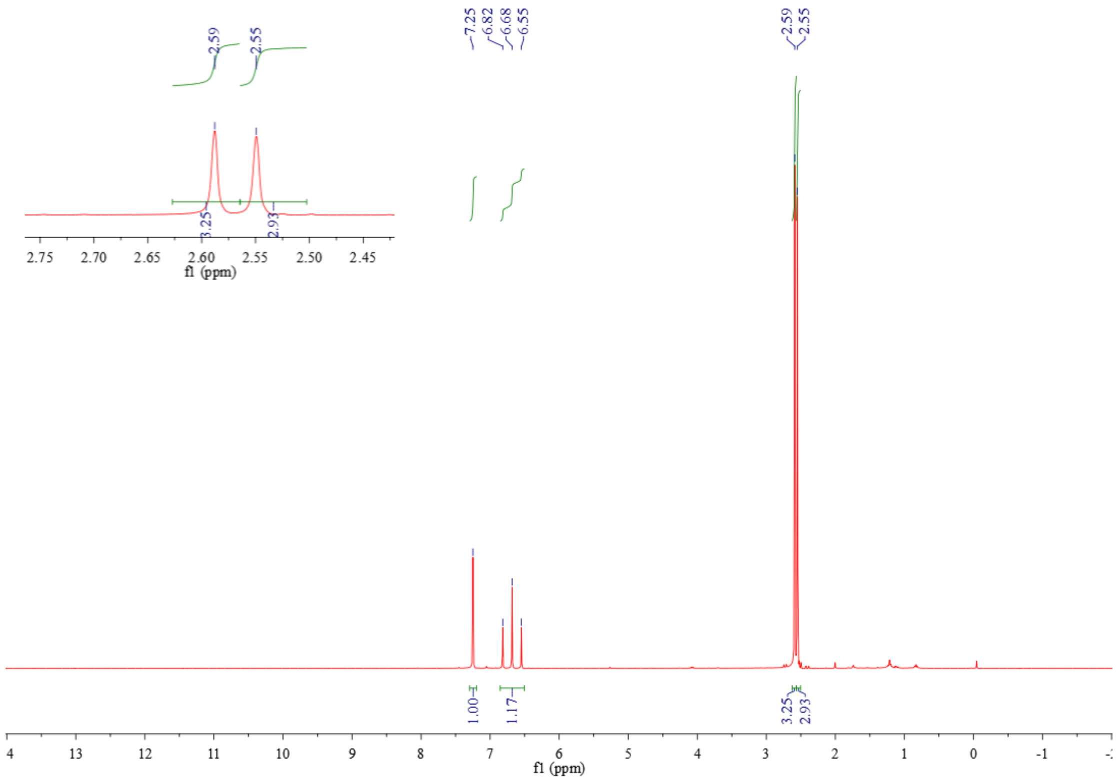

^{19}F NMR (376 MHz, $CDCl_3$) 2-(difluoromethyl)-4,6-dimethyl-pyridine-3-carbonitrile 5i

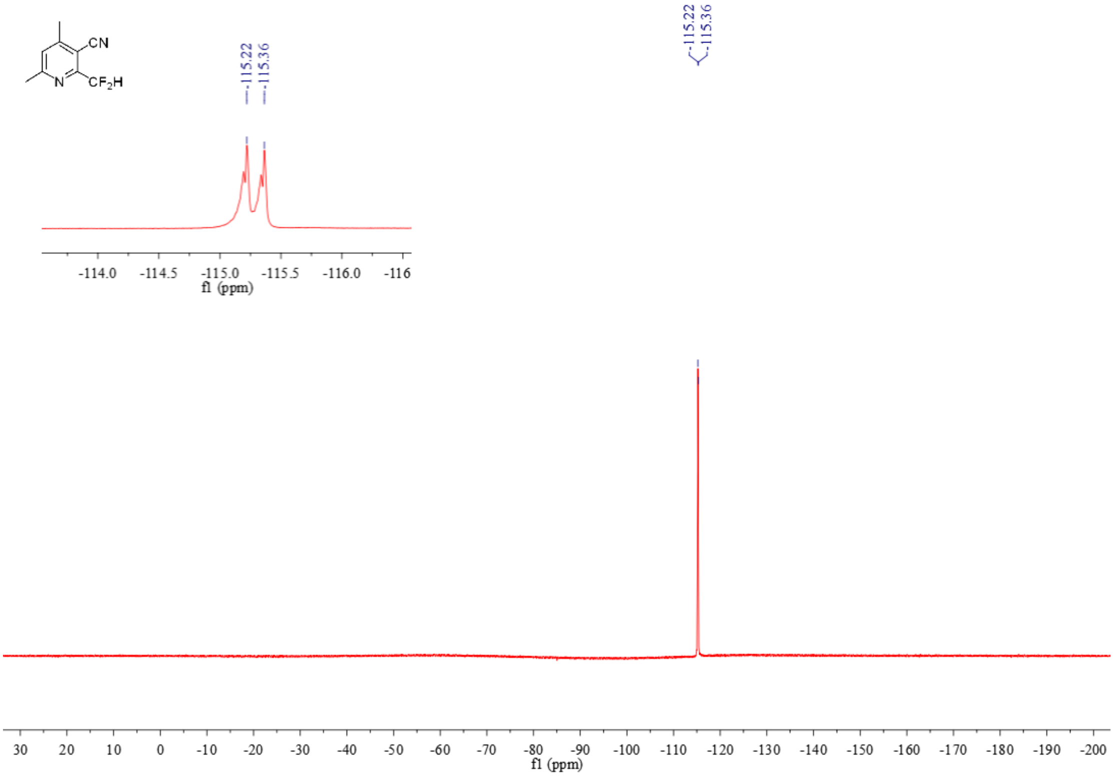

^{13}C NMR (101 MHz, $CDCl_3$) 2-(difluoromethyl)-4,6-dimethyl-pyridine-3-carbonitrile 5i

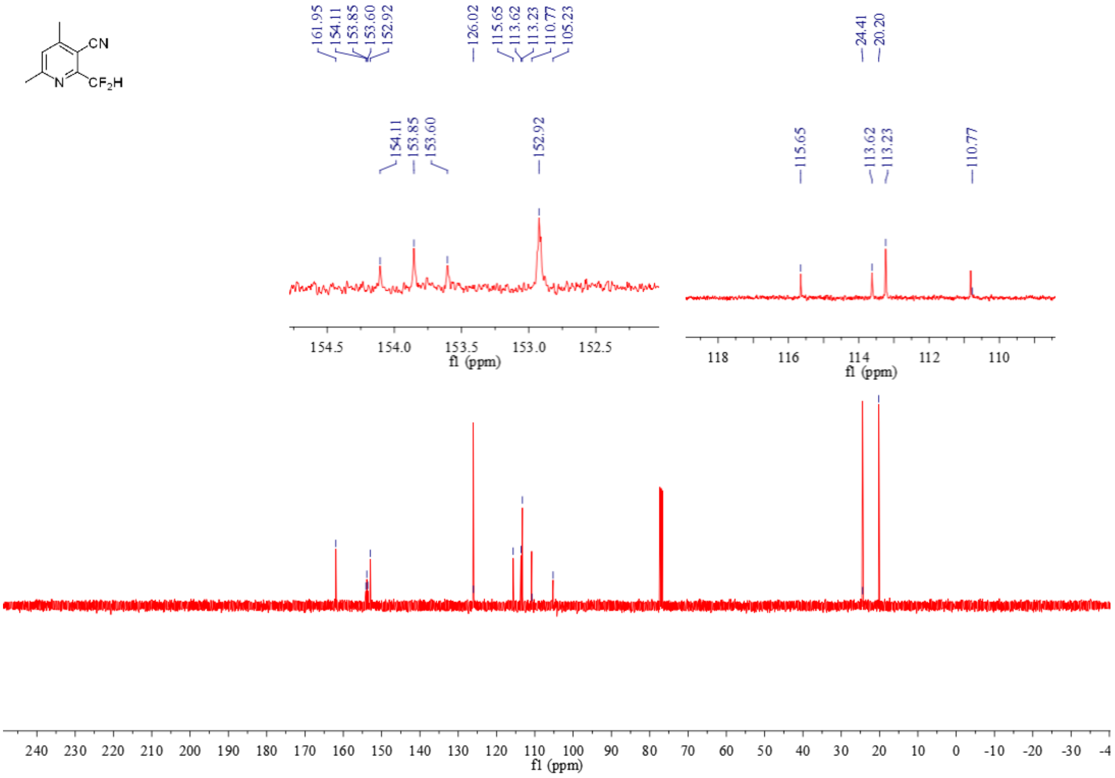

^{1}H NMR (400 MHz, $CDCl_3$) methyl 6-(difluoromethyl)pyrazine -2-carboxylate 5j

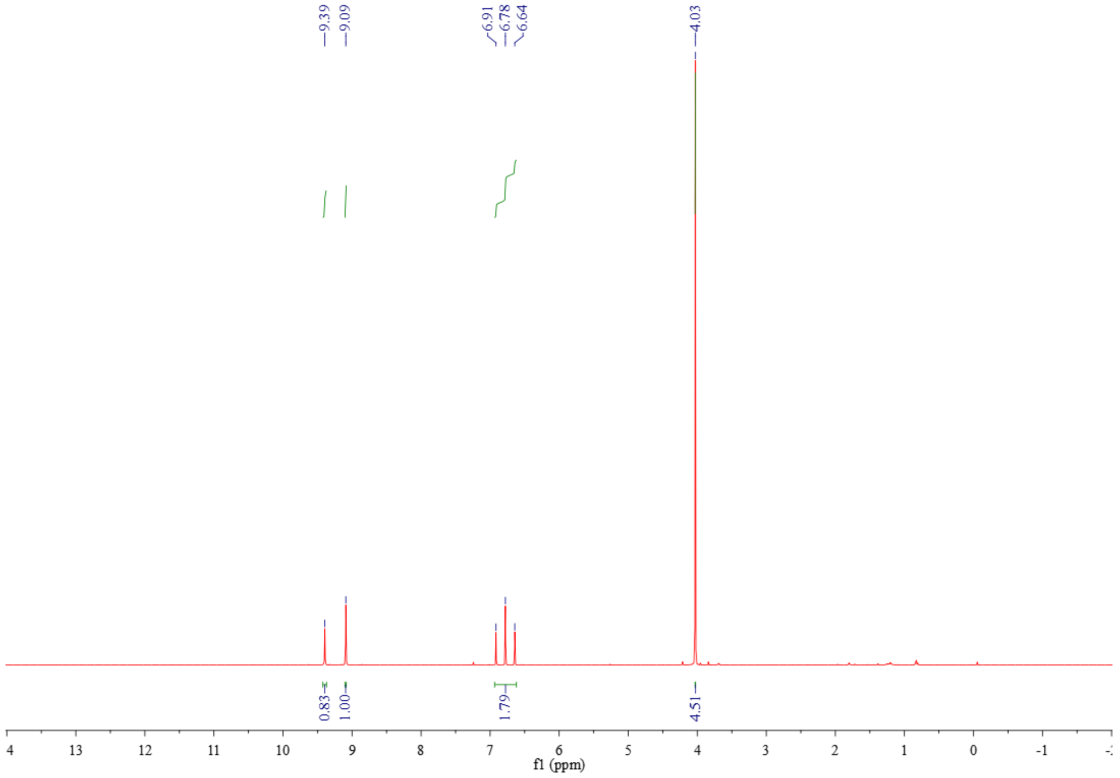

^{19}F NMR (376 MHz, $CDCl_3$) methyl 6-(difluoromethyl)pyrazine-2-carboxylate 5j

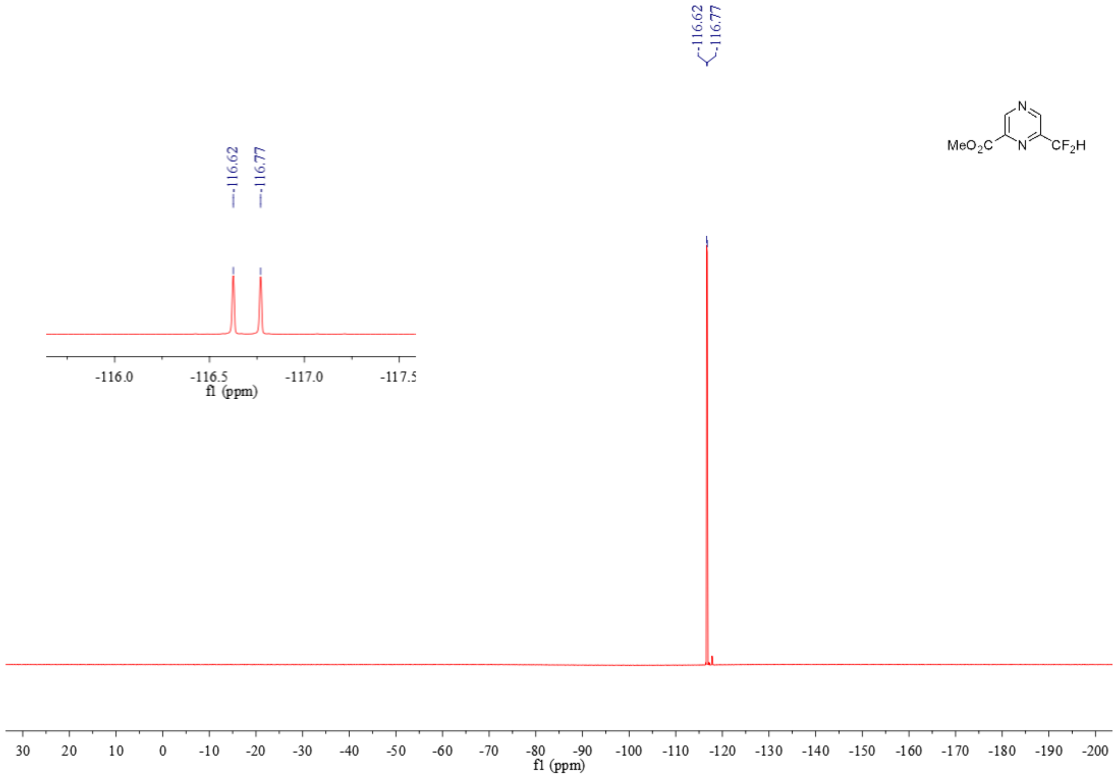

^{13}C NMR (101 MHz, $CDCl_3$) methyl 6-(difluoromethyl)pyrazine-2-carboxylate 5j

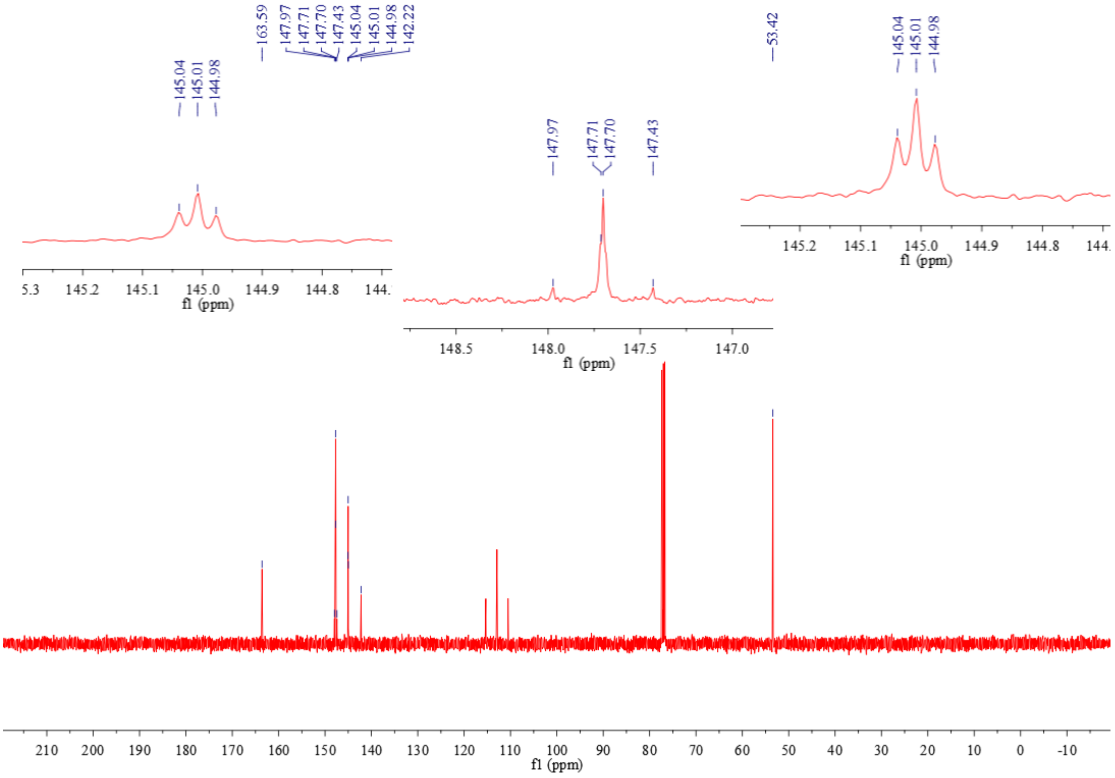

^{1}H NMR (400 MHz, $CDCl_3$) methyl 3-(difluoromethyl)pyrazine-2-carboxylate 5k

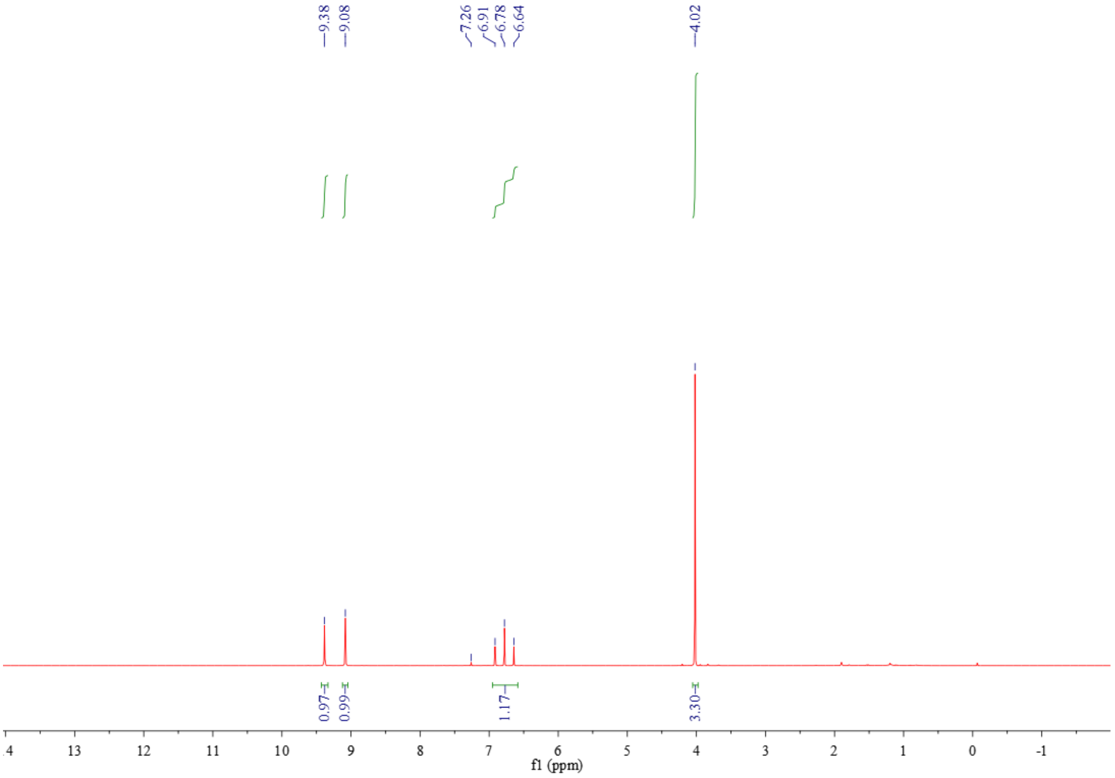

^{19}F NMR (376 MHz, $CDCl_3$) methyl 3-(difluoromethyl)pyrazine-2-carboxylate 5k

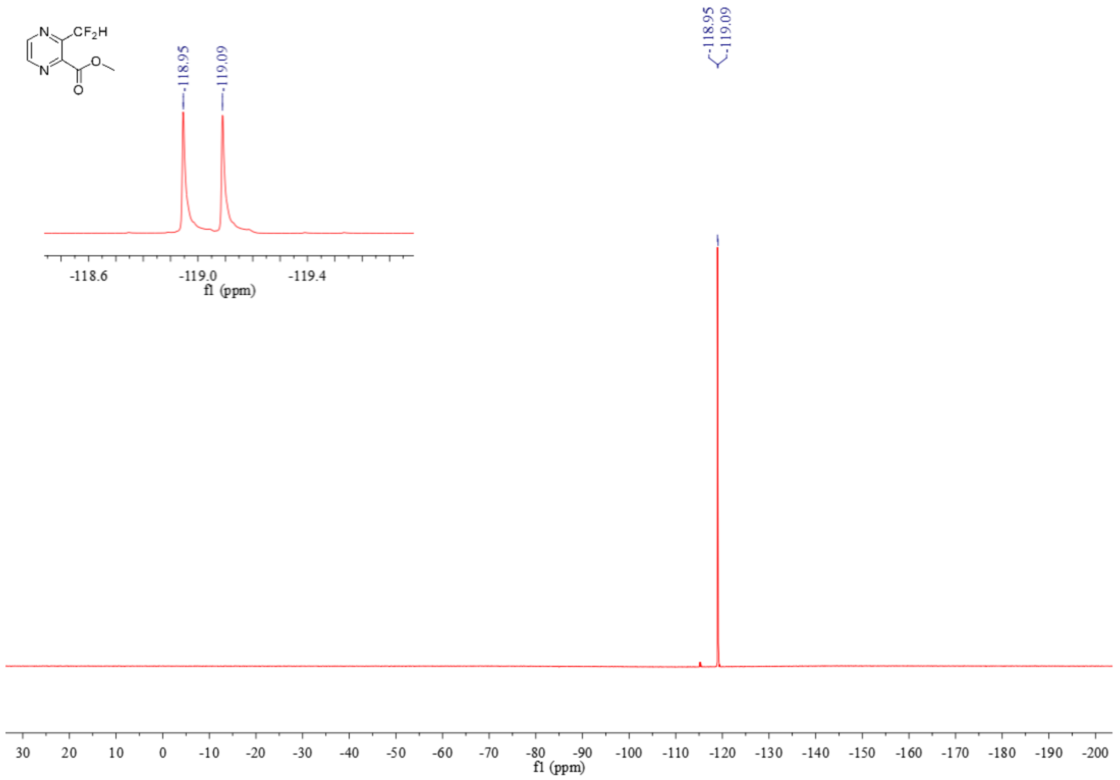

^{13}C NMR (101 MHz, $CDCl_3$) methyl 3-(difluoromethyl)pyrazine -2-carboxylate 5k

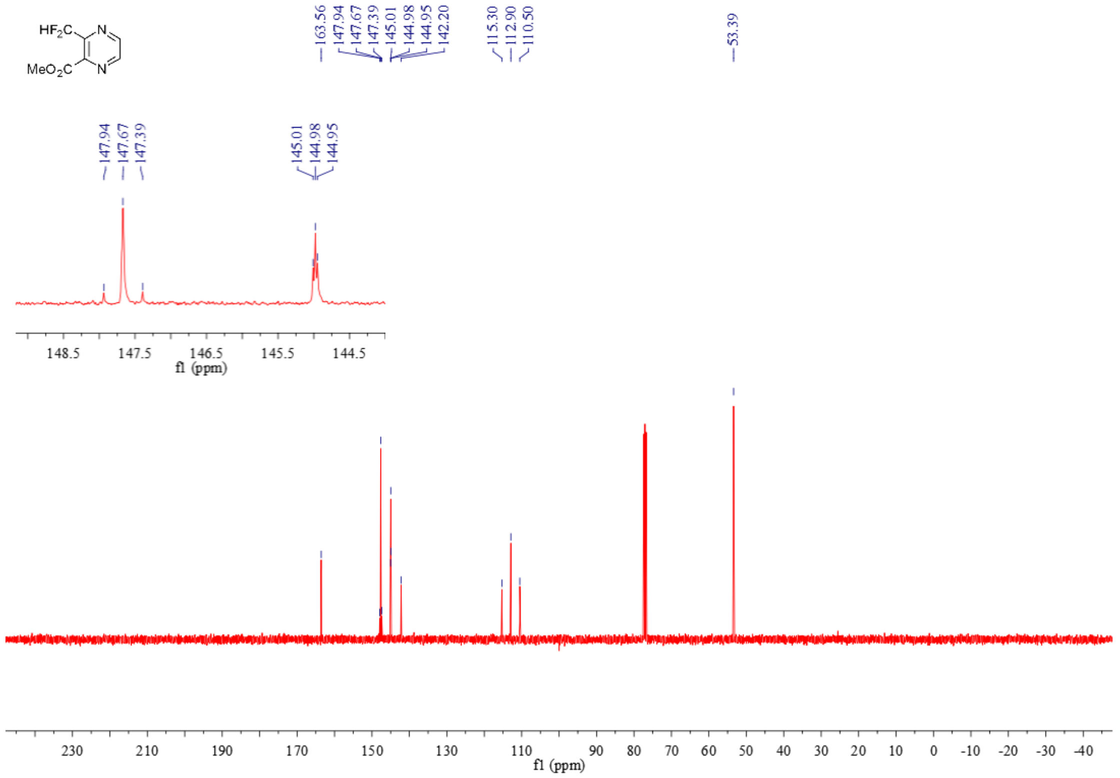

^{1}H NMR (400 MHz, $CDCl_3$) 2-(difluoromethyl)-6-pyrazol-1-yl-pyrazine 5l

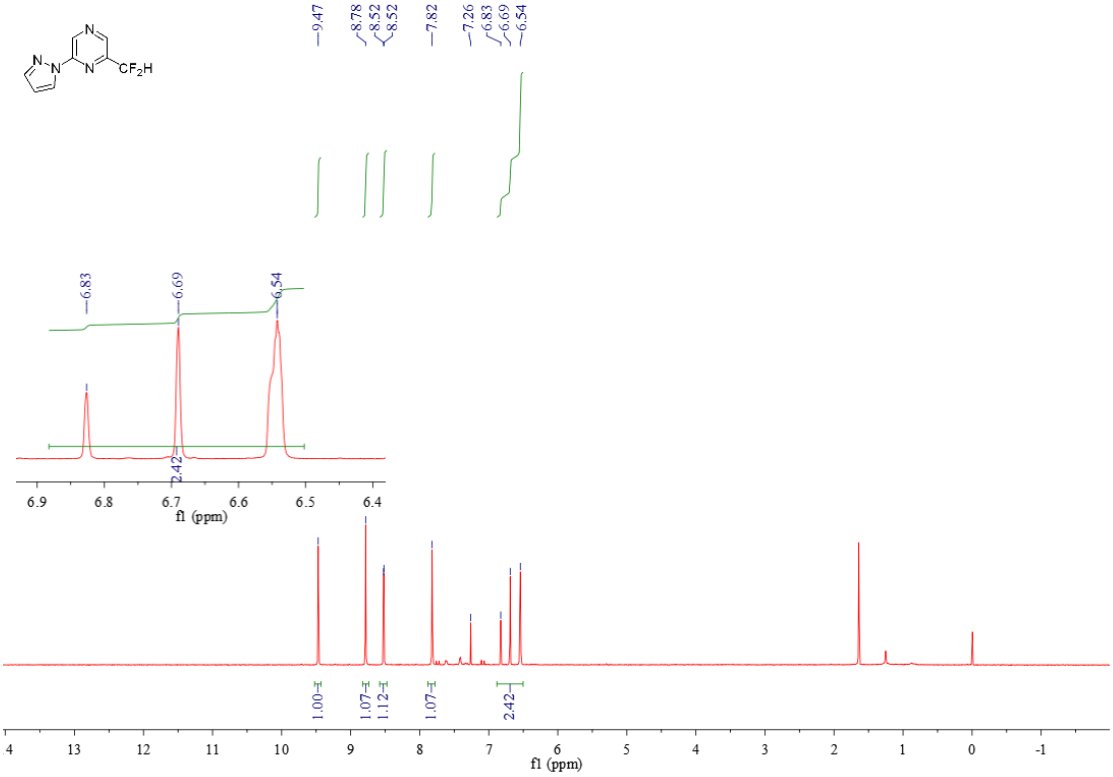

^{19}F NMR (376 MHz, $CDCl_3$) 2-(difluoromethyl)-6-pyrazol-1-yl-pyrazine 5l

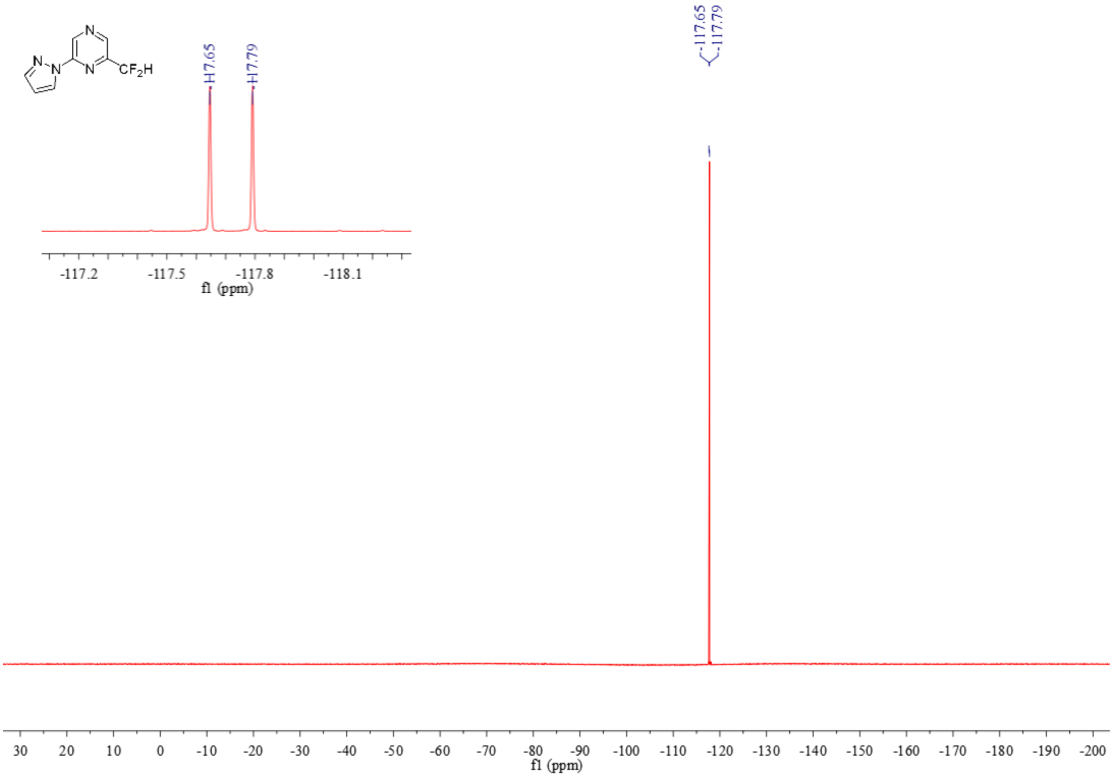

^{13}C NMR (101 MHz, $CDCl_3$) 2-(difluoromethyl)-6-pyrazol-1-yl-pyrazine 5l

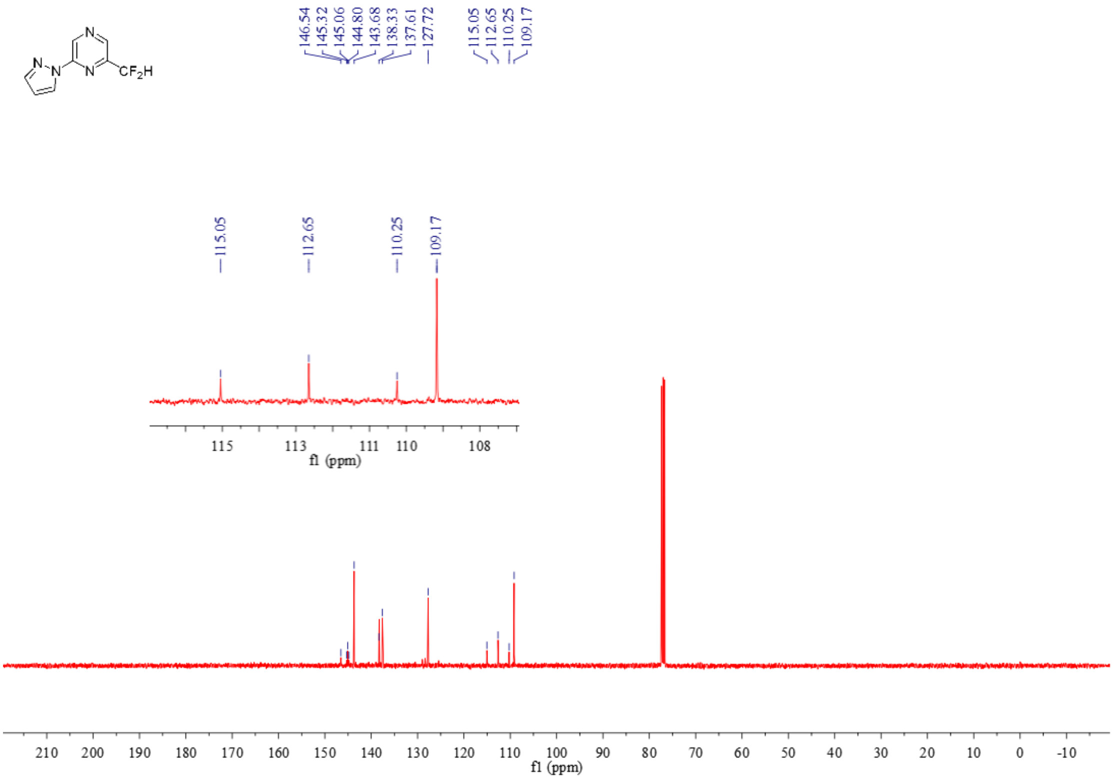

^{1}H NMR (400 MHz, $CDCl_3$) 2-(difluoromethyl)-3-methyl-quinoxaline 5m

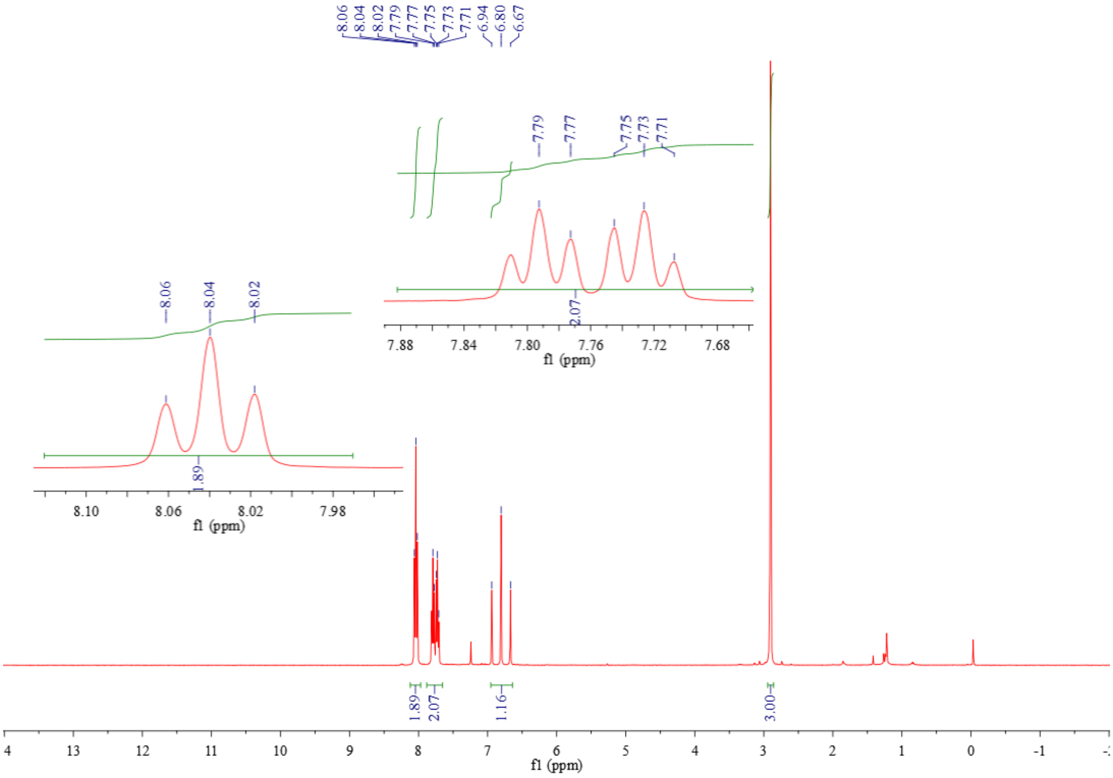

^{19}F NMR (376 MHz, $CDCl_3$) 2-(difluoromethyl)-3-methyl-quinoxaline 5m

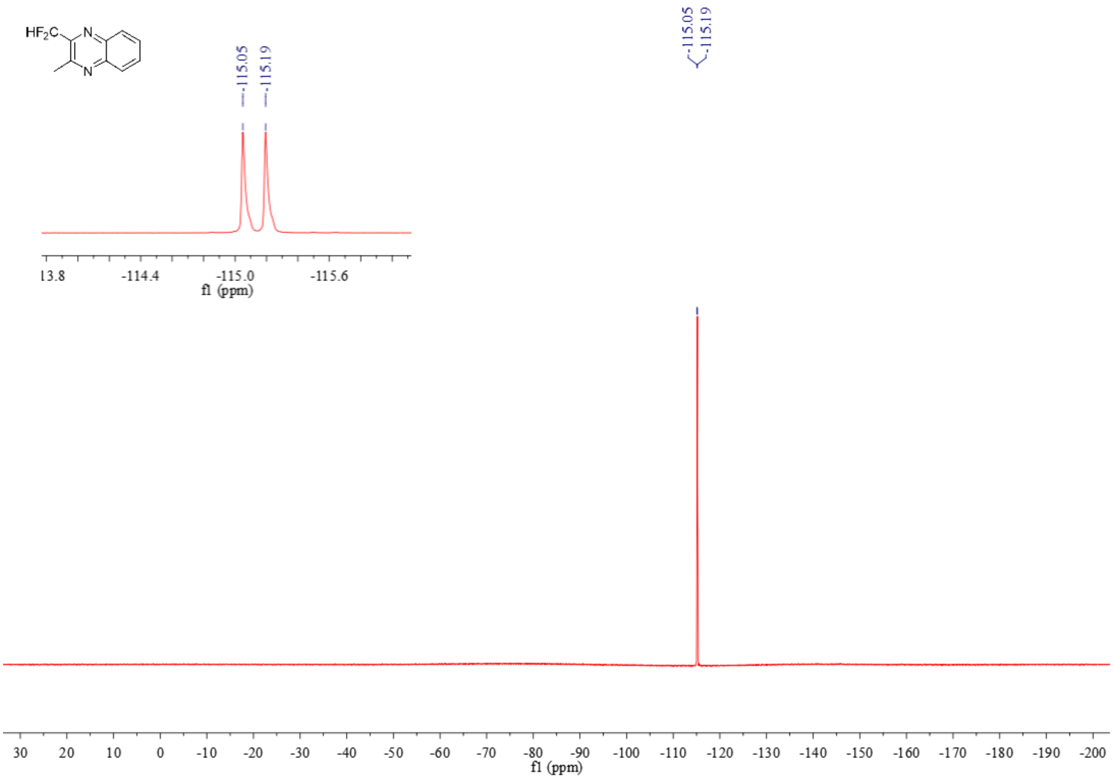

^{13}C NMR (101 MHz, $CDCl_3$) 2-(difluoromethyl)-3-methyl-quinoxaline 5m

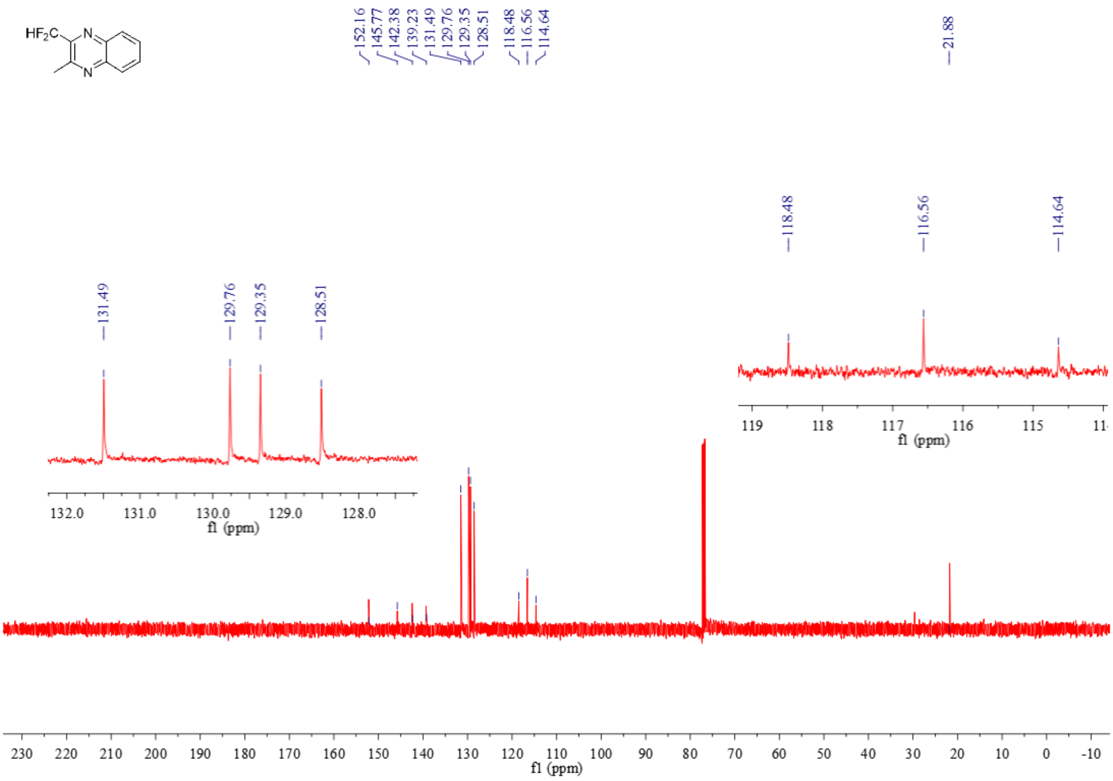

^{1}H NMR (400 MHz, $CDCl_3$) 4-(difluoromethyl)-6-methyl-2 -methylsulfanyl-pyrimidine 5n

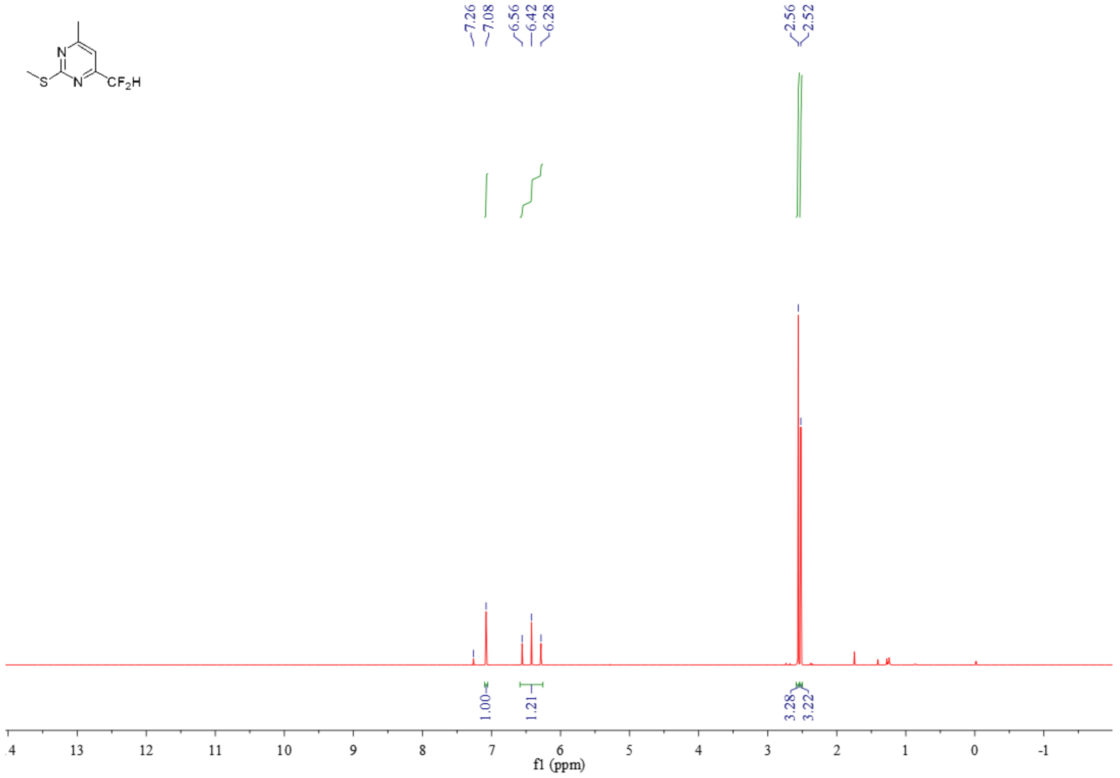

^{19}F NMR (376 MHz, $CDCl_3$) 4-(difluoromethyl)-6-methyl -2-methylsulfanyl-pyrimidine 5n

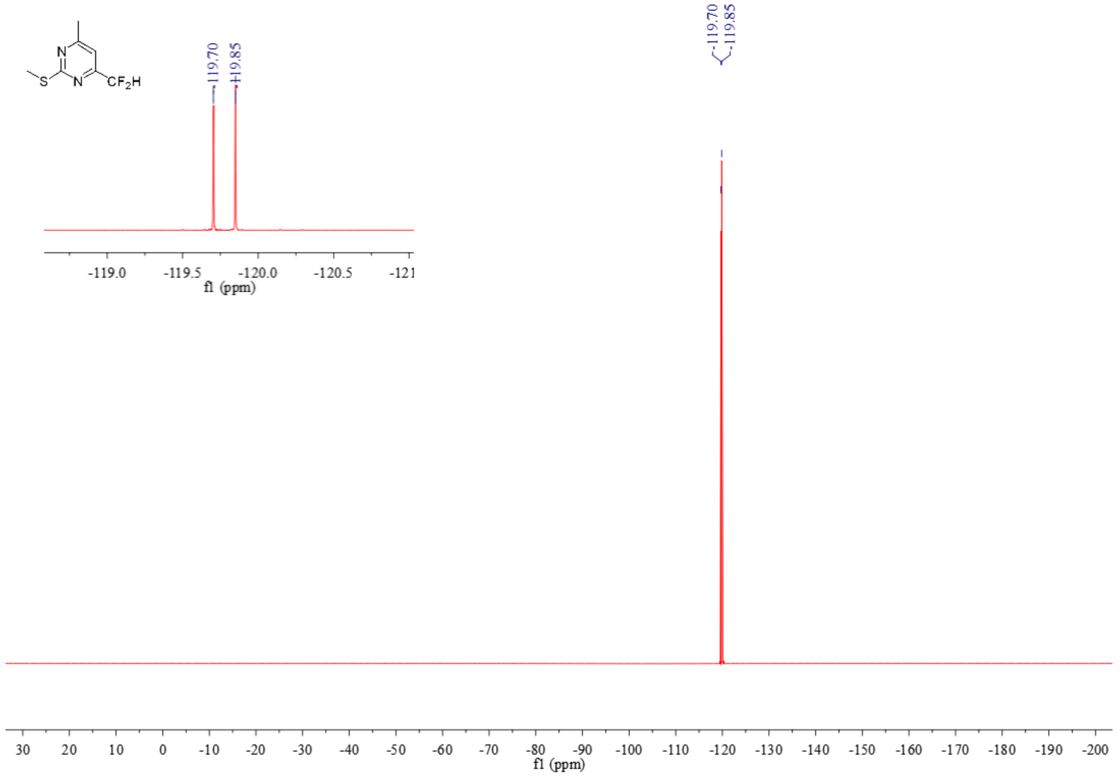

^{13}C NMR (101 MHz, $CDCl_3$) 4-(difluoromethyl)-6-methyl-2 -methylsulfanyl-pyrimidine 5n

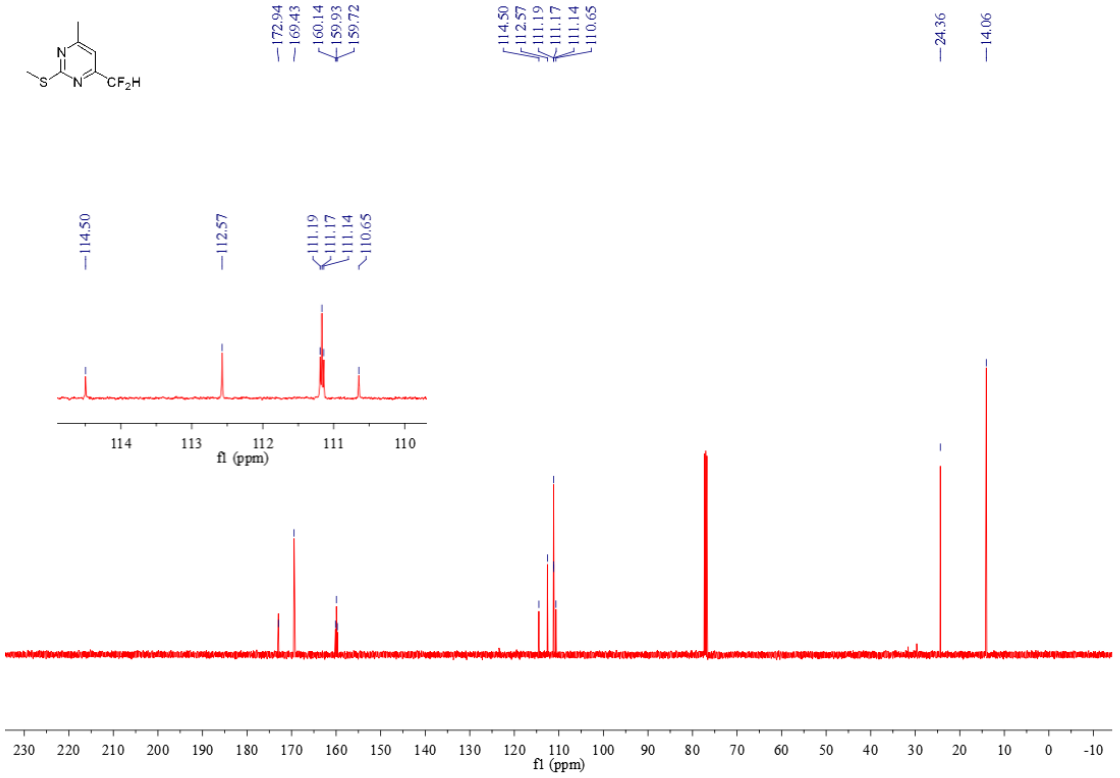

^{1}H NMR (400 MHz, $CDCl_3$) 4-(difluoromethyl)-6,7-dimethoxy-quinazoline 5o

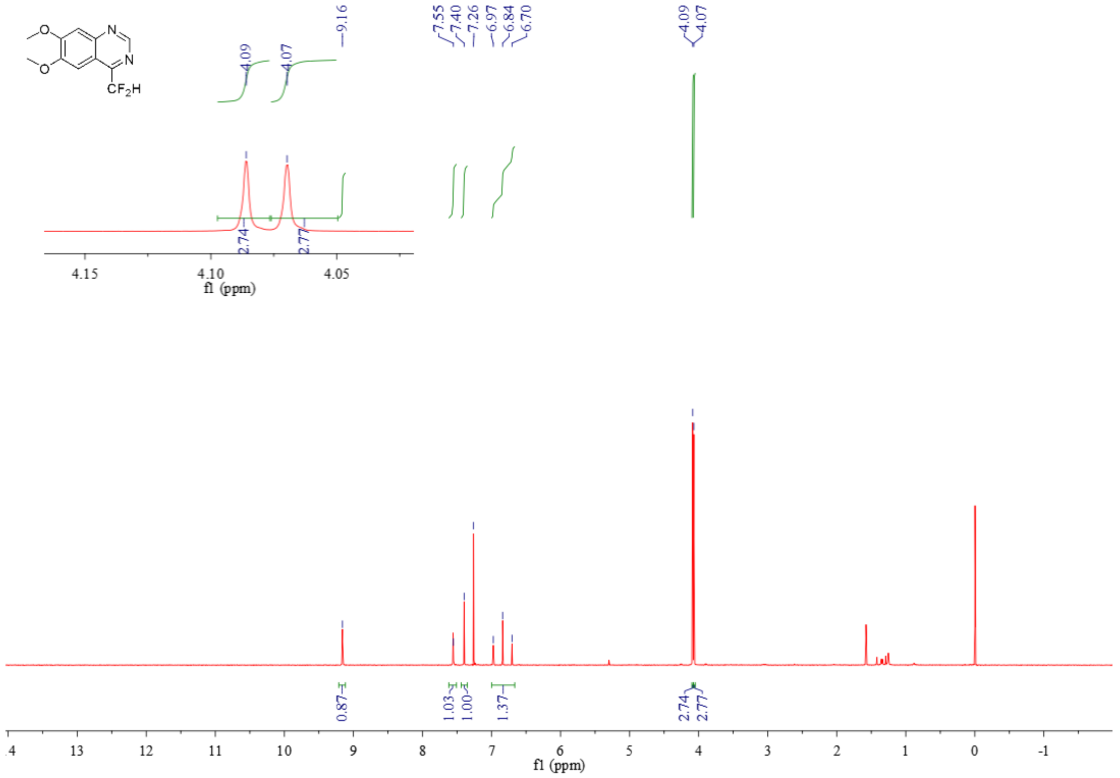

^{19}F NMR (376 MHz, $CDCl_3$) 4-(difluoromethyl)-6,7-dimethoxy-quinazoline 5o

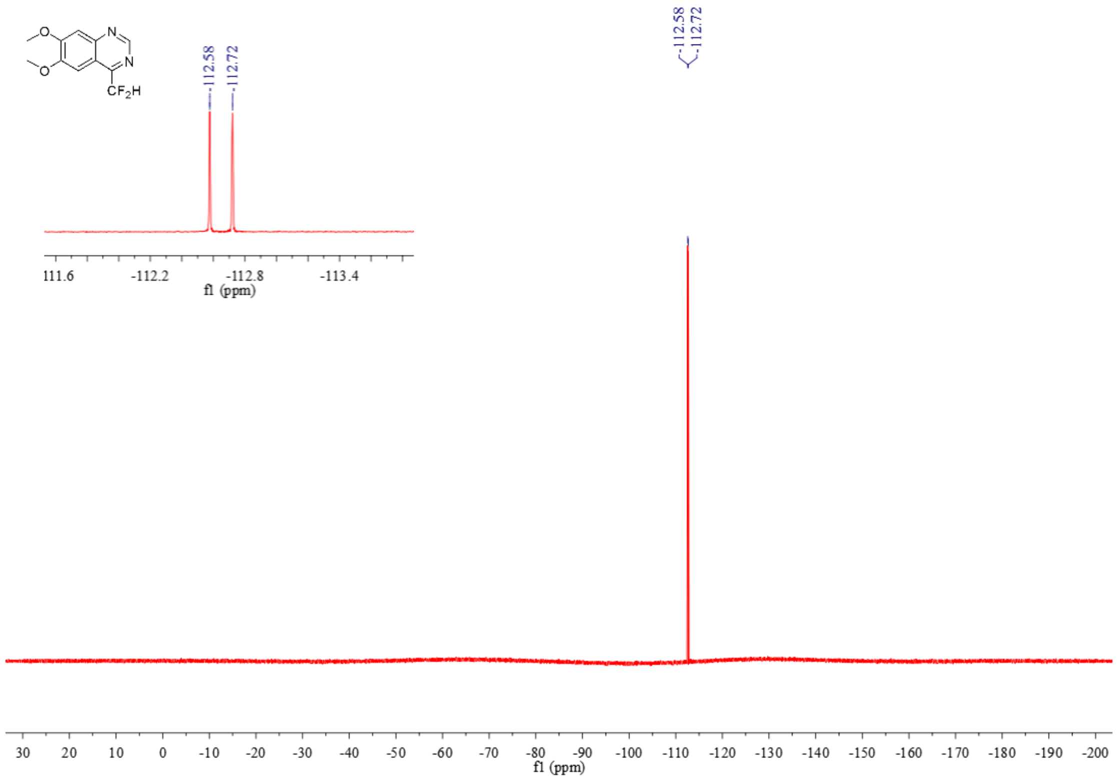

^{13}C NMR (101 MHz, $CDCl_3$) 4-(difluoromethyl)-6,7-dimethoxy-quinazoline 5o

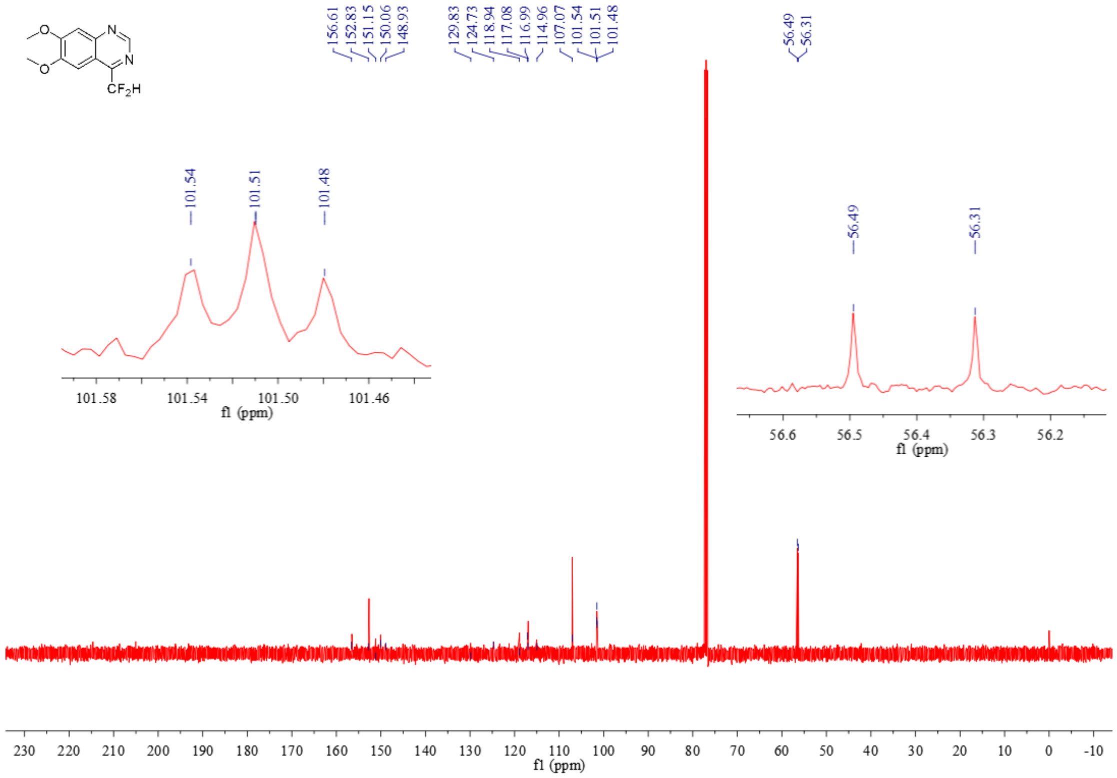

^{1}H NMR (400 MHz, $CDCl_3$) 6-chloro-2-(difluoromethyl)quinolone 5p

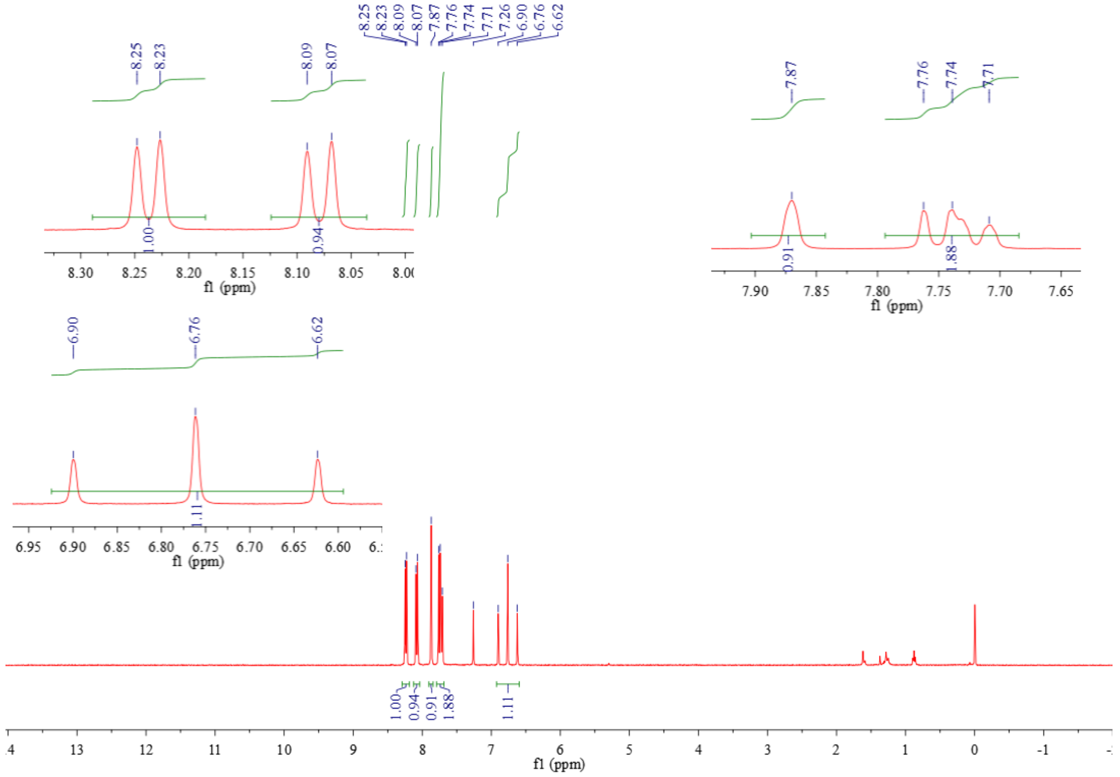

^{19}F NMR (376 MHz, $CDCl_3$) 6-chloro-2-(difluoromethyl)quinolone 5p

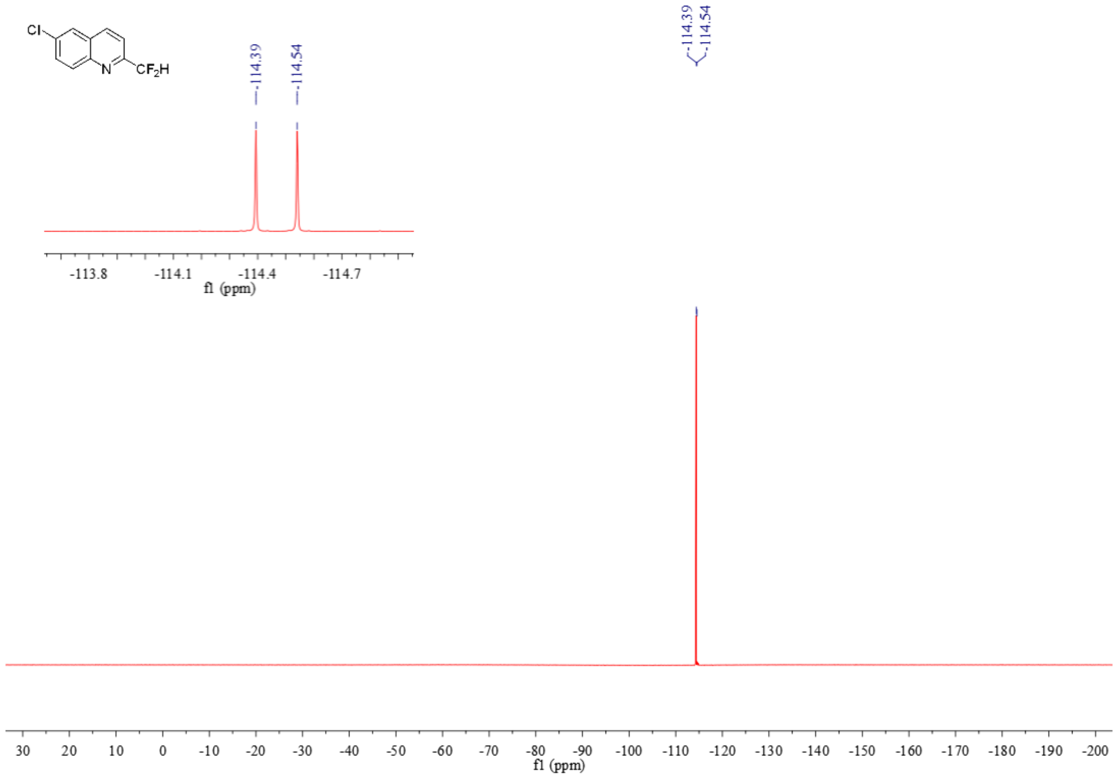

^{13}C NMR (101 MHz, $CDCl_3$) 6-chloro-2-(difluoromethyl)quinolone 5p

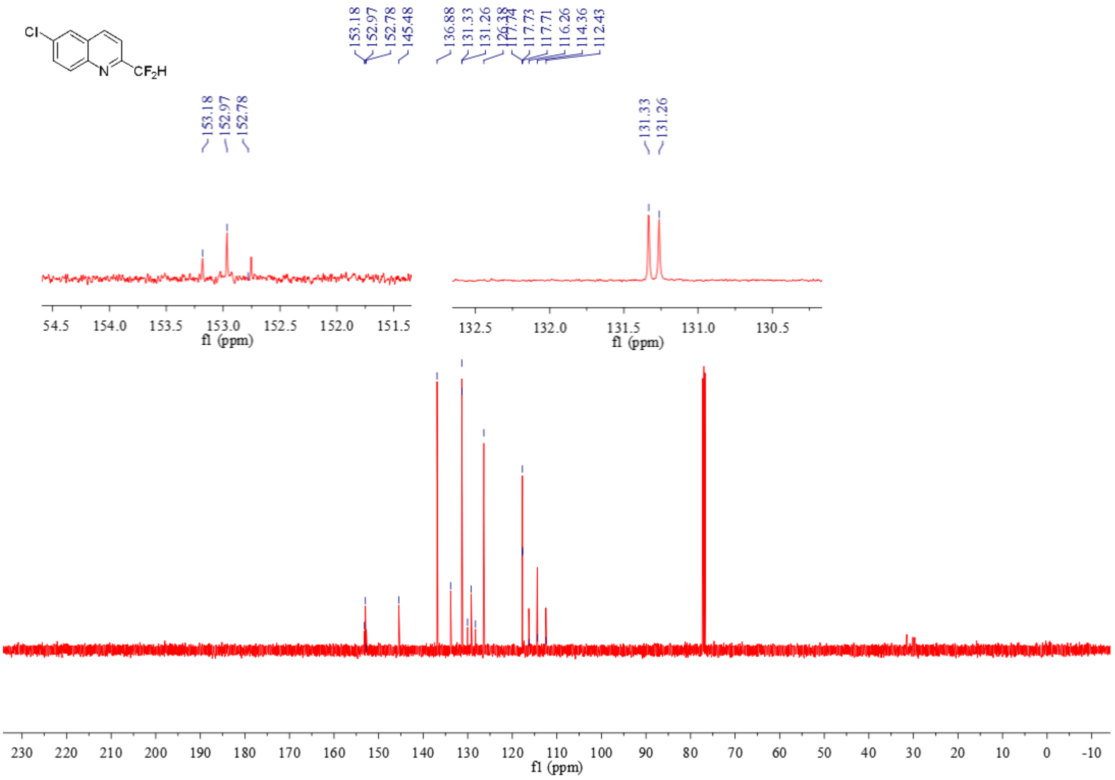

^{1}H NMR (400 MHz, $CDCl_3$) 4-(difluoromethyl)-1-isopentyl-1*H*-imidazo [4,5-*c*]quinolone 6a

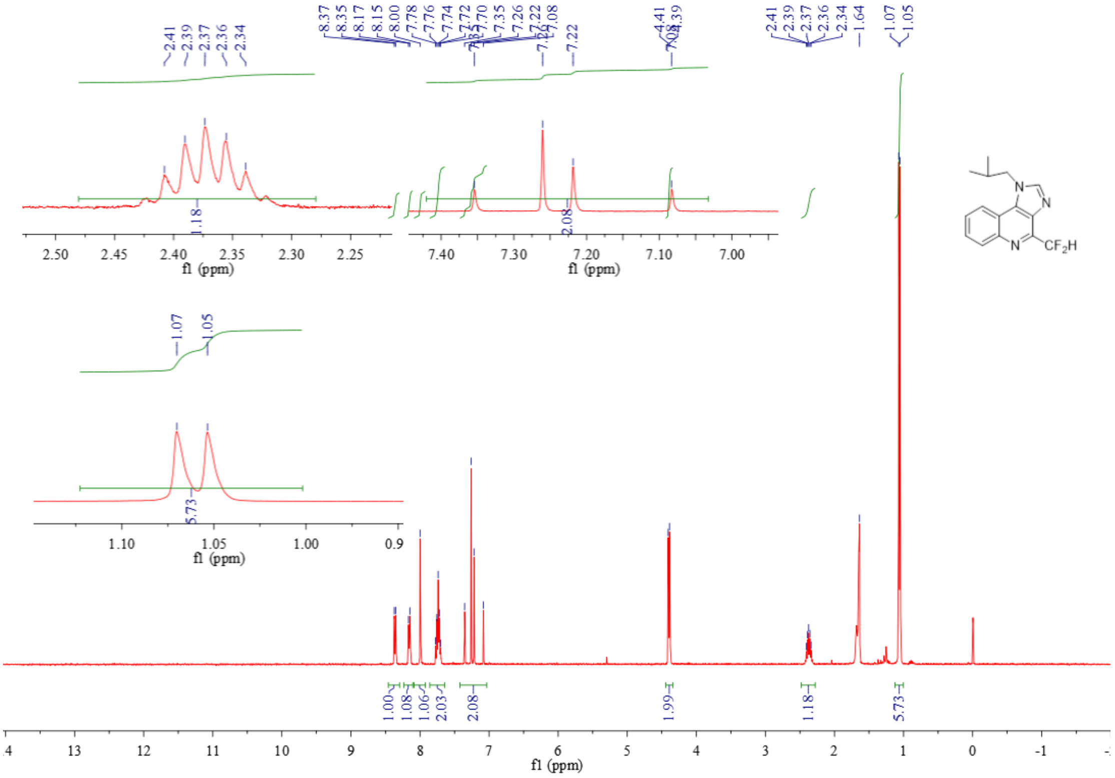

^{19}F NMR (376 MHz, $CDCl_3$) 4-(difluoromethyl)-1-isopentyl-1*H*-imidazo [4,5-*c*]quinolone 6a

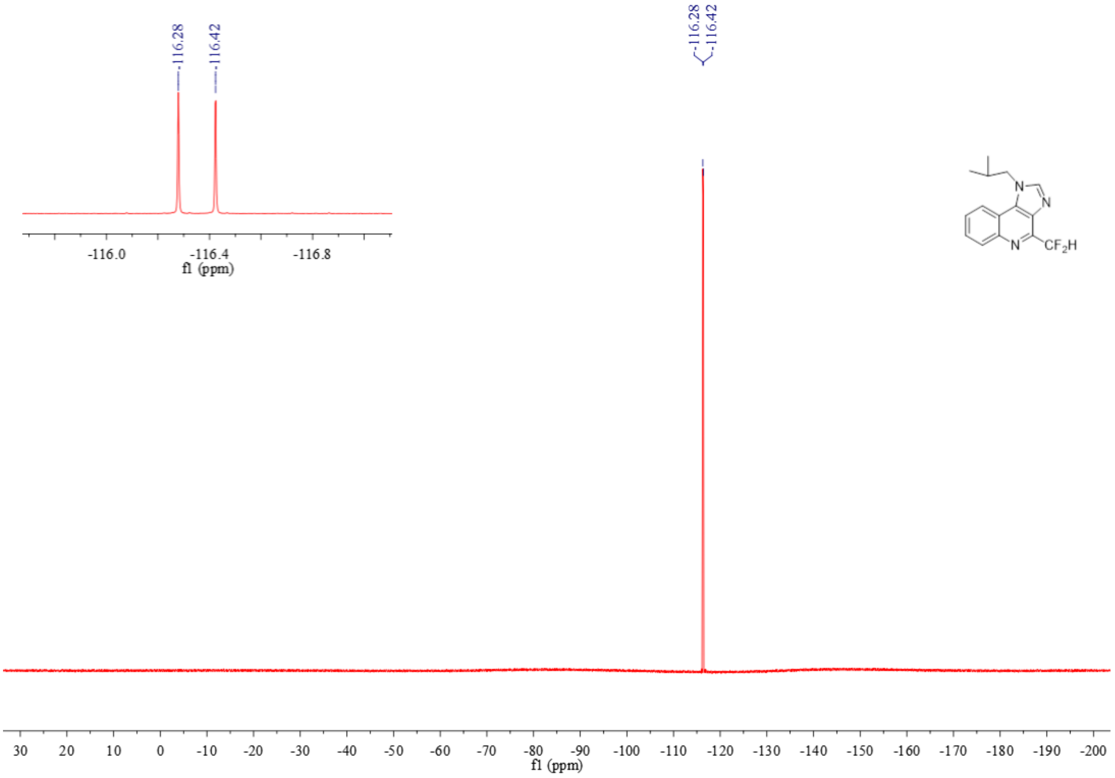

^{13}C NMR (101 MHz, $CDCl_3$) 4-(difluoromethyl)-1-isopentyl-1*H*-imidazo [4,5-*c*]quinolone 6a

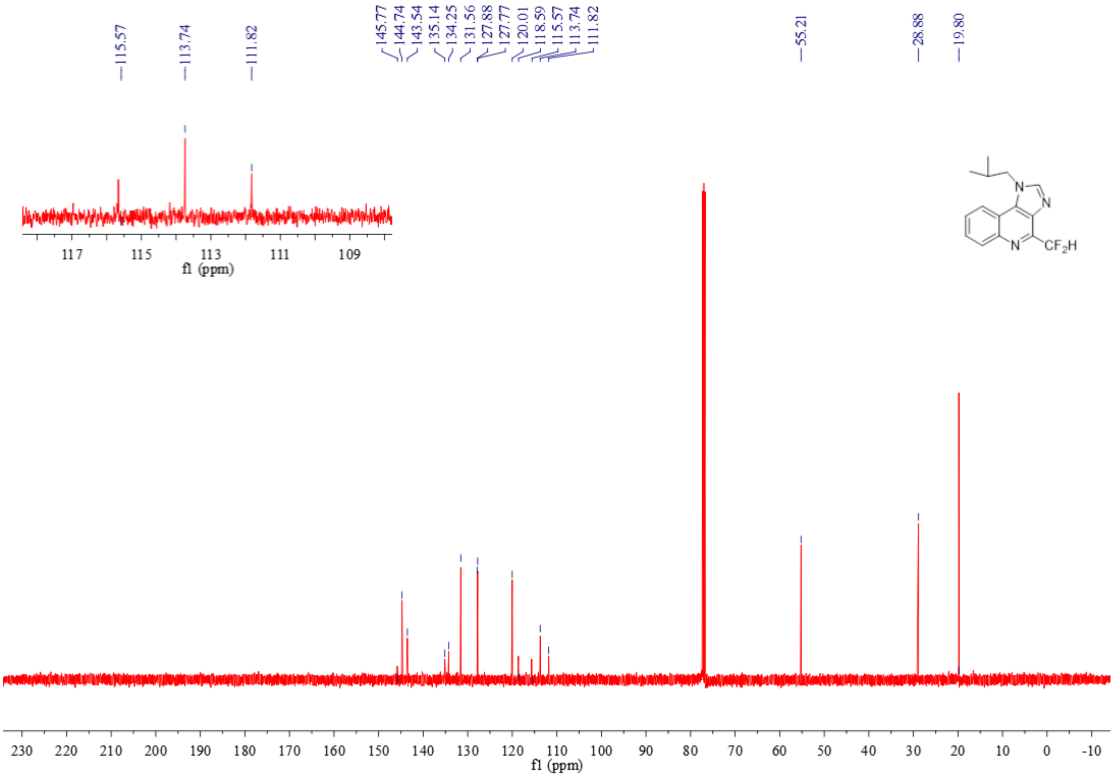

^{1}H NMR (400 MHz, CDCl3) 2-((5-chloro-3-iodoquinolin-8-yl)oxy)acetate

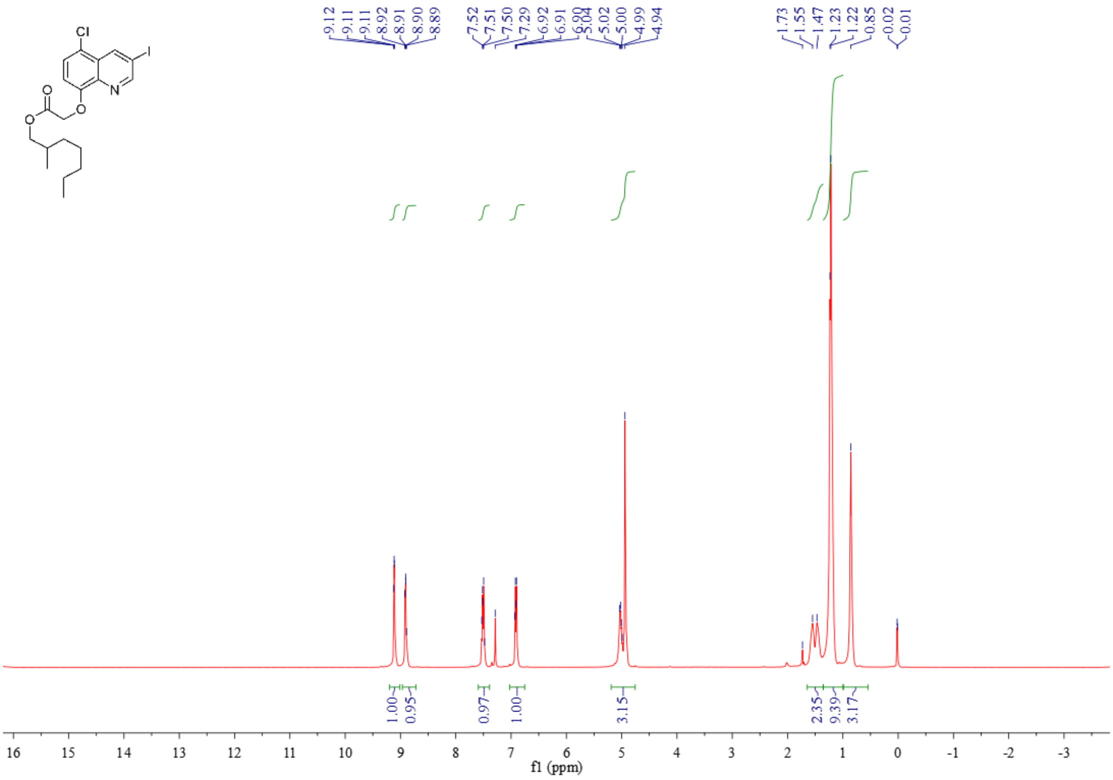

^{13}C NMR (101 MHz, $CDCl_3$) 2-((5-chloro-3-iodoquinolin-8-yl)oxy)acetate

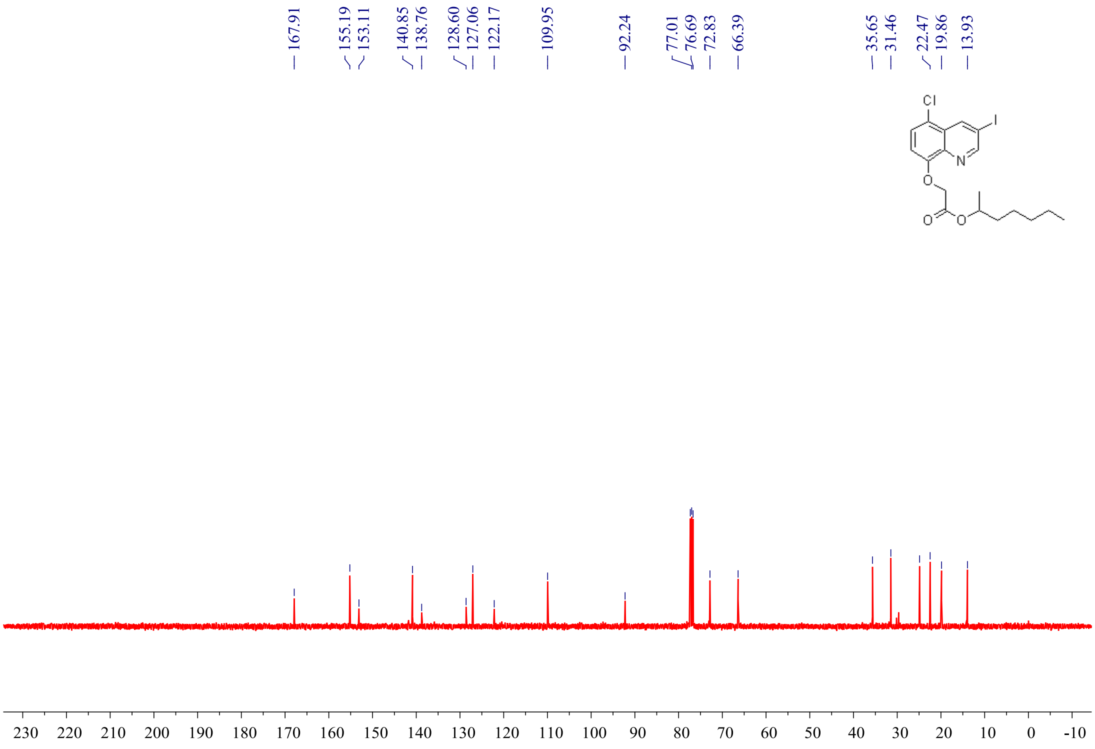

^{1}H NMR (400 MHz, $CDCl_3$) heptan-2-yl 2-((5-chloro-3-(difluoromethyl) quinolin-8-yl)oxy)acetate 6b

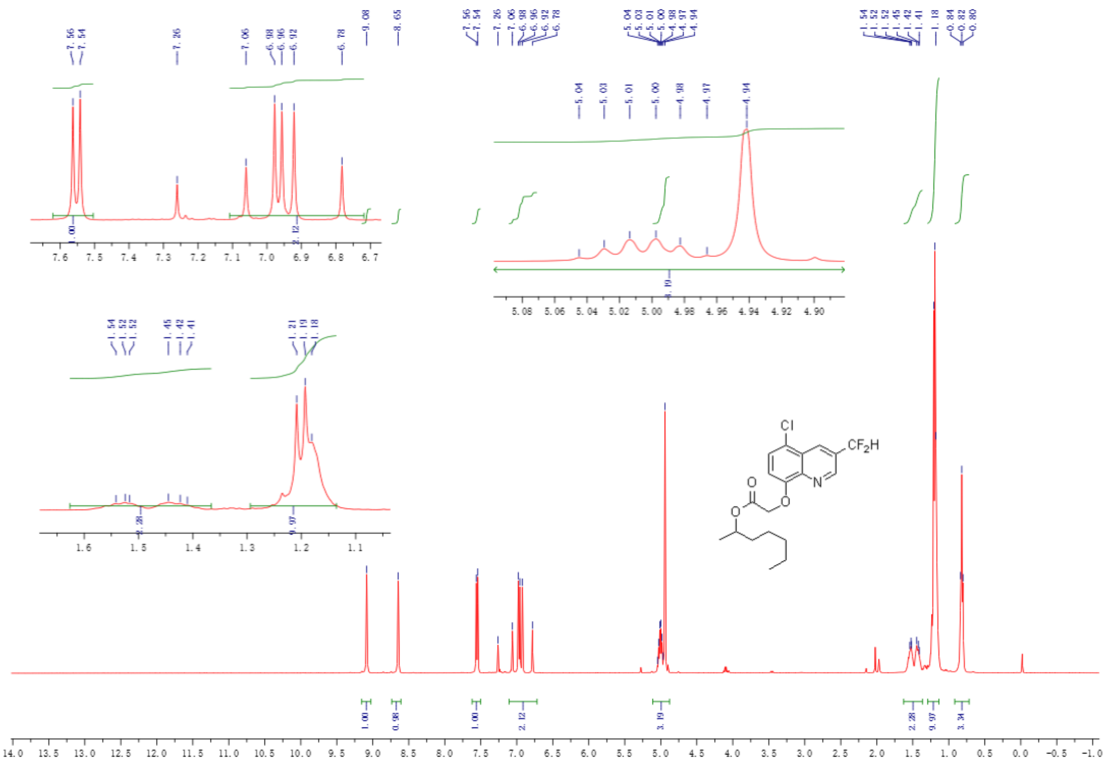

^{19}F NMR (376 MHz, $CDCl_3$) heptan-2-yl 2-((5-chloro-3-(difluoromethyl) quinolin-8-yl)oxy)acetate 6b

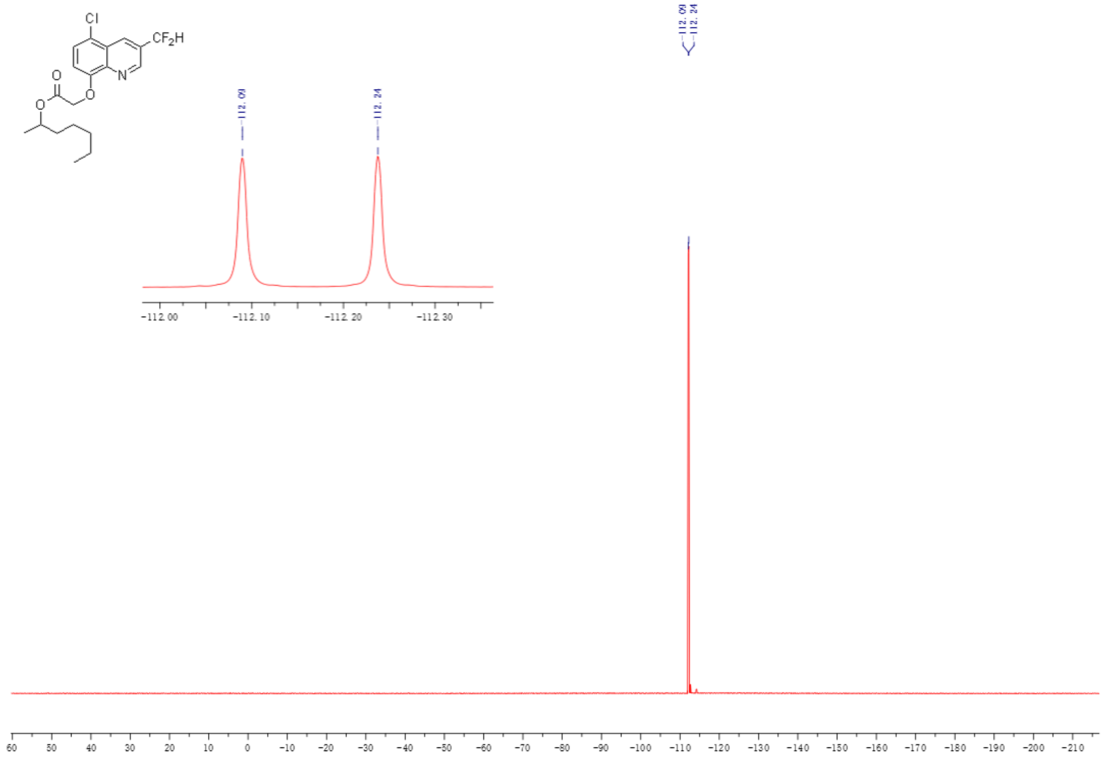

^{13}C NMR (101 MHz, $CDCl_3$) heptan-2-yl 2-((5-chloro-3-(difluoromethyl) quinolin-8-yl)oxy)acetate 6b

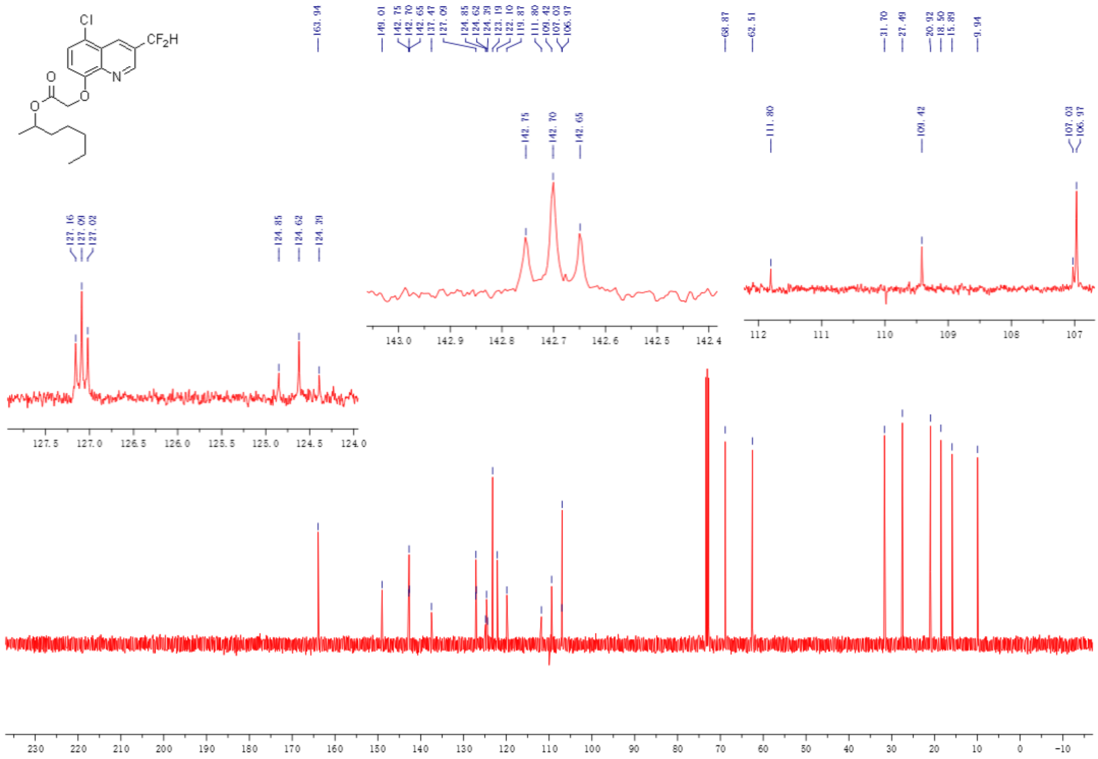

^{1}H NMR (400 MHz, $CDCl_3$) 2,5,7,8-tetramethyl-2-(4,8,12-trimethyltridecyl) chroman-6-yl 6-bromonicotinate

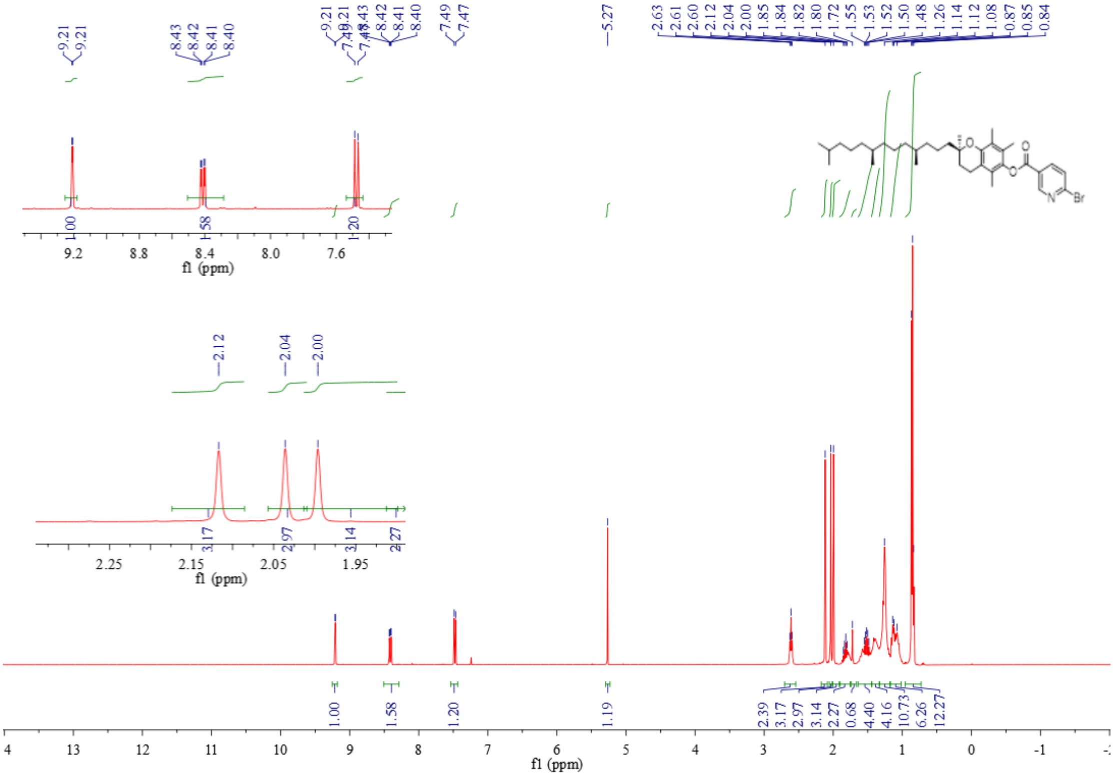

^{13}C NMR (101 MHz, $CDCl_3$) (2,5,7,8-tetramethyl-2-(4,8,12-trimethyltridecyl) chroman-6-yl 6-bromonicotinate

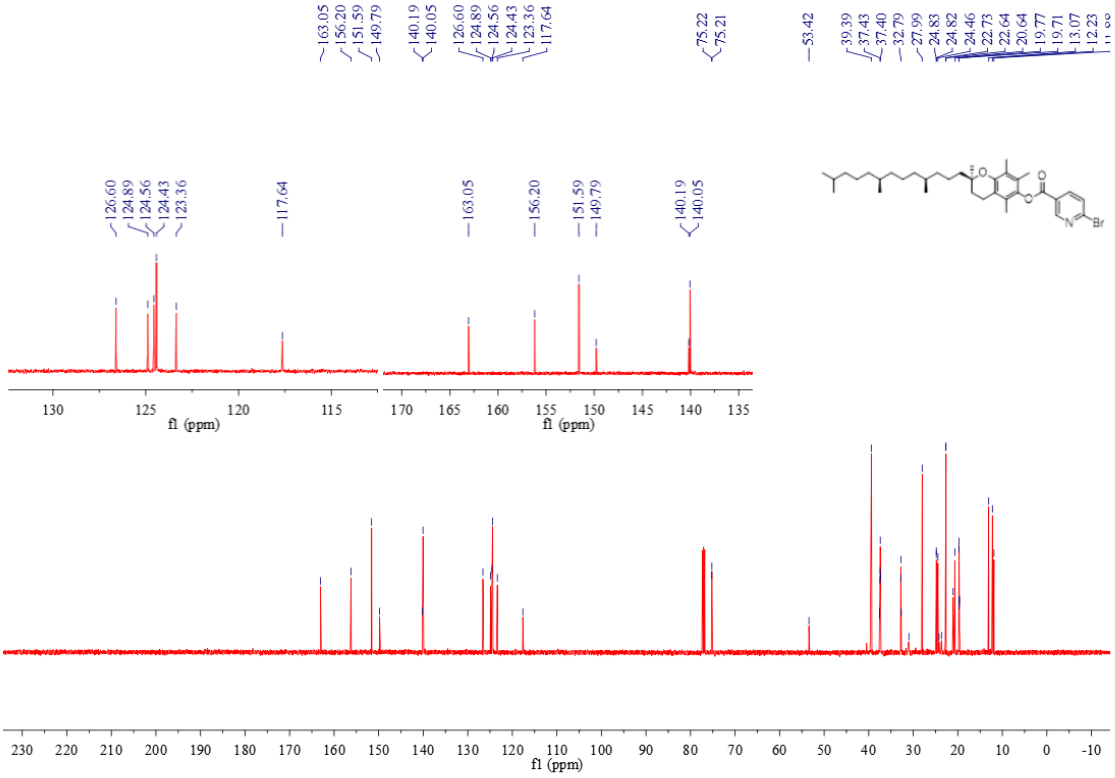

^{1}H NMR (400 MHz, $CDCl_3$) (*S*)-2,5,7,8-tetramethyl-2-((4*R*,8*R*)-4,8,12 –trimethyl tridecyl)chroman-6-yl-6-(difluoromethyl)nicotinate 6c

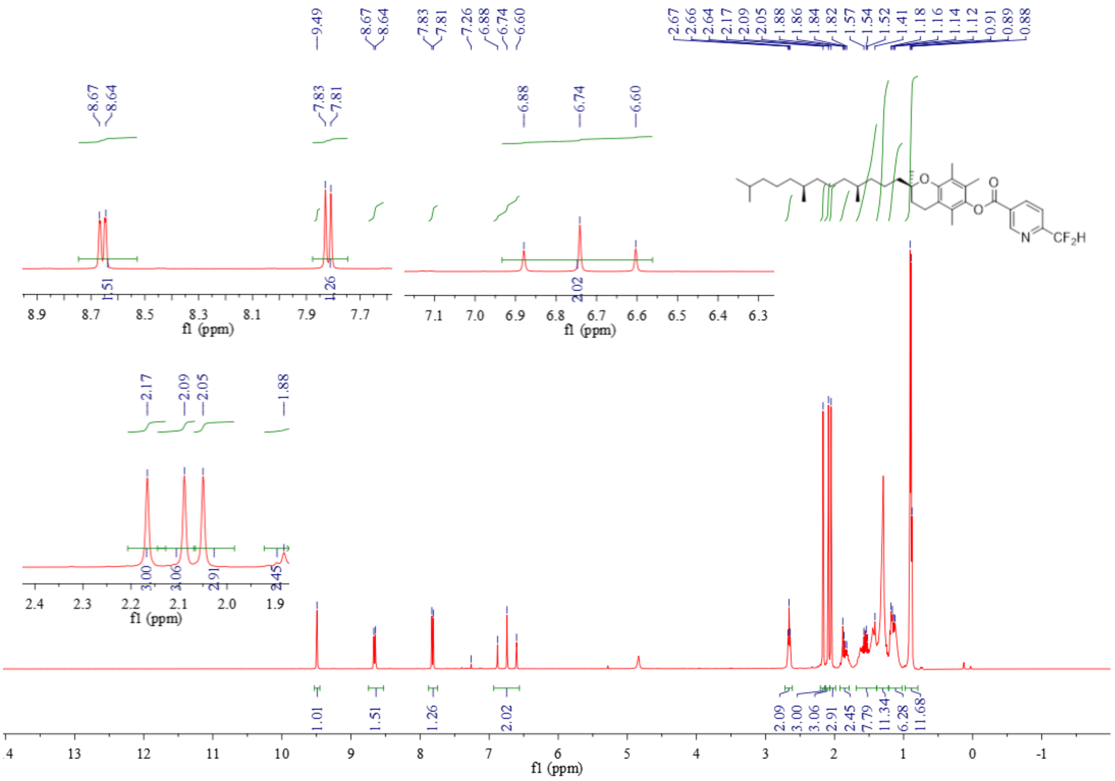

^{19}F NMR (376 MHz, $CDCl_3$) (*S*)-2,5,7,8-tetramethyl-2-((4*R*,8*R*)-4,8,12-trimethyltridecyl)chroman-6-yl-6-(difluoromethyl)nicotinate 6c

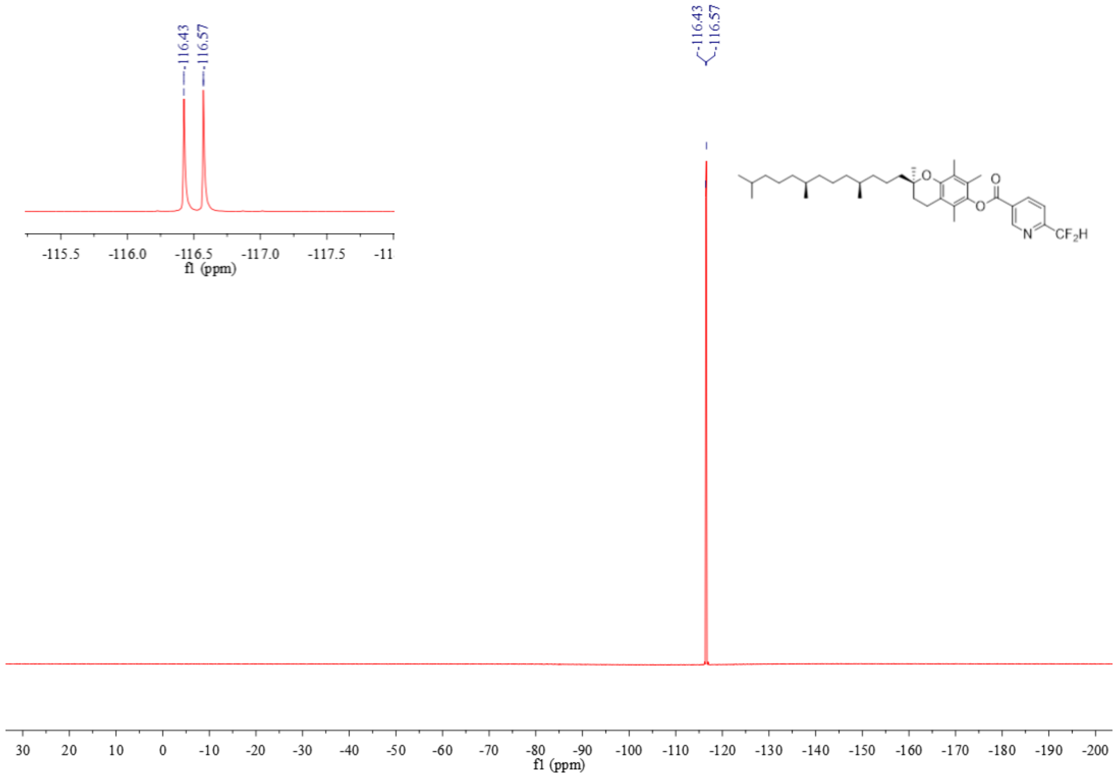

^{13}C NMR (101 MHz, $CDCl_3$) (*S*)-2,5,7,8-tetramethyl-2-((4*R*,8*R*)-4,8,12-trimethyltridecyl)chroman-6-yl-6-(difluoromethyl)nicotinate 6c

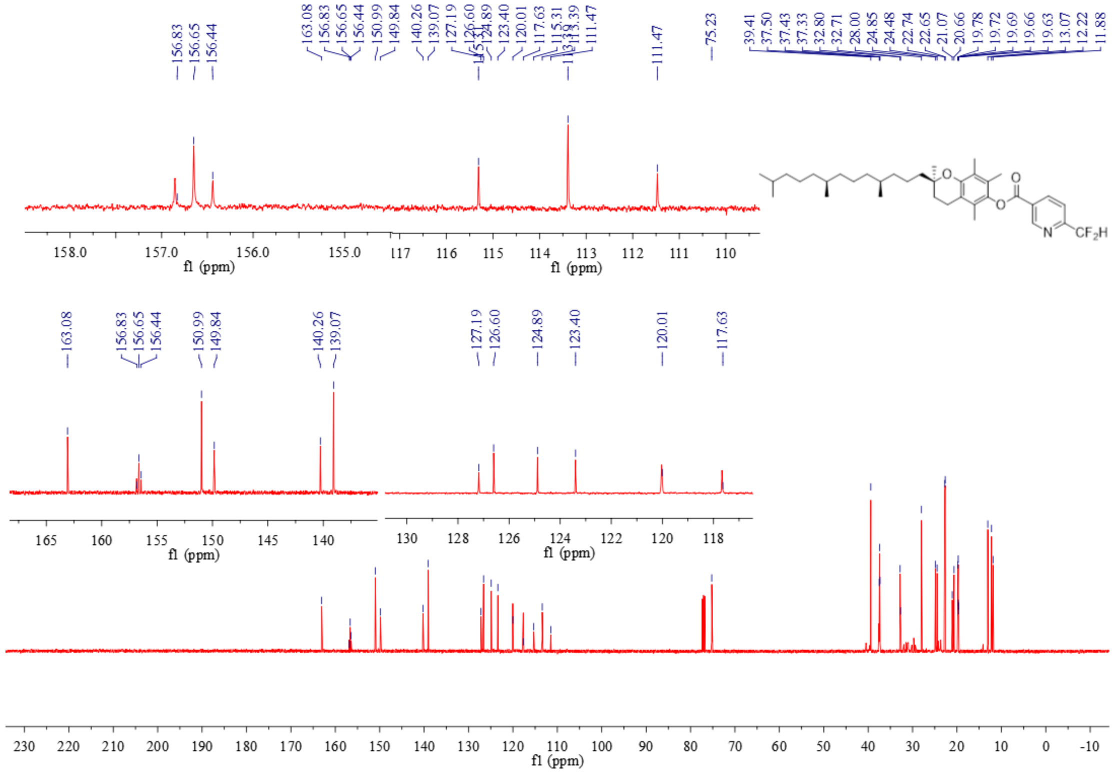

Supplement: Supplementary file 1 [file SC-008-C7SC00691H-s001.pdf]
